# Impact of Heterocycle Annulation on NIR Absorbance in Quinoid Thioacene Derivatives

**DOI:** 10.1002/chem.202200478

**Published:** 2022-03-29

**Authors:** Peng Hou, Sebastian Peschtrich, Nils Huber, Wolfram Feuerstein, Angela Bihlmeier, Ivo Krummenacher, Roland Schoch, Wim Klopper, Frank Breher, Jan Paradies

**Affiliations:** ^1^ Chemistry Department Paderborn University Warburger Straße 100 33098 Paderborn Germany; ^2^ Institute of Physical Chemistry Karlsruhe Institute of Technology (KIT) Kaiserstraße 12 76131 Karlsruhe Germany; ^3^ Institute of Inorganic Chemistry Karlsruhe Institute of Technology (KIT) Engesserstraße 15 76131 Karlsruhe Germany; ^4^ Institute of Inorganic Chemistry University of Würzburg Am Hubland 97074 Würzburg Germany

**Keywords:** density functional theory, EPR spectroscopy, heteroacenes, UV spectroscopy, radicals

## Abstract

The synthesis and characterisation of a homologous series of quinoid sulfur‐containing imidazolyl‐substituted heteroacenes is described. The optoelectronic and magnetic properties were investigated by UV/vis, fluorescence and EPR spectroscopy as well as quantum‐chemical calculations, and were compared to those of the corresponding benzo congener. The room‐temperature and atmospherically stable quinoids display strong absorption in the NIR region between 678 and 819 nm. The dithieno[3,2‐*b*:2′,3′‐*d*]thiophene and the thieno[2′,3′:4,5]thieno[3,2‐*b*]thieno[2,3‐*d*]thiophene derivatives were EPR active at room temperature. For the latter, variable‐temperature EPR spectroscopy revealed the presence of a thermally accessible triplet state, with a singlet‐triplet separation of 14.1 kJ mol^−1^.

## Graphical abstract

**A homologous series** of quinoid bisimidazolylidene‐derived thioacenes has been synthesised and investigated for their optoelectronic and magnetic properties. All investigated small‐band‐gap quinoids show stability at room temperature in the presence of oxygen and display strong NIR absorption. Three‐ and fourfold annulated quinoids display partially open‐shell singlet diradical character.

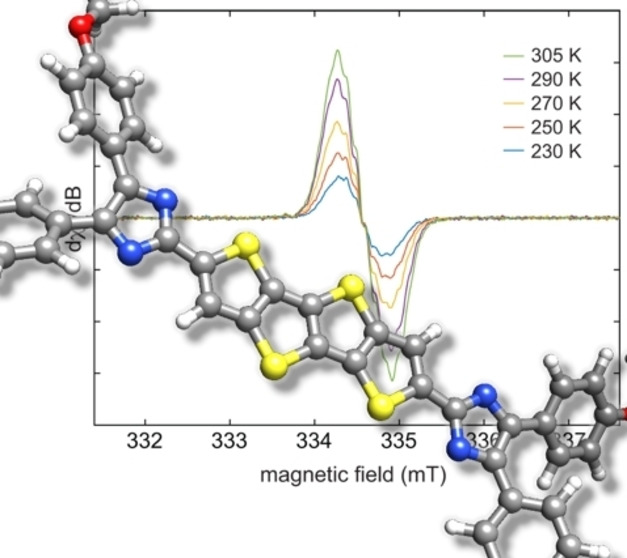

## Introduction

**Scheme 1:**
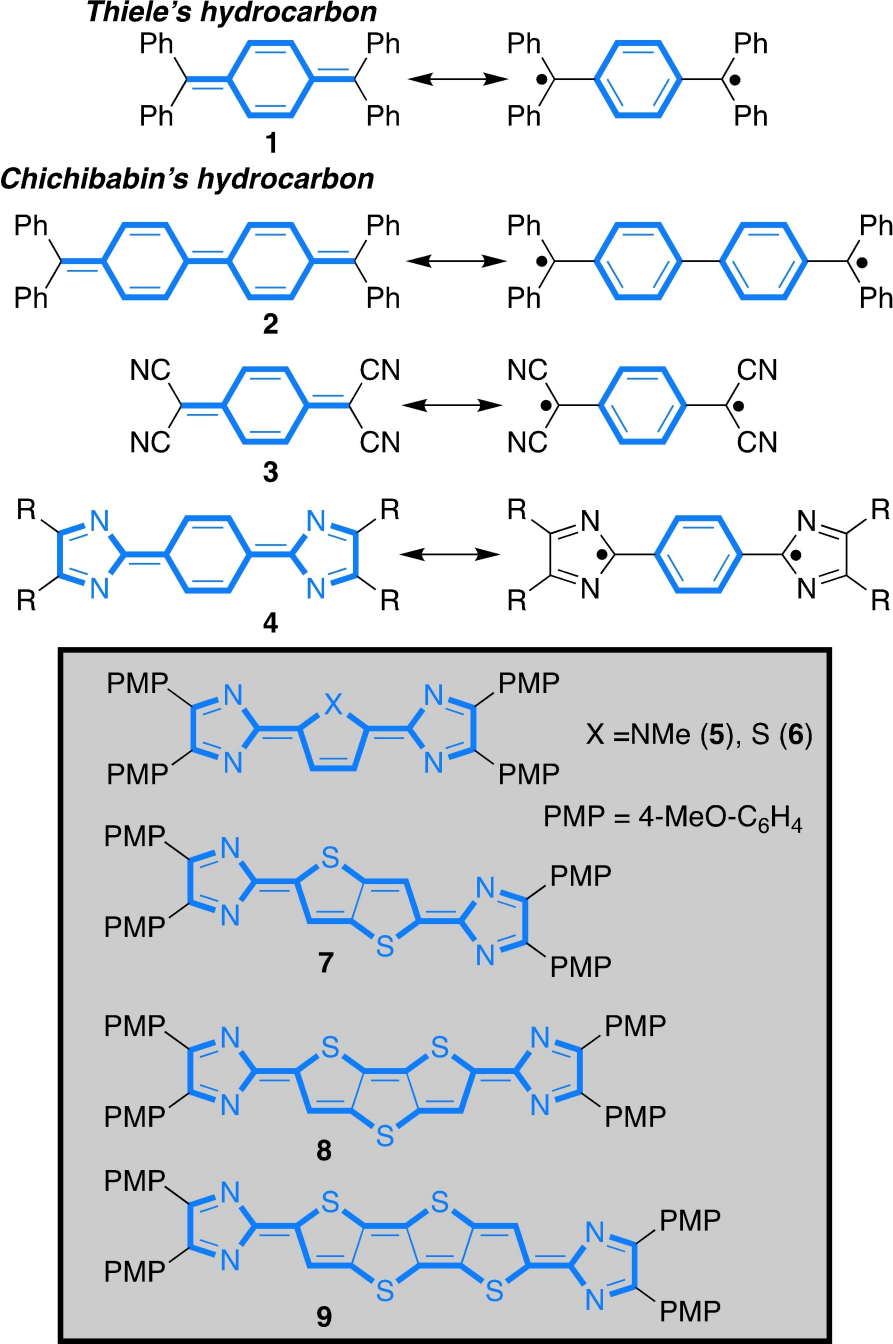
Quinoid structures (PMP=4‐methoxyphenyl).

Narrow‐band‐gap materials^[1]^ are applied in various fields of organic electronics because they exhibit interesting optical^[2]^ and magnetic properties.^[3]^ Conjugated polymers with alternating thiophene units are recognised as substances with enhanced materials properties, like thermal and oxidation stability, compared to the carbocyclic analogues.[^1c^, ^4^] For example, related materials with narrow band gaps have been used to harvest sunlight in the NIR region in order to increase the efficiency of organic solar cells.[^4^, ^5^] The incorporation of quinoid structures into π‐conjugated molecules or polymers provides high‐lying HOMO (highest occupied molecular orbital) and low‐lying LUMO (lowest unoccupied molecular orbitals) levels, giving rise to small band gaps.^[6]^ However, increasingly small band gaps in quinoid structures often result in diradical systems^[7]^ with loss of NIR absorption and increase in radical reactivity. Prominent examples of the latter are Thiele's (**1**) and Chichibabin's (**2**) hydrocarbons (Scheme 1).^[8]^

The diradical form features properties of open‐shell molecules, in which the electrons are not completely assigned in pairs to one orbital.^[7c]^ However, by extending the π‐system of the quinoid diradicals a remarkable oxygen and heat stability was achieved, making these compounds applicable as semiconductors.^[9]^ The stabilisation of the quinoid resonance form is ensured by introducing malononitrile acceptor groups in **3**, which in turn drastically decrease the HOMO level. Similarly, the imidazolylidene moiety is also capable of stabilising the quinoid resonance form of the benzo‐derived hydrocarbon **4**. In contrast to the malononitrile group, the electronic properties of the imidazole moiety can be readily modulated by peripheral substituents^[10]^ in order to achieve the desired electronic state (compare **3** and **4**, Scheme 1).^[11]^ Nonetheless, radical properties remain persistent. The introduction of a central heterocycle lead to the stable closed‐shell quinoids **5** (X=NMe) and **6** (X=S, Scheme 1)^[12]^ as a result of the reduced aromaticity of the central heterocyclic core.^[13]^ Despite their interesting optical properties, for example, strong NIR absorption of up to 850 nm, higher oligomers or annulated congeners^[14]^ remain so far unexplored so far. Successive extension of **6** by more thiophene units should have significant impact on the optical and electronic properties, potentially leading to strongly red‐shifted NIR‐absorbing materials with increased thermo and oxygen stability.

With this in mind we set out to probe the impact of increasing size of the heteroacene core on the optical and electronic properties of quinoids by synthesising and investigating the imidazolylidene substituted thiophene, thieno[3,2‐*b*]thiophene, dithieno[3,2‐*b*:2′,3′‐*d*]thiophene and thieno[2′,3′:4,5]thieno[3,2‐*b*]thieno[2,3‐*d*]thiophene derivatives **6**–**9**.

## Results and Discussion

### Synthesis

**Scheme 2:**
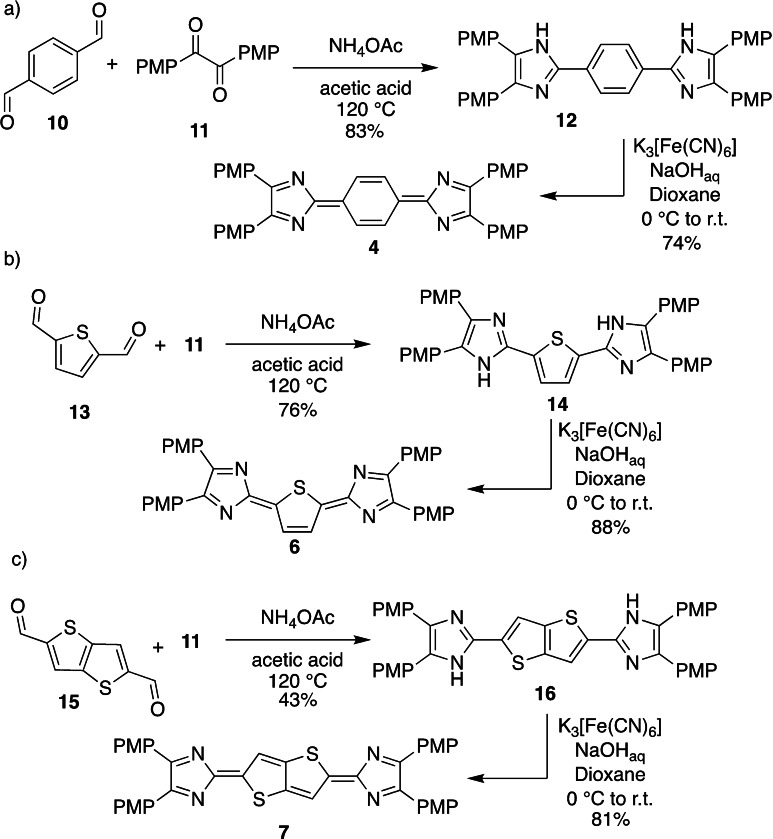
Synthesis of quinoid thiophene and thieno[3,2‐*b*]thiophene derivatives **6** and **7** (PMP=4‐methoxyphenyl).

To provide a good comparison of the sulfur‐containing extended heteroacenes we synthesised the benzo and thiophene derivatives **4** and **6** according to literature procedures (Scheme 2a and b).

**Figure 1:**
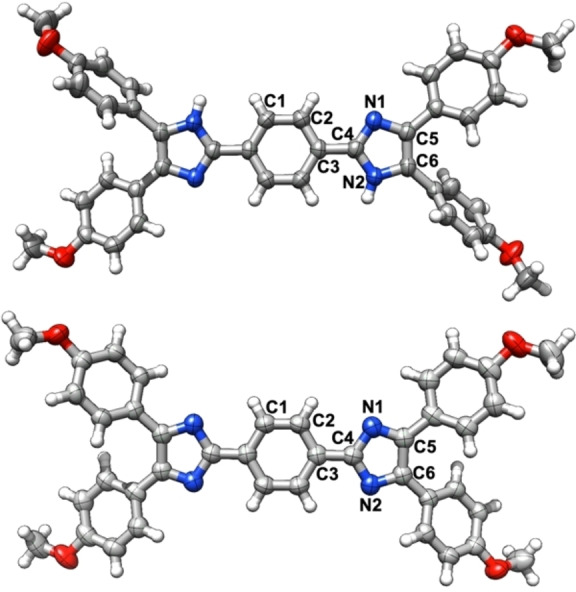
Molecular structure of **12** (top) and **4** (bottom); selected bond lengths of **12**: two THF molecules were omitted for clarity; C1−C2 1.3889(16) Å; C2−C3 1.4030(16) Å; C3−C4 1.4650(16) Å; C4−N1 1.3240(15) Å; N1−C5 1.3848(15) Å; N2−C6 1.3760(15) Å; C5−C6 1.3803(17) Å; selected bond lengths of **4**: C1−C2 1.359(2) Å; C2−C3 1.424(3) Å; C3−C4 1.404(2) Å; C4−N1 1.3844(19) Å; N1−C5 1.3213(19) Å; N1−C6 1.327(2) Å; C5−C6 1.486(2) Å.

Despite the significant interest in benzo‐derived quinoids,[^10^, ^11^] the synthesis and characterisation of the *p*‐methoxyphenyl derivative **4** has only been rudimentary described.^[10b]^ However, **4** is readily synthesed by a Debus‐Radziszewski reaction[^12a^, ^15^] of terephthalaldehyde (**10**) with *p*‐anisil (**11**) in 83 % yield, followed by oxidation of the bisimidazole intermediate **12** with ferrous cyanide in 74 % yield. The latter reaction was accompanied by a dramatic colour change from pale yellow to deep blue (for UV/vis spectra, see below). Single crystals suitable for X‐ray diffraction of **12** and **4** were obtained from concentrated THF and ethanol solutions, respectively. The molecular structures (Figure 1) support the formation of the quinoid structure of **4** as evidenced from the reduced bond length of C3−C4 to 1.404(2) Å compared to the bisimidazole precursor **12** (C3−C4 1.4650(16) Å). The central six‐membered ring of **4** displays alternating bond lengths of 1.359(2) Å (C1−C2) and 1.424(3) Å (C2−C3), whereas the unoxidised entity (**12**) features bond lengths typical for benzoid structures (C1−C2 1.3889(16) Å; C2−C3 1.4030(16) Å; C3−C4 1.4650(16) Å).^[16]^ Furthermore, C5−C6 is about 0.1 Å shorter in **12** than in **4**, whereas the shorter bond lengths N1−C5 and N2−C6 in **4** account for localised double bonds. The quinoid **6** was synthesised in two steps from **13** according to the method described by Nakamura (Scheme 2b)^[12a]^ and was adapted for the straightforward synthesis of the corresponding thieno[3,2‐*b*]thiophene derivative **7** from **15** (Scheme 2c). The reaction of the bisaldehyde **13** with *p*‐anisil (**11**) in the presence of ammonium acetate provided **14** in 43 % yield. The formation of the bisimidazole **14** was supported by the new ^1^H NMR resonances at *δ*=12.79 and 7.92 ppm ([D_6_]DMSO) for the N*H* and thiophene C*H* protons, respectively. The poor solubility of **14** in DMSO did not allow the determination of ^13^C NMR chemical shifts. The oxidation of **14** and **16** to the corresponding quinoid heteroacenes **6** and **7** was achieved by the reaction with alkaline ferrous cyanide in 1,4‐dioxane. The successful formation of the quinoids **6** and **7** was corroborated by the dramatic colour change from yellow to green (for spectroscopic details see below) and both materials were isolated in 88 and 81 % yield, respectively. The corresponding ^1^H NMR (CDCl_3_) spectra of **6** and **7** exhibit a singlet at *δ*=8.29 and 8.40 ppm for the thiophene C*H* proton. Finally, ESI mass spectrometry stand in line with the successful oxidation of both bisimidazoles to the corresponding quinoids.

**Scheme 3:**
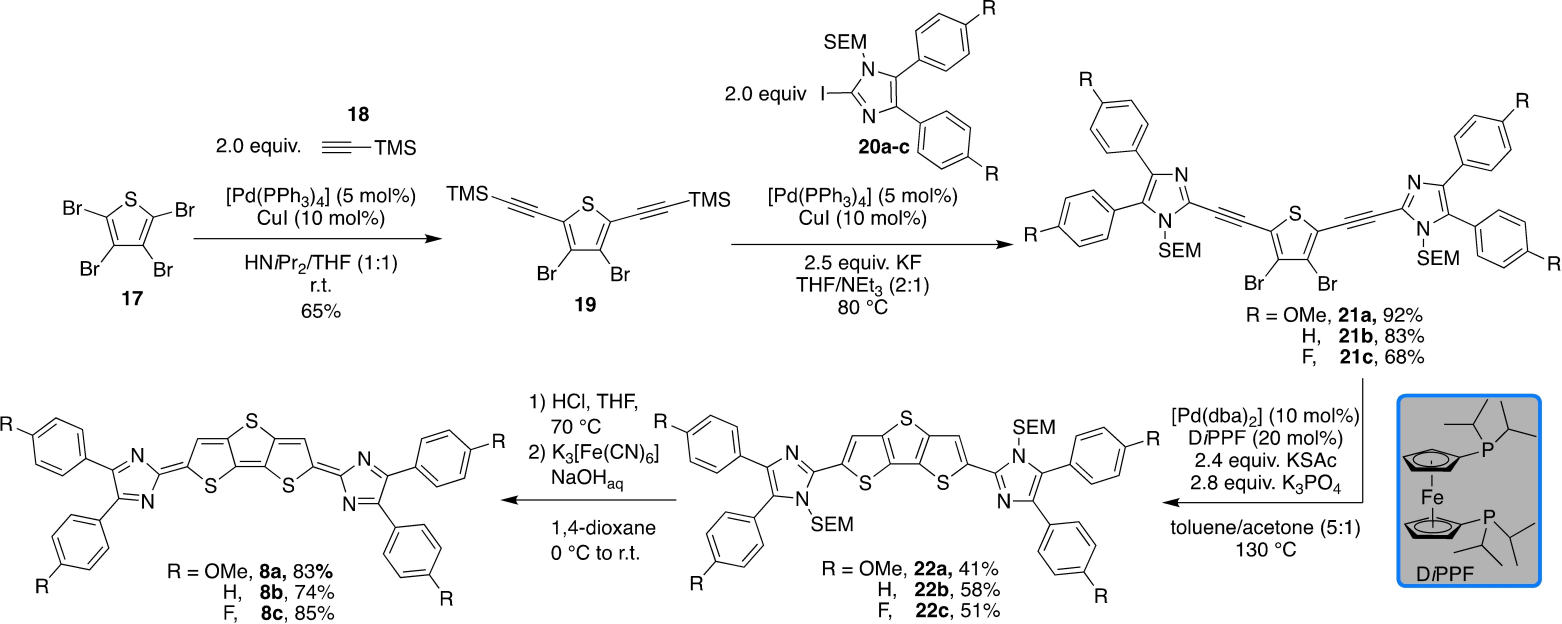
Synthesis of quinoid derivatives **8 a**–**c**.

**Scheme 4:**
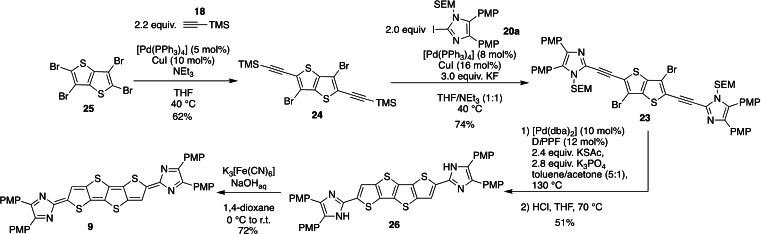
Synthesis of quinoid thieno[2′,3′:4,5]thieno[3,2‐*b*]thieno[2,3‐*d*]thiophene derivative **9** (PMP=4‐methoxyphenyl; SEM=trimethylsilylethoxymethyl).

The three‐ and fourfold condensed sulfur heteroacenes **8** and **9** were constructed through the domino carbon−sulfur cross‐coupling/cyclisation sequence developed in our group (Schemes 3 and 4).^[17]^ The four‐step synthesis of **8 a**–**c** commences by the reaction of tetrabromo thiophene (**17**) with trimethylsilyl‐protected (TMS) acetylene (**18**) under Sonogashira conditions and provided the 2,5‐bisalkyne **19** in 65 % yield as single regioisomer. At this stage, we selected three imidazole derivatives with different para substitution pattern (**20 a** R=OMe, **20 b** R=H, **20 c** R=F) to study the electronic impact of the imidazole moiety on the optical and electronic properties. In situ alkyne deprotection with potassium fluoride and subsequent Sonogashira coupling with each of the three trimethylsilylethoxymethyl‐protected (SEM) 2‐iodoimidazoles **20 a**–**c** provided the internal alkynes **21 a**–**c** in 68 to 92 % yield. SEM‐protection of the imidazole throughout the synthesis was necessary because otherwise the poor solubility of the imidazoles and – at a later stage – of the bisalkyne derivatives prevented clean reactions with high conversion. The formation of the bisalkyne **21 b** was unambiguously supported by X‐ray diffraction analysis (see the Supporting Information). Treatment of the bisalkynes **21 a**–**c** with potassium thioacetate in the presence of [Pd(dba)_2_]/D*i*PPF (dba=dibenzylideneacetone; D*i*PPF=1,1’‐bis(diisopropylphosphino)ferrocene) and potassium phosphate as base furnished the dithieno[3,2‐*b*:2′,3′‐*d*]thiophene congeners **22 a**–**c** in 41, 58 and 51 % yield, respectively. Since the deprotection of **22 a**–**c** led to insoluble materials, the latter were directly subjected to oxidation using ferrous cyanide/NaOH, again accompanied by a drastic colour change from brown/orange to dark blue. Thus, the quinoid materials **8 a**–**c** were obtained in 83, 74 and 85 % yield as black (**8 a**, **8 c**) or dark brown (**8 b**) solids. NMR spectroscopic data could not be obtained due to the insolubility of the materials, but ESI mass spectrometry confirmed the two‐electron oxidation by detection of the corresponding [*M*+H]^+^ cationic species.

Finally, the thieno[2′,3′:4,5]thieno[3,2‐*b*]thieno[2,3‐*d*]thiophene derivative **9** was synthesised according to a similar strategy using the thieno[3,2‐*b*]thiophene bisalkyne **23** as cyclisation precursor (Scheme 4). This intermediate was accessed in 74 % yield from the corresponding TMS‐protected bisalkyne **24**, which is readily available by Sonogashira coupling of the tetrabromo thieno[3,2‐*b*]thiophene **25** with **18**. Subsequent reaction with KSAc and [Pd(dba)_2_]/D*i*PPF induced the C−S cross‐coupling and cyclisation. The tetraheteroacene was deprotected in situ by treatment with HCl in THF to obtain **26** as red solid in 51 % yield. Again, the low solubility of **26** in common polar organic solvents did not allow the acquisition of NMR spectroscopic data but high resolution molecular mass spectrometry of **26** confirmed the successful synthesis of the oxidation precursor. The oxidation of **26** to the quinoid **9** was achieved by aqueous ferrous cyanide under basic conditions. The product was isolated as dark green powder in 72 % yield which evaded NMR spectroscopic analysis due to insolubility.

### Spectroscopic investigation

**Figure 2:**
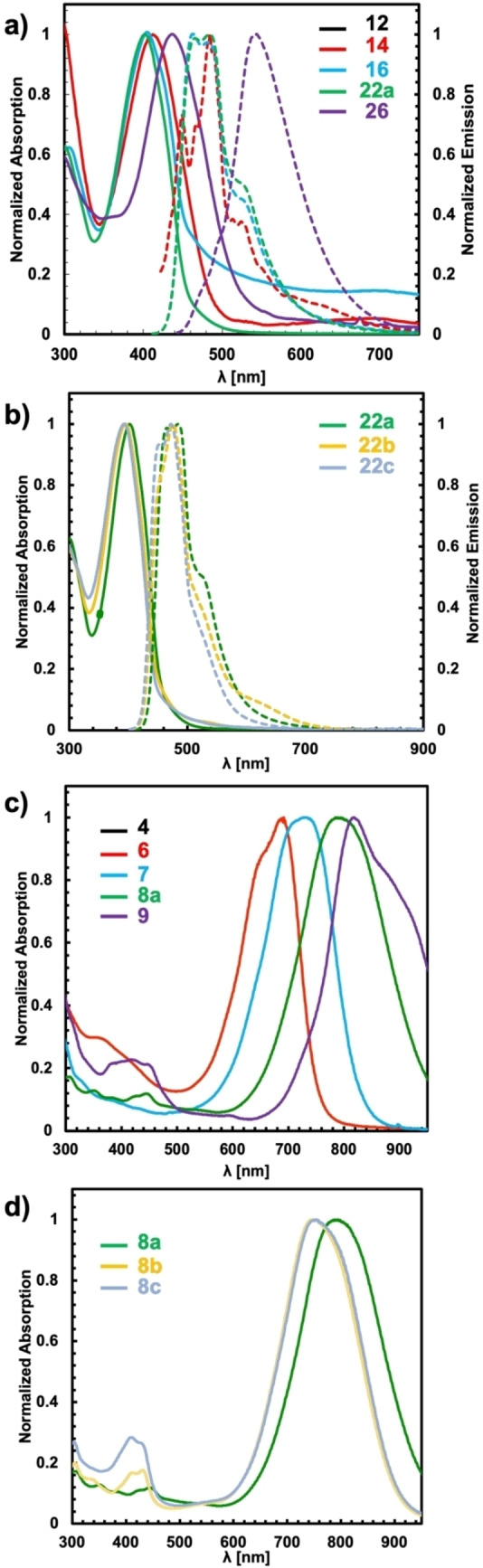
UV/vis spectroscopic data of a) and b) bisimidazole derivatives and c) and d) quinoid derivatives (solid lines correspond to absorption spectra; dashed lines correspond to emission spectra).

The bisimidazole derivatives as well as the quinoid heteroacenes were investigated by UV/vis and fluorescence spectroscopy. Spectra were recorded as 6.8–23.4 μM solutions in CH_2_Cl_2_ at 25 °C. The spectra and results are summarised in Table 1 and Figure 2. The thiophene derivative **14** displayed a red shift of 39 nm compared to the parent benzo derivative **12**. The absorption maximum for the homologue series of the oxidation precursors **14**, **16**, **22 a** and **26** was found between 402 and 436 nm (3.1 and 2.8 eV), which is attributed to a typical π‐π* transition (Figure 2a). The extension of the heterocenes has a small effect on the optical gap (Δ*E*_opt_) of the bisimidazole derivatives (Table 1). As expected, Δ*E*_opt_ decreases from 2.8 eV to 2.3 eV with increasing size of the heterocyclic core. The influence of the peripheral phenyl substituents on the electronic properties of the heteroacene core was probed by electron‐rich (R=OMe, **22 a**), electron‐neutral (R=H, **22 b**) and electron‐withdrawing (R=F, **22 c**) groups in the periphery. The change from a donor group (*p*‐OMe−C_6_H_4_, **22 a**) to a neutral substituent (C_6_H_5_, **22 b**) to an electron‐withdrawing substituent (*p*‐F−C_6_H_4_, **22 c**) results in a subtle blue shift of *λ*_max_ from 402 nm (3.08 eV, **22 a**) to 395 nm (3.14 eV, **22 b**) to 391 nm (3.17 eV, **22 c**; Figure 2b). All imidazole derivatives display fluorescence when irradiated at the *λ*_max_ edge. The emission is shifted to higher wavelength by 80 to 100 nm; this suggests only small structural differences between the ground and excited states.

**Table 1:** Summarised spectroscopic data of thioacenes and quinoid thioacenes.

Cmpd	*λ*_max_(CH_2_Cl_2_) [nm]	log *ϵ* (*λ*_max_) [M^−1^ cm^−1^]	*λ*_max‐FL_(CH_2_Cl_2_) [nm]	*λ*_ons_ [nm]	Δ*E*_opt_ [eV]	U_ox_ [V]	*E*_HOMO_ [eV]^[b]^	*E*_LUMO_ [eV]^[c]^
**12**	373	4.53	450	421	3.0	−0.35	−4.8	−1.8
**14**	412 (lit. 391)	4.42	470	440	2.8	0.52	−5.6	−2.8
**16**	404	4.65	483	492	2.5	0.43	−5.5	−3.0
**22 a** ^[a]^	402	4.48	484	461	2.7	0.40	−5.5	−2.8
**22 b** ^[a]^	395	4.66	476	458	2.7	0.54	−5.6	−2.9
**22 c** ^[a]^	391	4.28	472	453	2.7	0.71	−5.8	−3.1
**26**	436	4.65	544	543	2.3	0.00	−5.1	−2.8
**4**	678	4.95	–	778	1.6	−0.44	−4.7	−3.1
**6**	688	4.96	–	780	1.6	0.66	−5.8	−4.2
**7**	737	4.69	–	875	1.4	0.40	−5.5	−4.1
**8 a**	797	4.99	–	958	1.3	0.25	−5.4	−4.1
**8 b**	750	4.77	–	904	1.4	0.47	−5.6	−4.2
**8 c**	749	4.89	–	907	1.4	0.61	−5.7	−4.3
**9**	819	4.84	–	1090	1.1	0.07	−5.1	−4.1

[a] After SEM‐deprotection; [b] *E*_HOMO_=−(U_Ox_⋅e+5.1) eV referenced to ferrocene/ferrocenium (Fc/Fc^+^) couple;^[19]^ [c] *E*_LUMO_= Δ*E*_opt_+*E*_HOMO_.

The electronic structure changes significantly upon oxidation to the corresponding quinoid heteroacenes. The oxidation process is accompanied in all cases by a significant colour change of the reaction mixtures. Furthermore, oxidation leads to a loss of the fluorescence properties. UV/vis spectroscopy clearly shows the presence of an intense absorption band in the NIR region for all seven quinoid molecules (Figure 1c and d). In accordance with literature reports, we observed for **6** a broad absorption at 688 nm (1.80 eV) with large molar absorption coefficient (log *ϵ*=4.96).^[12a]^ Interestingly, the benzo derivative **4** exhibits similar absorption properties (*λ*_max_=678 nm (1.83 eV), log *ϵ*=4.95), indicating that the NIR‐absorption is only marginally influenced by the nature of the central acene moiety. The *λ*_max_ of the new thieno[3,2‐*b*]thiophene‐derived quinoid **7** is significantly red‐shifted to the NIR region to 737 nm (1.68 eV) with a significantly smaller molar absorption coefficient of log *ϵ*=4.69 compared to **6** (log *ϵ*=4.96). Further extension of the heteroacene core to the dithienothiophene‐derivative **8 a** causes a red‐shift by 60 to 797 nm (1.56 eV, log *ϵ*=4.99). The fourfold annulated system **9** displays the strongest red‐shift to 819 nm (1.51 eV) in comparison to the thiophene derivative **6**, with a comparable high molar absorption coefficient of log *ϵ*=4.84. The two‐electron oxidation to the quinoids results in a significant decrease of Δ*E*_opt_ by about one‐half. The trend of bigger heteroacene cores leading to smaller Δ*E*_opt_ is retained (cf. Table 1). Remarkably, the estimated HOMO energies of the bisimidazol and quinoids are very similar but the LUMO energies change dramatically for the oxidised species. In the thiophene series **6**,**7**,**8 a**, **9**, the energy level of the HOMO is raised by increased conjugation whereas the LUMO energy remains largely unaffected. The impact of the peripheral substituents on the optical properties of the quinoids was probed for the dithienothiophenes series **8 a**–**c**. The phenyl (**8 b**) and the fluoro derivative **8 c** exhibit both almost identical optical properties (Figure 2d and Table 1), including comparable extinction coefficients (log *ϵ*=4.77 and 4.89). In contrast, the methoxy substituted derivative **8 a** displayed an NIR absorption close to 800 nm (1.56 eV, log *ϵ*=4.99). This indicates that the substituent has a significant impact on both the position of the absorption maximum and on the extinction coefficients.

We analysed the compounds **7**, **8 b/c**, and **9** in solution and in the solid state by EPR spectroscopy (X‐band). Note that the EPR spectra of molecules such as Chichibabin's hydrocarbon **2** (Scheme 1) have been subject to very controversial discussions.[^8c^, ^18^] For diradicals of this type[^7c^, ^11b^, ^20^] it has been frequently observed that they exhibit a doublet EPR signal, which corresponds to a monoradical species. This observation is in line with a vivid discussion in the literature on the so‐called “diradical paradox”.^[21]^ Doublet EPR signatures from diradicals may result from self‐aggregation or monoradical impurities, for instance. These doublet signals may mask the weak intensity expected for a triplet diradical. The latter might also be not detectable due to a thermal equilibrium with a closed‐shell molecule or open‐shell singlet diradical.^[11d]^

**Figure 3:**
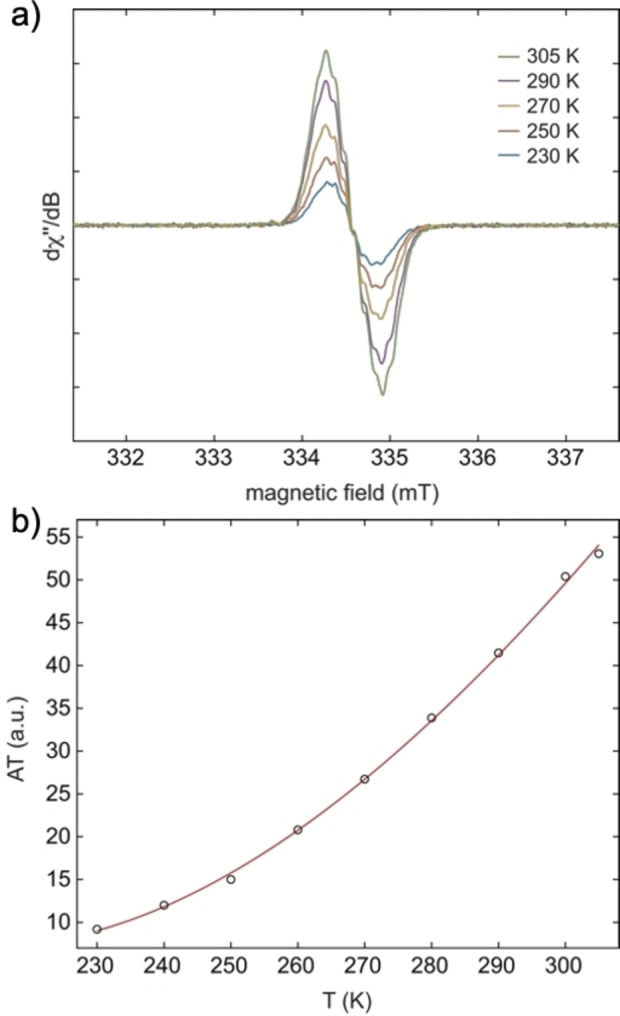
a) Variable‐temperature X‐band EPR spectra of **9** in tetrahydrofuran between 230 and 305 K; b) representation of the temperature dependence of the double integral EPR intensity (*A*) of **9** in tetrahydrofuran solution. Circles (○) represent the experimental results and the red line corresponds to the fit with the Bleaney‐Bowers equation.^[21]^

Indeed, variable‐temperature X‐band EPR spectra of solid **7** between 230 and 300 K show a signal around *g*=2.004 and a decrease in signal intensity with increasing temperature, which would be consistent with an *S*=1/2 impurity (Figure S1 of the Supporting Information). In THF solution, an additional monoradical impurity with proton hyperfine couplings of *a*(^1^H)=32 MHz (11 G, 3H) and 5 MHz (2 G, 2H) was detected (Figure S2). For **8 c** in THF between 230 and 305 K, a sharp doublet EPR signal was observed with an additional broad signal, which likely corresponds to a thermally populated triplet state (Figures S3 and S4). A detailed analysis, however, failed due to strong line broadening. The diradical nature of **9** was unambiguously confirmed by EPR measurements in THF solution. Variable‐temperature experiments between 230 and 305 K show that the EPR signal intensity increases with increasing temperature (Figure 3). A larger temperature window could not be accessed due to increasing occurrence of side reactions at higher temperatures. Nonetheless, fitting of the temperature‐dependent double‐integral intensity to the Bleaney‐Bowers model^[21]^ gives a singlet‐triplet gap of 2 *J*=−1178 cm^−1^ (Δ*E*_ST_=14.1 kJ mol^−1^), indicating a thermally excited triplet state (Figure 3b).

Finally, the electrochemical properties of the diradicals were investigated by cyclic voltammetry. In contrast to **6**, the higher thiophene derivatives **7**–**9** displayed irreversible redox properties and allowed only the estimation of the HOMO energies (Table 1). Due to the poor electrochemical stability of **7**–**9**, further information was not extracted.

## Computational Investigation

The electronic structure of the benzo and thioacene quinoids was investigated by density functional theory (DFT) as implemented in the Turbomole package^[23]^ using the PBE0^[24]^ functional in combination with the def2‐TZVP^[25]^ basis set. We optimised the geometries for different orbital occupations using the restricted and unrestricted Kohn‐Sham formalism: closed‐shell (RKS), open‐shell triplet (UKS triplet) and open‐shell broken‐symmetry singlet (UKS singlet). The relative energies obtained from these calculations are summarised in Table 2.

**Table 2:** Relative energies (PBE0/def2‐TZVP) of closed‐shell and open‐shell states in kJ mol^−1^ (the value in parentheses corresponds to the respective *S* ^2^ expectation value).

Cmpd	RKS	UKS triplet	UKS singlet	LUMO occ.^[a]^	Δ*E*_ST_^[b]^
**4**	6.0	26.1 (2.037)	0.0 (0.623)	0.41	40.6
**6**	0.1	42.5 (2.033)	0.0 (0.090)	0.15	45.4
**7**	1.7	31.3 (2.035)	0.0 (0.410)	0.23	42.1
**8 a**	6.4	20.2 (2.037)	0.0 (0.688)	0.53	33.4
**8 b**	4.8	24.0 (2.038)	0.0 (0.614)	n.c.^[c]^	37.4
**8 c**	4.9	23.6 (2.037)	0.0 (0.619)	n.c.^[c]^	36.8
**9**	12.7	13.5 (2.040)	0.0 (0.846)	0.74	24.9

[a] LUMO occupation obtained from CASSCF(2,2)/def2‐TZVP calculations on PBE0/def2‐TZVP optimised UKS singlet geometries; [b] singlet‐triplet gaps in kJ mol^−1^ computed from corrected UKS singlet energies; [c] not computed.

We find that the UKS singlet is energetically most favoured for all investigated compounds. In the series of thioacene quinoids **6**–**9**, we observe a lowering of the UKS triplet energy relative to the UKS singlet energy, while the closed‐shell state becomes less favourable. Together with the *S* ^2^ expectation value (0.090 for **6** and 0.864 for **9**), this points to an increasing diradical character. The bond lengths of the optimised UKS singlet geometries further support this finding (see the Supporting Information for details).

For compound **6**, the bond lengths are similar to the ones of the RKS geometry, being in line with a closed‐shell resonance structure. In contrast, the bond lengths of compound **9** are closer to the ones of the UKS triplet geometry and thus more consistent with a diradical resonance structure.

**Figure 4:**
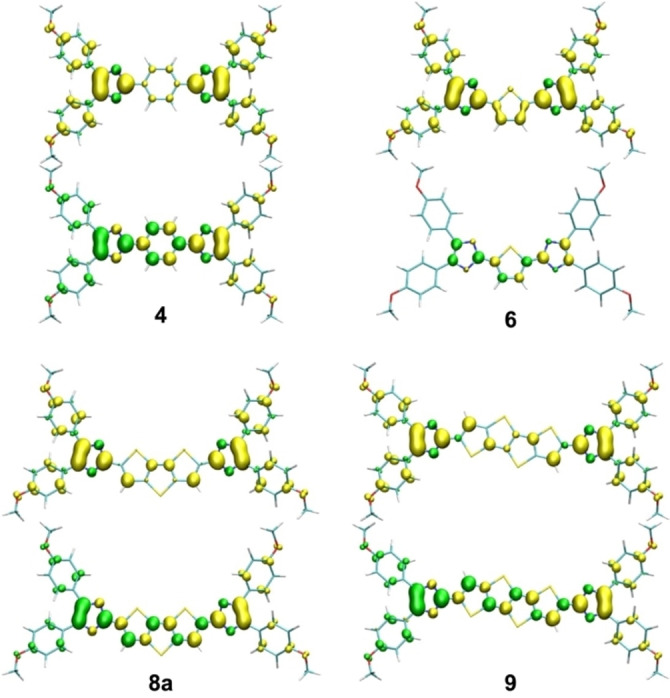
Spin densities (PBE0/def2‐TZVP) of the UKS triplet (top) and singlet (bottom) geometries (isovalue ±0.0036 bohr^−3^).

The spin densities of the UKS triplet and UKS singlet geometries are shown in Figure 4 for selected compounds. Their shape is in agreement with the position of the unpaired electrons in a diradical resonance structure. When we compare compounds **4** and **6**, we find that the UKS triplet spin densities look very similar but that the UKS singlet spin density of **6** is smaller in size, suggesting a smaller diradical character in the thiophene than in the benzo quinoid. Overall, the spin densities are mainly localised at the imidazolyl moieties, leading to a larger distance between the unpaired electrons in the series of compounds **6**–**9**.

In order to quantify the diradical character, complete active space self‐consistent field (CASSCF) calculations were performed on the UKS singlet geometries using the ORCA program.^[26]^ We used an active space of two electrons in two orbitals, that is, the HOMO and the LUMO. A pure singlet state can then be constructed from two configurations with either the HOMO or the LUMO being doubly occupied. In a perfect singlet diradical, both configurations contribute 50 % each, leading to an average LUMO occupation of 1 and consequently a diradical character of 100 %. The computed LUMO occupations of the investigated benzo and thioacene quinoids are given in Table 2. As already indicated by the singlet spin densities, the diradical character of the benzo quinoid **6** (41 %) is much larger than the one of the corresponding thiophene quinoid **4** (15 %). This can be attributed to the enhanced aromaticity of benzene compared to thiophene, thus favouring a diradical structure over a cross‐conjugated closed‐shell structure. We observe an increasing diradical character in the series of thioacene quinoids **6**–**9**, with the largest value for compound **9** (74 %).

According to the *S* ^2^ expectation value, the UKS singlet energy suffers from spin contamination errors in the wavefunction. It is therefore not well suited to compute the energy difference to the UKS triplet state. To obtain a better estimate, we corrected the UKS singlet energy using an approximate spin projection method.^[27]^ The singlet‐triplet gaps Δ*E*_ST_ evaluated with this approach are given in Table 2. It can be seen that a large diradical character is associated with a small singlet‐triplet gap. For compounds **6**–**9**, this is consistent with the above‐mentioned increasing distance between the unpaired electrons. Consequently, the smallest value for Δ*E*_ST_ is found for compound **9** (24.9 kJ mol^−1^). The trends for both the diradical character and the singlet‐triplet gaps match the findings of the EPR spectroscopic investigation. However, the computed value for the singlet‐triplet gap of compound **9** is larger than the experimentally derived one.

**Figure 5:**
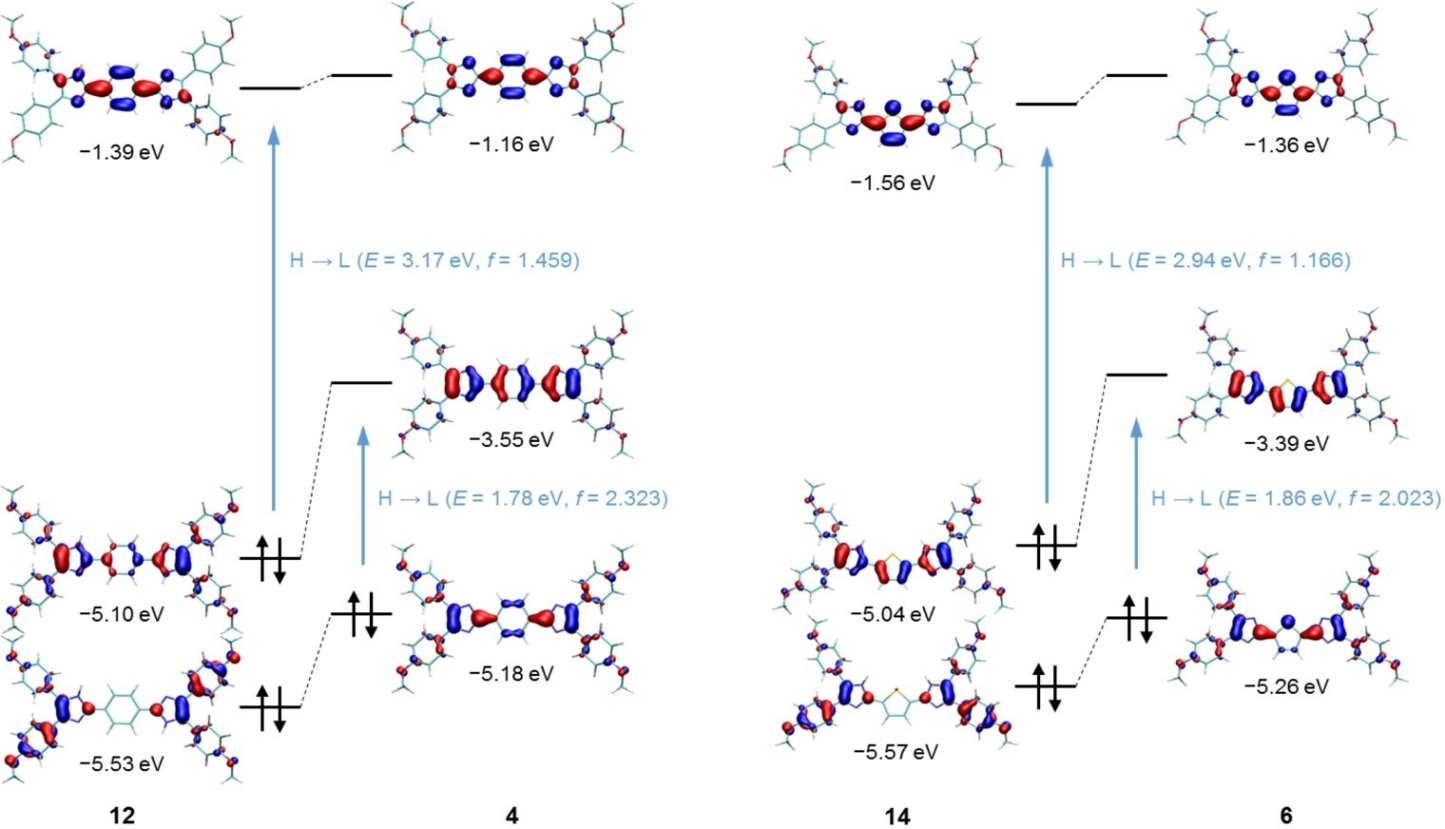
Frontier molecular orbitals (PBE0/def2‐TZVP) of the benzo and thiophene bisimidazole intermediates **12** and **14** as well as of the associated quinoids **4** and **6** (isovalue ±0.04 bohr^−3/2^). The given values correspond to RKS orbital energies (black) and to RKS excitation energies from TDDFT calculations (blue).

Furthermore, we investigated the optical properties of the synthesised compounds. In order to understand the experimentally observed red‐shift of the absorption maxima when comparing bisimidazole intermediates to quinoids, the frontier molecular orbitals of the respective benzo (**12**, **4**) and thiophene (**14**, **6**) derivatives are depicted in Figure 5.

Taking into account only the planar core structure, **12** and **14** can be classified as aromatic molecules with 18 electrons in the bonding π‐orbitals. Upon oxidation, the electrons in the HOMO are removed, so that 16‐π‐electron systems are obtained. The resulting HOMO‐LUMO gap is substantially smaller than for the aromatic molecules, thus qualitatively explaining the observed red‐shift in the absorption spectra. In addition, we computed the excitation energies and oscillator strengths in the framework of time‐dependent density functional theory (TDDFT). Based on the RKS results, the prominent absorption band in the spectra of **4**, **6**, **12**, and **14** can be assigned to the HOMO‐LUMO transition (H→L). Regarding the series of quinoid derivatives, TDDFT calculations were not only performed for the closed‐shell state (RKS) but also for the open‐shell broken‐symmetry singlet state (UKS singlet). In both cases, we essentially obtain one single excitation with significant oscillator strength (Table 3).

**Table 3:** TDDFT excitation energies and oscillator strengths (PBE0/def2‐TZVP) of the RKS and UKS singlet geometries.

Cmpd	RKS			UKS singlet	
	*E* [eV]	*f*	contribution	*E* [eV]	*f*
**4**	1.78	2.323	96.8 % H→L	1.80	1.641
**6**	1.86	2.023	97.5 % H→L	1.85	1.936
**7**	1.75	2.633	97.4 % H→L	1.72	2.172
**8 a**	1.58	2.692	97.1 % H→L	1.57	1.943
**8 b**	1.71	2.540	96.1 % H→L	1.67	1.911
**8 c**	1.69	2.546	96.3 % H→L	1.65	1.916
**9**	1.47	3.202	97.7 % H→L	1.49	1.980

This is the HOMO‐LUMO transition, being responsible for the NIR absorption band of all investigated quinoids. The excitation energies agree with the measured absorption maxima (*λ*_max_) within less than 0.1 eV, perfectly reproducing the experimentally observed trends regarding the extension of the thioacene core (**6**, **7**, **8 a**, **9**) as well as the influence of different substituents (**8 a**–**c**).

The oscillator strengths from the UKS singlet calculations are smaller than those from the RKS calculations. The reduction correlates well with the amount of diradical character. Besides, we performed TDDFT calculations of the UKS triplet state. The results qualitatively differ from the singlet ones by exhibiting an intense excitation in the region around 500 nm and a weaker excitation in the NIR region. Based on the experimental and computed values for the singlet‐triplet gap of compound **9**, the fraction of the triplet species is however too small to contribute to the UV/vis spectrum at ambient temperature.

## Conclusion

In summary, we have synthesised a homologous series of sulfur‐containing imidazolyl‐substituted heteroacenes that form quinoid structures upon oxidation. These thiophene‐derived quinoids, consisting of one to four annulated thiophene units, were isolated in moderate to good yields as bench‐ and oxygen‐stable solids. The optoelectronic and magnetic properties were investigated by UV/vis, fluorescence and EPR spectroscopy as well as quantum chemical calculations, and were compared to those of the benzo derivative **4**. The seven synthesised quinoid structures **4**, **6**, **7**, **8 a**–**c** and **9** display strong absorption in the NIR region between 678 and 819 nm corresponding to a HOMO‐LUMO transition, as confirmed by TDDFT calculations. Whereas the thiophene and thienothiophene derivatives **6** and **7** are closed‐shell molecules, the dithieno[3,2‐*b*:2′,3′‐*d*]thiophene derivatives **8 a**–**c** and the thieno[2′,3′:4,5]thieno[3,2‐*b*]thieno[2,3‐*d*]thiophene derivative **9** turn out to be EPR‐active open‐shell species at room temperature. The singlet‐triplet gap for **9** was determined by VT‐EPR spectroscopy to be 14.1 kJ mol^−1^ in favour of the singlet state. The stability of the sulfur‐containing quinoids arises from their higher tendency to form cross‐conjugated structures compared to all−carbon congeners, resulting in more extensive spin delocalisation and lower LUMO occupation numbers. This knowledge for the concise synthesis of singlet diradical systems like **8** and **9** enables for the design of novel organic open‐shell systems with exceptional thermal and oxygen stability.

## Experimental Section

Detailed experimental procedures, analytical data (NMR spectra, mass spectrometry data, UV/vis, fluorescence, EPR) and computational procedures including atomic coordinates are given in the Supporting Information.

Deposition Numbers 2121377 (for **20 a**), 2127274 (for **4**), 2127276 (for **12**), 2127275 (for **21 b**) contain the supplementary crystallographic data for this paper. These data are provided free of charge by the joint Cambridge Crystallographic Data Centre and Fachinformationszentrum Karlsruhe Access Structures service.

## Conflict of interest

The authors declare no conflict of interest.

1

## Supporting information

As a service to our authors and readers, this journal provides supporting information supplied by the authors. Such materials are peer reviewed and may be re‐organized for online delivery, but are not copy‐edited or typeset. Technical support issues arising from supporting information (other than missing files) should be addressed to the authors.

Supporting Information

Supporting Information

## Data Availability

The data that support the findings of this study are available in the supplementary material of this article.
