# Supplementary material for: Impact of Heterocycle Annulation on NIR Absorbance in Quinoid Thioacene Derivatives
Source: Chemistry. 2022 Mar 29;28(23):e202200478. doi: 10.1002/chem.202200478 (PMC9314731; doi:10.1002/chem.202200478)
Supplement: Supplementary file 1 — Supporting Information [file CHEM-28-0-s002.pdf]

Chemistry–A European Journal

Supporting Information

Impact of Heterocycle Annulation on NIR Absorbance in Quinoid Thioacene Derivatives

Peng Hou, Sebastian Peschtrich, Nils Huber, Wolfram Feuerstein, Angela Bihlmeier, Ivo Krummenacher, Roland Schoch, Wim Klopper, Frank Breher, and Jan Paradies*

CONTENTS

1. GENERAL INFORMATION 5

1.1. Chemical materials and synthesis techniques 5

1.2. Characterization methods 6

2. EXPERIMENTAL SECTION 8

2.1. Synthesis of Benzol derivatives 8

2.1.1. Synthesis of 2-(4-(4,5-bis(4-methoxyphenyl)-4,5-dihydro-1H-imidazol-2-yl)phenyl)-4,5-bis(4-methoxyphenyl)-1H-imidazole (12) 8

2.1.2. Synthesis of 3,6-bis(4,5-bis(4-methoxyphenyl)-2H-imidazol-2-ylidene)cyclohexa-1,4-diene (4) 8

2.2. Synthesis of Thiophene derivatives 10

2.2.1. Synthesis of thiophene-2,5-dicarbaldehyde (13) 10

2.2.2. Synthesis of 2,5-bis(4,5-bis(4-methoxyphenyl)-1H-imidazol-2-yl)thiophene (14) 10

2.2.3. Synthesis of 2,5-bis(4,5-bis(4-methoxyphenyl)-2H-imidazol-2-ylidene)-2,5-dihydrothiophene (6) 11

2.3. Synthesis of thieno[3,2-*b*]thiophene derivatives 12

2.3.1. Synthesis of 2,5-bis(4,5-bis(4-methoxyphenyl)-1H-imidazol-2-yl)thieno[3,2-b]thiophene (16) 12

2.3.2. Synthesis of 2,5-bis(4,5-bis(4-methoxyphenyl)-2H-imidazol-2-ylidene)-2,5-dihydrothieno[3,2-b]thiophene (7) 12

2.4. Synthesis of *p*-substituted diphenyl imidazole 14

2.4.1. Synthesis of 1,2-bis(4-fluorophenyl)-2-hydroxyethan-1-one (S1) 14

2.4.2. General synthesis procedure of 1*H*-imidazole (S2a-b) 14

2.4.2.1. 4,5-bis(4-methoxyphenyl)-1H-imidazole (S2a)[SI4] 15

2.4.2.2. 4,5-bis(4-fluorophenyl)-1H-imidazole (S2b) 15

2.4.3. General synthesis procedure of SEM protected imidazole (S3a-c) 16

2.4.3.1. 4,5-bis(4-methoxyphenyl)-1-((2-(trimethylsilyl)ethoxy)methyl)-1H-imidazole (S3a).......... 16

2.4.3.2. 4,5-diphenyl-1-((2-(trimethylsilyl)ethoxy)methyl)-1H-imidazole (S3b).............................. 17

2.4.3.3. 4,5-bis(4-fluorophenyl)-1-((2-(trimethylsilyl)ethoxy)methyl)-1H-imidazole (S3c)............... 17

2.4.4. General synthesis procedure of iodation of protected imidazole (20a-c) 18

2.4.4.1. 2-iodo-4,5-bis(4-methoxyphenyl)-1-((2-(trimethylsilyl)ethoxy)methyl)-1H-imidazole (20a) 18

2.4.4.2. 2-iodo-4,5-diphenyl-1-((2-(trimethylsilyl)ethoxy)methyl)-1H-imidazole (20b) 19

2.4.4.3. 4,5-bis(4-fluorophenyl)-2-iodo-1-((2-(trimethylsilyl)ethoxy)methyl)-1H-imidazole (20c).... 20

2.5. Synthesis of dithienothiophene derivatives 21

2.5.1. Synthesis of ((3,4-dibromothiophene-2,5-diyl)bis(ethyne-2,1-diyl))bis(trimethylsilane) (19) 21

2.5.2. General synthesis procedure of the internal alkynes (21a-c)... 21

2.5.2.1. 2,2'-((3,4-dibromothiophene-2,5-diyl)bis(ethyne-2,1-diyl))bis(4,5-bis(4-methoxyphenyl)-1-((2-(trimethylsilyl)ethoxy)methyl)-1H-imidazole) (21a) 22

2.5.2.2. 2,2'-((3,4-dibromothiophene-2,5-diyl)bis(ethyne-2,1-diyl))bis(4,5-diphenyl-1-((2-(trimethylsilyl)ethoxy)methyl)-1H-imidazole) (21b) 22

2.5.2.3. 2,2'-((3,4-dibromothiophene-2,5-diyl)bis(ethyne-2,1-diyl))bis(4,5-bis(4-fluorophenyl)-1-((2-(trimethylsilyl)ethoxy)methyl)-1H-imidazole) (21c)... 23

2.5.3. General synthesis procedure of the dithieno[3,2-*b*:2',3'-*d*]thiophene (22a-c) [SI6a-b] 24

2.5.3.1. 2-(6-(4,5-bis(4-methoxyphenyl)-1-((2-(trimethylsilyl)ethoxy)methyl)-2,3-dihydro-1H-imidazol-2-yl)dithieno[3,2-b:2',3'-d]thiophen-2-yl)-4,5-bis(4-methoxyphenyl)-1-((2-(trimethylsilyl)ethoxy)methyl)-1H-imidazole (22a)... 24

2.5.3.2. 2-(6-(4,5-diphenyl-1-((2-(trimethylsilyl)ethoxy)methyl)-2,3-dihydro-1H-imidazol-2-yl)dithieno[3,2-b:2',3'-d]thiophen-2-yl)-4,5-diphenyl-1-((2-(trimethylsilyl)ethoxy)methyl)-1H-imidazole (22b) 25

2.5.3.3. 2-(6-(4,5-bis(4-fluorophenyl)-1-((2-(trimethylsilyl)ethoxy)methyl)-2,3-dihydro-1H-imidazol-2-yl)dithieno[3,2-b:2',3'-d]thiophen-2-yl)-4,5-bis(4-fluorophenyl)-1-((2-(trimethylsilyl)ethoxy)methyl)-1H-imidazole (22c) 26

2.5.4. General deprotection procedure of SEM group 26

2.5.4.1. 2,6-bis(4,5-bis(4-methoxyphenyl)-1H-imidazol-2-yl)dithieno[3,2-b:2',3'-d]thiophene (S4a) 27

2.5.4.2. 2,6-bis(4,5-diphenyl-1H-imidazol-2-yl)dithieno[3,2-b:2',3'-d]thiophene (S4b) 27

2.5.4.3. 2,6-bis(4,5-bis(4-fluorophenyl)-1H-imidazol-2-yl)dithieno[3,2-b:2',3'-d]thiophene (S4c)... 27

2.5.5. General synthesis procedure of the oxidized quinoids (8a-c)[SI7] 28

2.5.5.1. 2,6-bis(4,5-bis(4-methoxyphenyl)-2H-imidazol-2-ylidene)-2,6-dihydrodithieno[3,2-b:2',3'-d]thiophene (8a) 28

2.5.5.2. 2,6-bis(4,5-diphenyl-2H-imidazol-2-ylidene)-2,6-dihydrodithieno[3,2-b:2',3'-d]thiophene (8b) 29

2.5.5.3. 2,6-bis(4,5-bis(4-fluorophenyl)-2H-imidazol-2-ylidene)-2,6-dihydrodithieno[3,2-b:2',3'-d]thiophene (8c) 29

2.6. thieno[2',3':4,5]thieno[3,2-*b*]thieno[2,3-*d*]thiophene derivatives 30

2.6.1. perbromothieno[3,2-b]thiophene (25)[SI8] 30

2.6.2. ((3,6-dibromothieno[3,2-b]thiophene-2,5-diyl)bis(ethyne-2,1-diyl))bis(trimethylsilane) (24) 30

2.6.3. 2,2'-((3,6-dibromothieno[3,2-b]thiophene-2,5-diyl)bis(ethyne-2,1-diyl))bis(4,5-bis(4-methoxyphenyl)-1-((2-(trimethylsilyl)ethoxy)methyl)-1H-imidazole) (23) 31

2.6.4. 2,6-bis(4,5-bis(4-methoxyphenyl)-1-((2-(trimethylsilyl)ethoxy)methyl)-1H-imidazol-2-yl)thieno[2',3':4,5]thieno[3,2-b]thieno[2,3-d]thiophene (26)[SI6a-b][SI10] 31

2.6.5. 2,6-bis(4,5-bis(4-methoxyphenyl)-2H-imidazol-2-ylidene)-2,6-dihydrothieno[2',3':4,5] thieno[3,2-b]thieno[2,3-d]thiophene (9) 32

3. CHARACTERIZATION 33

3.3. NMR spectra 33

3.4. Mass spectra 58

3.5. UV-Vis and fluorescence spectra 75

3.6. Cyclic voltammetry measurement 83

3.7. EPR measurement 91

3.8. Quantum chemical calculations 94

3.9. Crystallographic data 95

4. REFERENCE 107

1. General Information

1.1. Chemical materials and synthesis techniques

All the starting materials, reagents and solvents were commercially available and used as received without further purification unless specially mentioned otherwise. The chemical compounds were purchased from *Sigma Aldrich*, *ABCR*, *Alfa Aesar*, *TCI Carl Roth and Acros*. O_2- and moisture-free solvents were either used as commercially received or dried as follow: DCM – distillation with calcium hydride as desiccant, THF – distillation with anhydrous calcium dichloride as desiccant, toluol – distillation with anhydrous calcium dichloride as desiccant, diethyl ether – distillation with anhydrous calcium dichloride as desiccant, acetone – bubbling with argon for 10 mins with molecular sieves as desiccant or lyophilization with schlenk line technique for 3 circles and molecular sieves as desiccant. Molecular sieves were pre-dried in vacuum at 140 °C for 24 h and stored in oven at 120 °C or argon filled glovebox. Water was always of distilled quality.

All manipulations involving air and/or moisture sensitive compounds were carried out in oven dried (120 °C) glassware (round-bottom-flask, vial). Reactions using air and/or moisture sensitive compounds were performed under dry O_2-free Argon atmosphere with schlenk line techniques or in argon filled glovebox (*Glovebox System)*. Liquids were transferred with plastic syringes with steel cannulas (120 mm, Ø0.8 mm). The following cooling bath mixtures were employed for reactions taking place below room temperature: 0 °C-water/ice, −10 to 0 °C-ice/NaCl, −78 to −10 °C-acetone/liquid nitrogen. Reaction monitoring was done by thin-layer chromatography using silica gel coated aluminm TLC sheets (TCL silica gel 60, F_{254}). The purifications were carried out on silica gel in flash column chromatography under constant excess pressure of 0.8 bar of nitrogen gas. (Si 60). Organic solvents used for the flash column chromatography were distilled under reduced pressure with a rotary evaporator (water bath, temperature at 60 °C).

1.2. Characterization methods

Nuclear magnetic resonance spectroscopy (NMR)

^{1}H-NMR spectra were recorded on a *Bruker Avance* 300 NMR-spectrometer (300 MHz), a *Bruker Avance* 500 NMR-spectrometer (500 MHz) or a *Bruker Ascent* 700 NMR-spectrometer (700 MHz) in deuterated solvents. Chemical shifts are expressed in ppm (parts per million) referred to the residual proton and carbon signal of $CDCl_3$ (7.26 ppm for ^{1}H-NMR, 77.16 ppm for ^{13}C-NMR), DMSO-d_6 (2.50 ppm for ^{1}H-NMR, 39.52 ppm for ^{13}C-NMR), THF-d_8 (1.72 ppm, 3.58 ppm for ^{1}H-NMR, 67.21 ppm, 25.31 ppm for ^{13}C-NMR), toluol-d_6 (2.08 ppm, 6.97 ppm, 7.01 ppm, 7.09 ppm for ^{1}H-NMR, 137.48 ppm, 128.87 ppm, 127.96 ppm, 125.12 ppm and 20.43 ppm for ^{13}C-NMR). The description of ^{1}H-NMR signals multiplicity involved are: s = singlet, d = doublet, t = triplet, q = quartet, b = broad, m = multiplet etc. The ^{1}H coupling constants are given in absolute values in Hertz (Hz). The data are reported as followed: chemical shift, multiplicity, integration, absolute values of coupling constant in Hz. ^{13}C-NMR spectra were recorded on a *Bruker Avance* 500 NMR-spectrometer (500 MHz) or a *Bruker Ascent* 700 NMR-spectrometer (700 MHz) in deuterated solvents. All measurements performed by 500 MHz are at 303 K and all measurements performed by 700 MHz are at 298 K.

Mass spectrometry (MS)

The mass spectrometry was recorded on a *Finnigan* MAT 95 mass-spectrometer (ionization EI), a *Waters Synapt* 2G mass-spectrometer (ionization ESI). Molecular fragments are observed as mass-to-charge ratio (m/z) and the abbreviation [M] refers to neutral molecule while [M^+] refers to the molecule-ion.

Infrared spectroscopy (IR)

IR spectra were recorded from solids (ATR) on a *JASCO* FTIR 460 infrared spectrometer. The deposit of the absorption bands are given in wave numbers ($\tilde{\nu}$) in cm^{-1}. The indications of the absorption are categorized as follow: vs (very strong, < 20% transmission), s (strong, 20%-40% transmission), m (medium, 40%-60% transmission), w (weak, 60%-80% transmission) and vw (very weak, > 80% transmission).

Ultraviolet-visible light spectroscopy (UV-Vis)

UV-vis spectra were recorded on an *Agilent Technologies* Cary 50 UV-VIS-spectrometer. The measurements were performed in degassed DCM or THF solutions of the respective compounds.

Fluorescence spectroscopy

Luminescence spectra were recorded on a *JASCO* FP-8300 fluorescence-spectrometer. The measurements were performed in degassed DCM or THF solutions of the respective compounds.

Cyclic voltammetry (CV)

The CV spectra were recorded on a *Metrohm Autolab* PGSTAT 101 potentiostat. A platinum or a glassy carbon electrode was used as working electrode and a platinum electrode was used as counter electrode while a silver electrode was used as pseudo reference electrode. Tetrabutylammonium hexafluorophosphate (0.1 M) was added as conducting salt and ferrocene was added during the measurement as an internal standard to refer to the ferrocene/ferrocene$^+$ pair. The measurements were conducted in dry and degassed DCM under nitrogen atmosphere at ambient temperature.

2. Experimental Section

2.1. Synthesis of Benzol derivatives

2.1.1. Synthesis of 2-(4-(4,5-bis(4-methoxyphenyl)-4,5-dihydro-1H-imidazol-2-yl)phenyl)-4,5-bis(4-methoxyphenyl)-1H-imidazole (12)

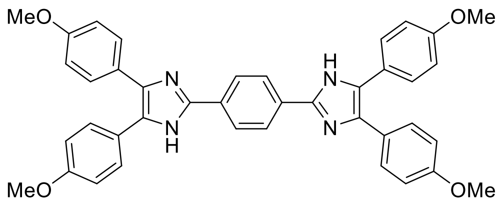

Terephthalaldehyde **10** (536.4 mg, 4.00 mmol, 1.0 equiv.), 4,4′-dimethoxy-benzil **11** (2.3 g, 8.4 mmol, 2.1 equiv.) and ammonium acetate (5.00 g, 64.0 mmol, 16.0 equiv.) were added to a flask followed by injecting 50 mL acetic acid[SI11]. The mixture was heated at 110 ºC for 5 h. Then the reaction was cooled and neutralized with ammonia solution until the pH was about 7. The yellow precipitate was filtered and washed with acetone 2 times then with DCM 2 times. The left yellow solid was dried. The product is a yellow solid with 83% yield (2.1 g).

^{1}H NMR (300 MHz, DMSO): δ [ppm] = 12.54 (s, 2H, H_{NH}), 8.14(s, 4H, H_{Ph}), 7.49 (d, $^3J_{HH}$ = 8.82 Hz, 4H, H_{Ph}), 7.44 (d, $^3J_{HH}$ = 8.82 Hz, 4H, H_{Ph}), 7.02 (d, $^3J_{HH}$ = 8.82 Hz, 4H, H_{Ph}), 6.89 (d, $^3J_{HH}$ = 8.93 Hz, 4H, H_{Ph}), 3.81 (s, 6H, H_{OCH3}), 3.76 (s, 6H, H_{OCH3}).

^{13}C NMR (125 MHz, DMSO): δ [ppm] = 131.7 (2C), 129.4 (4C), 127.9 (4C), 124.9 (4C), 114.5 (2C), 113.8 (4C), 113.4 (4C), 54.9 (4C), 54.7 (4C).

MS (ESI) *m/z*: calc. $[C_{40}H_{35}N_4O_4]^+$ = 635.2653, found: 635.2668.

2.1.2. Synthesis of 3,6-bis(4,5-bis(4-methoxyphenyl)-2H-imidazol-2-ylidene)cyclohexa-1,4-diene (4)

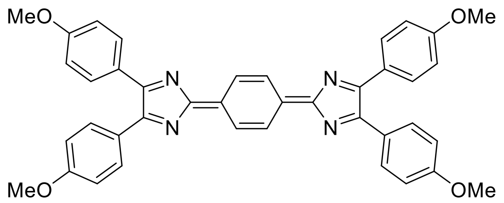

The oxidation was performed in an aqueous solution. 200 mg of **12** was added to 15 mL dioxane and stirred until the solid was fully suspended in the solvent then cooled with ice bath. 5 mL 4% NaOH solution was than added to the suspension. Then the mixture was flushed with nitrogen gas and 30 mL 15% $K_3[Fe(CN)_6]$ solution was added to the mixture[SI11]. A dark green precipitate was formed instantly.

The mixture was stirred for 3 hours at ambient temperature and the precipitation was precipitated by centrifugal machine and washed with water 3 times. The product is greenish solid with 74% yield(148 mg).

^{1}H NMR (700 MHz, $CDCl_3$): δ [ppm] = 8.54 (s, 4H, H_{Ph}), 7.81 (d, $^{3}J_{HH}$ = 8.78 Hz, 8H, H_{Ph}), 6.97 (d, $^{3}J_{HH}$ = 8.79 Hz, 8H, H_{Ph}), 3.89 (s, 12H, H_{OMe}).

^{13}C NMR (176 MHz, $CDCl_3$): δ [ppm] = 166.54 (4C), 166.34 (2C), 162.21 (4C), 135.82 (2C), 132.04 (8C), 131.83 (4C), 126.67 (4C), 114.34 (8C), 55.69 (4C).

MS (ESI) *m/z*: calc. $[C_{38}H_{33}N_4O_4S]^+$ = 633.2497, found: 633.2504.

2.2. Synthesis of Thiophene derivatives

2.2.1. Synthesis of thiophene-2,5-dicarbaldehyde (13)

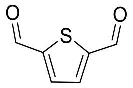

2,5-dibromothiophene (1.93 g, 8.00 mmol, 1.00 equiv.) was added to an oven-dried Schlenk-flask. The flask was flushed 10 mins with argon. Dry THF (40 mL) was injected into the flask via syringe. The mixture was stirred and the flask was cooled to -78 °C in acetone bath. *n*-BuLi (2.5 M in hexane, 7.00 mL, 2.10 equiv.) was added to the flask over half hour and the mixture was stirred for another half hour at -78 °C. DMF (1.75 g, 24.0 mmol, 3.00 equiv.) was slowly added to the mixture via syringe. Then the reaction was stirred overnight at room temperature. The mixture then was poured into HCl solution (0.5 M) and extracted with DCM 3 times [SI1]. The combined organic layer was collected and dried with $MgSO_4$. After removal of the solvent, the product was purified by column chromatography (SiO_2, pure DCM). The product is a colorless liquid with 30% yield (330 mg).

^{1}H NMR (500 MHz, $CDCl_3$): δ [ppm] = 10.01 (s, 2H, H_{CHO}), 7.83 (s, 2H, $H_{thiophene}$).

^{13}C NMR (125 MHz, $CDCl_3$): δ [ppm] = 183.5 (2C), 149.2 (2C), 135.3 (2C).

MS (EI) *m/z*: calc. [$C_6H_4O_2S$] = 139.9932, found: 139.9953.

2.2.2. Synthesis of 2,5-bis(4,5-bis(4-methoxyphenyl)-1H-imidazol-2-yl)thiophene (14)

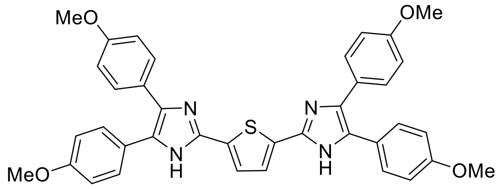

Thiophene-2,5-dicarbaldehyde **13** (210 mg, 1.50 mmol, 1.00 equiv.), 4,4′-dimethoxy-benzil **11** (850 mg, 3.15 mmol, 2.10 equiv.) and ammonium acetate (1.85 g, 24.0 mmol, 16.0 equiv.) were added to a flask followed by injecting10 mL acetic acid[SI1]. The mixture was heated at 110 ºC for 5 h. Then the reaction was cooled and neutralized with ammonia solution until the pH was about 7. The yellow precipitate was filtered and washed with acetone 2 times then with DCM 2 times. The left yellow solid was dried. The product is a yellow solid with 76% yield (729 mg).

^{1}H NMR (300 MHz, DMSO): δ [ppm] = 12.66 (br 2H, H_{NH}), 7.86 (d, $^3J_{HH}$ = 8.79 Hz, 8H, H_{Ph}), 7.60 (s, 2H, $H_{thiophene}$), 7.14 (d, $^3J_{HH}$ = 8.84 Hz, 8H, H_{Ph}), 3.83 (s, 12H, H_{OCH3}).

MS (ESI) *m/z*: calc. [$C_{38}H_{33}N_4O_4S$]$^+$ = 641.2218, found: 641.2267.

2.2.3. Synthesis of 2,5-bis(4,5-bis(4-methoxyphenyl)-2H-imidazol-2-ylidene)-2,5-dihydrothiophene (6)

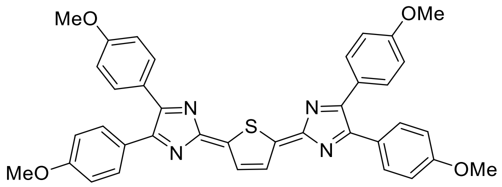

The oxidation was performed in an aqueous solution. 200 mg of **14** was added to 20 mL dioxane and stirred until the solid was fully suspended in the solvent then cooled with ice bath. 5 mL 4% NaOH solution was than added to the suspension. Then the mixture was flushed with nitrogen gas and 30 mL 15% $K_3[Fe(CN)_6]$ solution was added to the mixture. A dark green precipitate was formed instantly. The mixture was stirred for 30 mins at ambient temperature and the precipitation was filtrated and dried. The crude product was then purified by column chromatography (SiO_2, CH:EE:DCM = 3:1:1.5 to 2:1:0.5). The product is dark greenish solid with 88% yield (175 mg).

^{1}H NMR (300 MHz, $CDCl_3$): δ [ppm] = 8.30 (s, 1H, $H_{thiophene}$), 7.4 (dd, $^3J_{HH}$ = 14.68 Hz, 8H, H_{Ph}), 6.94 (dd, $^3J_{HH}$ = 8.84 Hz, 8H, H_{Ph}), 3.88 (s, 12H, H_{OCH3})

^{13}C NMR (125 MHz, $CDCl_3$): δ [ppm] = 162.1 (2C), 161.8 (2C), 138.7 (2C), 131.8 (4C), 131.5 (4C), 126.1 (2C), 114.2 (4C), 114.2 (4C), 55.6 (4C).

MS (ESI) *m/z*: calc. $[C_{38}H_{31}N_4O_4S]^+$ = 639.2061, found: 639.2205.

2.3. Synthesis of thieno[3,2-*b*]thiophene derivatives

2.3.1. Synthesis of 2,5-bis(4,5-bis(4-methoxyphenyl)-1H-imidazol-2-yl)thieno[3,2-b]thiophene (16)

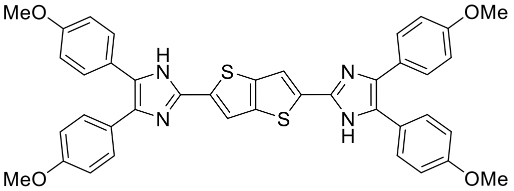

Thieno[3,2-b]thiophene-2,5-dicarboxaldehyde **15** (200 mg, 1.00 mmol, 1.00 equiv.), *p*-anisil **11** (550 mg, 2.00 mmol, 2.00 equiv.) and ammonium acetate (1.57 g, 20.4 mmol, 20.0 equiv.) were solved in 16 mL acetic acid under an argon atmosphere. The mixture was heated to 120 °C for 18 h. After adding ice cold water, it was neutralised with sodium hydroxide and the precipitated solid was filtrated and washed with water. The product was isolated by solving all by-products in boiling DCM. After drying the product **16** was obtained as a yellow solid (304 mg, 43%)[SI2]. ^{13}C-NMR could not be measured because of the poor solubility.

^{1}H NMR (500 MHz, DMSO): δ [ppm] = 12.79 (s, 2H, NH), 7.92 (s, 2H, $H_{thiophene}$), 7.48 - 7.38 (m, 8H, H_{ar}), 7.09 - 6.81 (m, 8H, H_{ar}), 3.78 (s, 12H, CH_3O)

MS (ESI) *m/z*: calc. [$C_{40}H_{33}N_4O_4S_2$] = 697.1943 [M]$^+$, found m/z: 697.1936

2.3.2. Synthesis of 2,5-bis(4,5-bis(4-methoxyphenyl)-2H-imidazol-2-ylidene)-2,5-dihydrothieno[3,2-b]thiophene (7)

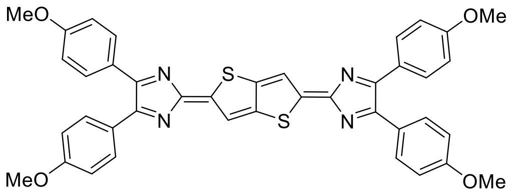

The respective thienoacene **16** (100 mg, 1.00 equiv.) was suspended in dioxane (85 mL/mmol) in an ultrasonic bath for 5 mins. After cooling to 0 °C, an aqueous solution of sodium hydroxide (1 M, 18.0 equiv.) was added and the mixture was stirred for 15 mins. An aqueous solution of potassium hexacyanoferrate(III) (0.2 M, 18.0 equiv.) was prepared and added dropwise. The reaction was slowly warmed to ambient temperature and stirred overnight. The solid was filtrated and washed with water. After drying under reduced pressure, 80.0 mg (81%) of a dark green powder were obtained. Analytical investigation was not completely possible because of poor solubility.

^{1}H NMR (700 MHz, $CDCl_3$): δ [ppm] = 8.40 (s, 2H, $H_{thiophene}$), 7.88 - 7.65 (m, 8H, H_{ar}), 7.04 - 6.84 (m, 8H, H_{ar}), 3.88 (s, 12H, CH_3O)

MS (ESI) *m/z*: calc. $C_{40}H_{30}N_4O_4S_2$: 695.1787 $[M]^+$, found m/z: 695.1774

2.4. Synthesis of *p*-substituted diphenyl imidazole

2.4.1. Synthesis of 1,2-bis(4-fluorophenyl)-2-hydroxyethan-1-one (S1)

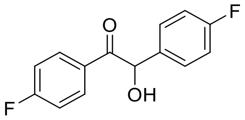

4-fluorobenzaldehyde (1.24 g, 10.0 mmol, 1.00 equiv.), thiazolium chloride (216 mg, 0.80mmol, 8%), Et_3N (0.7 mL) and 10 mL EtOH were added to a vial. The vial was then sealed, and the mixture was degassed for 5 mins. The mixture was stirred at 80 °C overnight. After the reaction mixture was cooled to the room temperature, the mixture was poured into saturated NaCl solution and extracted with DCM 3 times. The combined organic layer was collected and dried with $MgSO_4$ [SI3]. After removal the solvent, the product was purified by column chromatography (SiO_2, CH:EE = 5:1). The product is a white solid with 85% yield (1.06 g).

^{1}H NMR (300 MHz, $CDCl_3$): δ [ppm] = 7.93 (dd, $^3J_{HH}$ = 8.88 Hz, $^3J_{HF}$ = 5.28 Hz, 2H, H_{Ph}), 7.30 (dd, $^3J_{HH}$ = 8.69 Hz, $^3J_{HF}$ = 5.24 Hz, 2H, H_{Ph}), 7.05 (m, 4H, H_{Ph}), 5.59 (s, 1H, H_{HOCHCO}), 4.51 (S, 1H, H_{OH}).

^{13}C NMR (125 MHz, $CDCl_3$): δ [ppm] = 197.3 (1C), 165.2 - 167.25 (d, J_{CF}= 257.08 Hz, 1C), 162 - 163.9 (d, J_{CF}= 247.82 Hz, 1C), 135.0 (1C), 132.0 (2C), 129.9 (1C), 129.7 (2C), 116.1 - 116.5 (d and d, 4C), 75.5 (1C).

MS (ESI) *m/z*: calc. $[C_{14}H_{10}F_2O_2Na]^+$ = 271.0547, found: 271.0541.

2.4.2. General synthesis procedure of 1*H*-imidazole (S2a-b)

4,4′-Dimethoxybenzoin (1.00 equiv.) or 1,2-bis(4-fluorophenyl)-2-hydroxyethan-1-one S1 (1.00 euqiv.) and formamide (15.0 equiv.) were added to a flask and heated at 150 ºC for 6h. The reaction was cooled and added into 5% HCl aqueous solution then extracted with DCM. After drying with $MgSO_4$ and removal of the solvent, the residue was added with NH_4OAc (5.60 g, 72.0 mmol, 6.00 equiv.) in EtOH and reflux for another 6 h. After cooling of the mixture, the solvent was removed, and the residue was dissolved in DCM and washed with water. Then the organic layer was dried with $MgSO_4$, and the organic solvent was evaporated. The crude product was purified per column chromatography (SiO_2, CH:EE:EtOH = 3:2:0.8). The product is a white to pale yellow solid with 50-73% yield

2.4.2.1. 4,5-bis(4-methoxyphenyl)-1H-imidazole (S2a)[SI4]

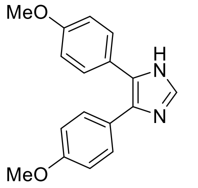

Starting from 4,4′-Dimethoxybenzoin (3.26 g, 12.0 mmol, 1.00 equiv.). The product is white solid with 73% yield (2.46 g)

^{1}H NMR (700 MHz, DMSO): δ [ppm] = 12.26 (s, 1H, H_{NH}), 7.68 (s, 1H, H_{NCHN}), 7.41 (d, $^3J_{HH}$ = 8.26 Hz, 2H, H_{Ph}), 7.32 (d, $^3J_{HH}$ = 8.26 Hz, 2H, H_{Ph}), 6.96 (d, $^3J_{HH}$ = 8.12 Hz, 2H, H_{Ph}), 6.85 (d, $^3J_{HH}$ = 8.15 Hz, 2H, H_{Ph}), 3.77 (s, 3H, H_{OMe}), 3.74 (s, 3H, H_{OMe}).

^{13}C NMR (125 MHz, DMSO): δ [ppm] = 158.5 (1C), 157.8 (1C), 135.2 (1C), 134.8 (1C), 129.1 (2C), 128.2 (1C), 128.1 (2C), 125.2 (1C), 123.8 (1C), 114.1 (2C), 113.6 (2C), 55.1 (1C), 55. 0(1C).

MS (ESI) *m/z*: calc. $[C_{17}H_{17}N_2O_2]^+$ = 281.1290, found: 281.1300, calc. $[C_{17}H_{16}N_2O_2+Na]^+$ = 303.1109, found: 303.1117,

IR: ῦ = 3107 (v), 3005 (vw), 2935 (vw, 2835 (v), 1750 (vw), 1613 (m), 1588 (w), 1524 (m), 1506 (s), 1449 (m), 1414 (w), 1373 (vw), 1292 (m), 1278 (m), 1248 (vs), 1170 (vs), 1131 (m), 1107 (m), 1031 (s), 964 (w), 954 (m), 931 (vs), 799 (s), 747 (m), 659 (m), 634 (m), 624 (m), 609 (w).

2.4.2.2. 4,5-bis(4-fluorophenyl)-1H-imidazole (S2b)

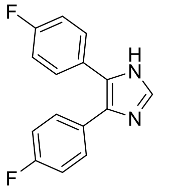

Starting from **S1** (4.98 g, 20.0 mmol, 1.00 equiv.). The product is pale yellow solid with 50% yield (2.54 g)

^{1}H NMR (500 MHz, DMSO): δ [ppm] = 12.51 (s, 1H, H_{NH}), 7.78 (s, 1H, H_{NCHN}), 7.45 (m, 4H, H_{Ph}), 7.19 (b, 4H, H_{Ph})

^{13}C NMR (125 MHz, DMSO): δ [ppm] = 162.3 (1C), 161.7(2C), 160.9 (1C), 160.3 (2C), 135.7 (1C), 135.2 (1C), 131.8 (2C), 130.1 (1C), 128.9 (1C), 127.7 (1C), 125.3 (2C)

MS (ESI) *m/z*: calc. $[C_{15}H_{11}F_2N_2]^+$ = 257.0890, found: 257.0886.

2.4.3. General synthesis procedure of SEM protected imidazole (S3a-c)

S2a-b or 4,5-diphenyl-*1H*-imidazole and NaH (60 wt% suspended in mineral oil, 1.20 equiv.) were added to an oven-dried Schlenk-Flask. The flask was equipped with rubber septum and then evacuated and filled with argon and placed in an ice bath. Dry THF was added slowly into the flask via syringe. The ice bath was removed and the reaction was stirred at room temperature for 30 min. SEMCl (1.10 equiv.) was injected in the reaction mixture over 10 min. The reaction was stirred overnight at ambient temperature. The reaction mixture was then poured into water, the aqueous layer was extracted with DCM. The combined organic layer was dried with $MgSO_4$, and the organic solvent was removed under reduce pressure. The crude product was purified per column chromatography. The product is white solid with 74-93% yield.

2.4.3.1. 4,5-bis(4-methoxyphenyl)-1-((2-(trimethylsilyl)ethoxy)methyl)-1H-imidazole (S3a)

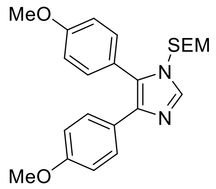

Starting from **S2a** (1.96 g, 7.00 mmol, 1.00 equiv.). The product was purified with column chromatography (SiO_2, CH:EE = 1:1) to afford a white solid with 93% yield (2.67 g).

^{1}H NMR (500 MHz, $CDCl_3$): δ [ppm] = 7.69 (s, 1H, H_{NCHN}), 7.45 (d, $^{3}J_{HH}$ = 8.82 Hz, 2H, H_{Ph}), 7.32 (d, $^{3}J_{HH}$ = 8.82 Hz, 2H, H_{Ph}), 6.96 (d, $^{3}J_{HH}$ = 8.77 Hz, 2H, H_{Ph}), 6.77 (d, $^{3}J_{HH}$ = 8.86 Hz, 2H, H_{Ph}), 5.08 (s, 2H, H_{NCH2O}), 3.86 (s, 3H, H_{OMe}), 3.77 (s, 3H, H_{OMe}), 3.46 (t, $^{3}J_{HH}$ = 8.25 Hz, 2H, $H_{OCH2CH2TMS}$), 0.88 (t, $^{3}J_{HH}$ = 8.30 Hz, 2H, $H_{OCH2CH2TMS}$), −0.02 (s, 9H, H_{TMS}).

^{13}C NMR (125 MHz, $CDCl_3$): δ [ppm] = 159.9 (1C), 158.5 (1C), 138.5 (1C), 137.8 (1C), 132.5 (2C), 128.1 (2C), 127.5 (1C), 127.5 (1C), 122.5 (1C), 114.5 (2C), 113.7 (2C), 73.9 (1C), 66.3 (1C), 55.4 (1C), 55.3 (1C), 17.9 (1C), −1.3 (3C).

MS (ESI) *m/z*: calc. $[C_{23}H_{31}N_2O_3Si]^+$ = 411.2104, found: 411.2128, calc. $[C_{23}H_{30}N_2O_3Si+Na]^+$ = 433.1923, found: 433.1924

IR: ṽ = 5952 (w), 2837 (vw), 1709 (w), 1669 (w), 1606 (m), 1577 (w), 1519 (m), 1497 (m), 1463 (m), 1442 (w), 1442 (w), 1293 (m), 1246 (vs), 1173 (s), 1088 (s), 1030 (s), 946 (w), 920 (w), 831 (vs), 769 (s), 694 (s), 657 (w), 643 (w), 616 (vw).

2.4.3.2. 4,5-diphenyl-1-((2-(trimethylsilyl)ethoxy)methyl)-1H-imidazole (S3b)

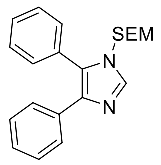

Starting from 4,5-diphenyl-*1H*-imidazole (1.96 g, 7.00 mmol, 1.00 equiv.). The product was purified with column chromatography (SiO_2, CH:EE = 1.5:1) to afford a white solid with 93% yield (2.28 g).

^{1}H NMR (500 MHz, $CDCl_3$): δ [ppm] = 7.79 (s, 1H, H_{NCHN}), 7.19 – 7.56 (m, 10H, H_{Phenyl}), 5.16 (s, 2H, H_{NCH2O}), 3.50 (t, $^3J_{HH}$ = 8.19 Hz, 2H, $H_{OCH2CH2TMS}$), 0.92 (t, $^3J_{HH}$ = 8.26 Hz, 2H, $H_{OCH2CH2TMS}$), 0.02 (s, 9H, H_{TMS}).

^{13}C NMR (125 MHz, $CDCl_3$): δ [ppm] = 138.8 (1C), 137.5 (1C), 134.5 (1C), 131.2 (2C), 130.3 (1C), 129.0 (2C), 128.8 (1C), 128.7 (1C), 128.3 (2C), 127.0 (2C), 126.7 (1C), 74.0 (1C), 66.4 (1C), 17.9 (1C), −1.3 (3C).

MS (ESI) *m/z*: calc. $[C_{21}H_{27}N_2OSi]^+$ = 351,1893, found: 351.1895.

IR: ṽ = 3084 (vw), 3060 (vw), 2955 (w), 2926 (w), 2899 (w), 1601 (m), 1503 (s), 1476 (m), 1443 (m), 1410 (m), 1383 (w), 1358 (m), 1331 (m), 1250 (s), 1235 (s), 1190 (s), 1178 (m), 1154 (w), 1094 (vs), 1068 (s), 1045 (m), 1030 (m), 951 (m), 931 (s), 919 (m), 855 (s), 830 (vs), 793 (s), 778 (s), 747 (s), 723 (s), 695 (vs), 676 (s), 651 (s), 614 (m).

2.4.3.3. 4,5-bis(4-fluorophenyl)-1-((2-(trimethylsilyl)ethoxy)methyl)-1H-imidazole (S3c)

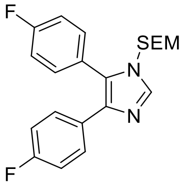

Starting from **S2b** (1.79 g, 7.00 mmol, 1.00 equiv.). The product was purified with column chromatography (SiO_2, CH:EE = 1.5:1) to afford a white solid with 74% yield (2.00 g).

^{1}H NMR (500 MHz, $CDCl_3$): δ [ppm] = 7.73 (s, 1H, H_{NCHN}), 7.44 (dd, $^3J_{HH}$ = 8.87 Hz, $^4J_{HF}$ = 5.45 Hz, 2H, H_{Ph}), 7.38 (dd, $^3J_{HH}$ = 8.80 Hz, $^4J_{HF}$ = 5.32 Hz, 2H, H_{Ph}), 7.13 (t, $^3J_{HH}$ = 8.69 Hz, 2H, H_{Ph}), 6.92 (t, $^3J_{HH}$ = 8.84 Hz, 2H, H_{Ph}), 5.09 (s, 2H, H_{NCH2O}), 3.49 (t, $^3J_{HH}$ = 8.22 Hz, 2H, H $H_{OCH2CH2TMS}$), 0.89 (t, $^3J_{HH}$ = 8.22 Hz, 2H, $H_{OCH2CH2TMS}$), −0.01 (s, 9H, H_{TMS}).

^{13}C NMR (125 MHz, $CDCl_3$): δ [ppm] = 163 - 164.1 (d, $^1J_{CF}$ = 146.41 Hz, 1C), 161.0 - 162.1 (d, $^1J_{CF}$ = 146.4 Hz, 1C), 138.4 (1C), 137.6 (1C), 133.0 - 133.03 (d, $^3J_{CF}$ = 8.20 Hz, 2C), 130.4 (d, $^4J_{CF}$ = 3.38 Hz,

1C), 128.6 - 128.7 (d, $^3J_{CF}$ =87 Hz, 2C), 127.4 (1C), 126.0 (d, $^4J_{CF}$ = 3.41 Hz, 1C), 116.2 - 116.3 (d, $^2J_{CF}$ = 21.51 Hz, 2C), 115.1 - 115.2 (d, $^2J_{CF}$ = 21.38 Hz, 2C), 74.1 (1C), 66.5 (1C), 17.9 (1C), −1. (3C).

MS (ESI) *m/z*: calc. $[C_{21}H_{25}N_2SiOF_2]^+$ = 387.1704, found: 387.1704 .

IR: ũ = 2953 (wv), 2887 (vw), 1774 (vw), 1607 (vw)，1598 (vw)，1516 (vs), 1496 (s), 1404 (w), 1358 (w)，1334 (w), 1334 (w), 1247 (s) 1223 (vs), 1191 (m), 1158 (s), 1087 (s), 1034 (vw), 1016 (vw), 953 (w), 860 (s), 836 (vs), 816 (s), 768 (m), 720 (w), 694 (w), 660 (w), 619 (w).

2.4.4. General synthesis procedure of iodation of protected imidazole (20a-c)

Compound **S3a-c** was added to an oven-dried Schlenk-flask. The flask was evacuated for 10 mins and filled with argon. Dry THF was injected into the flask via syringe. The mixture was stirred until all starting material was dissolved, then the flask was cooled to −78 °C in acetone bath. *n*-BuLi (2.5 M in hexane, 1.10 equiv.) was added to the flask over half hour and the mixture was stirred for another half hour at −78 °C. Iodine (1.50 equiv.) was dissolved in dry THF, and the solution was slowly added to the mixture via syringe. Then the reaction was stirred overnight at room temperature. Then the mixture was poured into saturated $NaHSO_3$ solution and extracted with DCM 3 times. The combined organic layer was collected and dried with $MgSO_4$. After removal the solvent, the product was purified by column chromatography. The product is a white solid with 38-97.5% yield. **18a** was used in synthesis of **8a** and **9** in section *2.4* and *2.5*

2.4.4.1. 2-iodo-4,5-bis(4-methoxyphenyl)-1-((2-(trimethylsilyl)ethoxy)methyl)-1H-imidazole (20a)

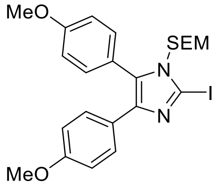

Starting from **S3a** (2.70 g, 6.50 mmol, 1.00 equiv.). The product was purified with column chromatography (SiO_2, CH:EE = 5.5:1) to afford a white solid with 76% yield (2.64 g).

^{1}H NMR (500 MHz, $CDCl_3$): δ [ppm] = 7.39 (d, $^3J_{HH}$ = 8.93 Hz, 2H, H_{Ph}), 7.29 (d, $^3J_{HH}$ = 8.76 Hz, 2H, H_{Ph}), 6.96 (d, $^3J_{HH}$ = 8.74 Hz, 2H, H_{Ph}), 6.75 (d, $^3J_{HH}$ = 8.95 Hz, 2H, H_{Ph}), 5.06 (s, 2H, H_{NCH2O}), 3.86 (s, 3H, H_{OMe}), 3.76 (s, 3H, H_{OMe}), 3.49 (t, $^3J_{HH}$ = 8.66 Hz, 2H, $H_{OCH2CH2TMS}$), 0.88 (t, $^3J_{HH}$ = 8.31 Hz, 2H, $H_{OCH2CH2TMS}$), −0.01 (s, 9H, H_{TMS}).

^{13}C NMR (125 MHz, $CDCl_3$): δ [ppm] = 160.2 (1C), 158.7 (1C), 141.9 (1C), 132.6 (2C), 131.7 (1C), 128.1 (2C), 126.6 (1C), 122.4 (1C), 114.5 (2C), 113.7 (2C), 90.8 (1C), 66.6 (1C), 55.4 (1C), 55.30 (1C), 18.1 (1C), −1.2 (3C).

MS (ESI) *m/z*: calc. $[C_{23}H_{30}N_2O_3SiI]^+$ = 537.1070, found: 537.1051

IR: ῦ = 3001 (vw), 2924 (vw), 2829 (vw), 1613 (m), 1581 (m), 1519 (s), 1495 (s), 1455 (m), 1392 (m), 1320 (m), 1293 (m), 1245 (vs), 1183 (s), 1171 (s), 1121 (w), 1078 (s), 1035 (s), 956 (m), 942 (m), 920 (m), 863 (m), 833 (vs), 767 (s), 728 (m), 707 (m), 667 (w), 647 (w), 634 (m).

2.4.4.2. 2-iodo-4,5-diphenyl-1-((2-(trimethylsilyl)ethoxy)methyl)-1H-imidazole (20b)

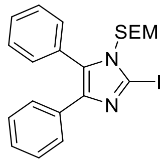

Starting from **S3b** (2.10 g, 6.00 mmol, 1.00 equiv.). The product was purified with column chromatography (SiO_2, CH:EE = 5:1) to afford a white solid with 98% yield (2.80 g).

^{1}H NMR (300 MHz, $CDCl_3$): δ [ppm] = 7.17 - 7.48 (m, 10H, H_{Phenyl}), 5.11 (s, 2H, H_{NCH2O}), 3.49 (t, $^3J_{HH}$ = 8.30 Hz, 2H, $H_{OCH2CH2TMS}$), 0.89 (t, $^3J_{HH}$ = 8.37 Hz, 2H, $H_{OCH2CH2TMS}$), 0.00 (s, 9H, H_{TMS}).

^{13}C NMR (125 MHz, $CDCl_3$): δ [ppm] = 142.4 (1C), 131.9 (1C), 133.1 (1C), 131.6 (2C), 130.5 (1C), 129.5 (1C), 129.3 (2C), 128.5 (2C), 127.3 (1C), 127.2 (2C), 91.8 (1C), 75.7 (1C), 66.9 (1C), 18.3 (1C), −1.0 (3C).

MS (ESI) *m/z*: calc. $[C_{21}H_{26}IN_2OSi]^+$ = 477.0859, found: 477.0874.

IR: ῦ = 3066 (vw), 2951 (w), 2922 (w), 2890 (w), 1602 (w), 1501 (w),1479 (m), 1450 (m), 1404 (m), 1354 (s), 1323 (m), 1249 (s), 1234 (m), 1209 (w), 1178 (w), 1127 (m), 1082 (s), 1071 (vs), 1021 (w), 986 (m), 955 (m), 940 (m), 923 (m), 854 (s), 835 (vs), 802 (m), 764 (vs), 716 (m), 700 (vs), 670 (m), 662 (m), 622 (w), 609 (m).

2.4.4.3. 4,5-bis(4-fluorophenyl)-2-iodo-1-((2-(trimethylsilyl)ethoxy)methyl)-1H-imidazole (20c)

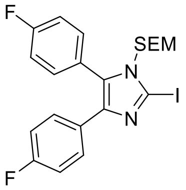

Starting from **S3c** (1.58 g, 4.10 mmol, 1.00 equiv.). The product was purified with column chromatography (SiO_2, CH:EE = 3.5:1) to afford a white solid with 38% yield (0.80 g).

^{1}H NMR (500 MHz, DMSO): δ [ppm] = 7.37 (m, 4H, H_{Ph}), 7.14 (t, $^{3}J_{HH}$ = 8.62 Hz, 2H, H_{Ph}), 6.90 (t, $^{3}J_{HH}$ = 8.73 Hz, 2H, H_{Ph}), 5.05 (s, 2H, H_{NCH2O}), 3.53 (t, $^{3}J_{HH}$ = 8.28 Hz, 2H, H $H_{OCH2CH2TMS}$), 0.90 (t, $^{3}J_{HH}$ = 8.30 Hz, 2H, $H_{OCH2CH2TMS}$), 0.00 (s, 9H, H_{TMS}).

^{13}C NMR (125 MHz, $CDCl_3$): δ [ppm] = 163.3 – 164.6 (d, $^{1}J_{CF}$ = 155.80 Hz, 1C), 161.4 – 162.6 (d, $^{1}J_{CF}$ = 152.29 Hz, 1C), 141.9 (1C), 133.4 (d, $^{3}J_{CF}$ = 8.32 Hz, 2C), 131.6 (1C), 129.9 (d, $^{2}J_{CF}$ = 21.47 Hz, 1C), 128.8 - 128.9 (d, $^{3}J_{CF}$ = 7.86 Hz, 2H), 126.2 (d, $^{4}J_{CF}$ = 3.59 Hz, 1C), 116.5 – 116.7 (d, $^{2}J_{CF}$ = 21.61 Hz, 2C), 115.4 - 115.6 (d, $^{2}J_{CF}$ = 21.47 Hz, 2C), 91.9 (1C), 75.8 (1C), 67.0 (1C), 18.4 (1C), −1.0 (3C).

MS (ESI) *m/z*: calc. $[C_{21}H_{23}F_2IN_2OSi]^+$ = 513.0671, found: 513.0673.

IR: ũ = 2954 (w), 2894 (vw), 1596 (w), 1572 (w), 1515 (s), 1494 (s), 1453 (w), 1391 (m), 1360 (m), 1323 (m), 1247 (m), 1221 (s), 1159 (m), 1124 (m), 1084 (s), 1028 (w), 1014 (w), 957 (w), 913 (w), 858 (s), 834 (vs), 770 (m), 692 (w), 663 (w).

2.5. Synthesis of dithienothiophene derivatives

2.5.1. Synthesis of ((3,4-dibromothiophene-2,5-diyl)bis(ethyne-2,1-diyl))bis(trimethylsilane) (19)

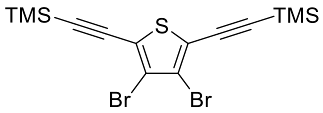

Tetrabromothiophene (2.40 g, 6.00 mmol, 1.00 equiv.) **17** and CuI (114 mg, 0.60 mmol, 10 mol%) were added to a Schlenk-flask and the flask was degased and filled with argon. $Pd(PPh_3)_4$ (173.3 mg, 0.15 mmol, 5 mol%) was added to the flask and degassed THF (20 mL) and DIPA (20 mL) was injected via syringe. At last, TMS-Acetylene (978 mg, 1.95 mL) was injected dropwise into the flask. The reaction was stirred at room temperature overnight. Then the reaction mixture was poured into saturated NH_4Cl solution, extracted with DCM 3 times. The organic layer was collected and dried with $MgSO_4$ then the solvent was removed under reduced presure. The product was purified by column chromatography (SiO_2, pure CH). The product is a brown solid with 65% yield (1.68 g) [SI5].

^{1}H NMR (500 MHz, $CDCl_3$): δ [ppm] = 0.27 (s, 9H, H_{TMS}).

^{13}C NMR (125 MHz, $CDCl_3$): δ [ppm] = 121.4 (1C), 119.6 (1C), 105.8 (1C), 95.2 (1C), −0.2 (1C).

HRMS (EI) *m/z:* calc. [$C_{14}H_{18}Br_2Si_2S$] = 433.9014, found: 433.8999.

IR: ṽ = 2953 (w), 2895 (vw), 2151 (m), 1483 (m), 1309 (m), 1249 (s), 839 (vs), 760 (s), 702 (m), 634 (m).

2.5.2. General synthesis procedure of the internal alkynes (21a-c)

((3,4-dibromothiophene-2,5-diyl)bis(ethyne-2,1-diyl))bis(trimethylsilane) **19** (1.00 euqiv.) and iodid-imidazole derivates **20a-c** (2.00 equiv.) were added to a crimp sealed vial. The vial was degassed and filled with argon. Then in Glovebox, $Pd(PPh_3)_4$ (5 mol%), CuI (10 mol%) and KF (2.20 to 2.50 euqiv.) were added to the vial. Afterwards degassed THF and TEA were injected via syringe. The reaction was stirred at 50 to 80 °C overnight. Then the reaction mixture was poured into saturated NH_4Cl solution, extracted with DCM. The whole organic layer was gathered and dried with $MgSO_4$ and the solvent was removed. The product was purified by column chromatography. The products **21a-c** are dark yellow to dark red-orange solid with 68-92% yield.

2.5.2.1. 2,2'-((3,4-dibromothiophene-2,5-diyl)bis(ethyne-2,1-diyl))bis(4,5-bis(4-methoxyphenyl)-1-((2-(trimethylsilyl)ethoxy)methyl)-1H-imidazole) (21a)

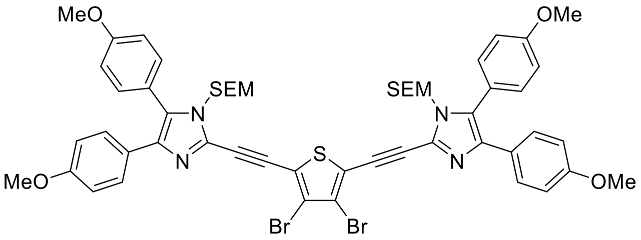

Starting from **19** (86.8 mg, 0.20 mmol, 1.00 equiv.) and **20a** (215 mg, 0.40 mmol, 2.00 equiv.). The product was purified with column chromatography (SiO_2, CH:EE = 3:1) to afford dark yellow solid with 92% yield (182 mg).

^{1}H NMR (500 MHz, $CDCl_3$): δ [ppm] = 7,47 (d, $^3J_{HH}$ = 8.86 Hz, 4H, H_{PhOMe}), 7.38 (d, $^3J_{HH}$ = 8.74 Hz, 4H, H_{PhOMe}), 6.97 (d, $^3J_{HH}$ = 8.78 Hz, 4H, H_{PhOMe}), 6.77 (d, $^3J_{HH}$ = 8.82 Hz, 4H, H_{PhOMe}), 5.32 (s, 4H, H_{NCH2O}), 3.85 (s, 6H, H_{OMe}), 3.76 (s, 6H, H_{OMe}), 3.68 (t, $^3J_{HH}$ = 8.27 Hz, 4H, $H_{OCH2CH2TMS}$), 0.93 (t, $^3J_{HH}$ = 8.25 Hz, 4H, $H_{OCH2CH2TMS}$), −0.03 (s, 18H, H_{TMS}).

^{13}C NMR (125 MHz, $CDCl_3$): δ [ppm] = 160.2 (2C), 158.9 (2C), 140.1 (2C), 132.4 (4C), 130.4 (2C), 130.4 (2C), 128.4 (4C), 126.4 (2C), 121.6 (2C), 121.4 (2C), 119.7 (2C), 114.5 (4C), 113.7 (4C), 89.0 (2C), 84.6 (2C), 73.6 (2C), 67.0 (2C), 55.3 (2C), 55.2 (2C), 18.1 (2C), −1.3 (6C).

MS (ESI) *m/z*: calc. $[C_{54}H_{59}N_4O_6Si_2Br_2S]^+$ = 1107.2035, found: 1107.1840.

IR: ῦ = 3000 (vw), 2948 (w), 2893 (w), 2833 (w), 2203 (w), 1612 (m), 1576 (w), 1520 (s), 1489 (s), 1462 (m), 1440 (m), 1406 (w), 1361 (w), 1322 (m), 1291 (s), 1248 (vs), 1176 (s), 1082 (s), 1033 (s), 965 (m), 939 (w), 916 (w), 834 (vs), 758 (m), 748 (m), 733 (w), 695 (m), 655 (w), 643 (w), 629 (w).

2.5.2.2. 2,2'-((3,4-dibromothiophene-2,5-diyl)bis(ethyne-2,1-diyl))bis(4,5-diphenyl-1-((2-(trimethylsilyl)ethoxy)methyl)-1H-imidazole) (21b)

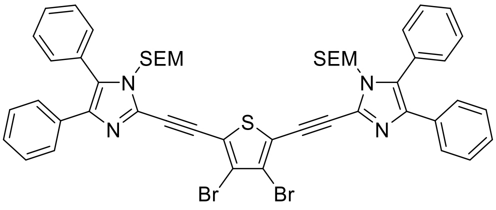

Starting from **19** (163 mg, 0.38 mmol, 1.00 equiv.) and **20b** (358 mg, 0.75 mmol, 2.00 equiv.). The product was purified with column chromatography (SiO_2, CH:EE = 8:1) to afford a dark yellow solid with 82.5% yield (306 mg).

^{1}H NMR (500 MHz, CDCl$_3$): δ [ppm] = 7.22 - 7.56 (m, 20H, H_{Phenyl}), 5.38 (s, 4H, $H_{N\mathit{CH2}O}$), 3.72 (t, $^3J_{HH}$ = 8.30 Hz, 4H, $H_{O\mathit{CH2}CH2TMS}$), 0.96 (t, $^3J_{HH}$ = 8.18 Hz, 4H, $H_{OCH2\mathit{CH2}TMS}$), 0.00 (s, 18H, H_{TMS}).

^{13}C NMR (125 MHz, CDCl$_3$): δ [ppm] = 140.5 (2C), 133.6 (2C), 131.4 (2C), 131.1 (4C), 131.0 (2C), 129.6 (2C), 129.3 (2C), 129.1 (4C), 128.3 (4C), 127.4 (4C), 127.3 (2C), 121.5 (2C), 120.0 (2C), 88.8 (2C), 84.7 (2C), 73.8 (2C), 67.1 (2C), 18.2 (2C), −1.2 (6C).

MS (ESI) *m/z*: calc. $[C_{50}H_{51}Br_2N_4O_2SSi_2]^+$ = 987.1618, found: 987.1635.

IR: ũ = 3055 (vw), 2951 (w), 2897 (w), 2865 (w), 2206 (w), 1602 (w), 1545 (vw), 1526 (vw), 1502 (w), 1474 (w), 1446 (m), 1440 (m), 1402 (w), 1360 (m), 1316 (m), 1258 (w), 1244 (s), 1213(w), 1196 (w), 1152 (vw), 1124 (w), 1102 (s), 1067 (m), 1042 (w), 1028 (w), 997 (vw), 960 (w), 949 (w), 914 (m), 855 (s), 835 (vs), 789 (s), 778 (s), 766 (s), 752 (m), 715 (m), 695 (vs), 664 (m), 650 (m), 616 (w), 569 (vw), 542 (w), 499 (w), 450 (w), 373 (w).

2.5.2.3. 2,2'-((3,4-dibromothiophene-2,5-diyl)bis(ethyne-2,1-diyl))bis(4,5-bis(4-fluorophenyl)-1-((2-(trimethylsilyl)ethoxy)methyl)-1H-imidazole) (21c)

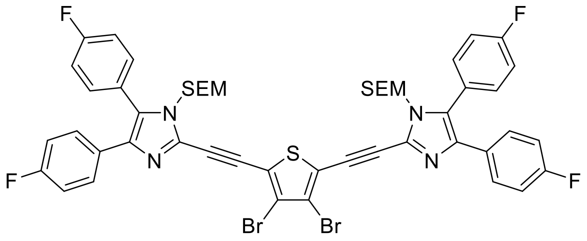

Starting from **19** (86.8 mg, 0.20 mmol, 1.00 equiv.) and **20c** (205 mg, 0.40 mmol, 2.00 equiv.). The product was purified with column chromatography (SiO_2, CH:EE = 7:1) to afford a dark red-orange solid with 67.8% yield (144 mg).

^{1}H NMR (500 MHz, DMSO): δ [ppm] = 7.45 (m, 8H, H_{Ph}), 7.15 (t, $^3J_{HH}$ = 8.66 Hz, 4H, H_{Ph}), 6.92 (t, $^3J_{HH}$ = 8.83 Hz, 4H, H_{Ph}), 5.33 (s, 4H, $H_{N\mathit{CH2}O}$), 3.71 (t, $^3J_{HH}$ = 8.21 Hz, 2H, H $H_{O\mathit{CH2}CH2TMS}$), 0.94 (t, $^3J_{HH}$ = 8.25 Hz, 2H, $H_{OCH2\mathit{CH2}TMS}$), -0.02 (s, 18H, H_{TMS}).

^{13}C NMR (125 MHz, CDCl$_3$): δ [ppm] = 163.2 - 164.3 (d, $^1J_{CF}$ = 136.07 Hz, 2C), 161.3 - 162.3 (d, $^1J_{CF}$ = 132.71 Hz, 2C), 139.9 (2C), 132.9 - 133.0 (d, $^3J_{CF}$ = 8.30 Hz, 4C), 131.0 (2C), 123.0 (2C), 129.5 (d, $^4J_{CF}$ =3.25 Hz, 2C), 128.9 - 129.0 (d, $^3J_{CF}$ = 8.07 Hz, 4H), 125.2 (d, $^4J_{CF}$ = 3.54 Hz, 2C), 121.4 (2C), 120.1 (2C), 116.3 - 116.4 (d, $^2J_{CF}$ = 24.43 Hz, 4C), 115.2 - 115.4 (d, $^2J_{CF}$ = 21.65 Hz, 4C), 88.5 (2C), 84.8 (2C), 73.7 (2C), 67.2 (2C), 18.1 (2C), −1.3 (6C).

MS (ESI) *m/z*: calc. $[C_{50}H_{47}Br_2F_4N_4O_2SSi_2]^+$ = 1057.1261, found: 1057.1283.

IR: ṽ = 3055 (vw), 2949 (w), 2892 (w), 2202 (w), 1606 (m), 1516 (s), 1488 (s), 1443 (m), 1404 (m), 1359 (m), 1318 (m), 1223 (s), 1158 (s), 1080 (s), 1015 (w), 965 (w), 913 (w), 833 (vs), 756 (m),694 (m), 626 (w).

2.5.3. General synthesis procedure of the dithieno[3,2-*b*:2',3'-*d*]thiophene (22a-c) [SI6a-b]

Internal alkynes **21a-c** were separately added to vials, then $Pd(dba)_2$ (10 mol%), dippf (20 mol%), K_3PO_4 (6.00 euqiv.), KSAc (4.00 euqiv.) were added to the vials under O_2 free condition. Degassed toluene and acetone were injected to the mixtures in argon atmosphere. The reactions were stirred at 120 °C overnight. After the mixture was cooled, the mixture was diluted with DCM and filtrated per silica gel. The solvents then were removed from the filtrated solutions. The products were isolated per column chromatography. The products **22a-c** were obtained as dark orange to brown solid with 41-58% yield.

2.5.3.1. 2-(6-(4,5-bis(4-methoxyphenyl)-1-((2-(trimethylsilyl)ethoxy)methyl)-2,3-dihydro-1H-imidazol-2-yl)dithieno[3,2-b:2',3'-d]thiophen-2-yl)-4,5-bis(4-methoxyphenyl)-1-((2-(trimethylsilyl)ethoxy)methyl)-1H-imidazole (22a)

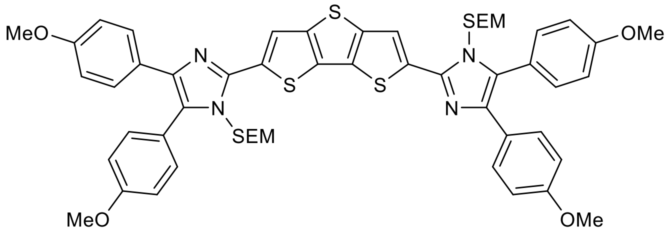

Starting from **21a** (181 mg, 0.16 mmol, 1.00 equiv.). The product was purified with column chromatography (SiO_2, CH:EE:DCM = 2:0.5:1.8) to afford a dark brown with 41% yield (68.2 mg).

^{1}H NMR (500 MHz, $CDCl_3$): δ [ppm] = 7.85 (s, 2H, $H_{thiophen}$), 7.51 (d, $^3J_{HH}$ = 8.78 Hz, 4H, $H_{\textit{Ph}OMe}$), 7.35 (d, $^3J_{HH}$ = 8.46 Hz, 4H, $H_{\textit{Ph}OMe}$), 7.01 (d, $^3J_{HH}$ = 8.34 Hz, 4H, $H_{\textit{Ph}OMe}$), 6.79 (d, $^3J_{HH}$ = 8.85 Hz, 4H, $H_{\textit{Ph}OMe}$), 5.24 (s, 4H, $H_{N\textit{CH2}O}$), 3.89 (s, 6H, $H_{\textit{OMe}}$), 3.78 (s, 6H, $H_{\textit{OMe}}$), 3.41 (t, $^3J_{HH}$ = 8.43 Hz, 4H, $H_{O\textit{CH2}CH2TMS}$), 0.90 (t, $^3J_{HH}$ = 8.43 Hz, 4H, $H_{OCH2\textit{CH2}TMS}$), 0.01 (s, 18H, $H_{\textit{TMS}}$).

^{13}C NMR (125 MHz, $CDCl_3$): δ [ppm] = 160.2 (2C), 158.6 (2C), 142.5 (2C), 142.2 (2C), 138.1 (2C), 134.3 (2C), 132.9 (4C), 132.0 (2C), 129.6 (2C), 128.3 (4C), 127.0 (2C), 122.7 (2C), 120.1 (2C), 114.6 (4C), 113.7 (4C), 72.7 (2C), 66.2 (2C), 55.5 (2C), 55.3 (2C), 18.1 (2C), −1.3 (6C).

MS (ESI) *m/z*: calc. $[C_{54}H_{61}N_4O_6Si_2S_3]^+$ = 1013.3290, found: 1013.3360, calc. $[C_{54}H_{62}N_4O_6Si_2S_3]^{2+}$ = 507.1680, found: 507.1726

IR: ṽ = 2952 (w), 2926 (w), 2899 (w), 2834 (vw), 2361 (w9, 1713 (w), 1667 (w), 1612 (m), 1576 (w), 1518 (s), 1493 (s), 1463 (m), 1440 (w), 1417 (w), 1395 (w), 1364 (w), 1329 (w), 1292 (m), 1247 (vs), 1175 (s), 1077 (s), 1029 (s), 954 (w), 939 (w), 916 (vw), 858 (m), 833 (vs), 801 (s), 765 (m), 746 (m), 699 (m), 620 (w), 601 (w), 541 (w).

2.5.3.2. 2-(6-(4,5-diphenyl-1-((2-(trimethylsilyl)ethoxy)methyl)-2,3-dihydro-1H-imidazol-2-yl)dithieno[3,2-b:2',3'-d]thiophen-2-yl)-4,5-diphenyl-1-((2-(trimethylsilyl)ethoxy)methyl)-1H-imidazole (22b)

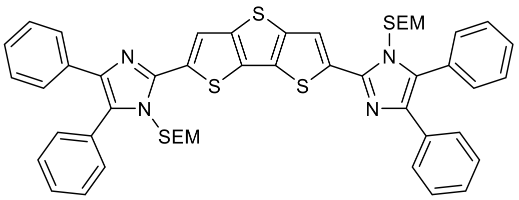

Starting from **21b** (140 mg, 0.14 mmol, 1.00 equiv.). The product was purified with column chromatography (SiO_2, CH:DCM:acetone = 3:1.5:0.5) to afford a dark orange solid with 58% yield (73.5 mg).

^{1}H NMR (500 MHz, $CDCl_3$): δ [ppm] = 7.91 (s, 2H), 7.20 - 7.61 (m, 20H, H_{Phenyl}), 5.30 (s, 4H, $H_{N\mathit{CH2}O}$), 3.43 (t, $^3J_{HH}$ = 8.43 Hz, 4H, $H_{O\mathit{CH2}CH2TMS}$), 0.92 (t, $^3J_{HH}$ = 8.46 Hz, 4H, $H_{OCH2\mathit{CH2}TMS}$), 0.01 (s, 18H, H_{TMS}).

^{13}C NMR (125 MHz, $CDCl_3$): δ [ppm] = 142.9 (2C), 142.5 (2C), 138.4 (2C), 134.2 (2C), 134.1 (2C), 132.1 (2C), 131.5 (4C), 130.7 (2C), 130.6 (2C), 129.2 (4C), 129.0 (2C), 128.2 (4C), 127.2 (4C), 126.8 (2C), 120.3 (2C), 72.8 (2C), 66.2 (2C), 18.1 (2C), −1.3 (6C).

MS (ESI) *m/z*: calc. $[C_{50}H_{53}N_4O_2S_3Si_2]^+$ = 893.2869, found: 893.2881.

IR: ṽ = 3057 (vw), 2949 (w), 2928 (w), 2888 (w), 1737 (w), 1602 (w),1578 (vw), 1552 (vw), 1501 (w), 1477 (m), 1443 (m), 1395 (w), 1362 (m), 1327 (w), 1247 (s), 1189 (w), 1124 (w), 1075 (s), 1028 (m), 951 (w), 935 (vw), 915 (w), 856 (s), 831 (vs), 771 (s), 717 (m), 693 (vs), 667 (m), 627 (vw), 615 (vw), 602 (vw).

2.5.3.3. 2-(6-(4,5-bis(4-fluorophenyl)-1-((2-(trimethylsilyl)ethoxy)methyl)-2,3-dihydro-1H-imidazol-2-yl)dithieno[3,2-b:2',3'-d]thiophen-2-yl)-4,5-bis(4-fluorophenyl)-1-((2-(trimethylsilyl)ethoxy)methyl)-1H-imidazole (22c)

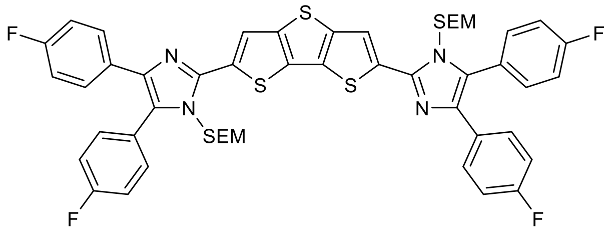

Starting from **21c** (135 mg, 0.13 mmol, 1.00 equiv.). The product was purified with column chromatography (CH:DCM:acetone = 3:1.5:0.5) to afford a dark brown solid with 51% yield (62.8 mg).

^{1}H NMR (500 MHz, CDCl$_3$): δ [ppm] = 7.91 (s, 2H, $H_{Thiophene}$), 7.55 (dd, $^3J_{HH}$ = 8.84 Hz, $^3J_{FH}$ = 5.41 Hz, 4H, H_{Ph}), 7.49 (dd, $^3J_{HH}$ = 8.51 Hz, $^3J_{FH}$ = 5.42 Hz, 4H, H_{Ph}), 7.26 (t, $^3J_{HH}$ = 8.57 Hz, 4H, H_{Ph}), 7.00 (t, $^3J_{HH}$ = 8.79 Hz, 4H, H_{Ph}), 5.28 (s, 4H, H_{NCH2O}), 3.49 (t, $^3J_{HH}$ = 8.36 Hz, 4H, s $H_{OCH2CH2TMS}$), 0.98 (t, $^3J_{HH}$ = 8.42 Hz, 4H, $H_{OCH2CH2TMSs}$), 0.06 (s, 18H, H_{TMS}).

^{13}C NMR (125 MHz, CDCl$_3$): δ [ppm] = 163.07–164.29 (d, $^1J_{CF}$ = 153.30 Hz, 2C), 161.11-162.30 (d, $^1J_{CF}$ = 149.32 Hz, 2C), 142.98 (2C), 142.43 (2C), 137.93 (2C), 133.90 (2C), 133.32 – 133.38 (d, $^3J_{CF}$ = 8.21 Hz, 4C), 132.10 (2C), 130.06 (d, $^4J_{CF}$ =3.21, 2C), 129.29 (2C), 128.83 – 128.90 (d, $^3J_{CF}$ = 7.97 Hz, 4C), 126.3 (d, $^4J_{CF}$ = 3.56 Hz, 2C), 120.41 (2C), 116.39 – 116.56 (d, $^2J_{CF}$ = 21.80 Hz, 4C), 115.15 – 115.32 (d, $^2J_{CF}$ = 21.41 Hz, 4C), 72.92 (2C), 66.29 (2C), 18.15 (2C), -1.30 (6C).

MS (ESI) *m/z*: calc. $[C_{50}H_{49}F_4N_4O_2S_3Si_2]^+$ = 965.2492, found: 965.2474.

2.5.4. General deprotection procedure of SEM group

The deprotection procedure of compounds **22a-c** were performed as follow: **22a-c** were separated suspended in THF together with excessive load of HCl (in dioxane, 4M) and heated at 50-70°C overnight. After the mixture was cooled down, a pale yellow to orange precipitate were formed and filtered. The collected solids were rinsed with THF 2 times and cyclohexane 2 times, then the suspensions were centrifugalized and dried to afford a yellow solid. Due to the very poor solubility of the products, NMR-data cannot be recorded. The deprotected products were used without further purification for oxidation.

2.5.4.1. 2,6-bis(4,5-bis(4-methoxyphenyl)-1H-imidazol-2-yl)dithieno[3,2-b:2',3'-d]thiophene (S4a)

Starting from **22a** (50.0 mg, 1.00 equiv.). The deprotected product was used without further purification. The product is a yellow solid with 85% yield (32.0 mg)

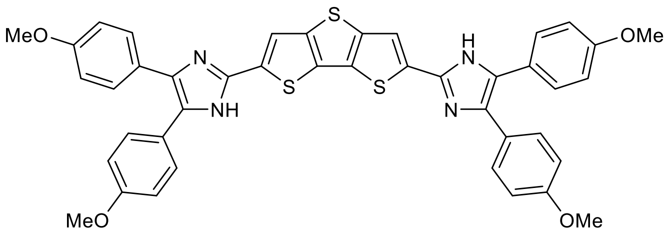

MS (ESI) *m/z*: calc. $[C_{42}H_{33}N_4O_4S_3]^+$ = 753.1659, found: 753.1716.

IR: ṽ = 3366(vw), 2661 (vw), 2534 (vw), 2360 (vw), 1637 (s), 1610 (s),1572 (m), 1527 (m), 1503 (s), 1462 (m), 1441 (m), 1384 (m), 1297 (m), 1250 (vs), 1175 (s), 1111 (w), 1087 (w), 1067 (w), 1023 (s), 969 (vw), 934 (vw), 830 (vs), 801 (m), 704 (w), 623 (vw), 585 (w), 531 (m), 503 (m)s.

2.5.4.2. 2,6-bis(4,5-diphenyl-1H-imidazol-2-yl)dithieno[3,2-b:2',3'-d]thiophene (S4b)

Starting from **22b** (50.0 mg, 1.00 equiv.). The deprotected product was used without further purification. The product is a yellow solid with 84% yield (29.7 mg).

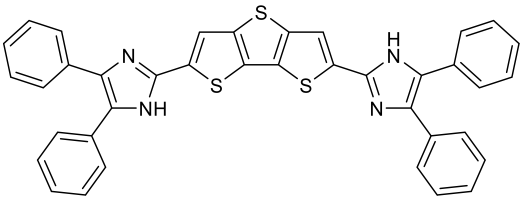

MS (ESI) *m/z*: calc. $[C_{38}H_{25}N_4S_3]^+$ = 633.1236, found: 633.1234.

IR: ṽ = 3320 (w), 3033 v(w), 2530 (w), 1636 (vs), 1599 (s), 1575 m),1555 (m), 1479 (m), 1441 (m), 1381 (s), 1295 (w), 1254 (m), 1179 (m), 1158 (w), 1162 (vw), 1088 (m), 1076 (m), 1024 (m), 967 (vw), 918 (m), 867 (m), 839 (m), 762 (vs), 693 (vs), 597 (s), 505 (s), 484 (m), 429 (m).

2.5.4.3. 2,6-bis(4,5-bis(4-fluorophenyl)-1H-imidazol-2-yl)dithieno[3,2-b:2',3'-d]thiophene (S4c)

Starting from **22c** (40.0 mg, 1.00 equiv.). The deprotected product was used without further purification. The product is a pale yellow solid with 63% yield (18.4 mg).

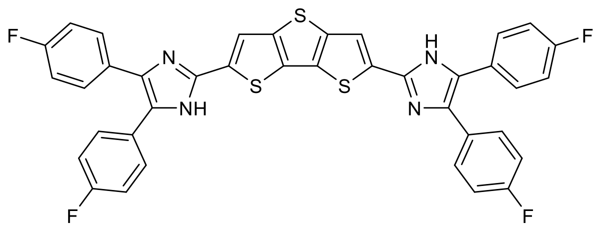

MS (ESI) *m/z*: calc. $[C_{38}H_{21}F_4N_4S_3]^+$ = 705.0859, found: 705.0803.

IR: ũ = 3342 (w), 3050 (vw), 2862 (vw), 2508 (vw), 1637 (s), 1608 (m),1590 (m), 1560 (m), 1523 (m), 1501 (s), 1408 (m), 1383 (m), 1327 (w), 1300 (w), 1230 (s), 1160 (s), 1092 (w), 1013 (w), 933 (vw), 835 (vw), 813 (s), 736 (w), 732 (w), 696 (w), 625 (w), 596 (m), 579 (w), 552 (w), 522 (m), 436 (w).

2.5.5. General synthesis procedure of the oxidized quinoids (8a-c)[SI7]

3.10 - 4.10 mg of the deprotected products **S4a-c** were suspended in 1-1.4 mL dioxane in ultrasonic bath for 5 mins. Several drops of 1M NaOH solution was then added to the suspension till the suspension turned into a clear solution and the solution was stirred at ambient temperature for 30 mins. The mixture was then flushed with nitrogen gas and 3 mL 0.04M $K_3[Fe(CN)_6]$ solution was slowly giving to the mixture. A dark blue precipitation was formed instantly. The mixture was stirred for 2 h at ambient temperature and the precipitation was filtrated, rinsed with distilled water, and dried. The yields for **8a - c** are 74-85%.

2.5.5.1. 2,6-bis(4,5-bis(4-methoxyphenyl)-2H-imidazol-2-ylidene)-2,6-dihydrodithieno[3,2-b:2',3'-d]thiophene (8a)

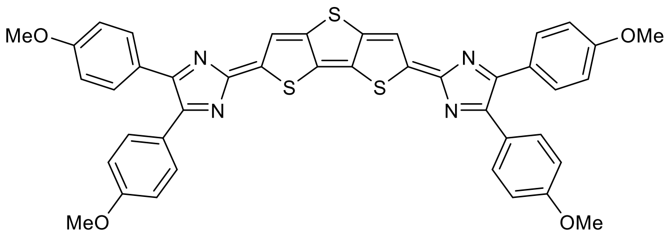

Starting from **S4a** (3.50 mg, 1.00 equiv.). The product is dark blue solid with 83% yield (2.90 mg).

MS (ESI) *m/z*: calc. $[C_{42}H_{31}N_4O_4S_3]^+$ = 751.1516, found: 753.1660, calc. $[C_{42}H_{32}N_4O_4S_3]^{2+}$ = 376.0787, found: 376.0786.

IR: ũ = 3085 (vw), 2958 (m), 2923 (m), 2853 (w), 2551 (vw), 2049 (vw), 1660 (w), 1601 (s), 1573 (m), 1518 (m), 1506 (m), 1461 (m), 1421 (m), 1402 (m), 1317 (m), 1301 (m), 1240 (vs), 1172 (s), 1090 (s),

1016 (vs), 904 (s), 829 (s), 792 (vs), 731 (m), 717 (m), 700 (m), 680 (m), 644 (w), 622 (m), 601 (m), 591 (m), 549 (m), 530 (m), 493 (w), 455 (vw), 289 (w).

2.5.5.2. 2,6-bis(4,5-diphenyl-2H-imidazol-2-ylidene)-2,6-dihydrodithieno[3,2-b:2',3'-d]thiophene (8b)

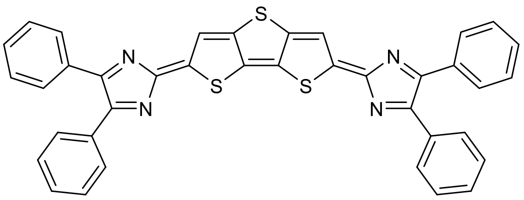

Starting from **S4b** (3.10 mg, 1.00 equiv.). The product is a dark blue solid with 74% yield (2.30 mg).

MS (ESI) *m/z*: calc. $[C_{38}H_{23}N_4S_3]^+$ = 631.1085, found: 631.1086.

IR: ῡ = 3426 (vw), 3050 (w), 2959 (w), 2918 (w), 1657 (vw), 1657 (vw),1599 (w), 1556 (m), 1470 (w), 1447 (w), 1435 (w), 1405 (w), 1324 (m), 1297 (m), 1259 (vs), 1239 (w), 1176 (w), 1156 (w), 1093 (m), 1032 (s), 1014 (s), 961 (m), 902 (s), 825 (s), 801 (s), 764 (s), 734 (m), 718 (m), 690 (s), 670 (s), 601 (m), 546 (w), 481 (m), 461 (w), 408 (m).

2.5.5.3. 2,6-bis(4,5-bis(4-fluorophenyl)-2H-imidazol-2-ylidene)-2,6-dihydrodithieno[3,2-b:2',3'-d]thiophene (8c)

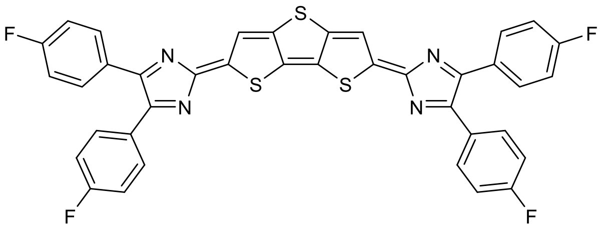

Starting from **S4c** (4.10 mg, 1.00 equiv.). Dark blue solid with 85% yield (3.50 mg).

MS (ESI) *m/z*: calc. $[C_{38}H_{19}F_4N_4S_3]^+$ = 703.0708, found: 703.0714.

IR: ῡ = 2954 (w), 2922 (w), 2848 (w), 2114 (w), 2039 (w), 1662 (vw), 1598 (s), 1565 (w), 1514 (m), 1505 (s), 1473 (m), 1412 (m), 1309 (s), 1294 (m), 1260 (s), 1228 (vs), 1157 (vs), 1085 (m), 1022 (s), 1012 (s), 962 (w), 904 (ss), 834 (vs), 811 (s), 716 (m), 678 (w), 623 (s), 595 (s), 547 (m), 521 (m), 441 (w), 391 (m).

2.6. thieno[2',3':4,5]thieno[3,2-*b*]thieno[2,3-*d*]thiophene derivatives

2.6.1. perbromothieno[3,2-b]thiophene (25)[SI8]

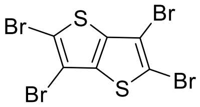

Thienothiophene (2.00 g, 14.3 mmol, 1.00 equiv.) was suspended in 16 mL of chloroform and a solution of bromine (11.4 g, 71.3 mmol, 5.00 equiv.) in 16 mL chloroform was added dropwise at ambient temperature. The mixture was heated to 60 °C for 5 hours and stirred at 40 °C overnight. After TLC control the suspension was dissolved with DCM and treated with saturated Na_2SO_3 solution. The product was filtrated and dried under reduced pressure. The isolated pale yellow solid (5.30 g, 82%) was without further analysis because of the poor solubility and applied directly in the next syntheses. An ^{1}H-NMR showed no significant signals.

2.6.2. ((3,6-dibromothieno[3,2-b]thiophene-2,5-diyl)bis(ethyne-2,1-diyl))bis(trimethylsilane) (24)

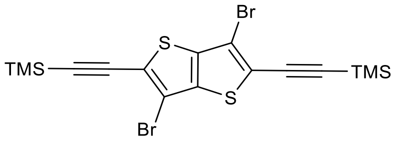

Tetrabromothienothiophene **25** (1.00 g, 2.20 mmol, 1.00 euqiv.), copper(I) iodide (42.0 mg, 0.20 mmol, 10 mol%) and $Pd(PPh_3)_4$ (127 mg, 0.10 mmol, 5 mol%) were solved in 7 mL of triethylamine and 14 mL of THF under an argon atmosphere. TMS-acetylene (431 mg, 4.40 mmol, 2.00 equiv.) was added dropwise and the mixture was stirred overnight at 40 °C. The suspension was diluted with DCM and poured into a saturated solution of NH_4Cl [SI9]. The aqueous layer was extracted with DCM. The combined organic layers were washed with water, dried over $MgSO_4$ and the solvent was removed under reduced pressure. The product was isolated by column chromatography (SiO_2, pure CH) to afford a red solid with 62% yield (661 mg).

^{1}H NMR (700 MHz, DMSO): δ [ppm] = 0.29 (s, 18H, H_{TMS})

^{13}C NMR (176 MHz, $CDCl_3$): δ [ppm] = 137.8, 124.1, 109.4, 107.5, 95.7, -0.1

MS (ESI) *m/z*: calc. [$C_{16}H_{19}Si_2S_2Br_2$] = 488.8833 [M]$^+$, found m/z: 488.8824

2.6.3. 2,2'-((3,6-dibromothieno[3,2-b]thiophene-2,5-diyl)bis(ethyne-2,1-diyl))bis(4,5-bis(4-methoxyphenyl)-1-((2-(trimethylsilyl)ethoxy)methyl)-1H-imidazole) (23)

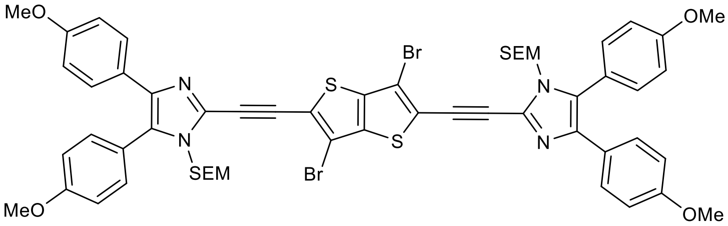

The thienothiophene **24** (85.0 mg, 0.17 mmol, 1.00 equiv.), the imidazole **20a** (186 mg, 0.35 mmol, 2.00 equiv.), potassium fluoride (30.0 mg, 0.52 mmol, 3.00 equiv.), tetrakis(triphenylphosphine)-palladium(0) (16.0 mg, 0.01 mmol, 8 mol%) and copper(I) iodide (5.30 mg, 0.03 mmol, 16 mol%) were solved in 0.6 mL triethylamine and 1.1 mL THF under an argon atmosphere. The mixture was stirred overnight at 40 °C. The solution was diluted with DCM and poured into a saturated solution of NH_4Cl. The aqueous layer was extracted with DCM. The combined organic layers were washed with water, dried over $MgSO_4$ and the solvent was removed under reduced pressure. The product was isolated by column chromatography (SiO_2, DCM:CH:EE = 10:5:1) to afford a red solid with 74% yield (148 mg).

^{1}H NMR (700 MHz, DMSO): δ [ppm] = 7.47 (d, $^3J_{HH}$ = 8.9 Hz, 4H, H_{Ph}), 7.39 (d, $^3J_{HH}$ = 8.7 Hz, 4H, H_{Ph}), 6.98 (d, $^3J_{HH}$ = 8.8 Hz, 4H, H_{Ph}), 6.79 (d, $^3J_{HH}$ = 8.8 Hz, 4H, H_{Ph}), 5.35 (s, 4H, H_{NCH2O}), 3.87 (s, 6H, H_{CH3O}), 3.78 (s, 6H, H_{CH3O}), 3.72 – 3.68 (m, 4H, H_{OCH2}), 0.96 – 0.92 (m, 4H, H_{CH2Si}), -0.04 (s, 18H, H_{SiCH3})

^{13}C NMR (176 MHz, $CDCl_3$): δ [ppm] = 160.3, 158.9, 140.0, 139.0, 132.4, 130.6, 130.4, 128.5, 126.4, 123.3, 121.6, 114.5, 113.8, 109.6, 90.2, 85.4, 73.6, 67.1, 55.4, 55.3, 18.2, -1.25

MS (ESI) *m/z*: calc. [$C_{56}H_{59}N_4O_6Si_2S_2Br_2$] = 1161.1781 [M]$^+$, found m/z: 1161.1721

2.6.4. 2,6-bis(4,5-bis(4-methoxyphenyl)-1-((2-(trimethylsilyl)ethoxy)methyl)-1H-imidazol-2-yl)thieno[2',3':4,5]thieno[3,2-b]thieno[2,3-d]thiophene (26)[SI6a-b][SI10]

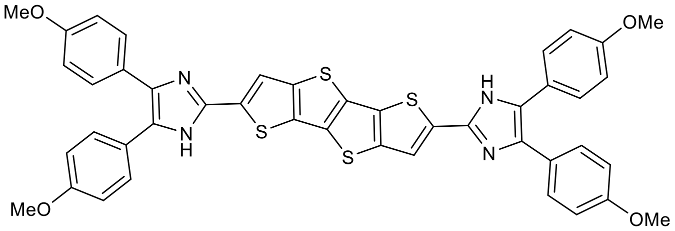

The alkine **23** (170 mg, 0.15 mmol, 1.00 equiv.), K_3PO_4 (87.0 mg, 0.41 mmol, 2.80 equiv.), Pd(dba)$_2$ (8.40 mg, 0.02 mmol, 10 mol%), dippf (7.30 mg, 0.02 mmol, 12 mol%) and KSAc (40.0 mg, 0.35 mmol, 2.40 equiv.) were suspended in 3.4 mL of a 5:1 mixture of toluene/acetone under an argon atmosphere.

The suspension was heated to 130 °C and stirred overnight. It was filtered through a layer of celite which was rinsed with DCM. The solvents were removed under reduced pressure and the raw residue was solved in 6 mL THF and 3 mL concentrated hydrochloric acid. The mixture was heated to 70 °C for 4 h. The resulting precipitant was filtrated and washed with water and DCM. After drying under reduced pressure, 61.0 mg (51%) of a red solid was obtained. ^{13}C-NMR could not be measured because of the poor solubility.

MS (ESI) *m/z*: calc. [$C_{44}H_{33}N_4O_4S_4$] = 809.1385 [M]$^+$, found m/z: 809.1378

2.6.5. 2,6-bis(4,5-bis(4-methoxyphenyl)-2H-imidazol-2-ylidene)-2,6-dihydrothieno[2',3':4,5] thieno[3,2-b]thieno[2,3-d]thiophene (9)

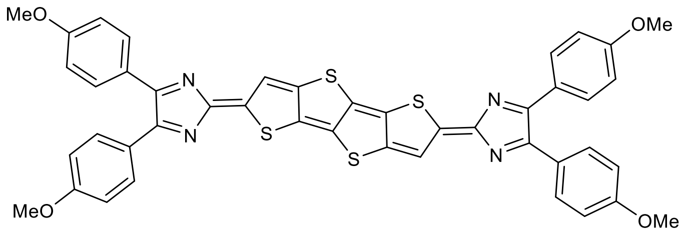

The respective thienoacene **25** (50.0 mg, 1.00 equiv.) was suspended in dioxane (85 mL/mmol) in an ultrasonic bath for 5 mins. After cooling to 0 °C, an aqueous solution of sodium hydroxide (1 M, 18.0 equiv.) was added and the mixture was stirred for 15 mins. An aqueous solution of potassium hexacyanoferrate(III) (0.2 M, 18.0 equiv.) was prepared and added dropwise. The reaction was slowly warmed to ambient temperature and stirred overnight. The solid was filtrated and washed with water. After drying under reduced pressure, 36 mg (72% tetrathienoacen **9**) of a dark green powder were obtained. Analytical investigation was not completely possible because of poor solubility.

MS (ESI) *m/z*: calc. [$C_{44}H_{31}N_4O_4S_4$] = 807.1228 [M]$^+$, found m/z: 807.1215

3. Characterization

3.3. NMR spectra

12 in DMSO, ^{1}H NMR (303 K, 300 MHz)

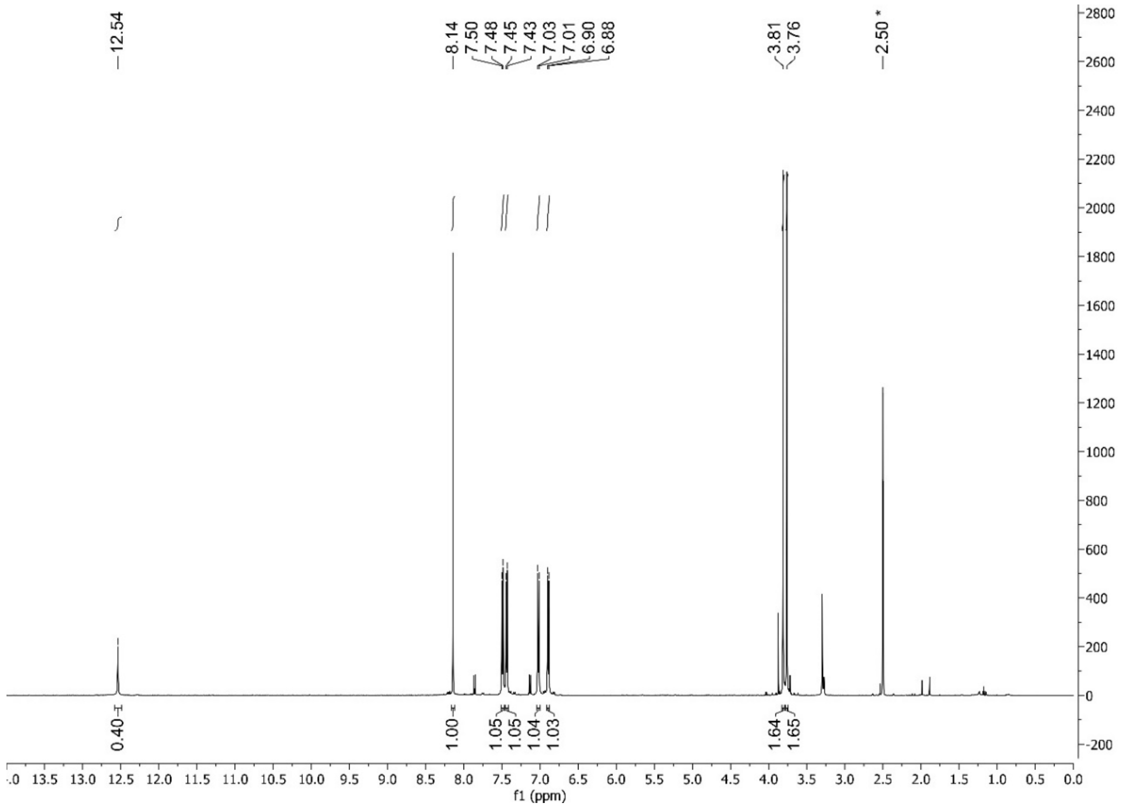

12 in DMSO, ^{13}C NMR (303 K, 125 MHz)

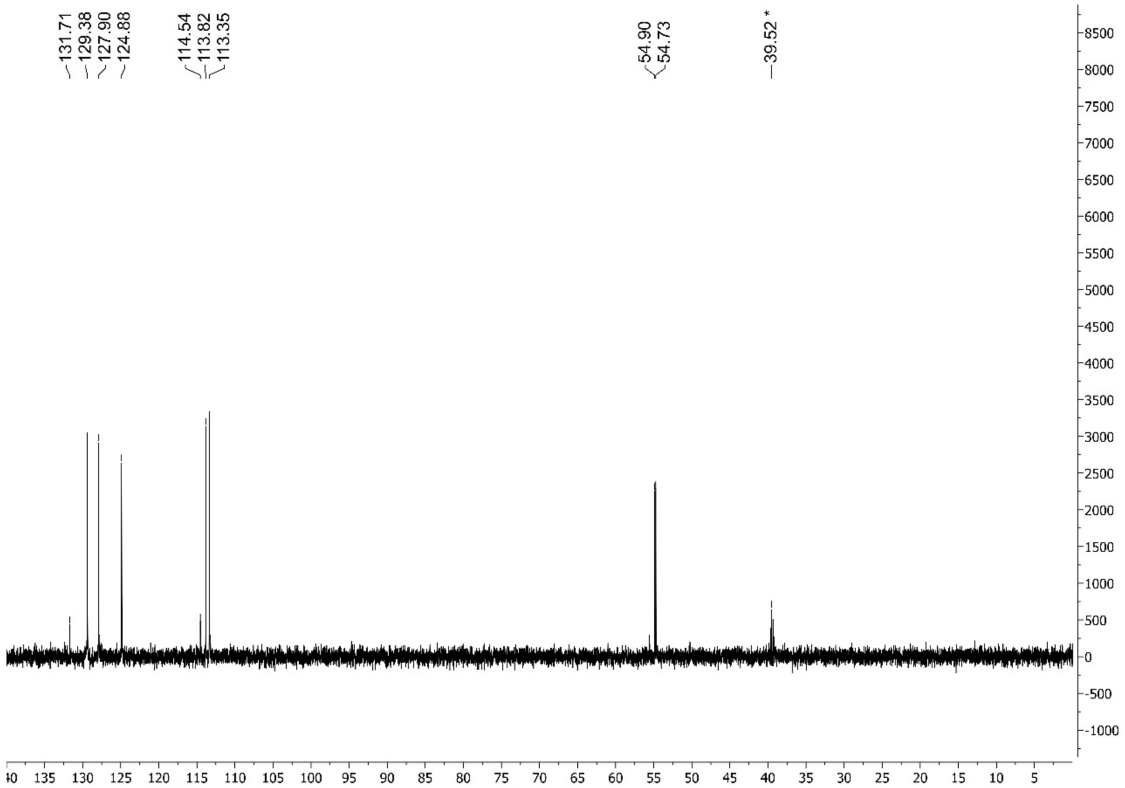

4 in $CDCl_3$, ^{1}H NMR (298 K, 700 MHz)

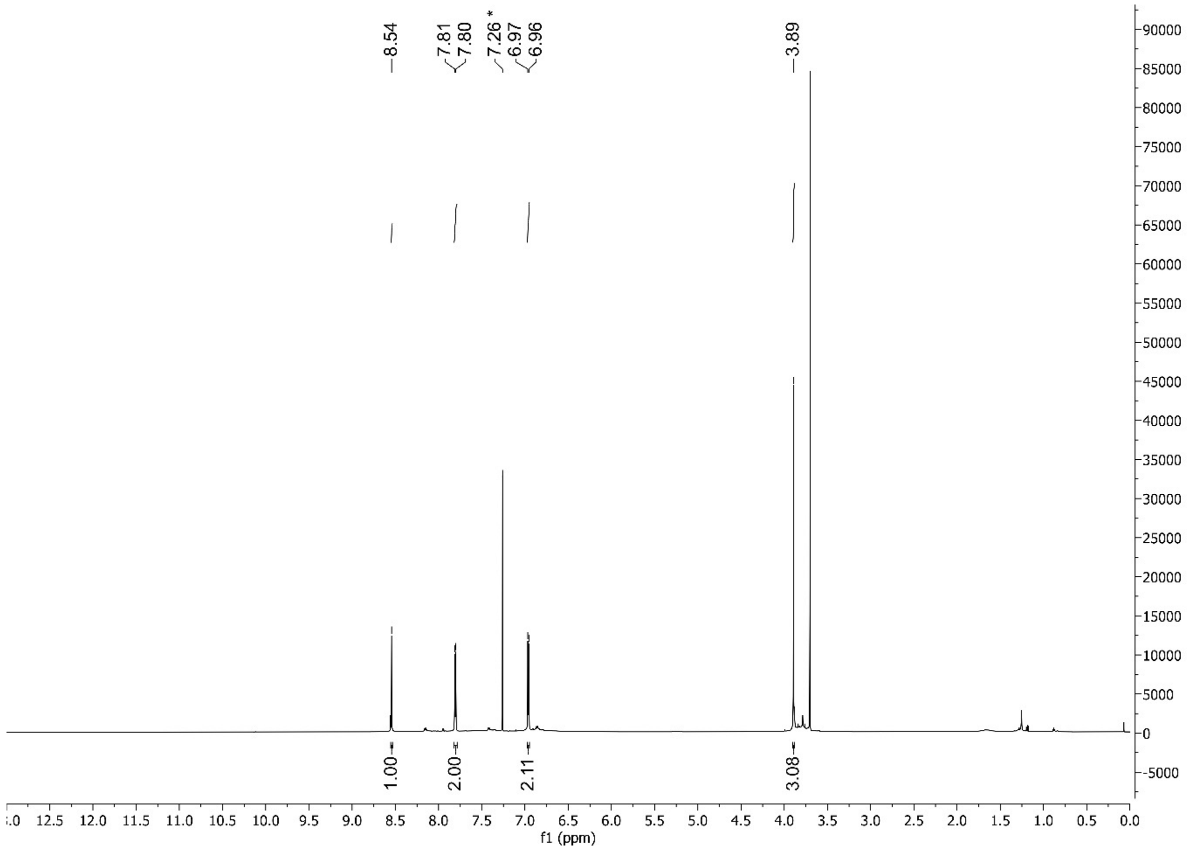

4 in $CDCl_3$, ^{13}C NMR (298 K, 176 MHz)

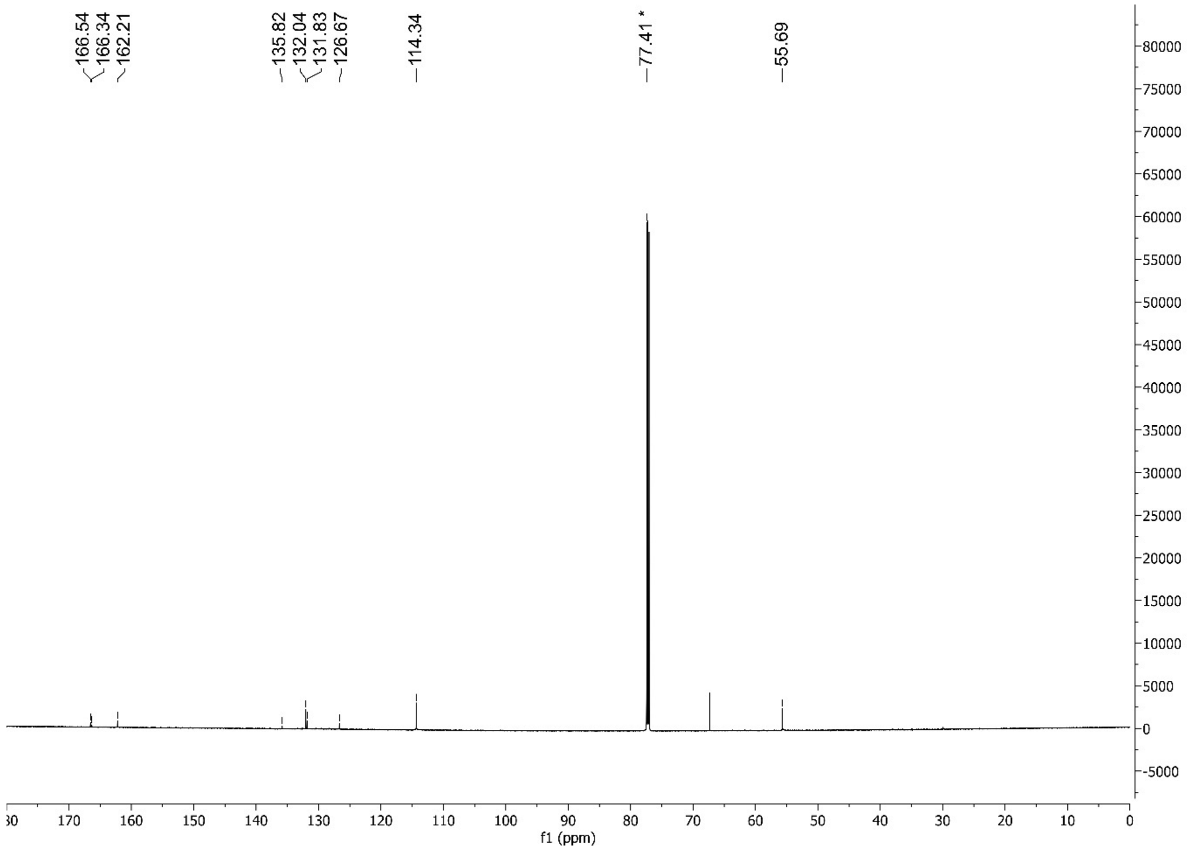

13 in $CDCl_3$, ^1H NMR (303 K, 500 MHz)

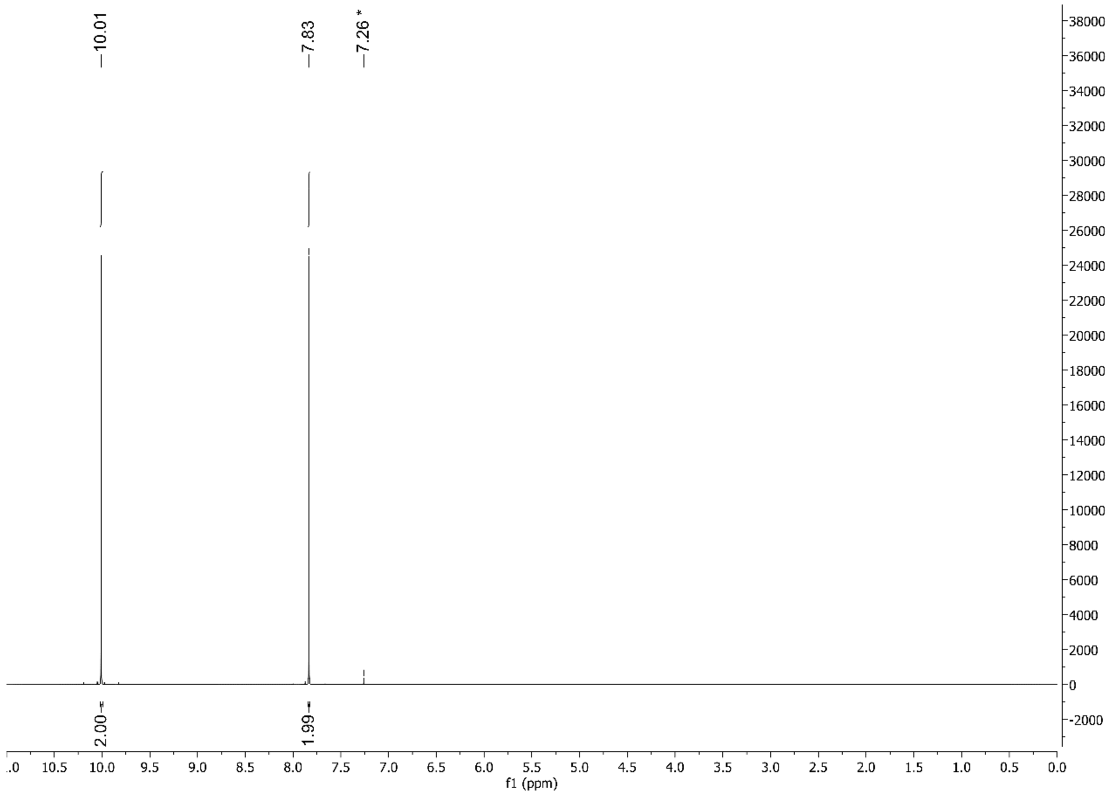

13 in $CDCl_3$, ^{13}C NMR (303 K, 125 MHz)

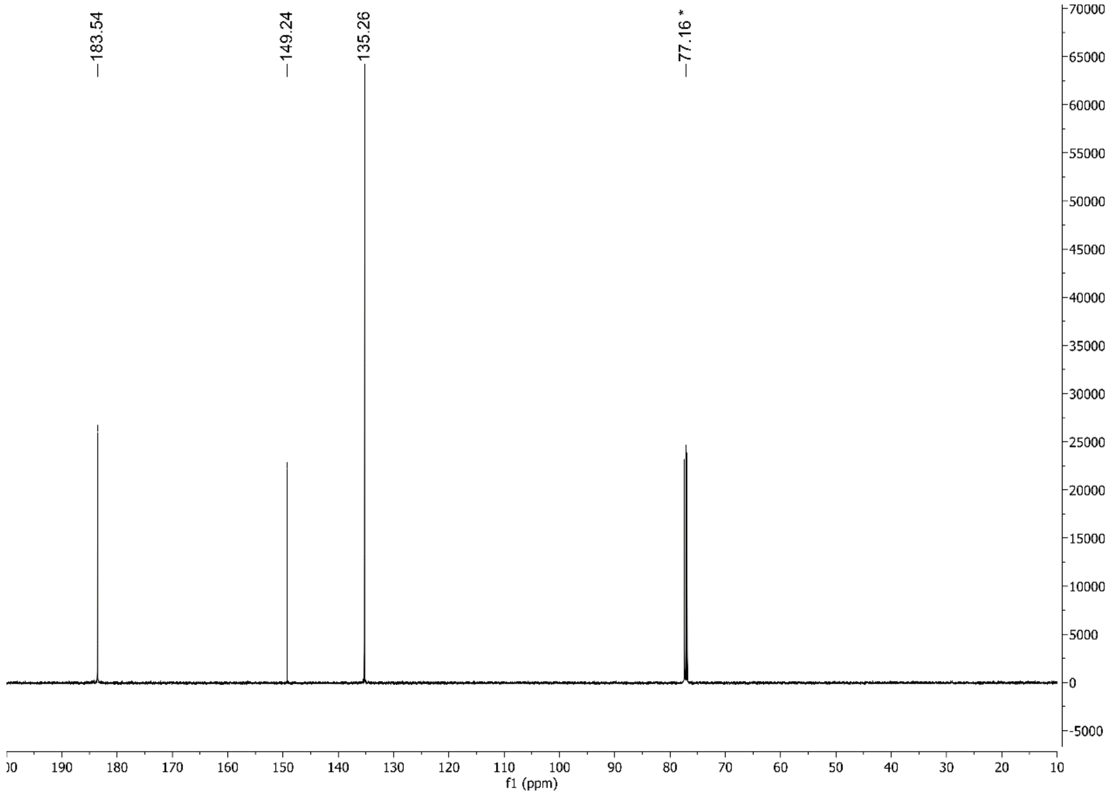

14 in DMSO, ^{1}H NMR (303 K, 300 MHz)

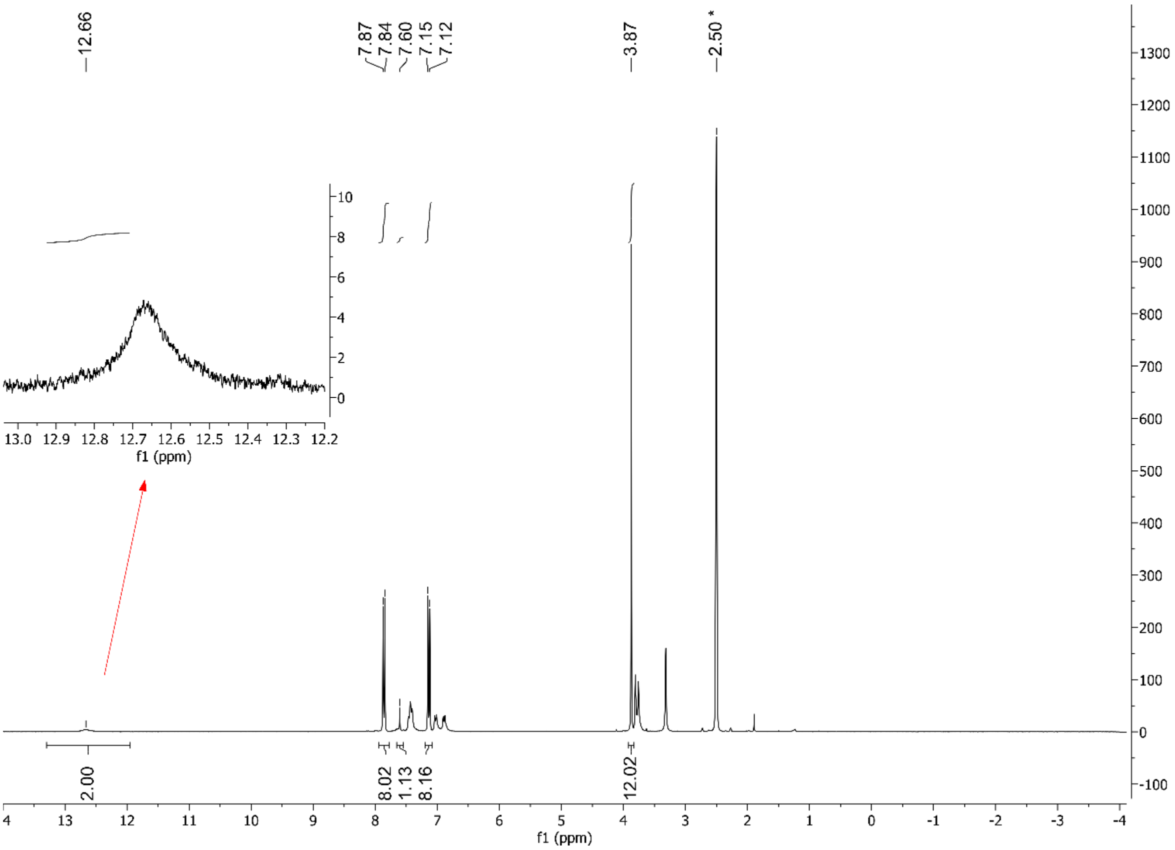

6 in $CDCl_3$, ^{1}H NMR (303 K, 300 MHz)

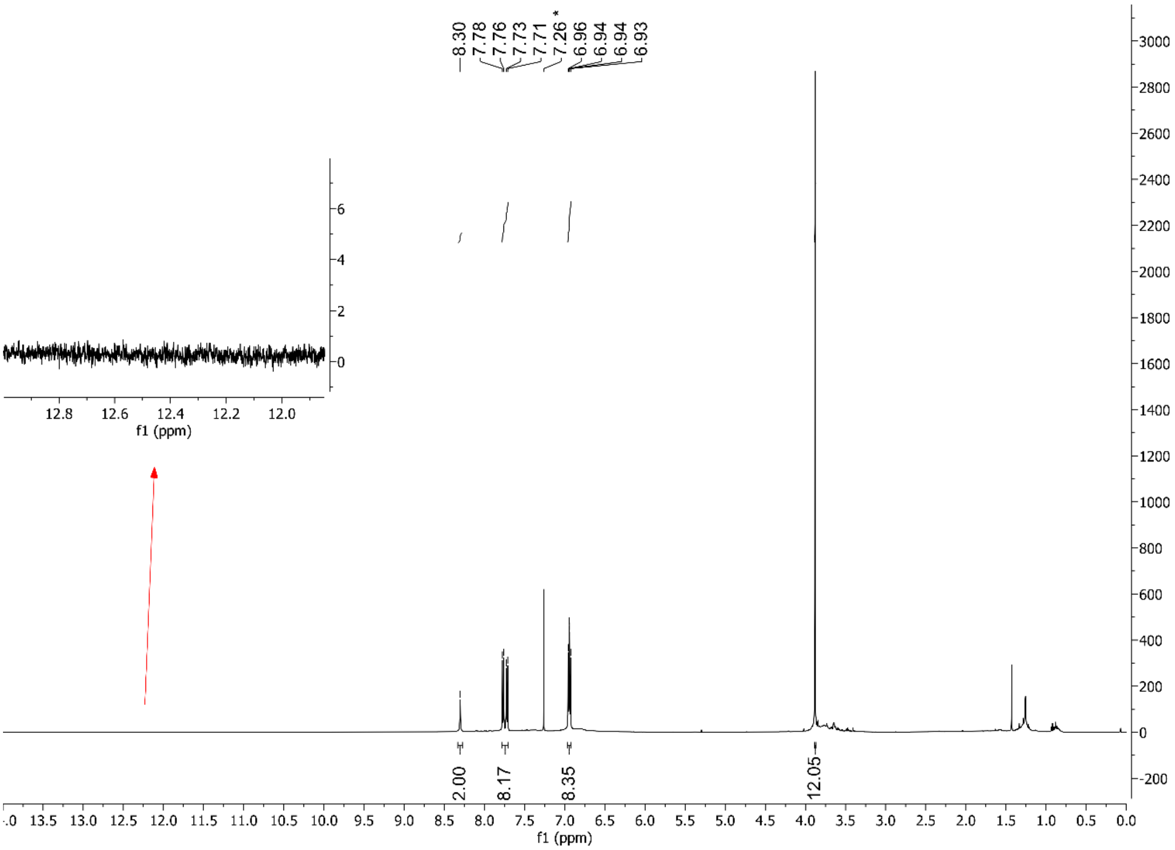

6 in $CDCl_3$, ^{13}C NMR (303 K, 125 MHz)

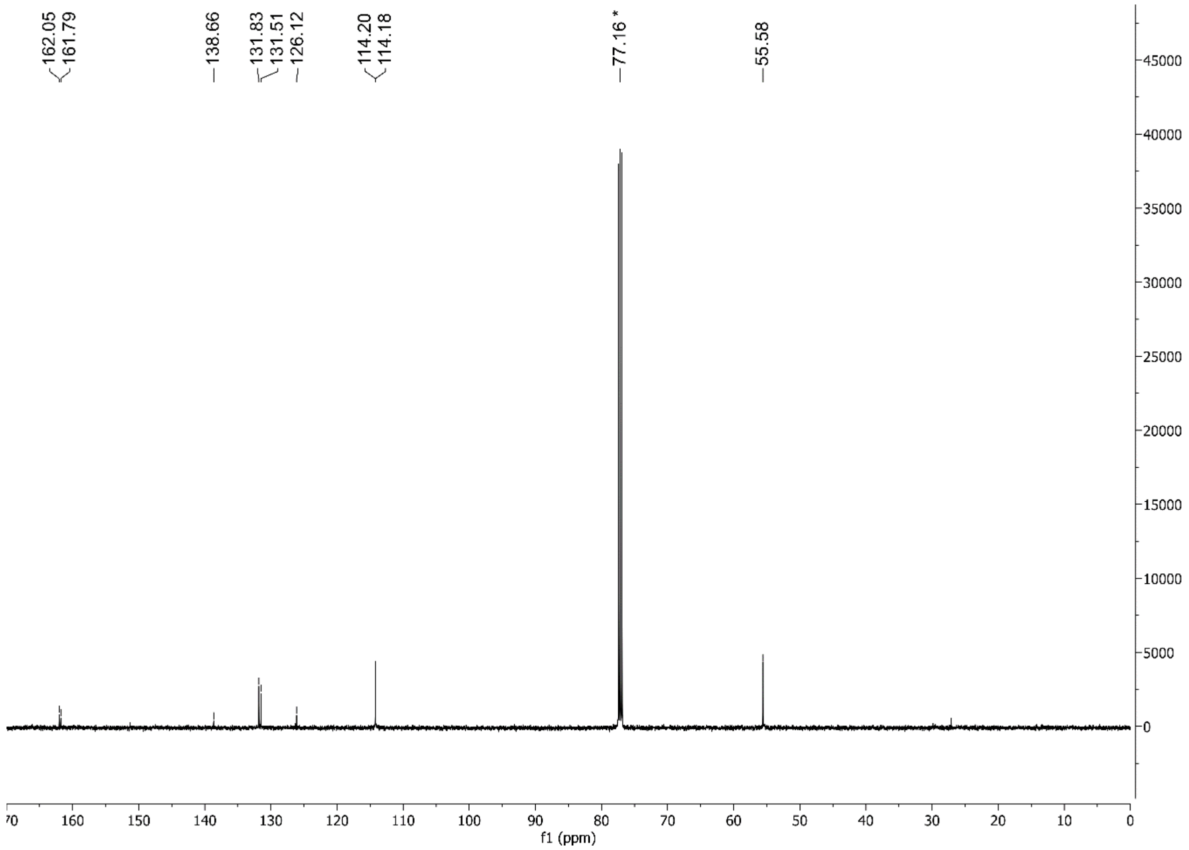

16 in DMSO, ^{1}H NMR (303 K, 500 MHz)

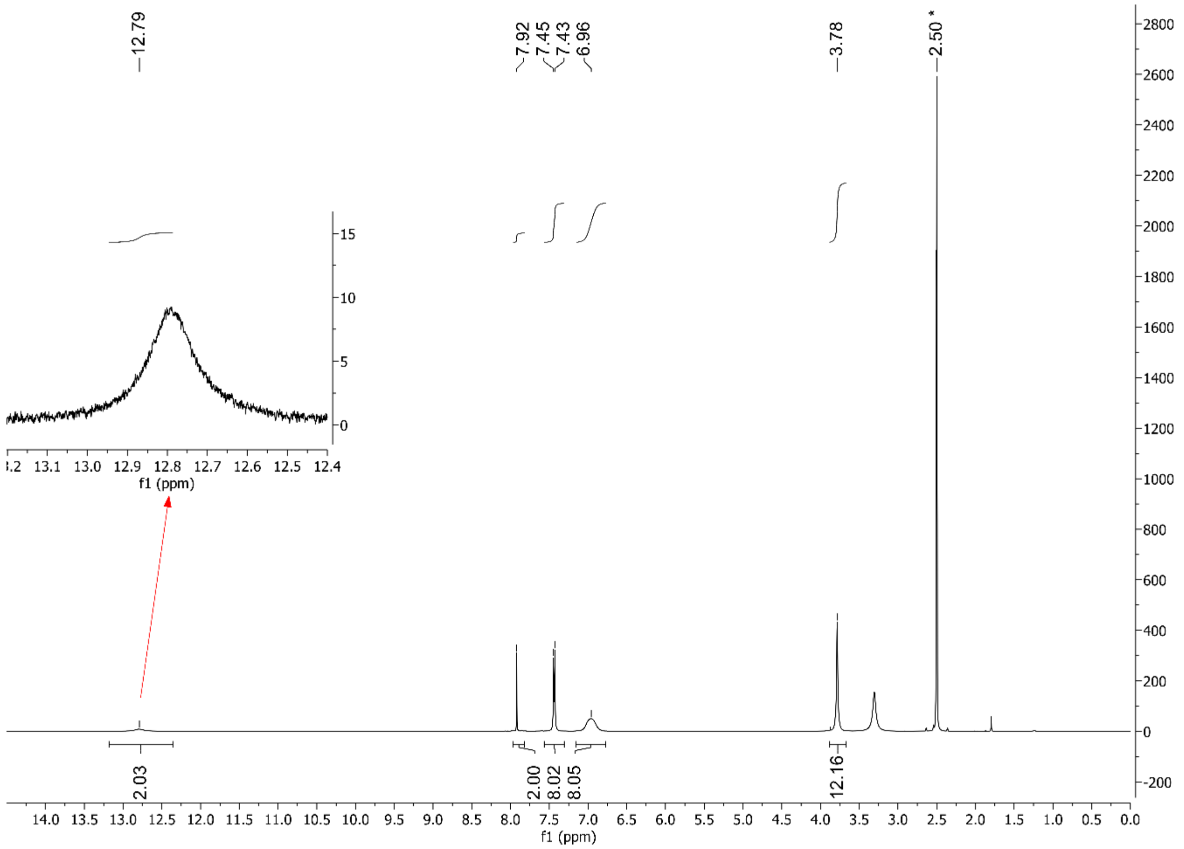

7 in $CDCl_3$, ^{1}H NMR (298 K, 700 MHz)

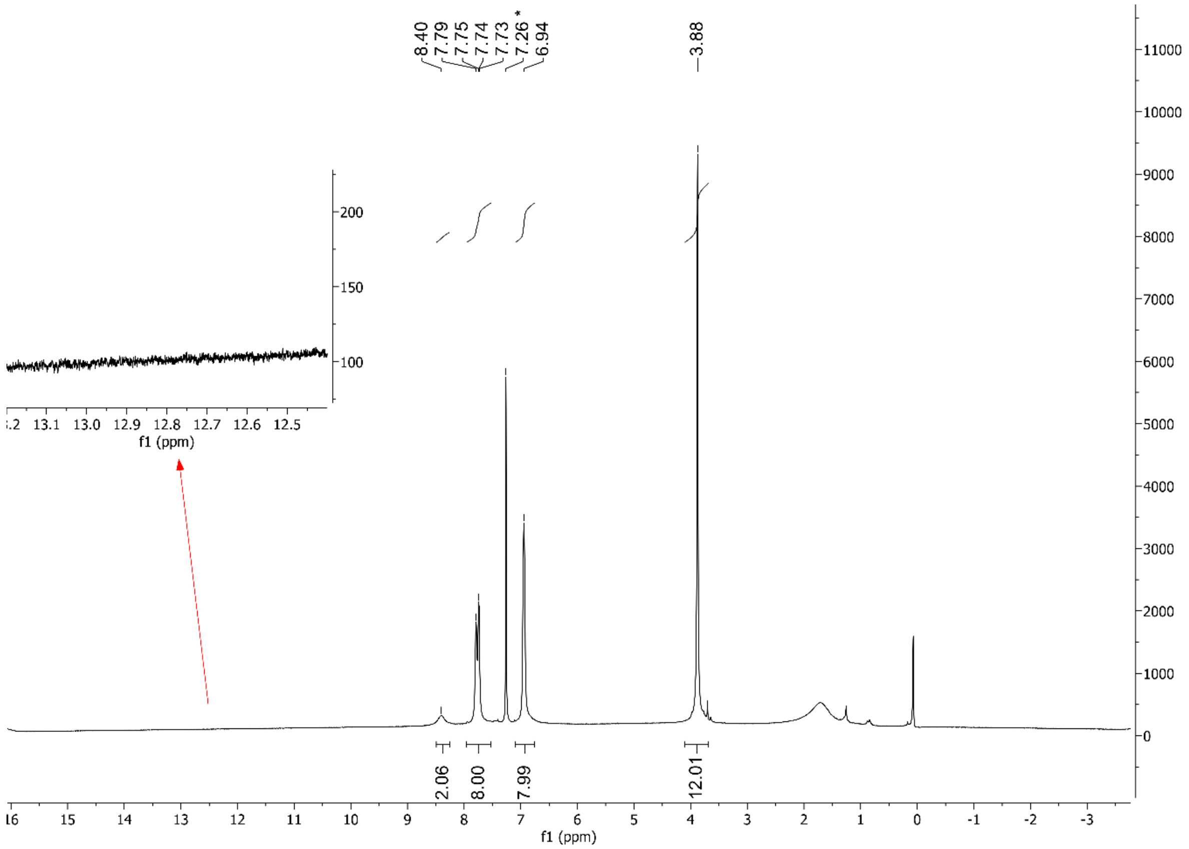

7 in $CDCl_3$, ^{13}C NMR (298 K, 175 MHz)

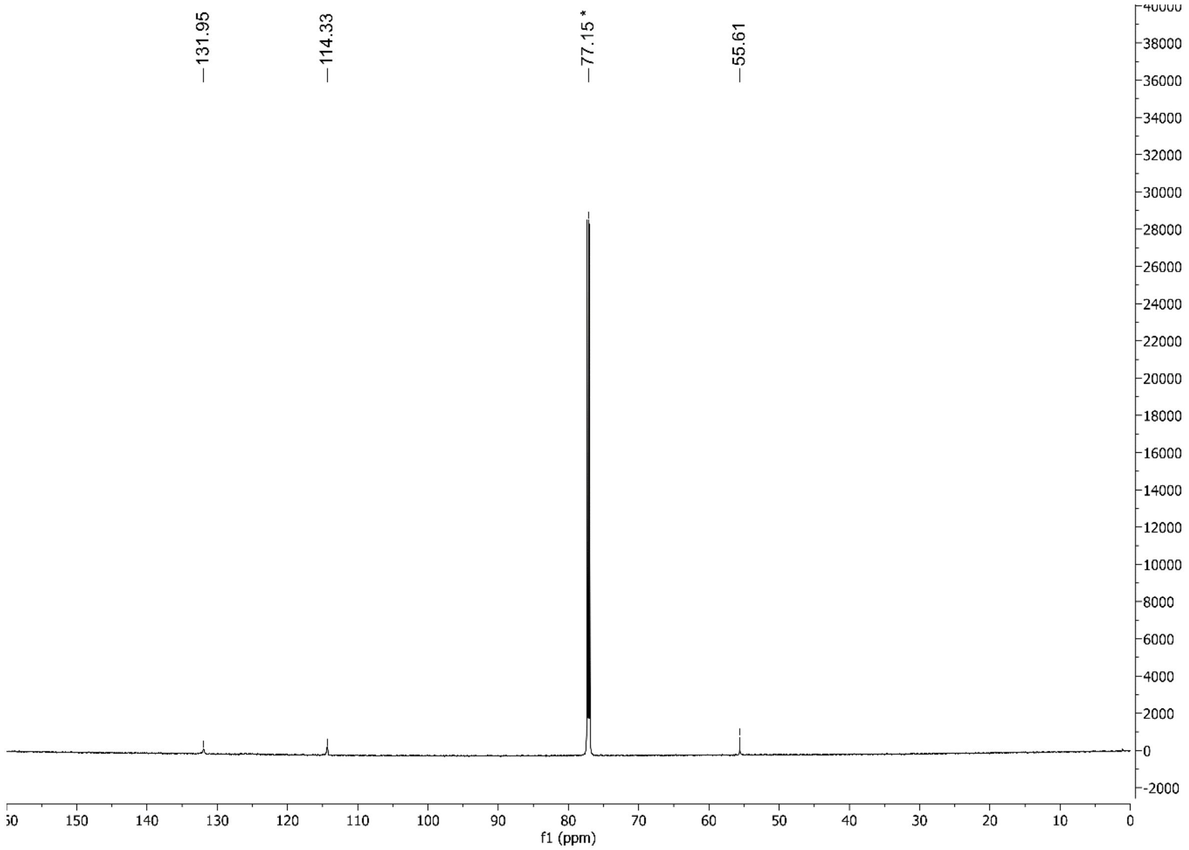

S1 in $CDCl_3$, ^1H NMR (300 K, 300 MHz)

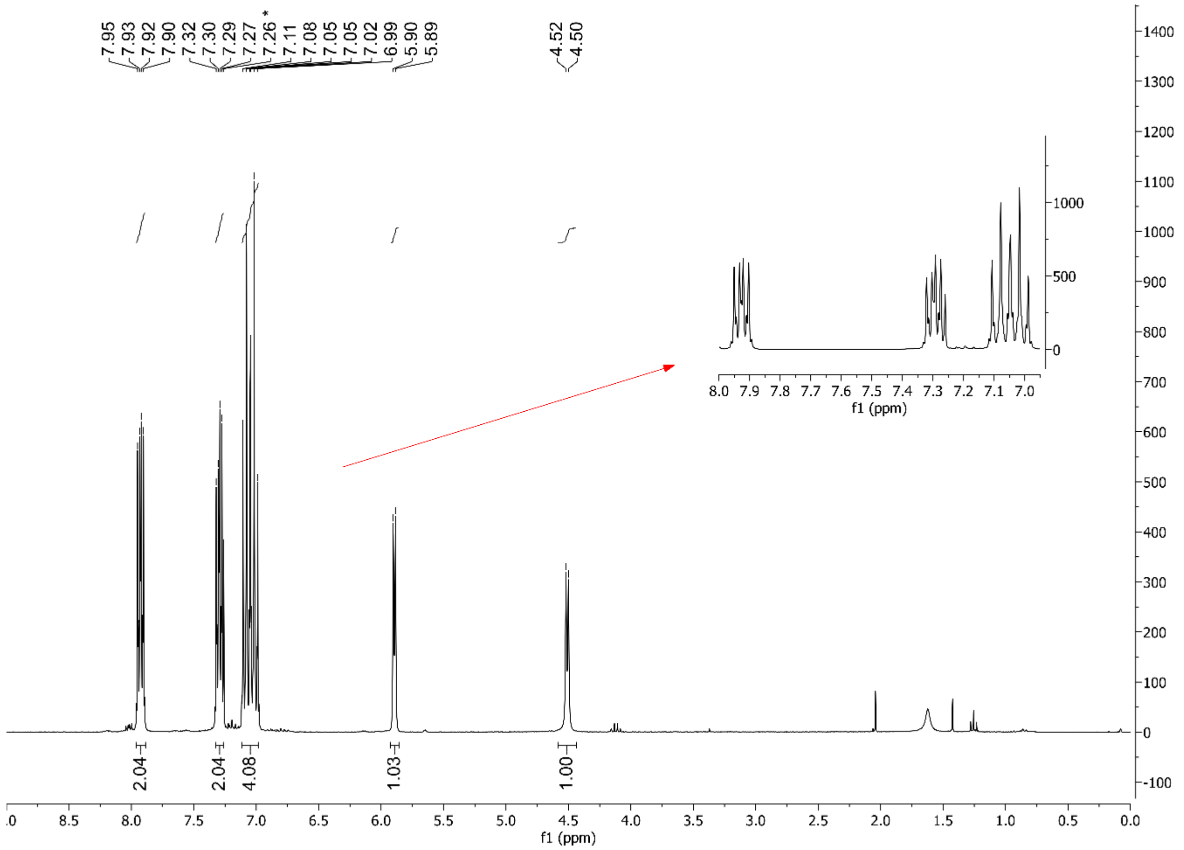

S1 in $CDCl_3$, ^{13}C NMR (303 K, 125 MHz)

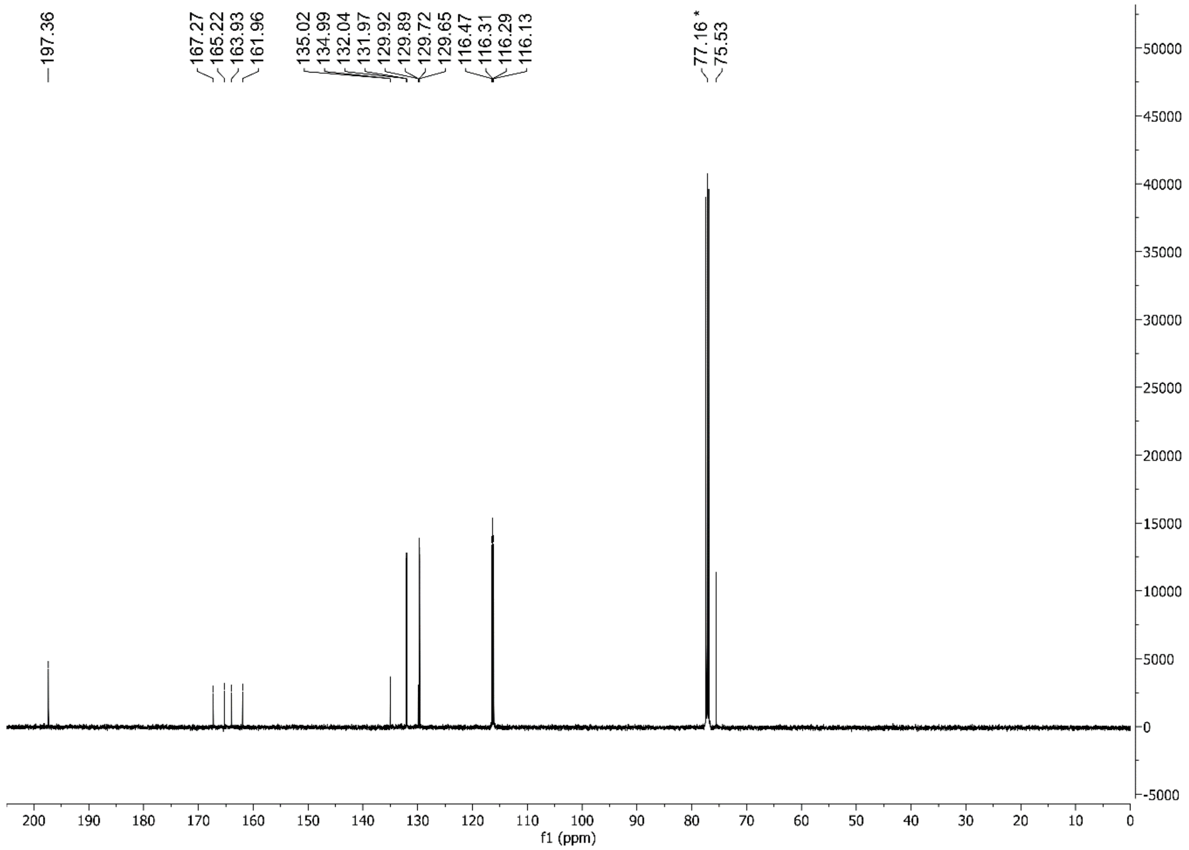

S2a in DMSO, ^{1}H NMR (298 K, 700 MHz)

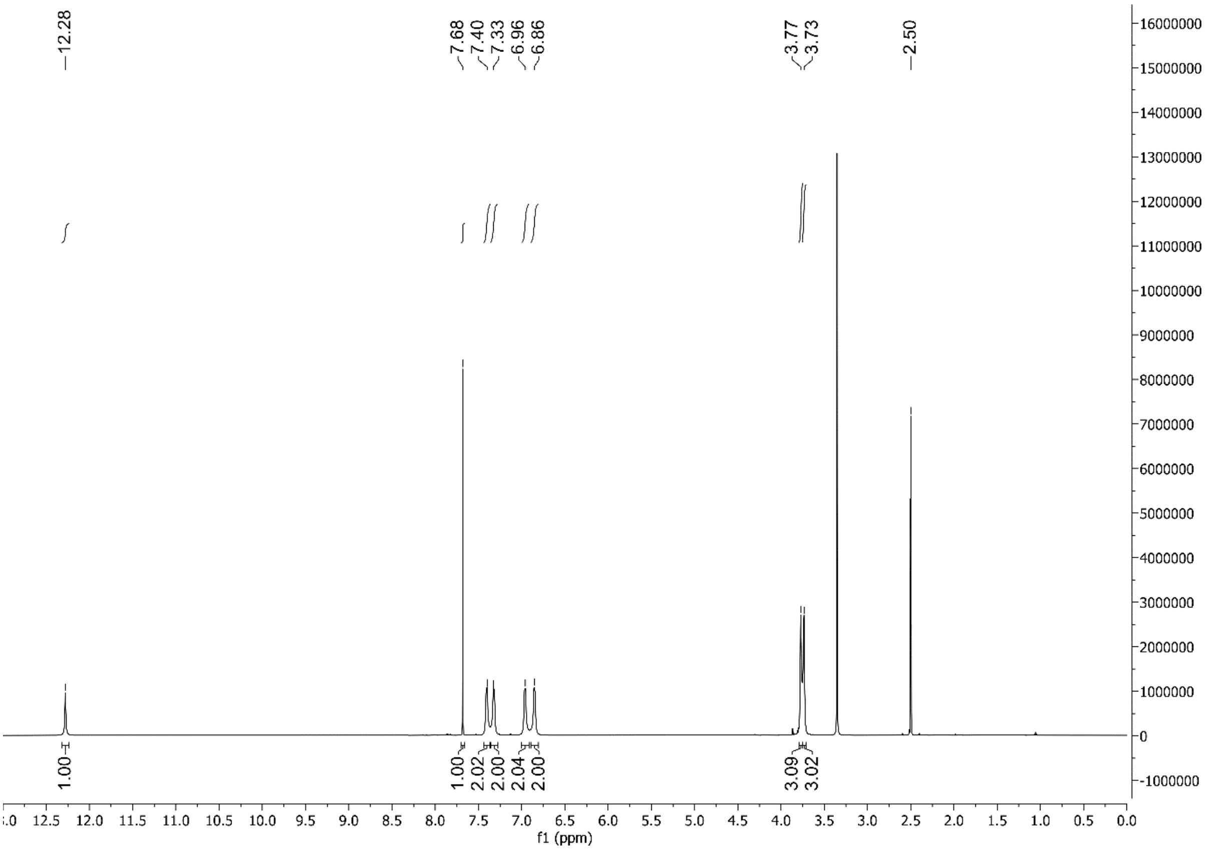

S2a in DMSO, ^{13}C NMR (303 K, 125 MHz)

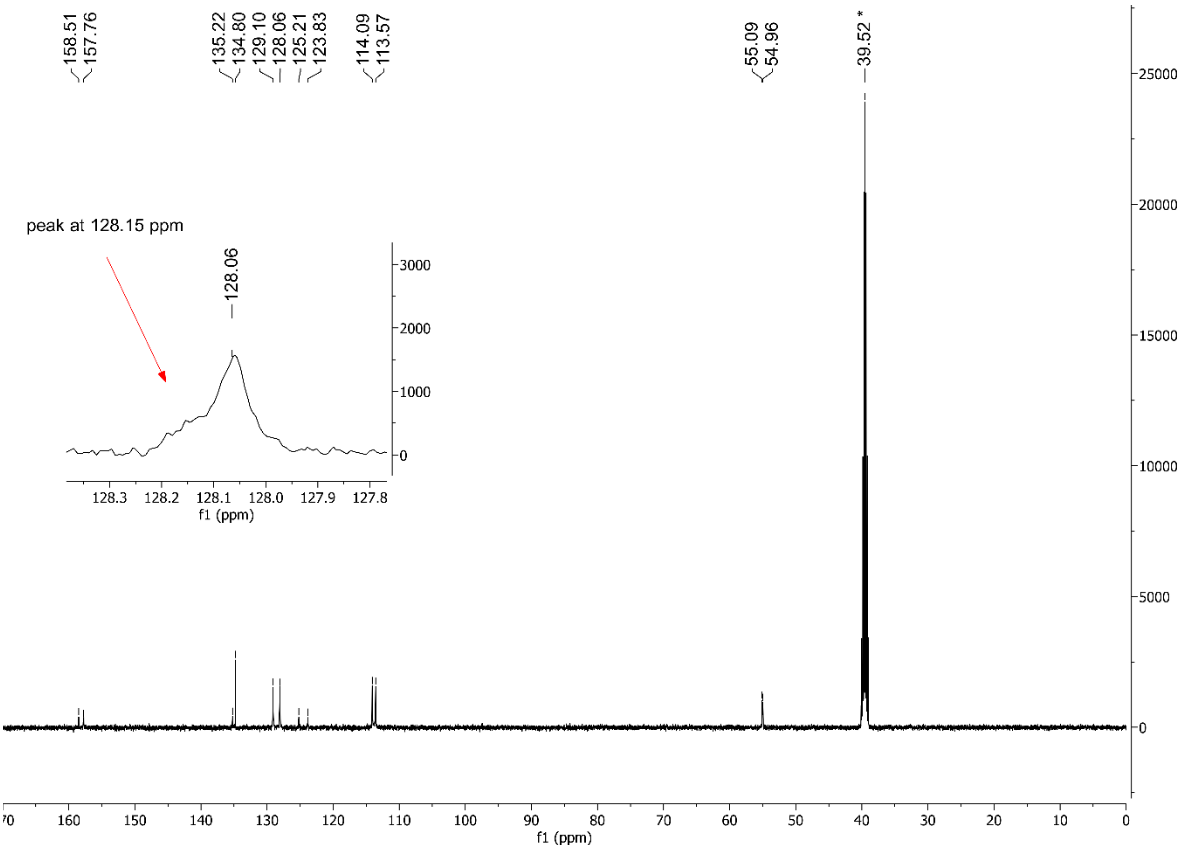

S2b in DMSO, ^{1}H NMR (303 K, 500 MHz)

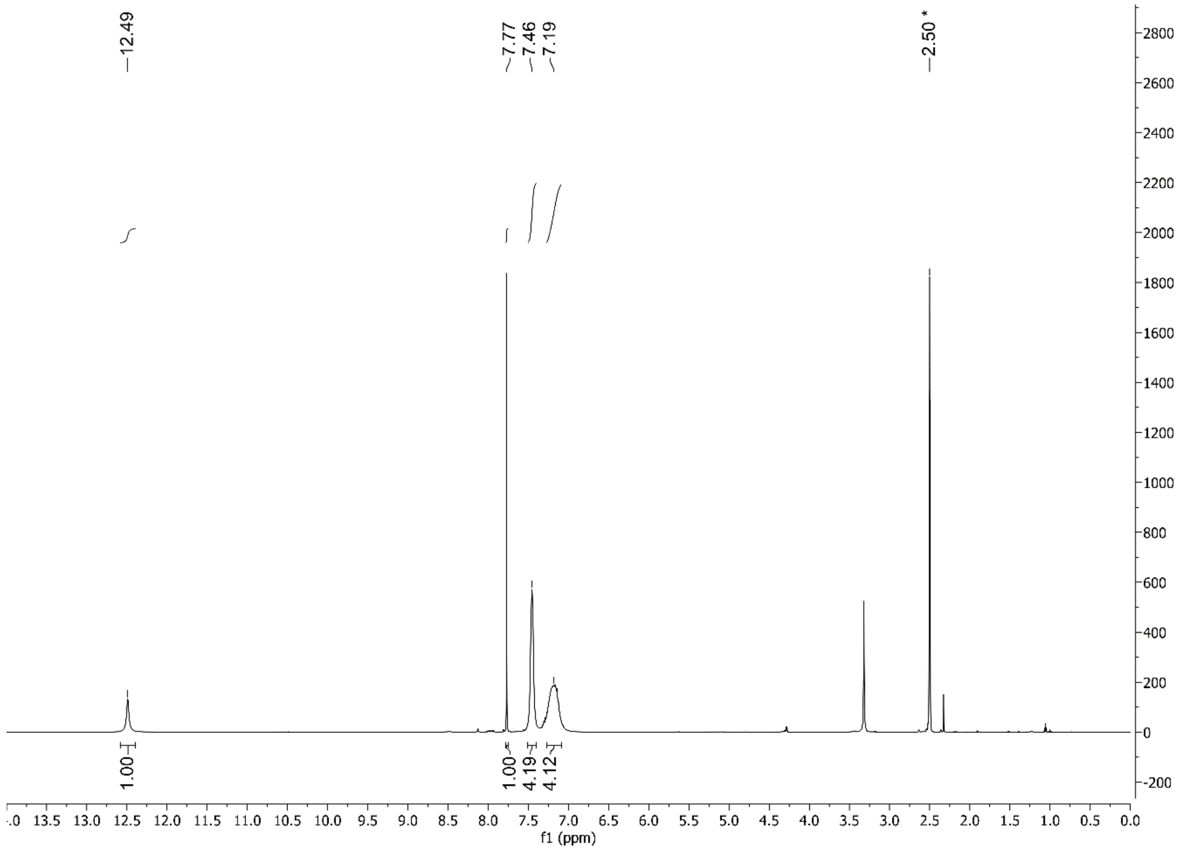

S2b in DMSO, ^{13}C NMR (303 K, 125 MHz)

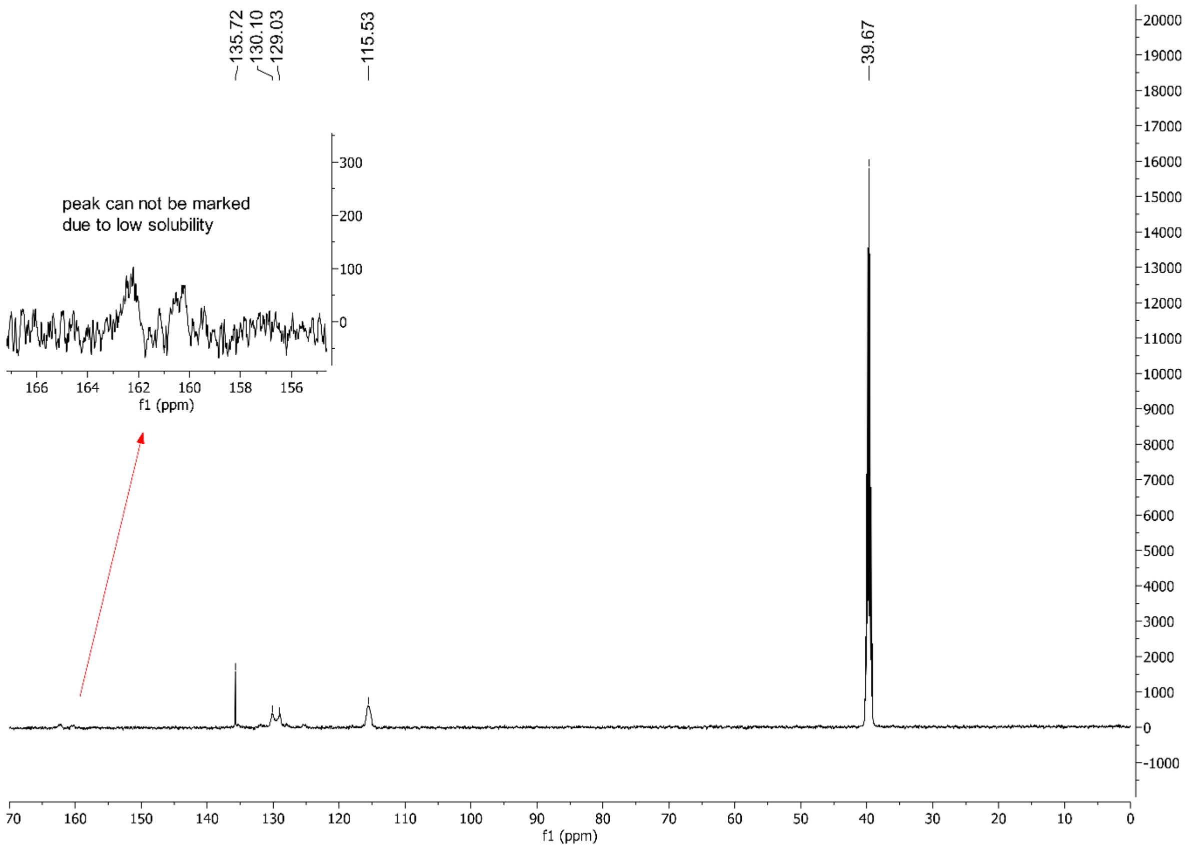

S3a in $CDCl_3$, ^1H NMR (303 K, 500 MHz)

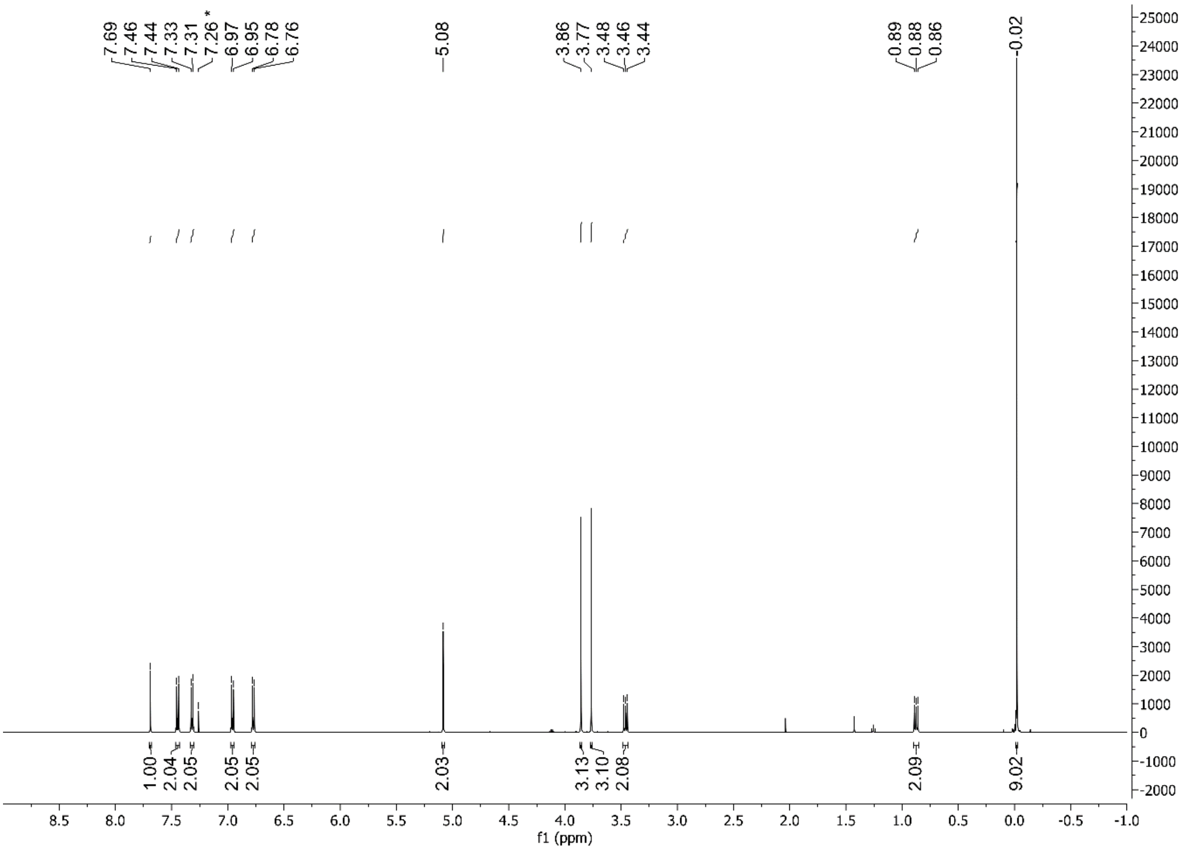

S3a in $CDCl_3$, ^{13}C NMR (303 K, 125 MHz)

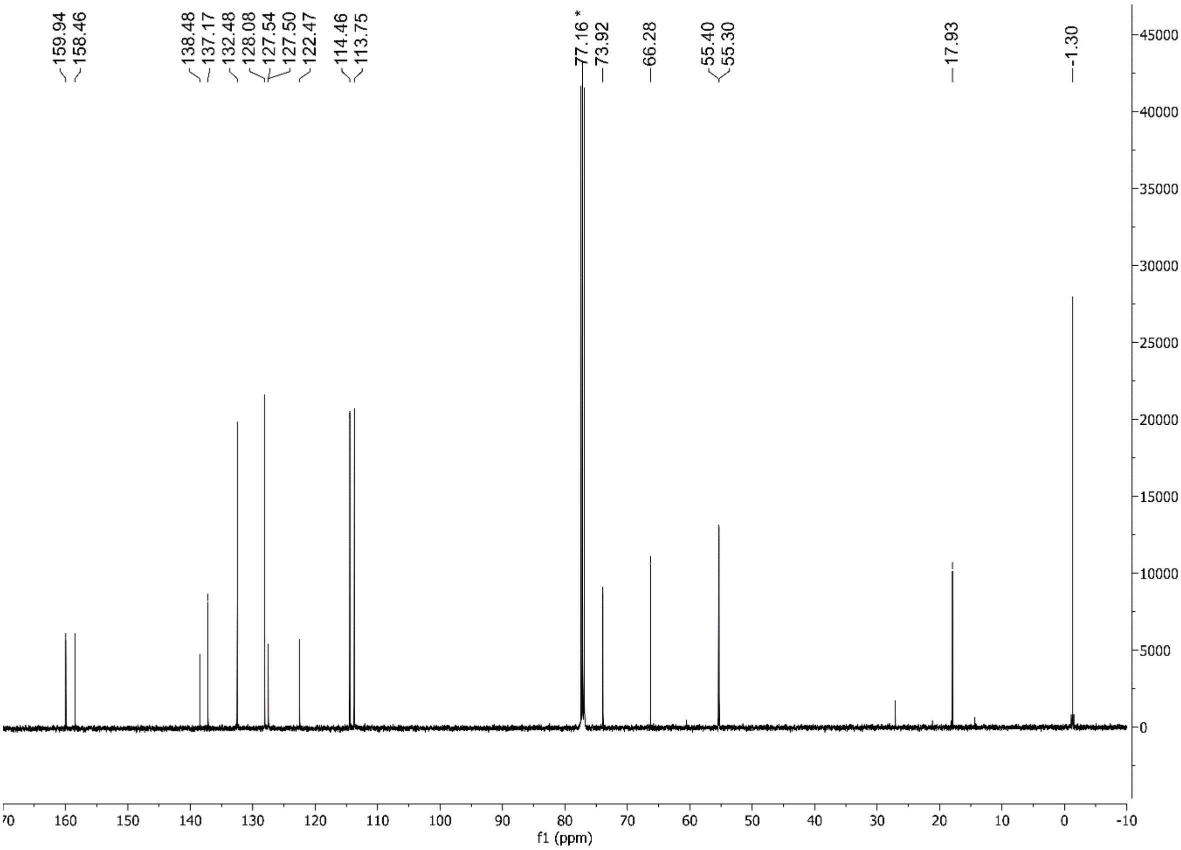

S3b in $CDCl_3$, ^1H NMR (303 K, 500 MHz)

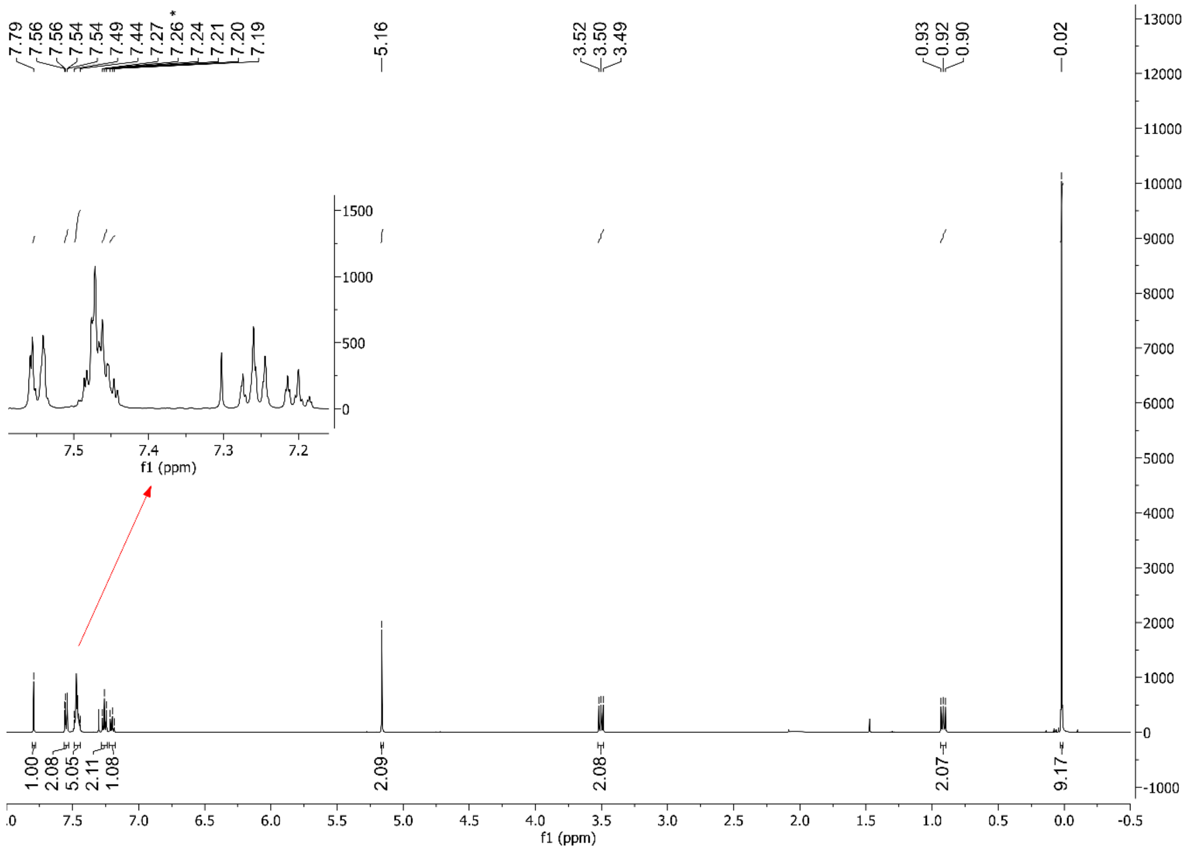

S3b in $CDCl_3$, ^{13}C NMR (303 K, 125 MHz)

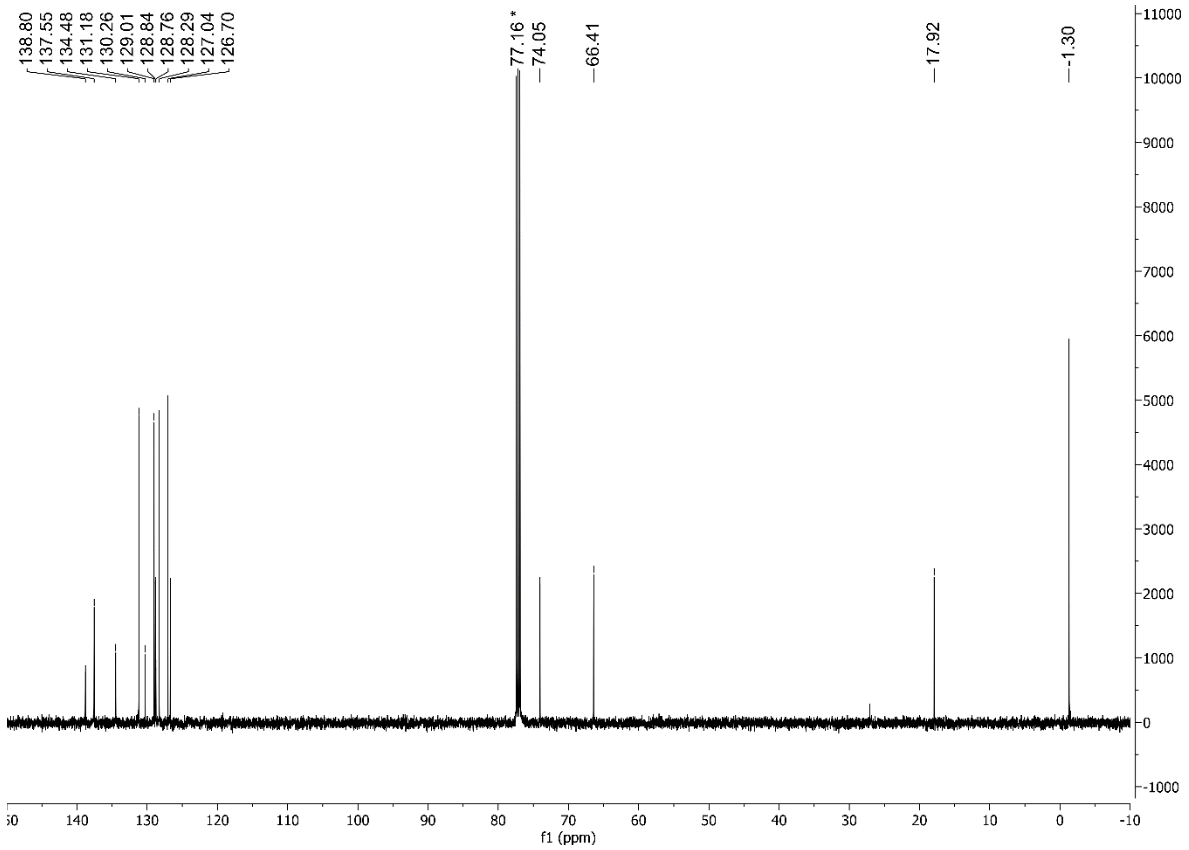

S3c in $CDCl_3$, ^1H NMR (303 K, 125 MHz)

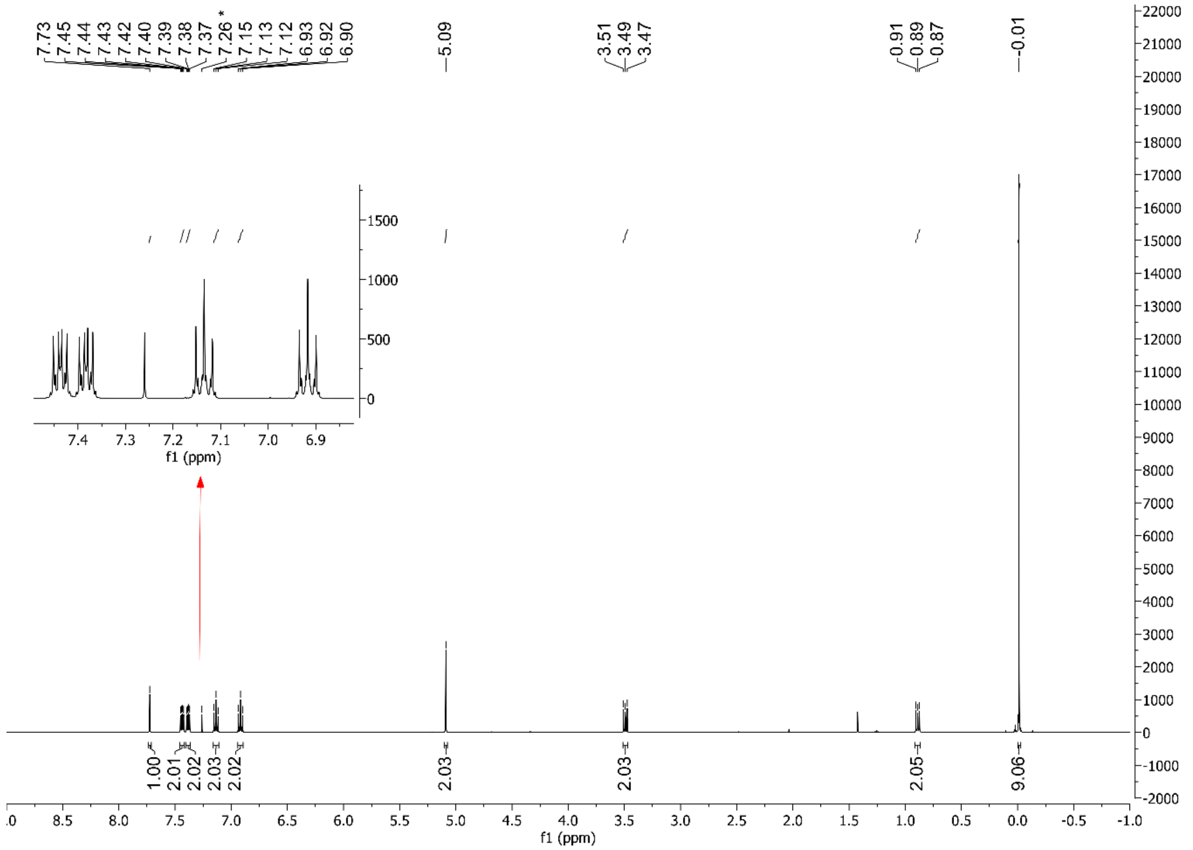

S3c in $CDCl_3$, ^{13}C NMR (303 K, 125 MHz)

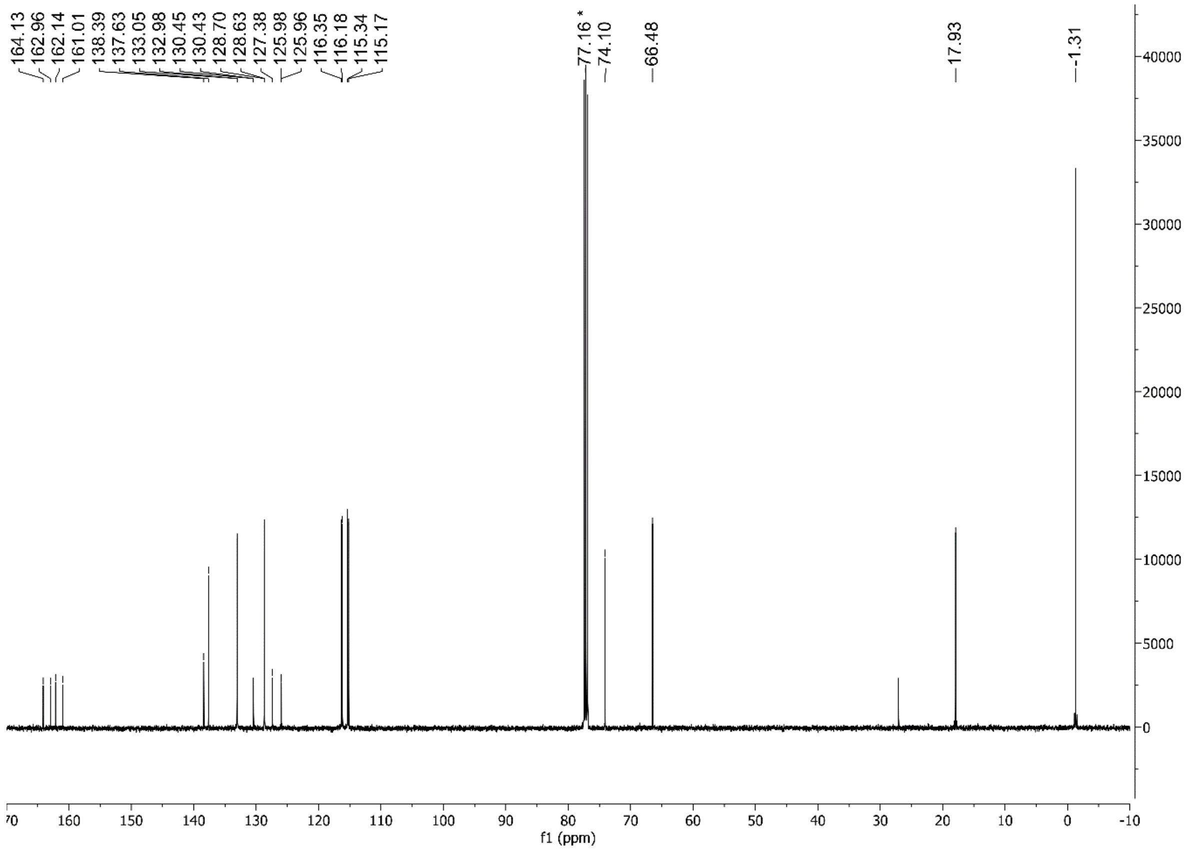

20a in $CDCl_3$, ^1H NMR (303 K, 500 MHz)

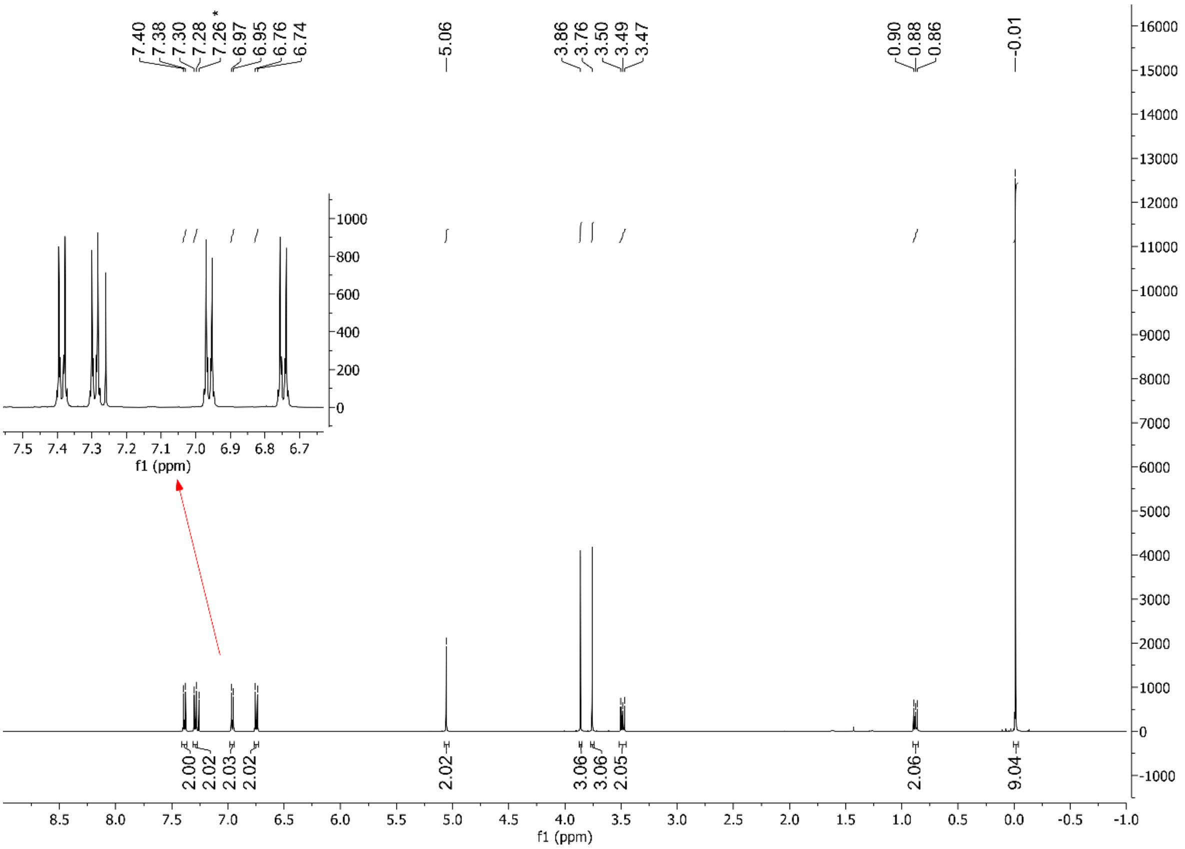

20a in $CDCl_3$, ^{13}C NMR (303 K, 125 MHz)

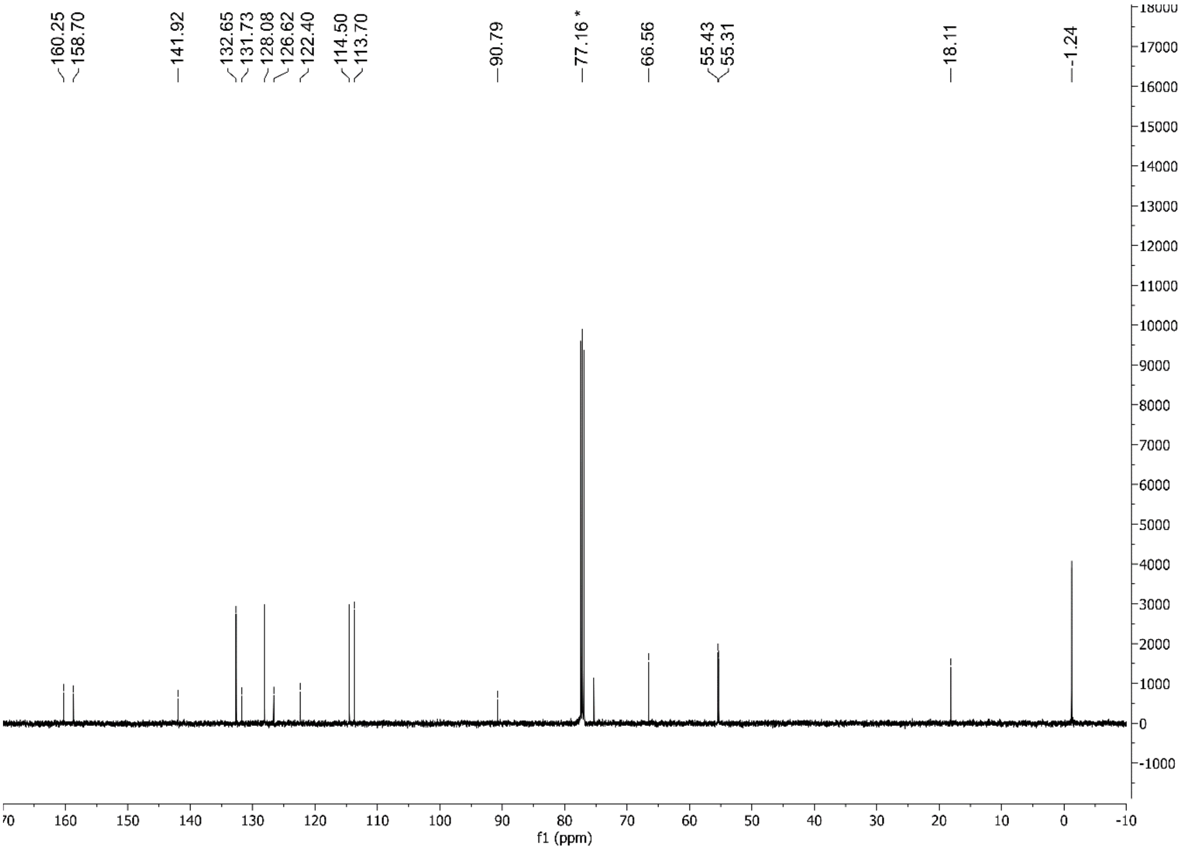

20b in $CDCl_3$, ^{1}H NMR (300 K, 300 MHz)

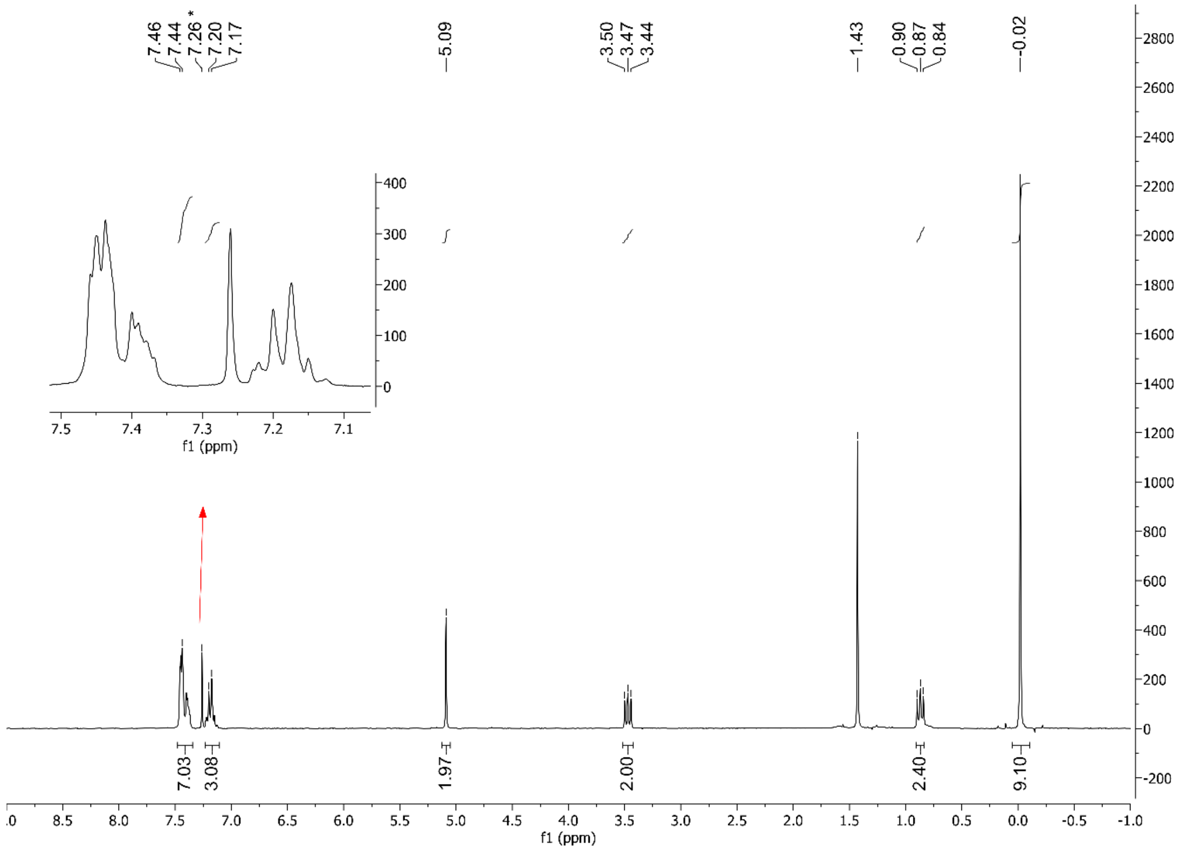

20b in $CDCl_3$, ^{13}C NMR (300 K, 125 MHz)

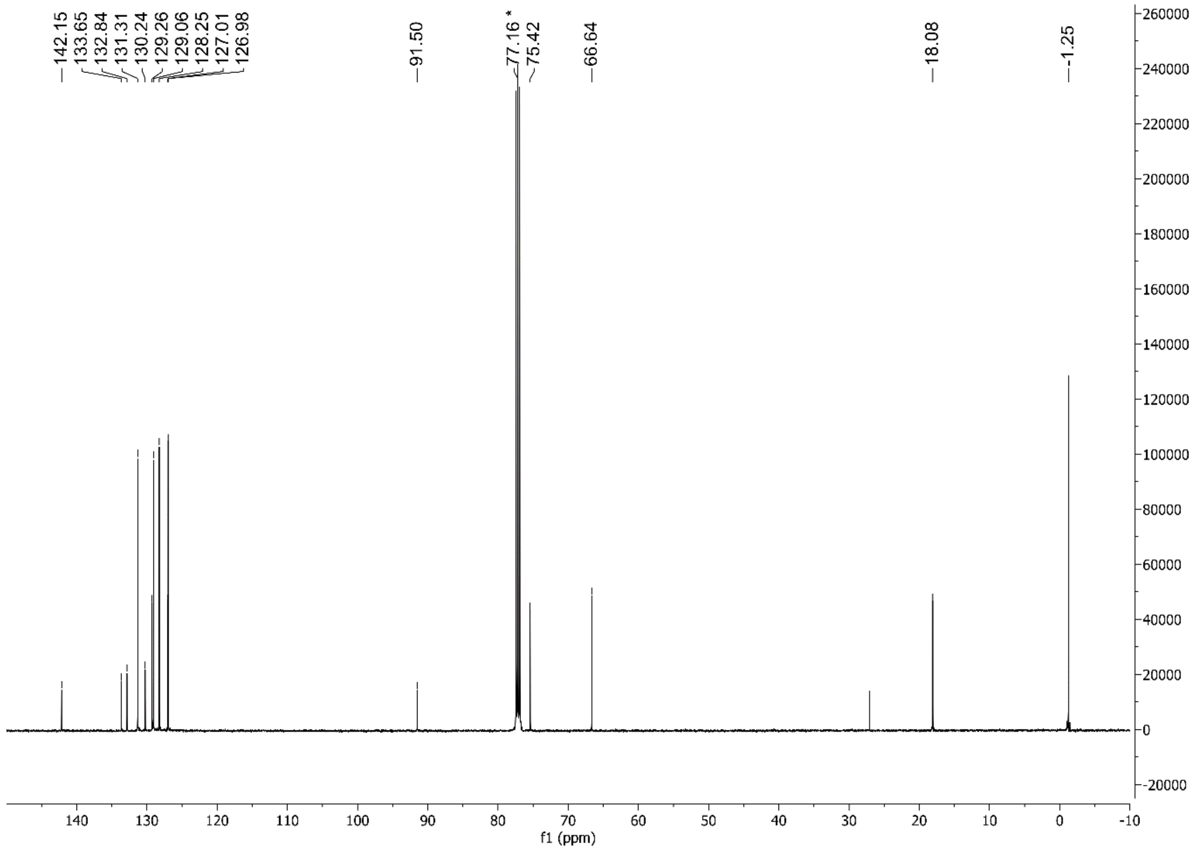

20c in $CDCl_3$, ^1H NMR (303 K, 500 MHz)

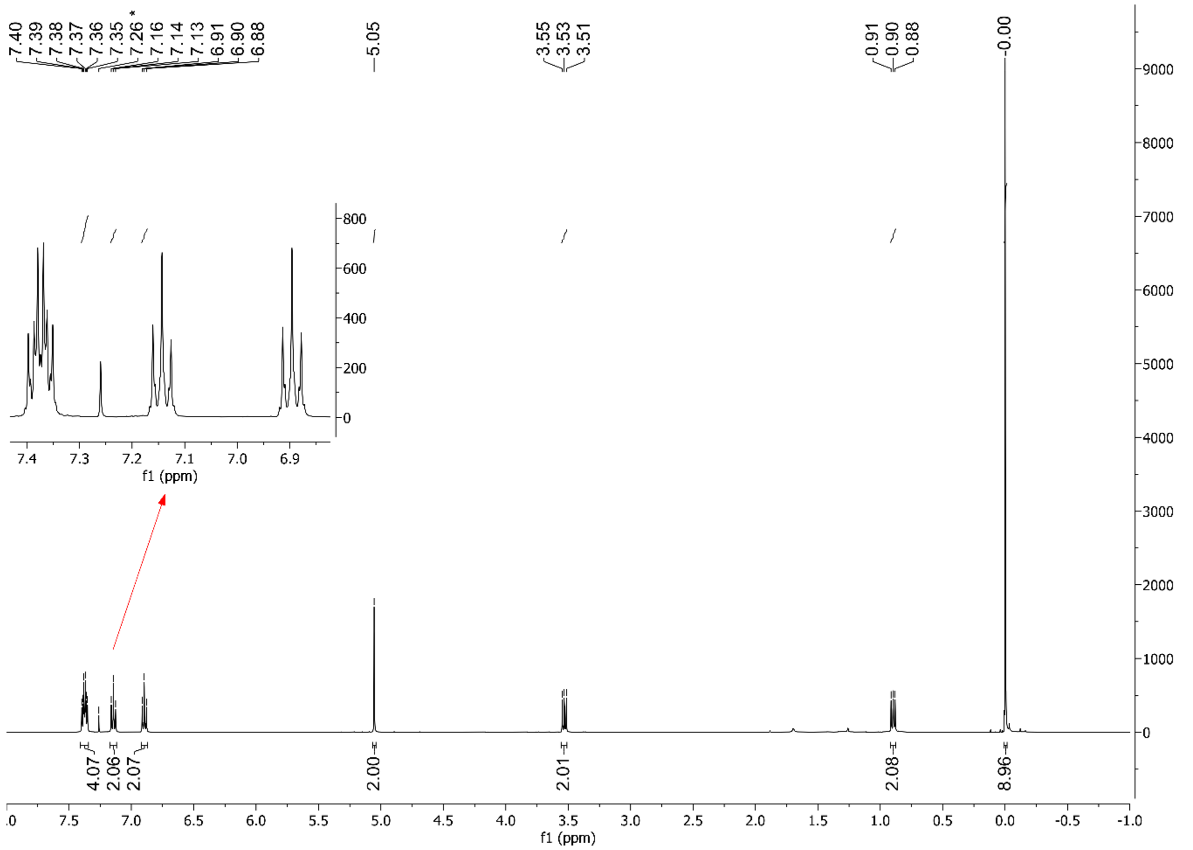

20c in $CDCl_3$, ^{13}C NMR (303 K, 125 MHz)

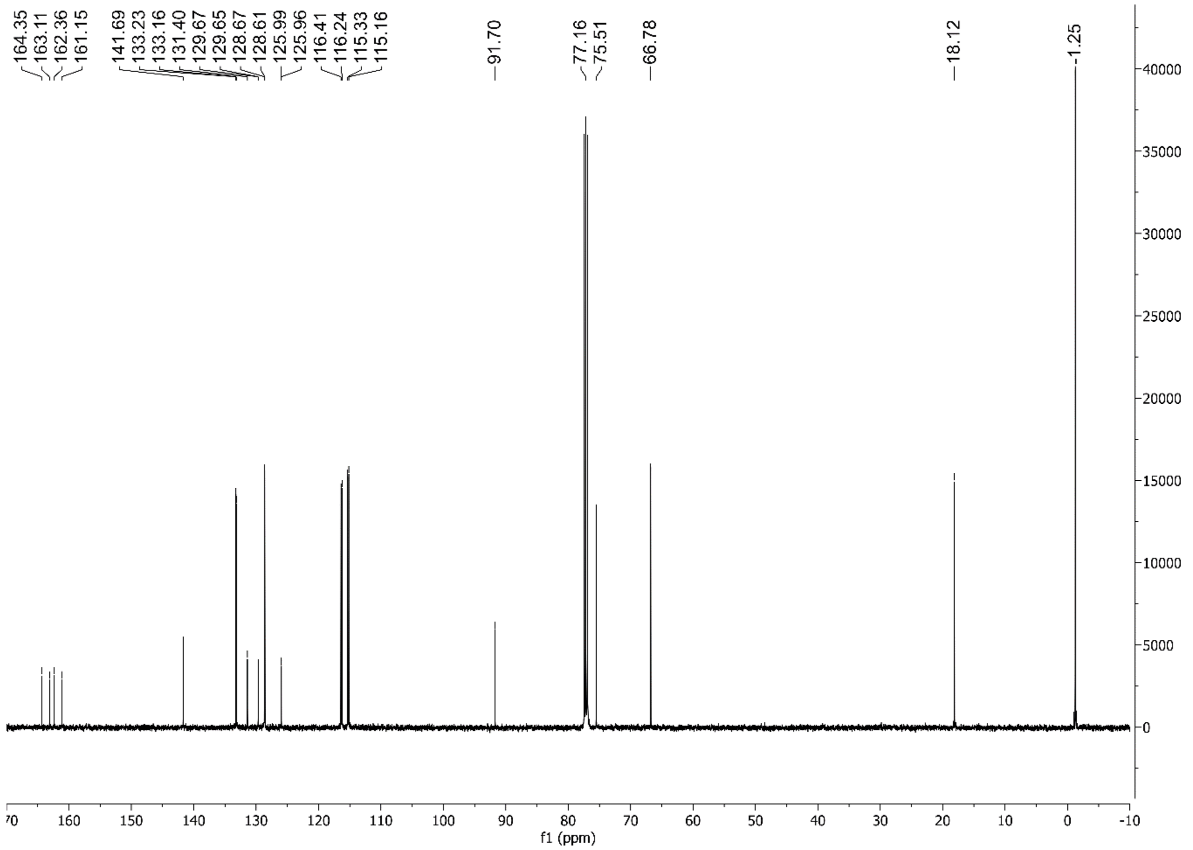

19 in $CDCl_3$, ^1H NMR (303 K, 500 MHz)

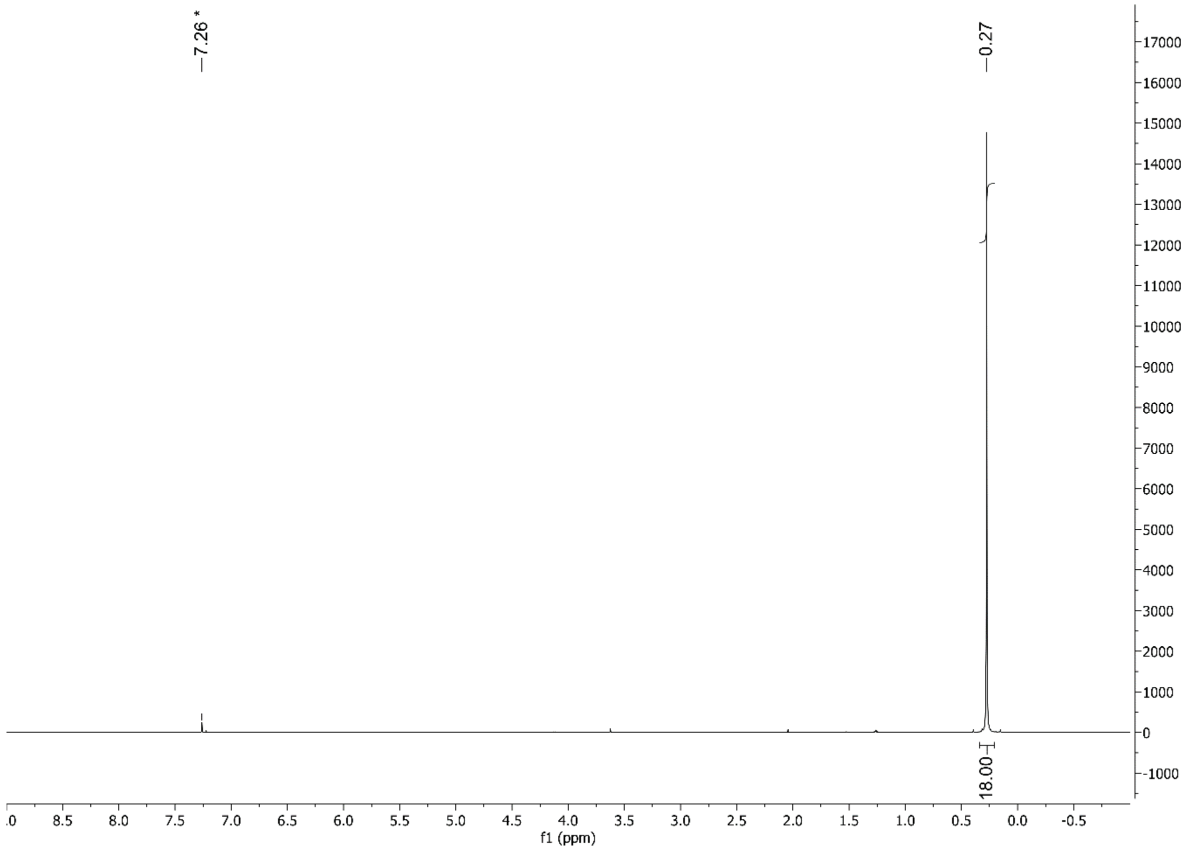

19 in $CDCl_3$, ^{13}C NMR (303 K, 125 MHz)

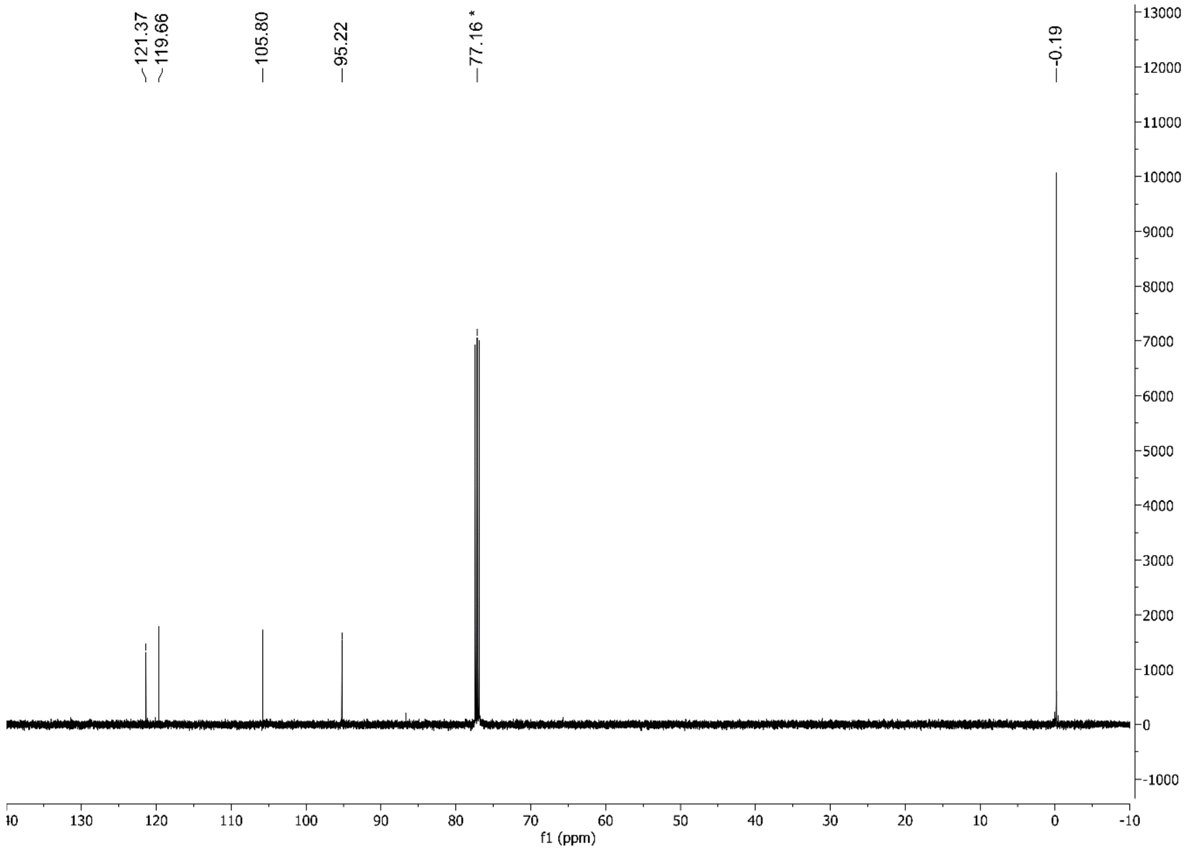

21a in CDCl3, ^{1}H NMR (303 K, 500 MHz)

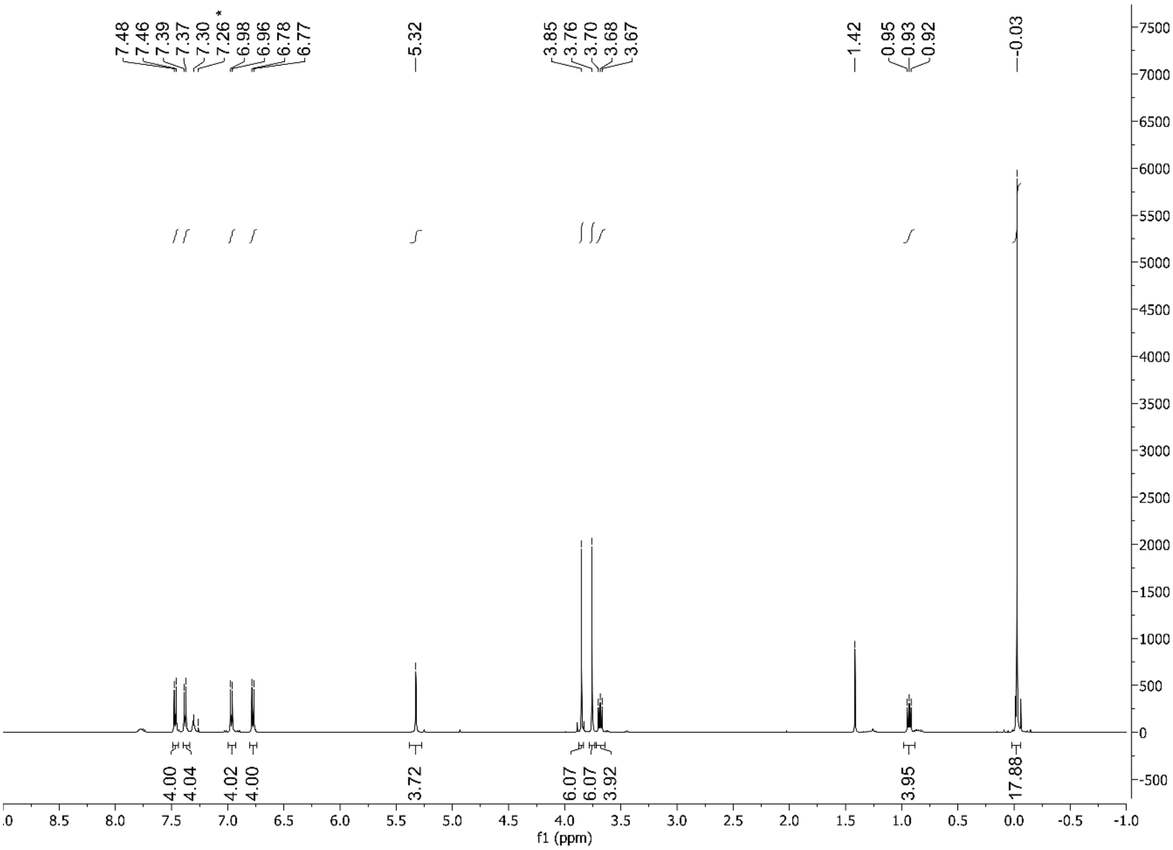

21a in CDCl3, ^{13}C NMR (303 K, 125 MHz)

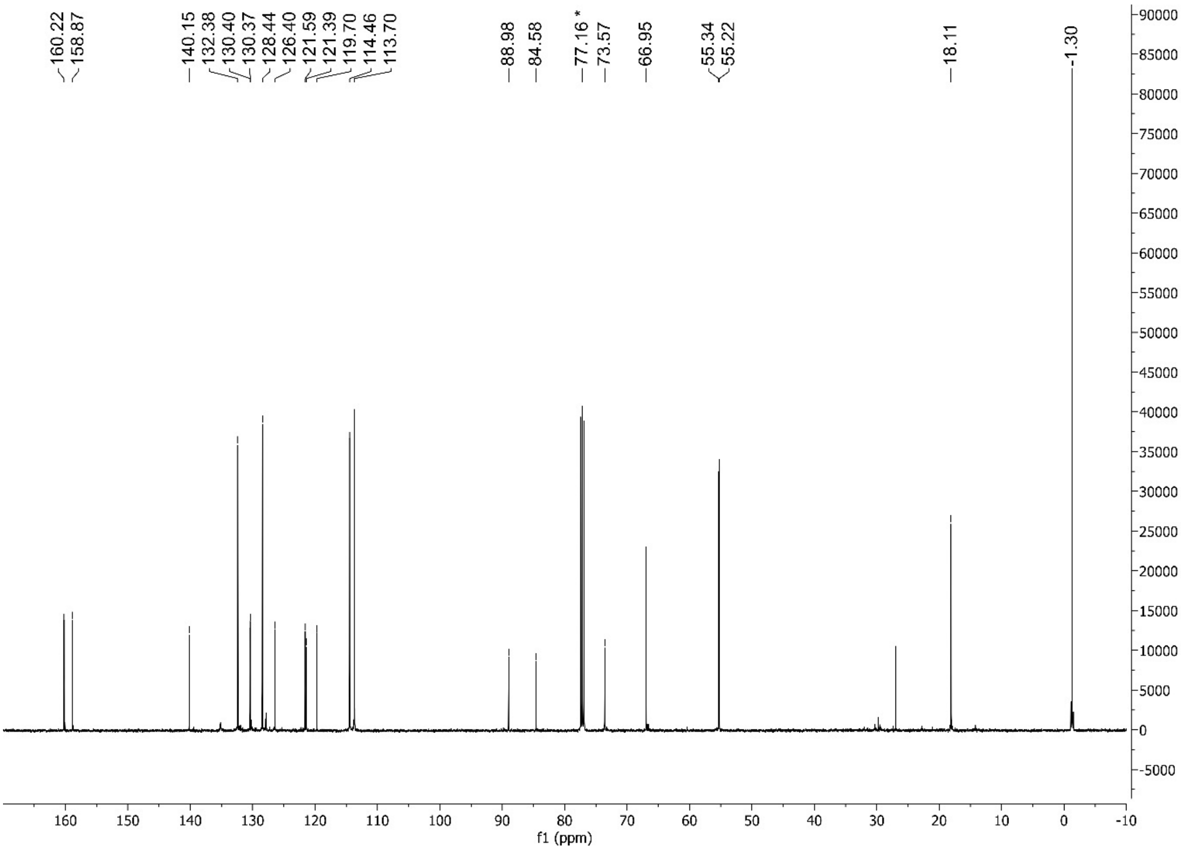

21b in $CDCl_3$, ^1H NMR (303 K, 500 MHz)

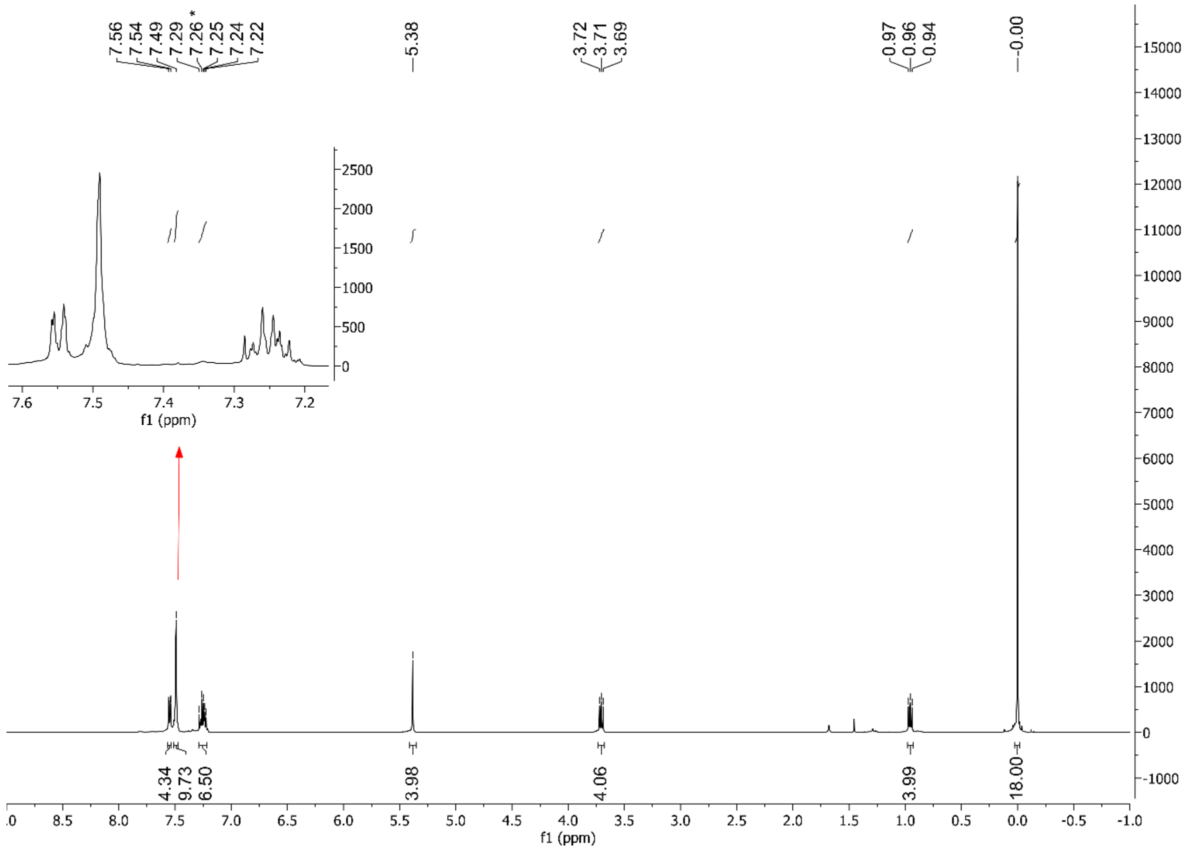

21b in $CDCl_3$, ^{13}C NMR (303 K, 125 MHz)

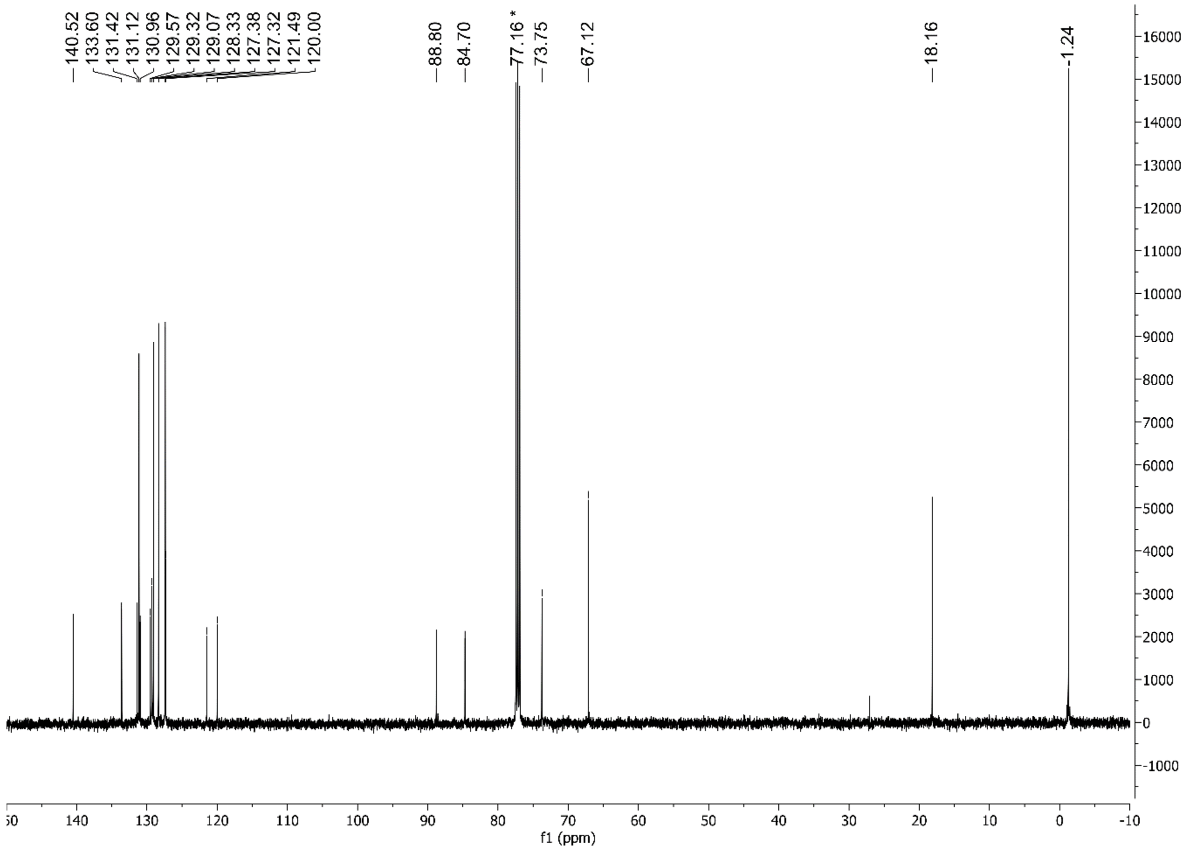

21c in $CDCl_3$, ^1H NMR (303 K, 500 MHz)

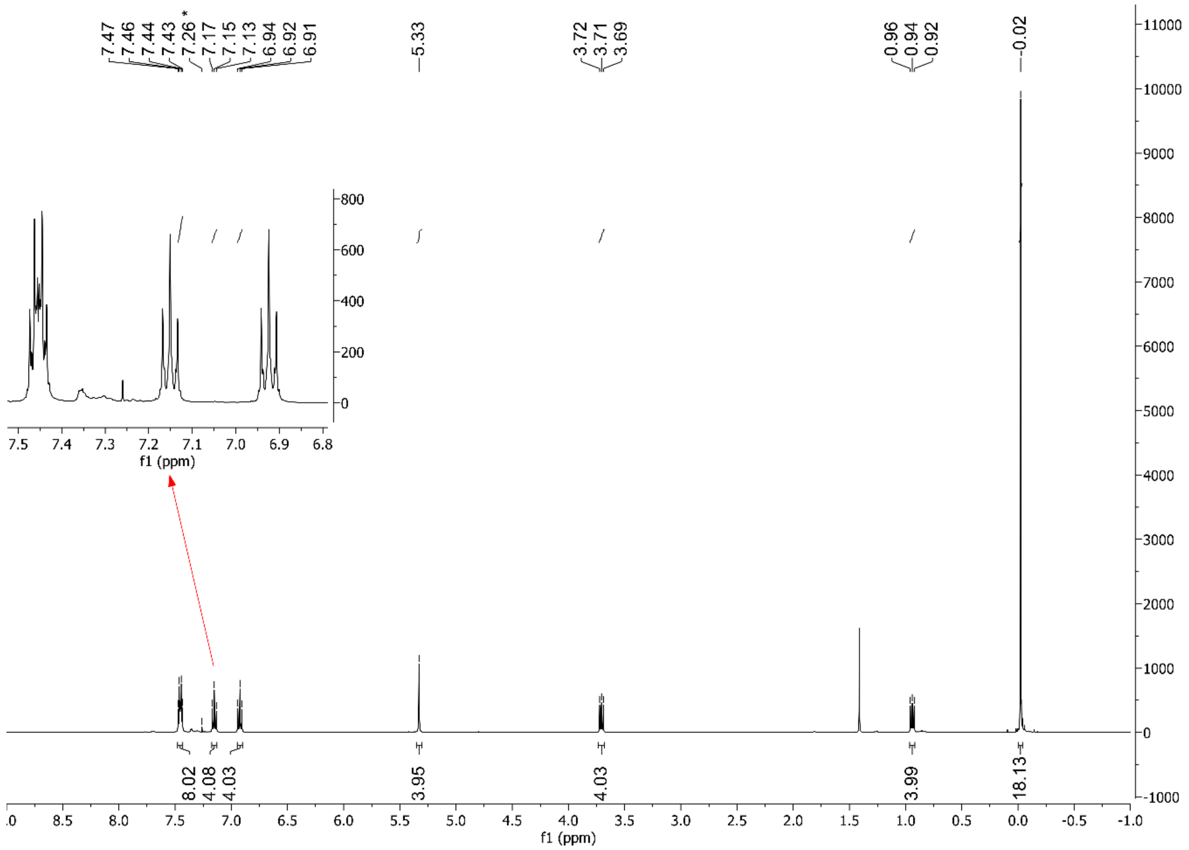

21c in $CDCl_3$, ^{13}C NMR (303 K, 125 MHz)

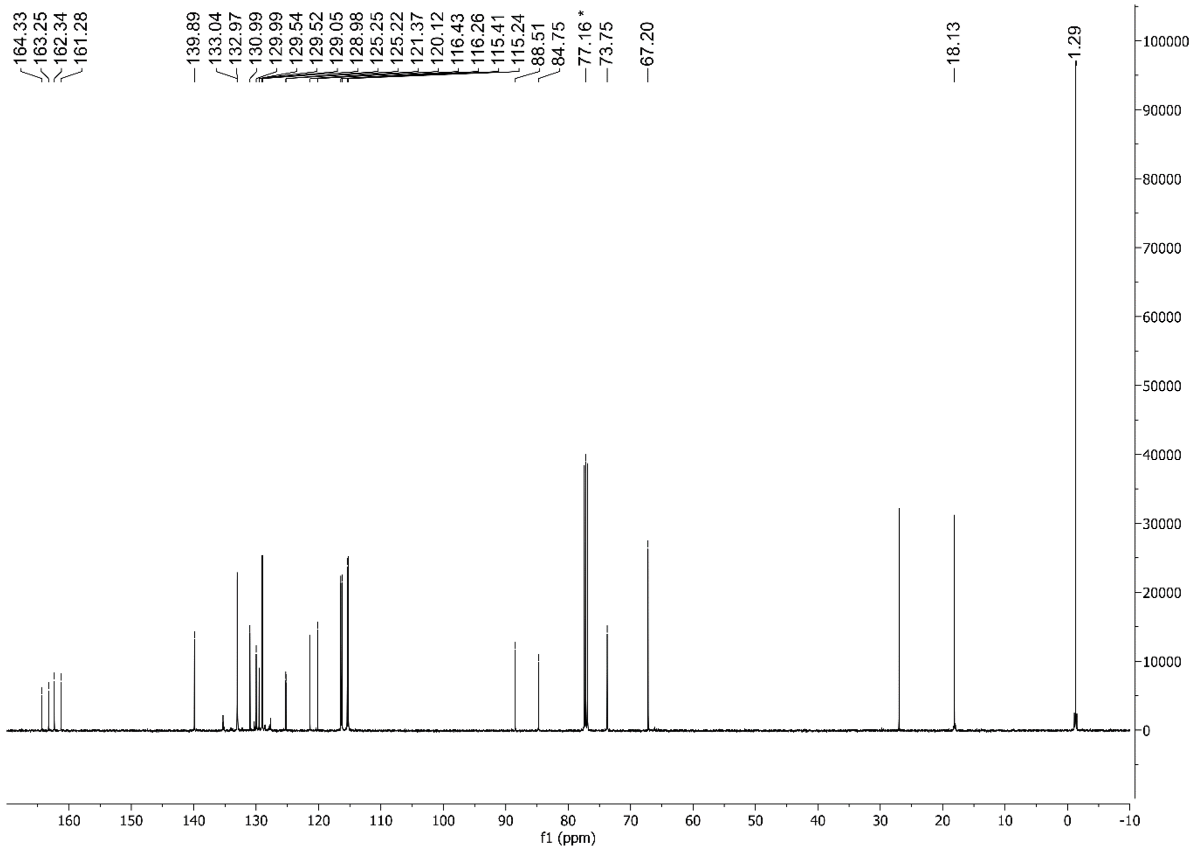

22a in CDCl3, ^{1}H NMR (303 K, 500 MHz)

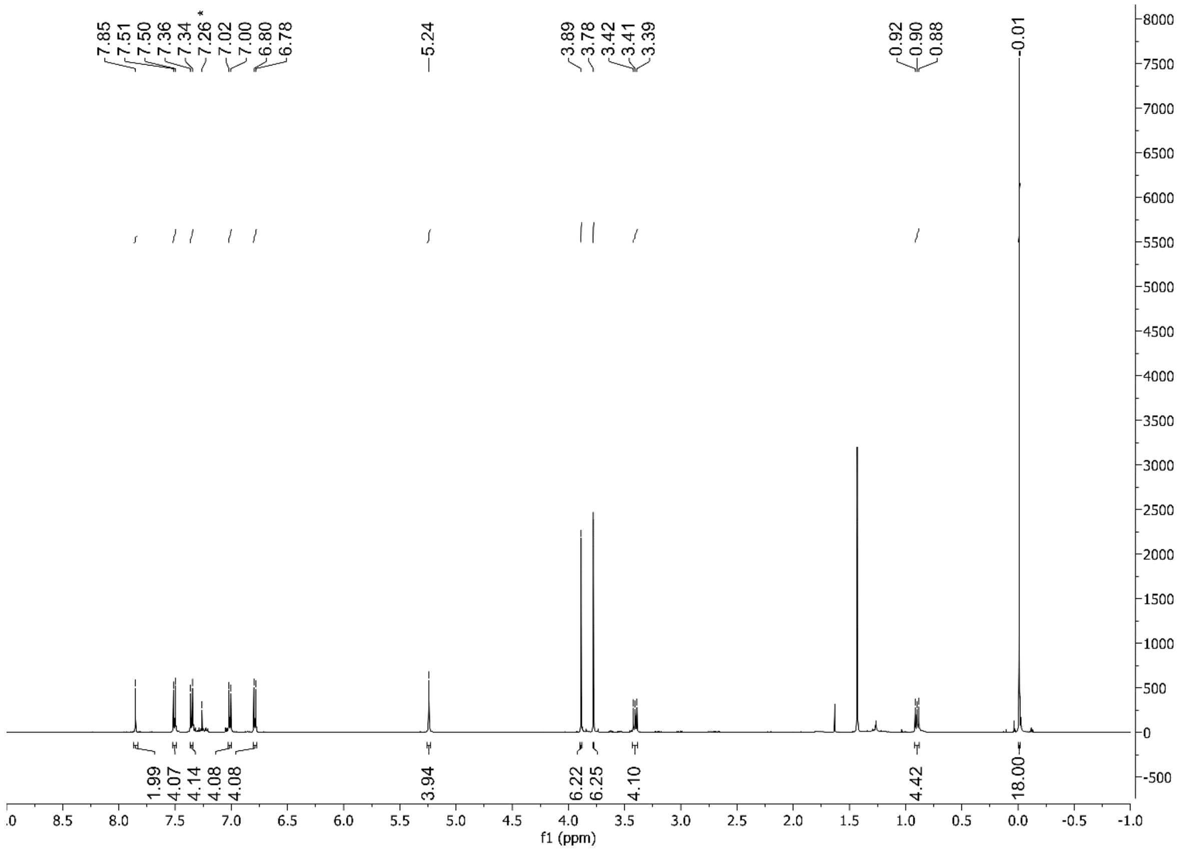

22a in CDCl3, ^{13}C NMR (303 K, 125 MHz)

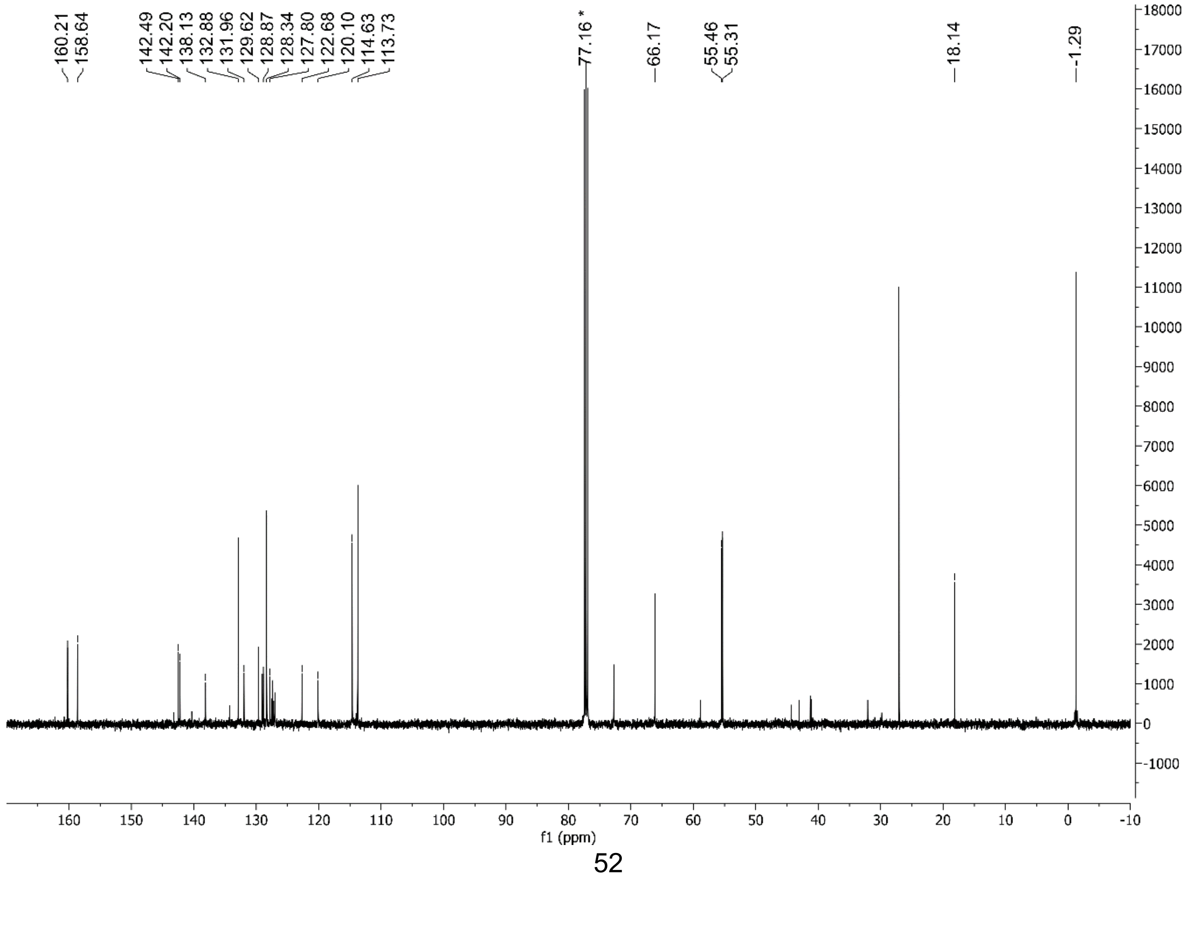

22b in CDCl3, ^{1}H NMR (303 K, 500 MHz)

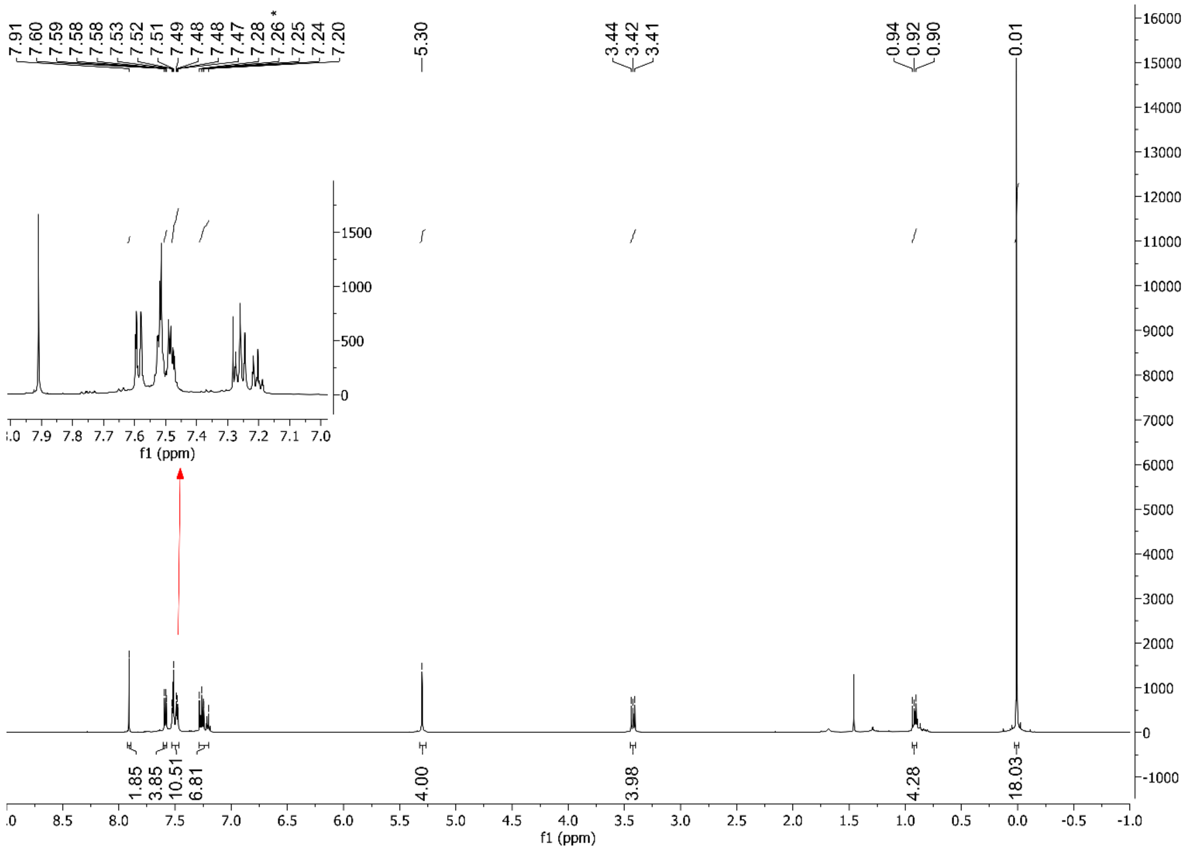

22b in CDCl3, ^{13}C NMR (303 K, 125 MHz)

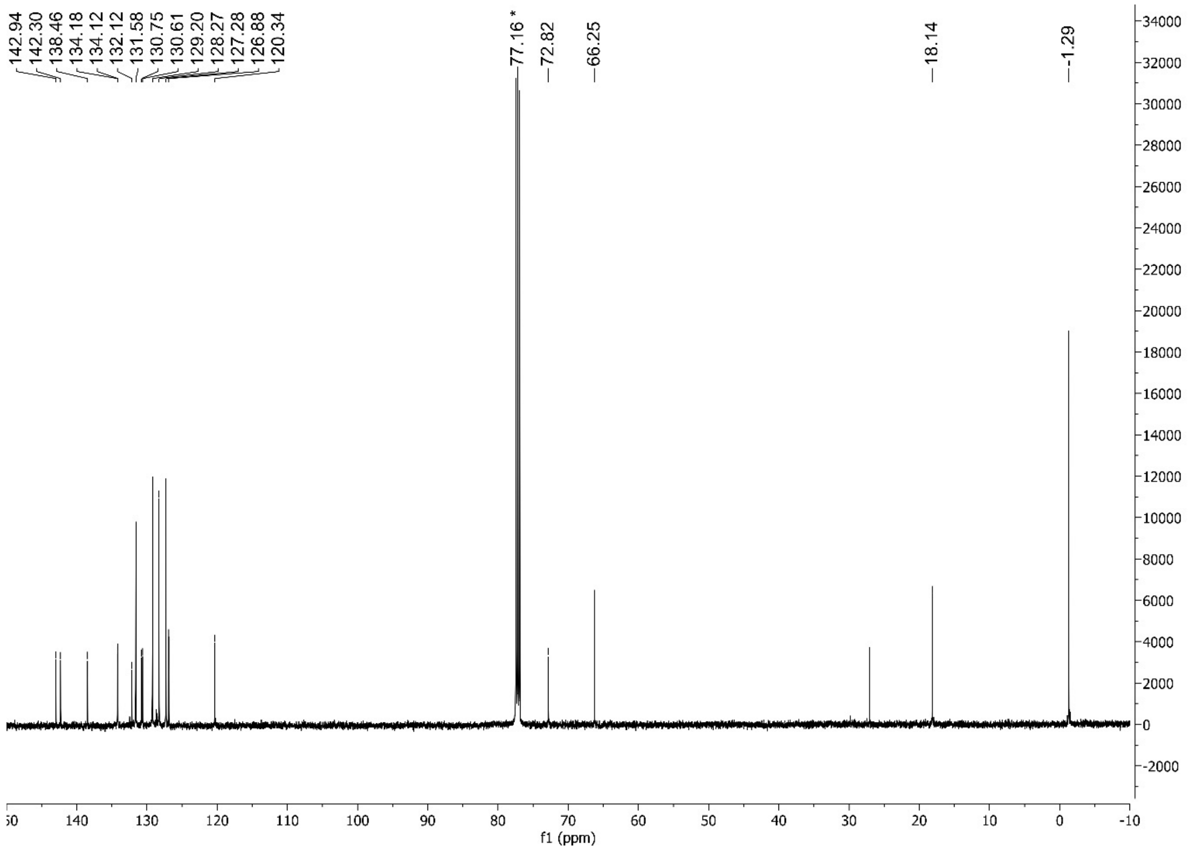

22c in $CDCl_3$, ^1H NMR (303 K, 500 MHz)

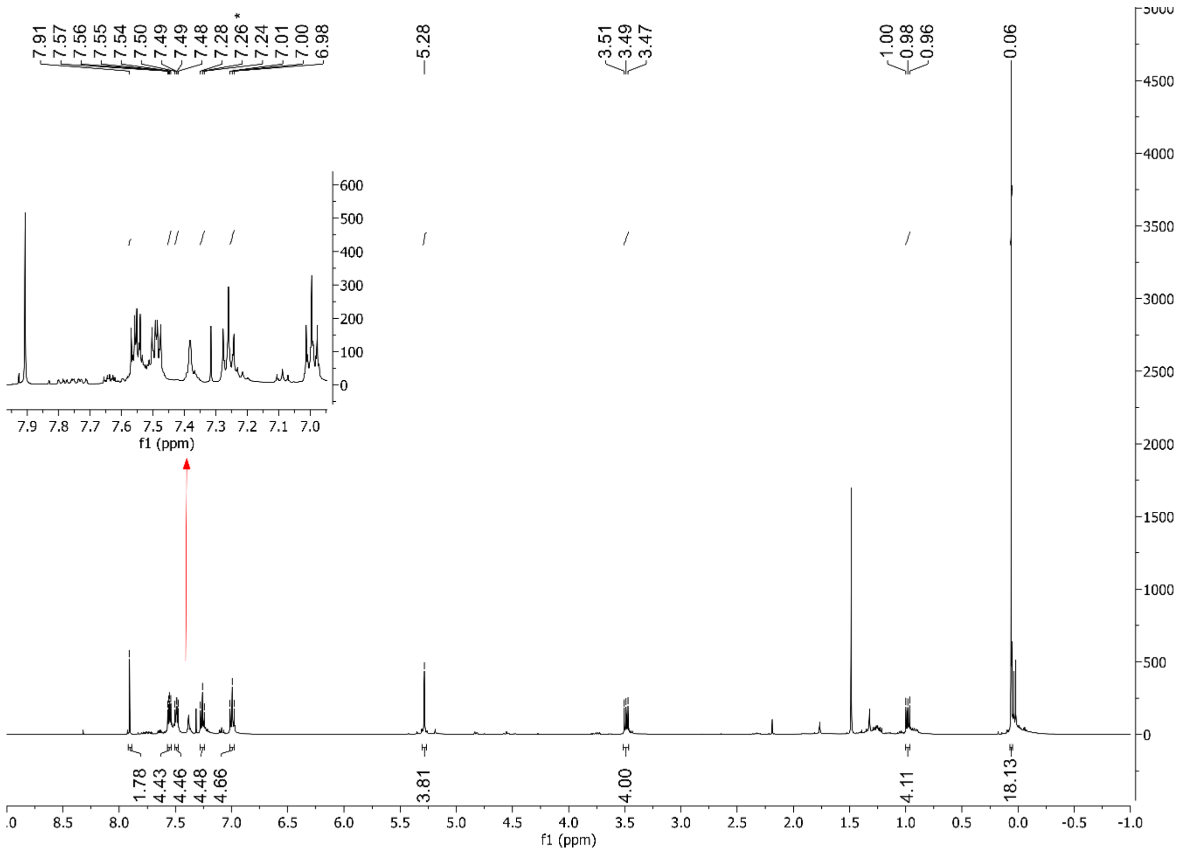

22c in $CDCl_3$, ^{13}C NMR (303 K, 125 MHz)

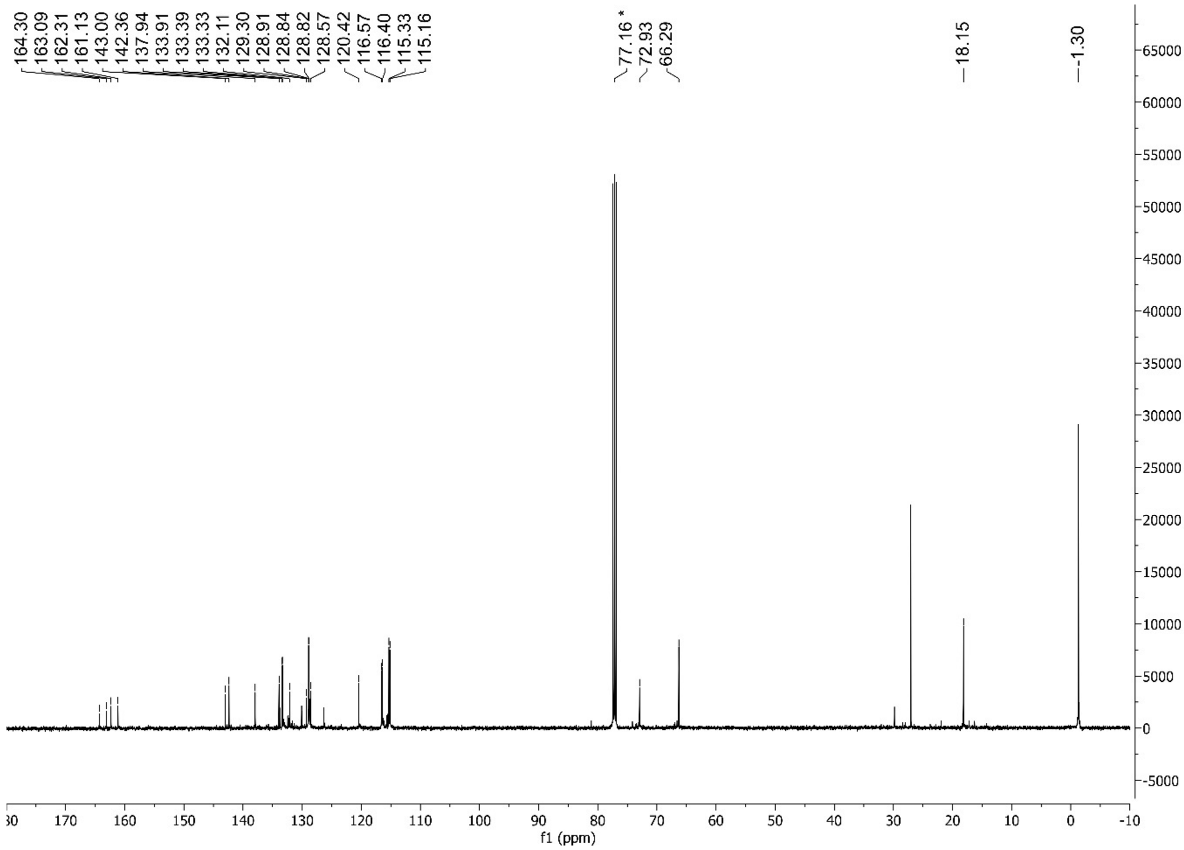

24 in $CDCl_3$, ^1H NMR (298 K, 700 MHz)

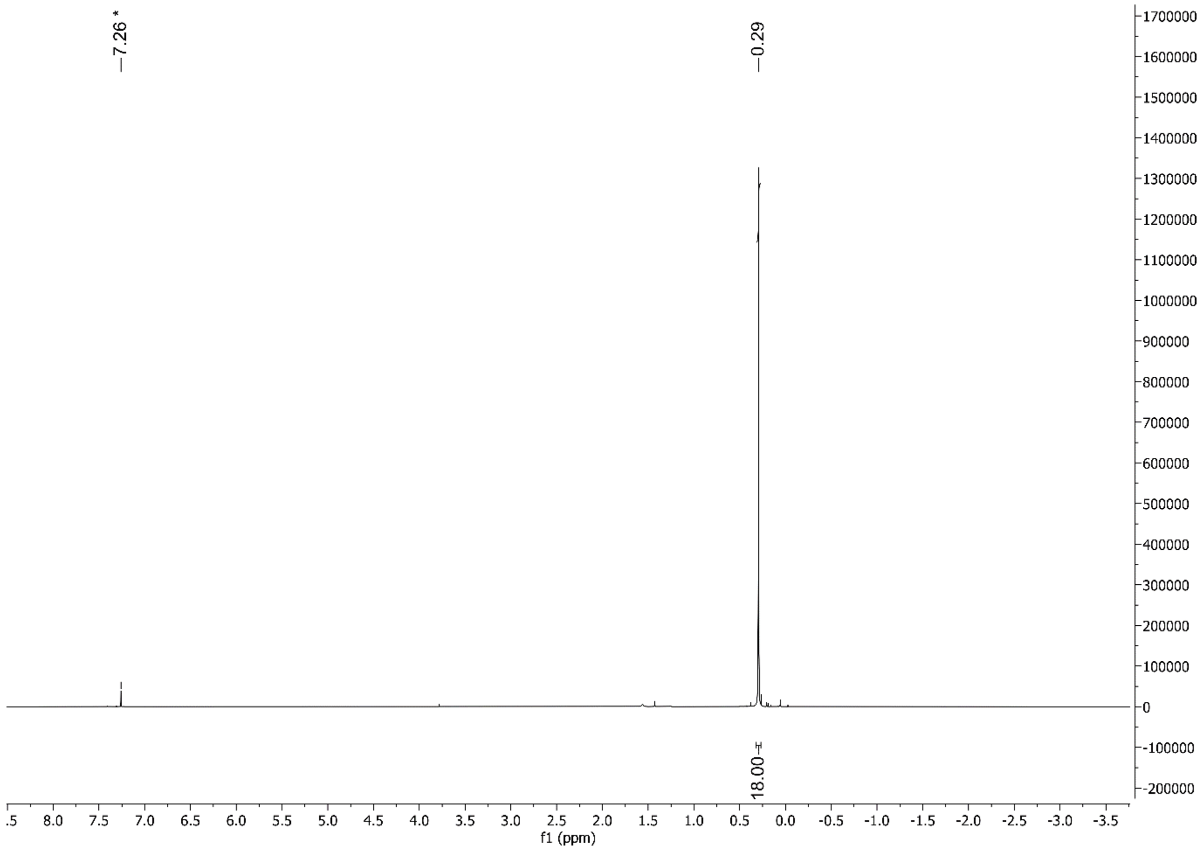

24 in $CDCl_3$, ^{13}C NMR (298 K, 175 MHz)

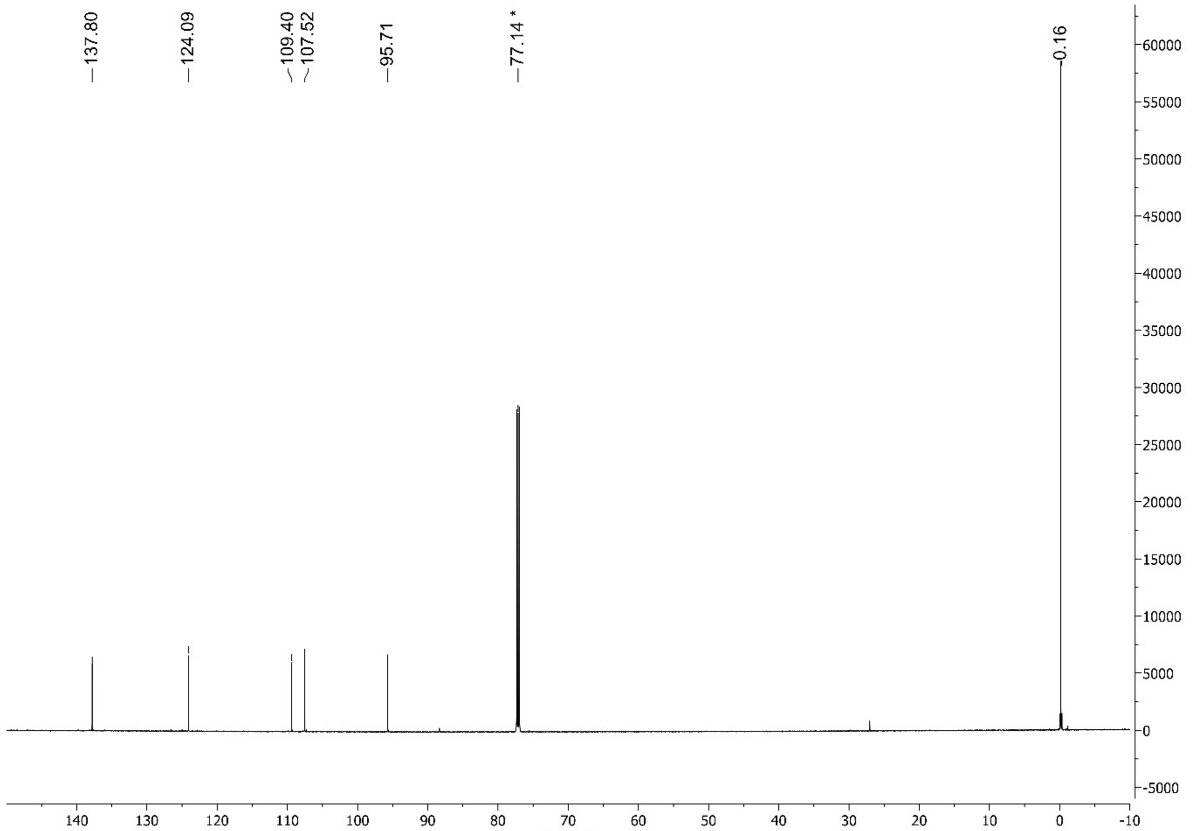

23 in CDCl3, ^{1}H NMR (298 K, 700 MHz)

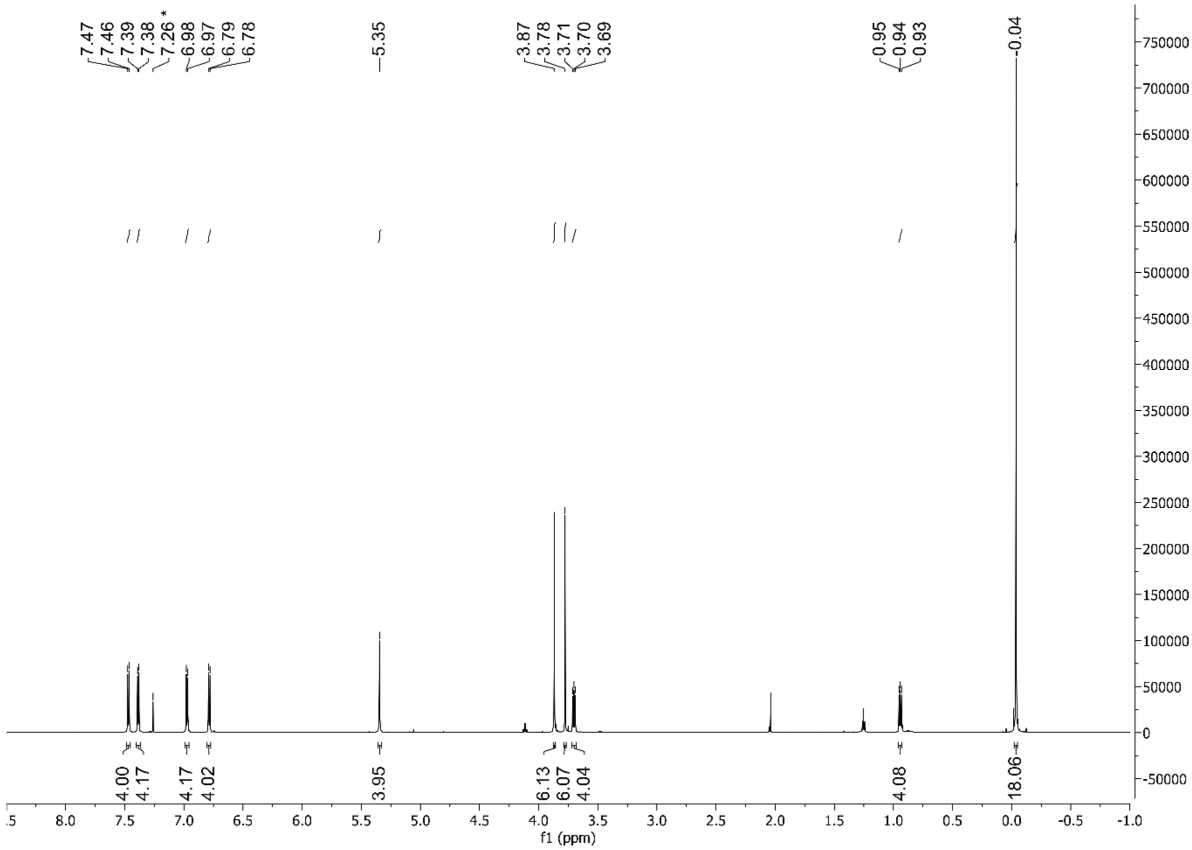

23 in CDCl3, ^{13}C NMR (298 K, 175 MHz)

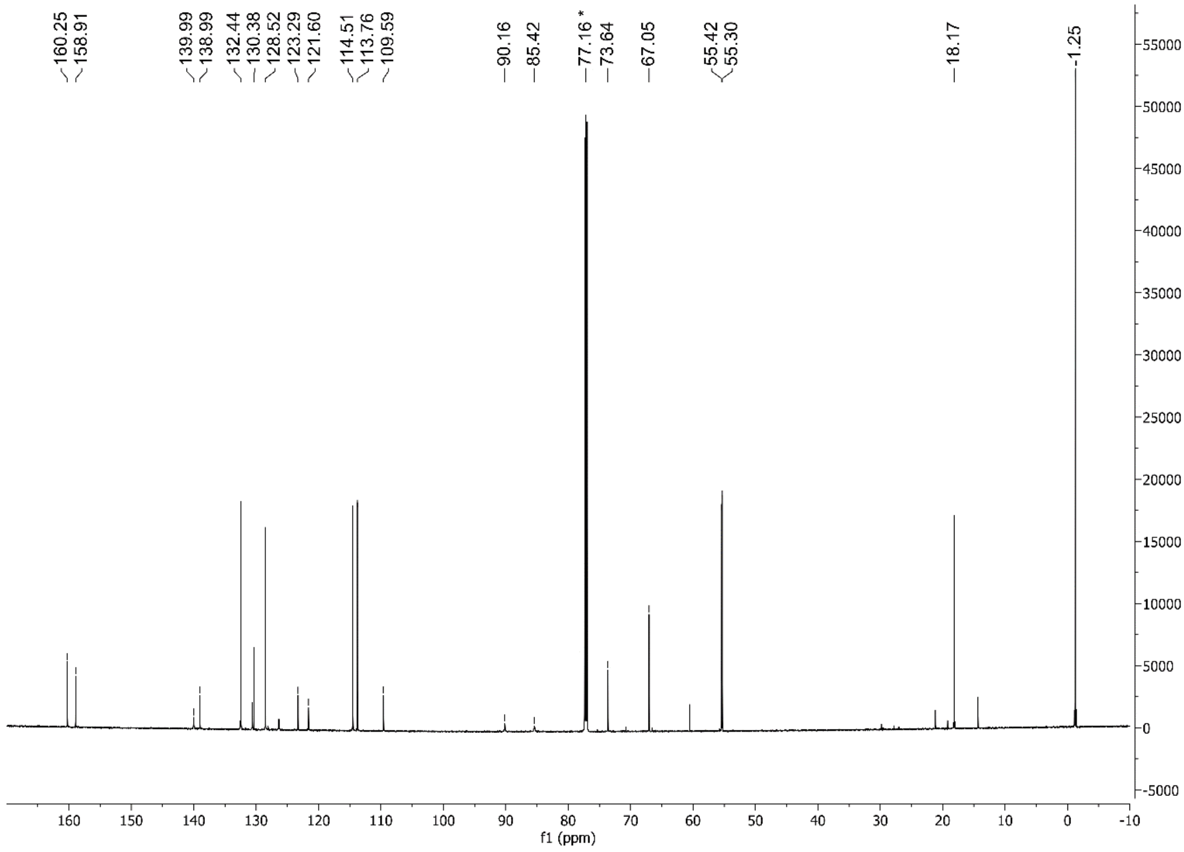

^1H or (and) ^{13}C NMR spectra of **14**, **16**, **S4a-c** and **25** cannot be recorded due to the poor solubility. Both ^1H and ^{13}C NMR of **8a-c** and **9** cannot be obtained due to the paramagnetic feature of the quinoids (biradical).

3.4. Mass spectra

12 MS (ESI) *m/z*: calc. $[C_{40}H_{35}N_4O_4]^+$ = 635.2653, found: 635.2668

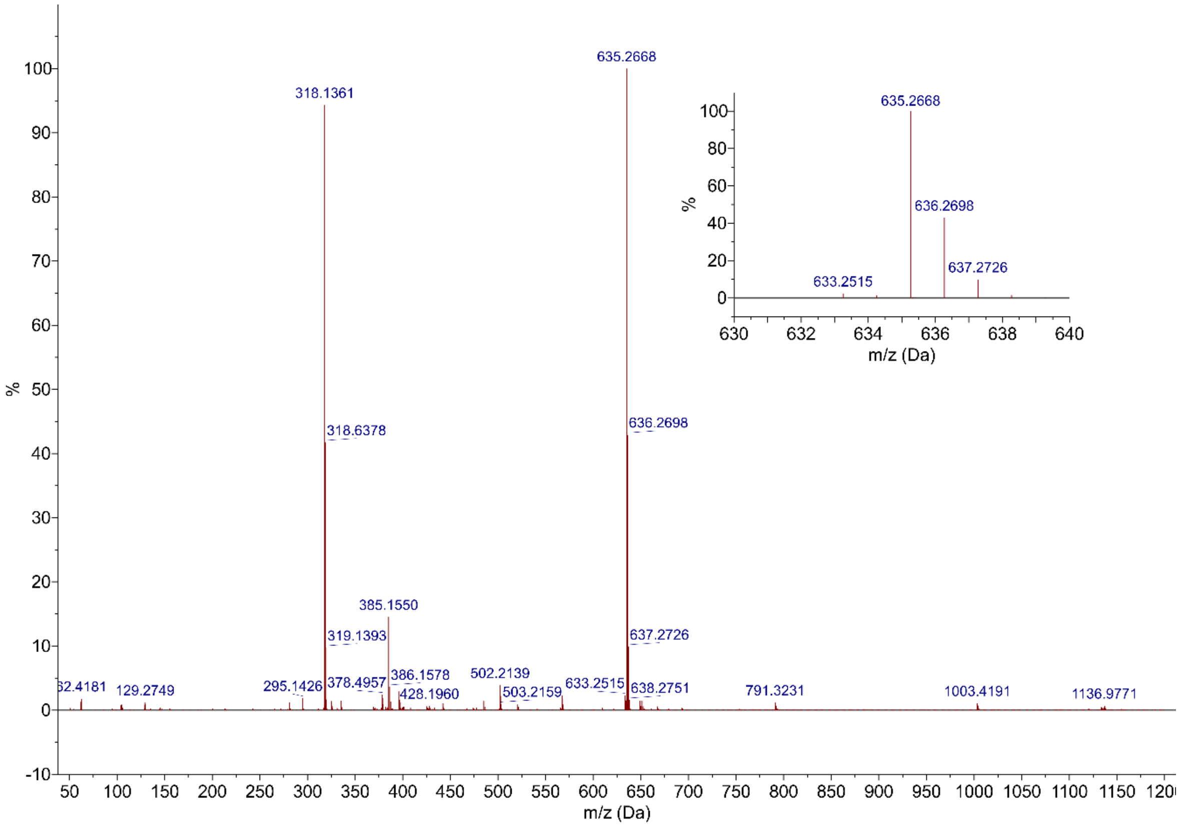

4 MS (ESI) *m/z*: calc. $[C_{38}H_{33}N_4O_4S]^+$ = 633.2497, found: 633.2504

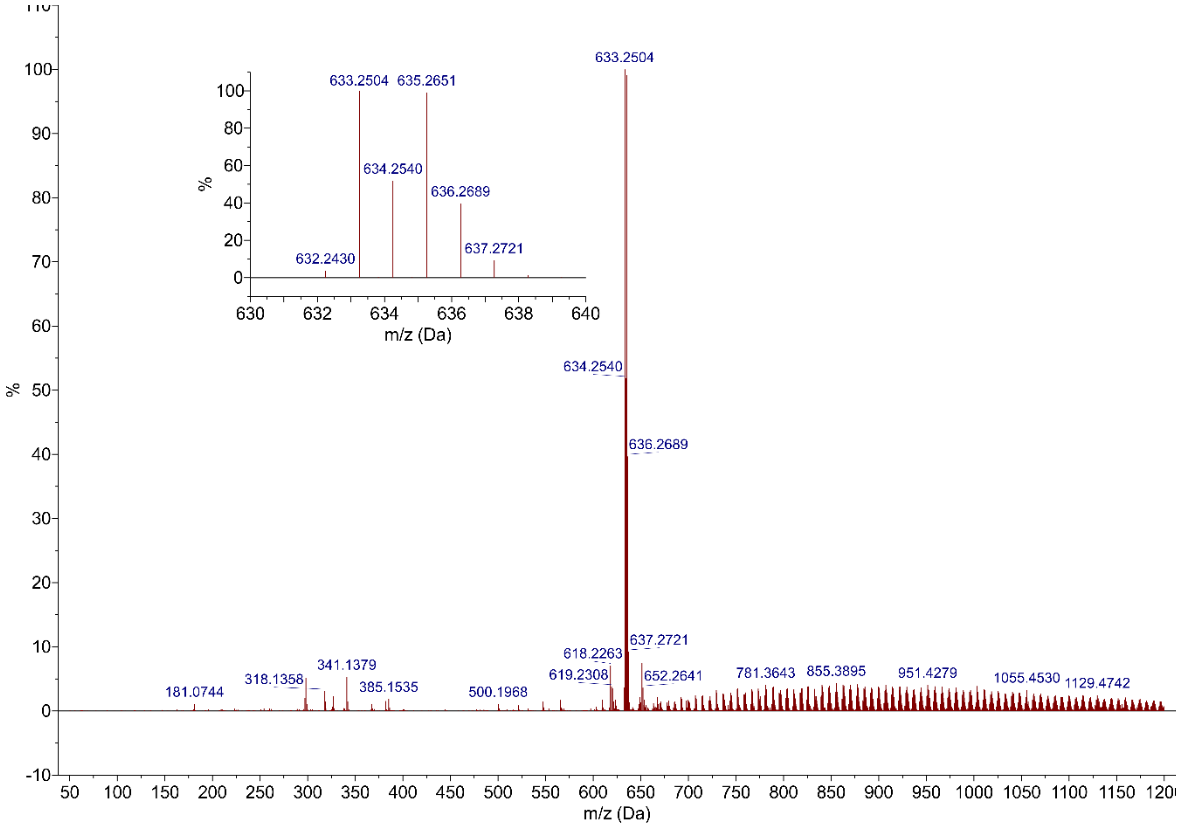

13 MS (EI) *m/z*: calc. [$C_6H_4O_2S$] = 139.9932, found: 139.9953.

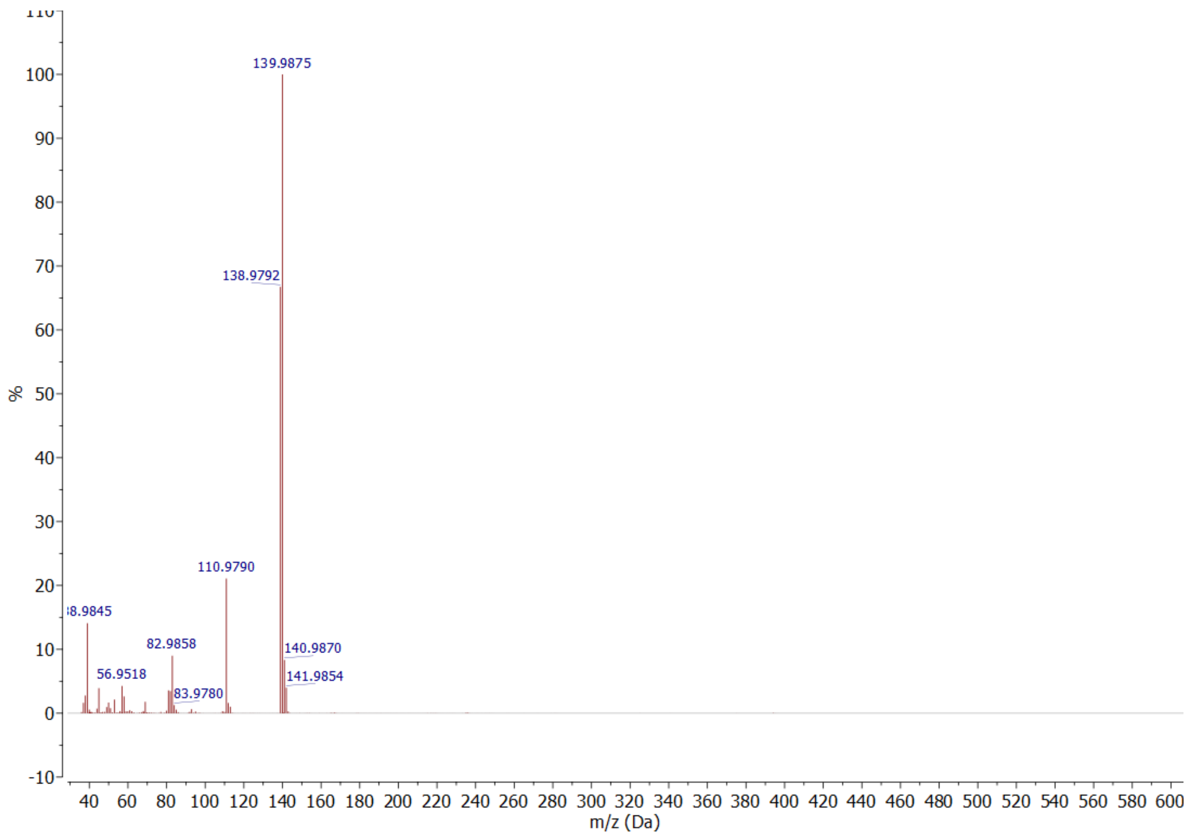

14 MS (ESI) *m/z*: calc. [$C_{38}H_{33}N_4O_4S$]$^+$ = 641.2218, found: 641.2267

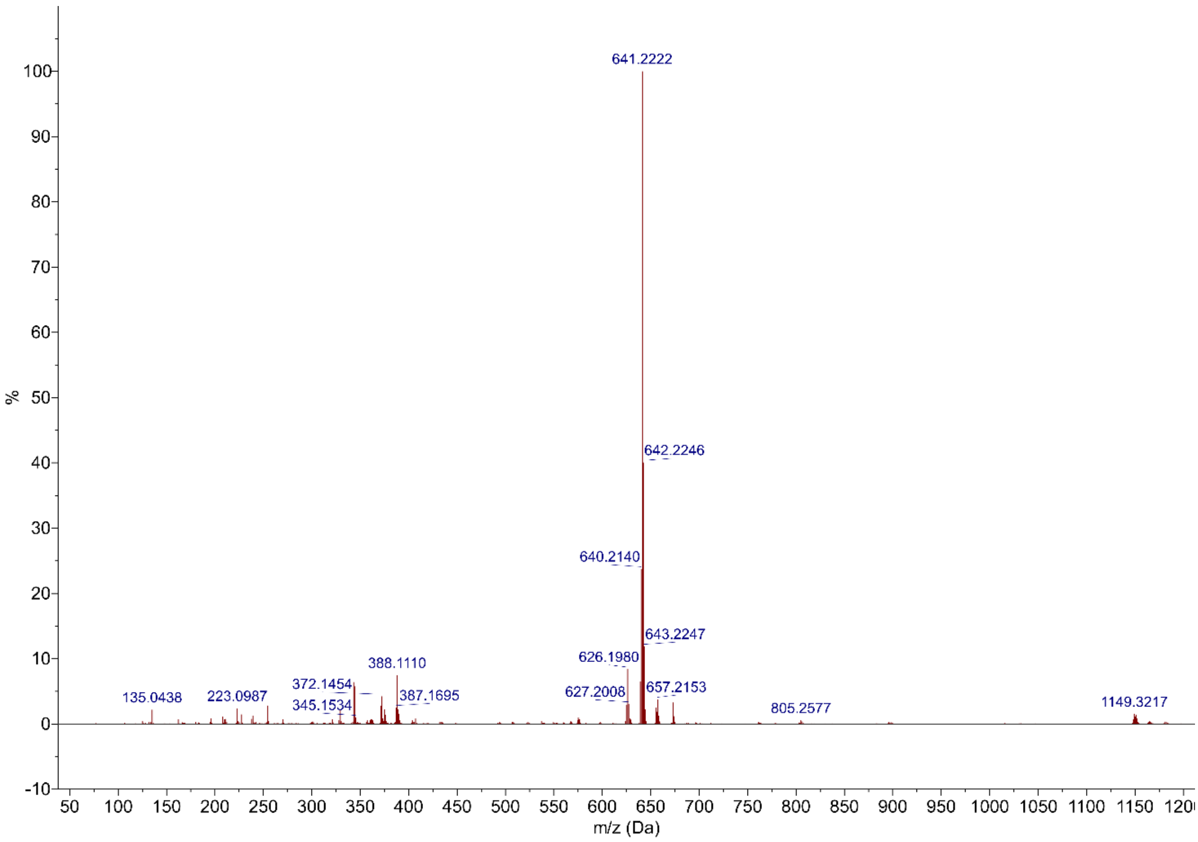

6 MS (ESI) *m/z*: calc. $[C_{38}H_{31}N_4O_4S]^+$ = 639.2061, found: 639.2205

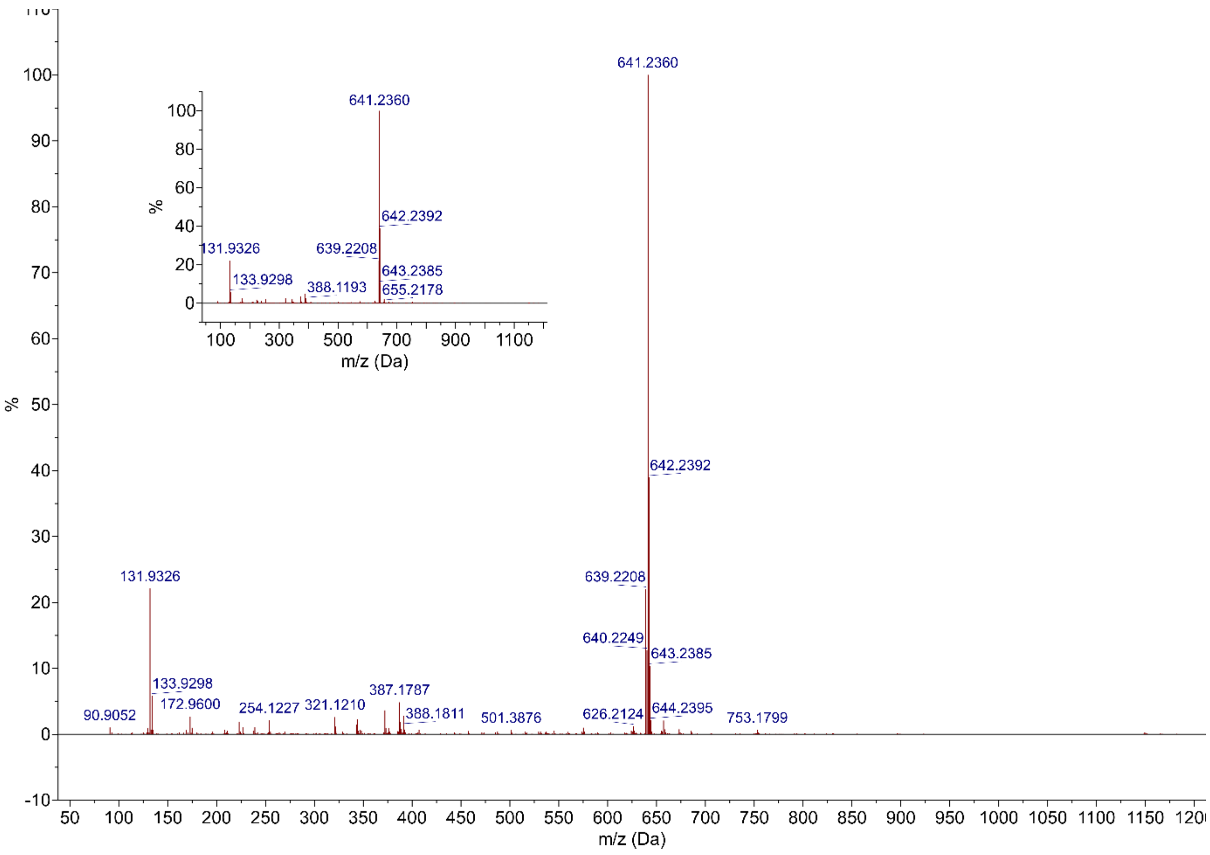

16 MS (ESI) *m/z*: calc. $[C_{40}H_{33}N_4O_4S_2]$ = 697.1943 $[M]^+$, found m/z: 697.1936

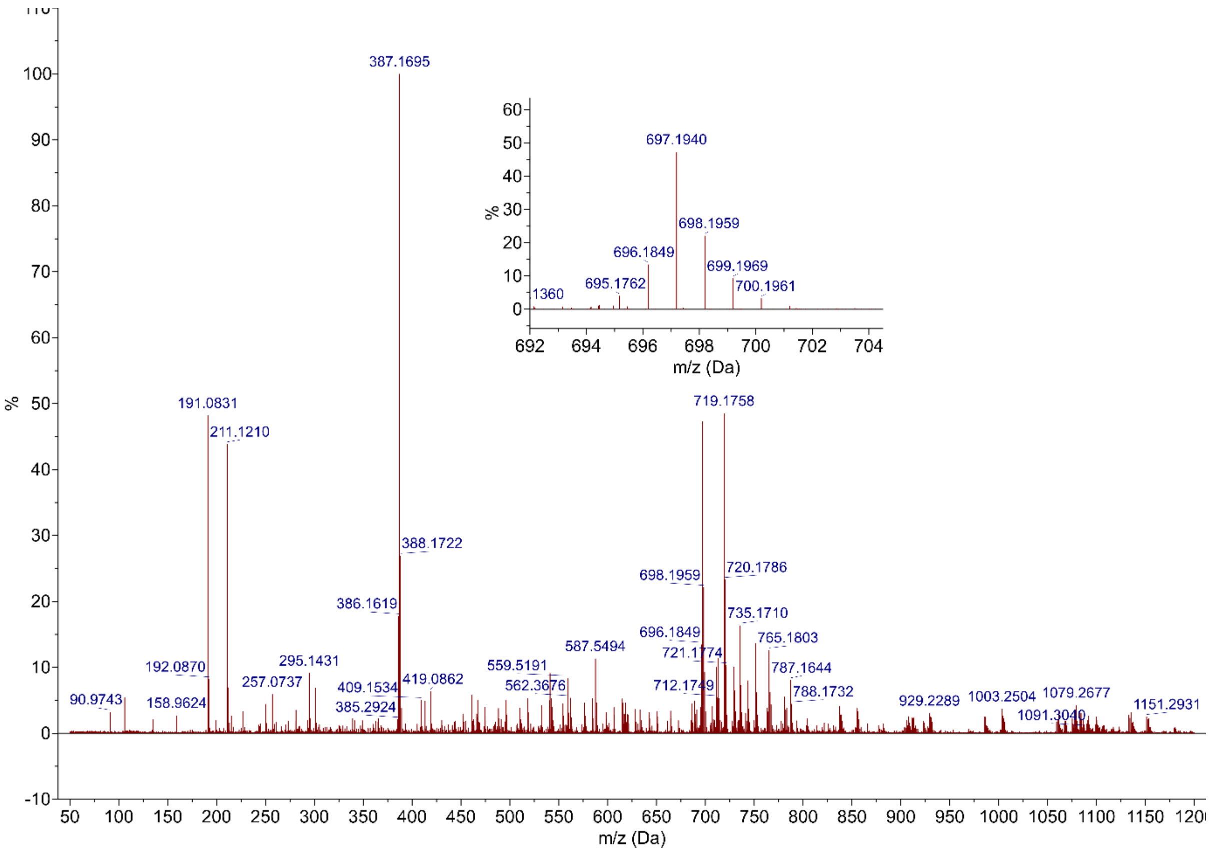

7 MS (ESI) *m/z*: calc. $C_{40}H_{30}N_4O_4S_2$: 695.1787 [M]$^+$, found m/z: 695.1774

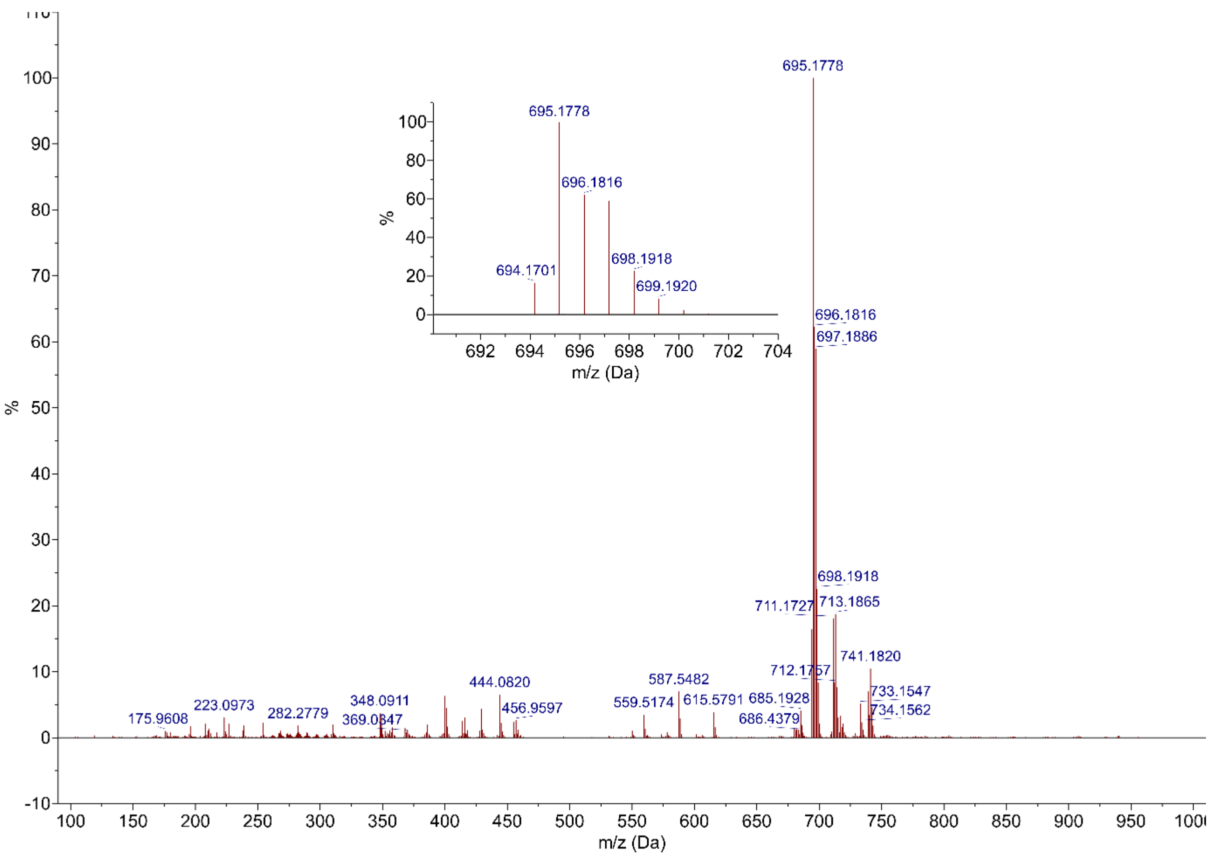

S1 MS (ESI) *m/z*: calc. $[C_{14}H_{10}F_2O_2Na]^+$ = 271.0547, found: 271.0541

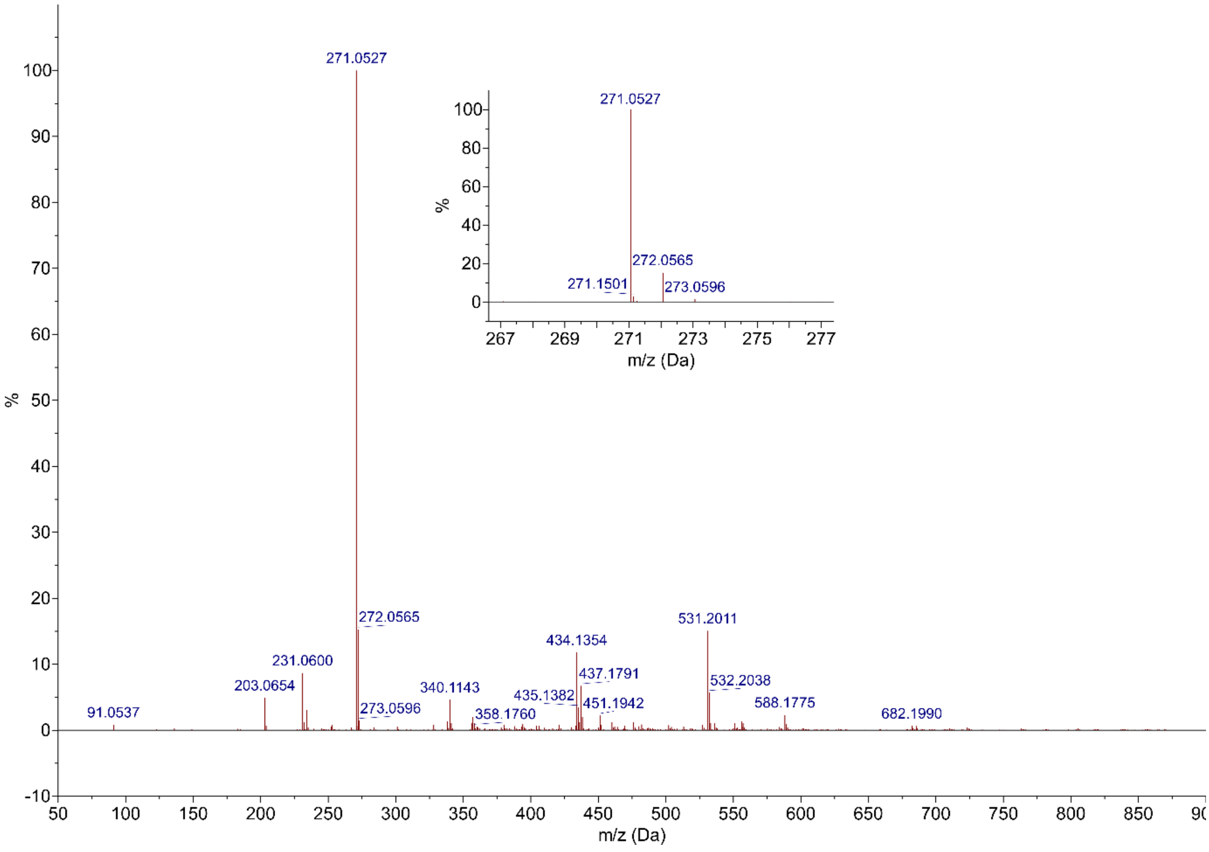

S2a MS (ESI) *m/z*: calc. $[C_{17}H_{17}N_2O_2]^+$ = 281.1290, found: 281.1300, calc. $[C_{17}H_{16}N_2O_2+Na]^+$ = 303.1109, found: 303.1117

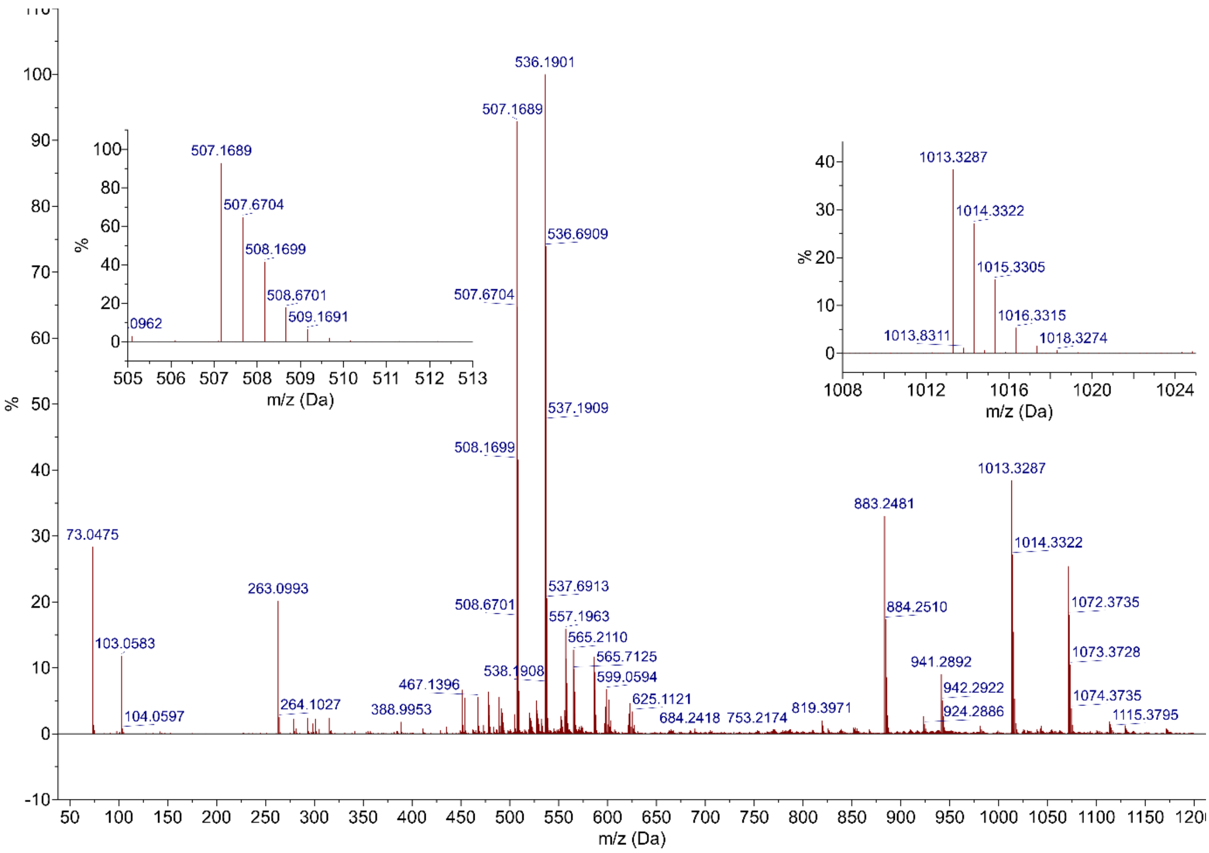

S2b MS (ESI) *m/z*: calc. $[C_{15}H_{11}F_2N_2]^+$ = 257.0890, found: 257.0886

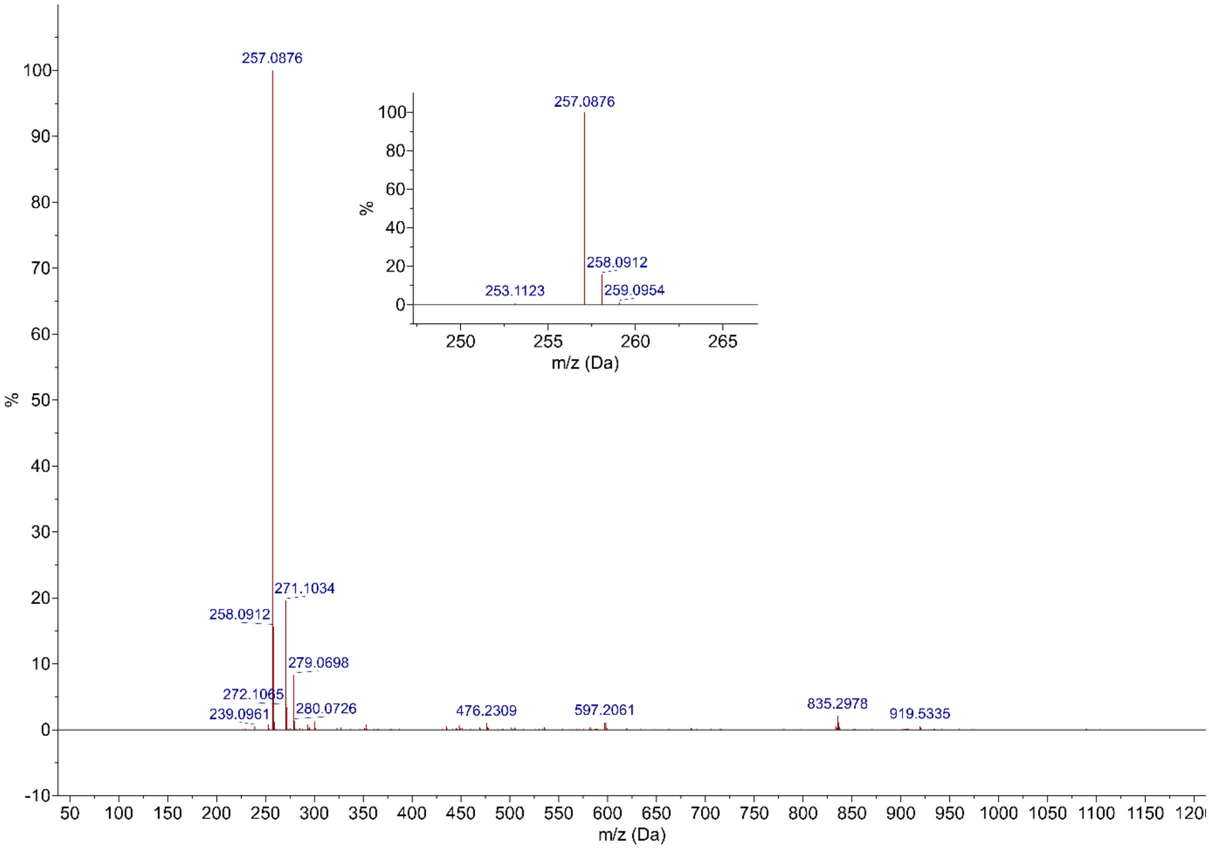

S3a MS (ESI) *m/z*: calc. $[C_{23}H_{31}N_2O_3Si]^+$ = 411.2104, found: 411.2128, calc. $[C_{23}H_{30}N_2O_3Si+Na]^+$ = 433.1923, found: 433.1924

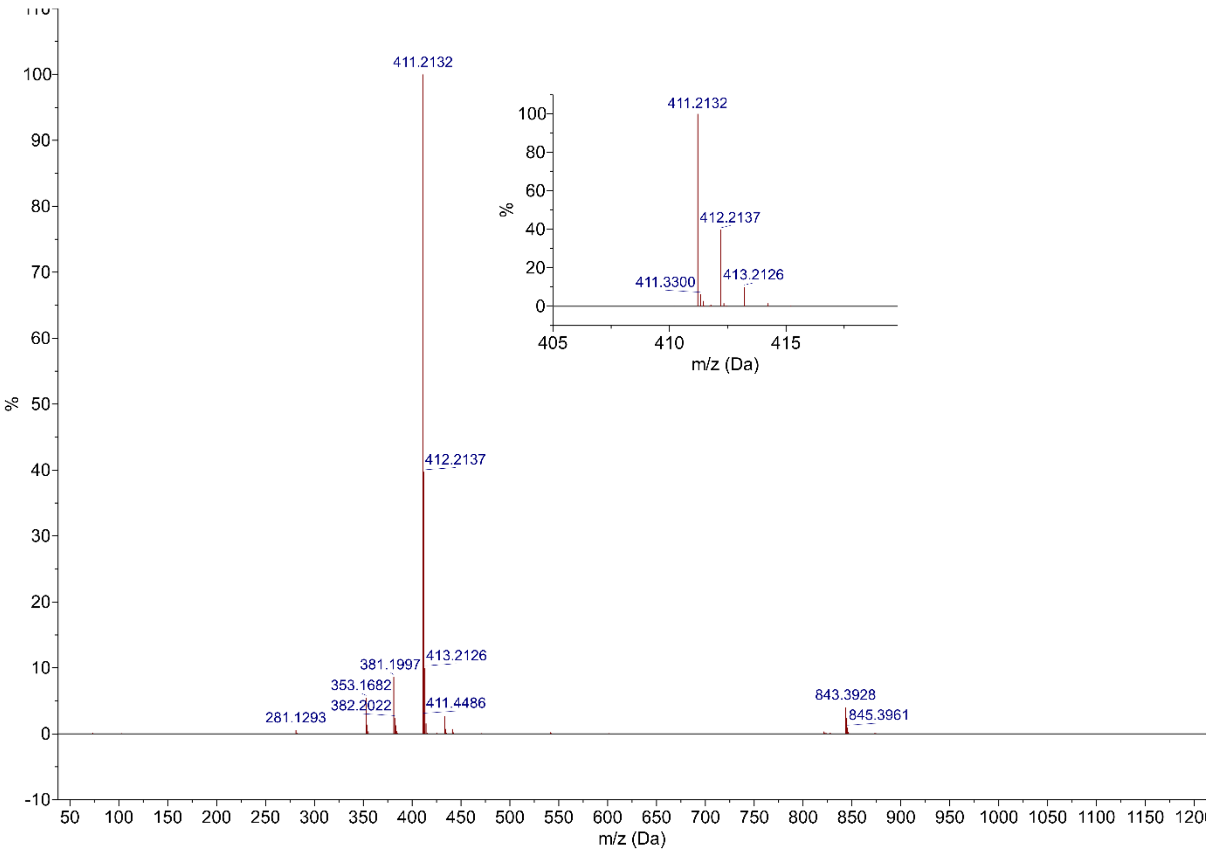

S3b MS (ESI) *m/z*: calc. $[C_{21}H_{27}N_2OSi]^+$ = 351,1893, found: 351.1895

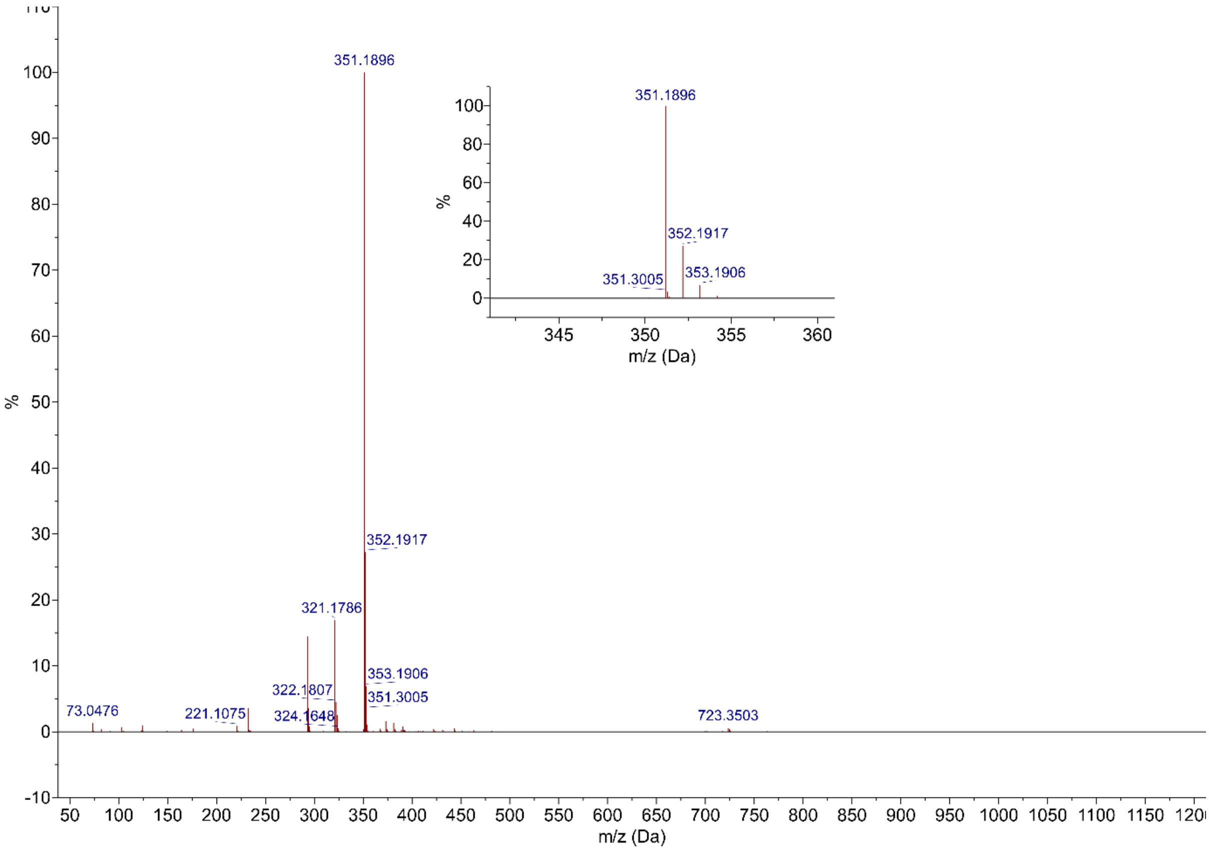

S3c MS (ESI) *m/z*: calc. $[C_{21}H_{25}N_2SiOF_2]^+$ = 387.1704, found: 387.1704

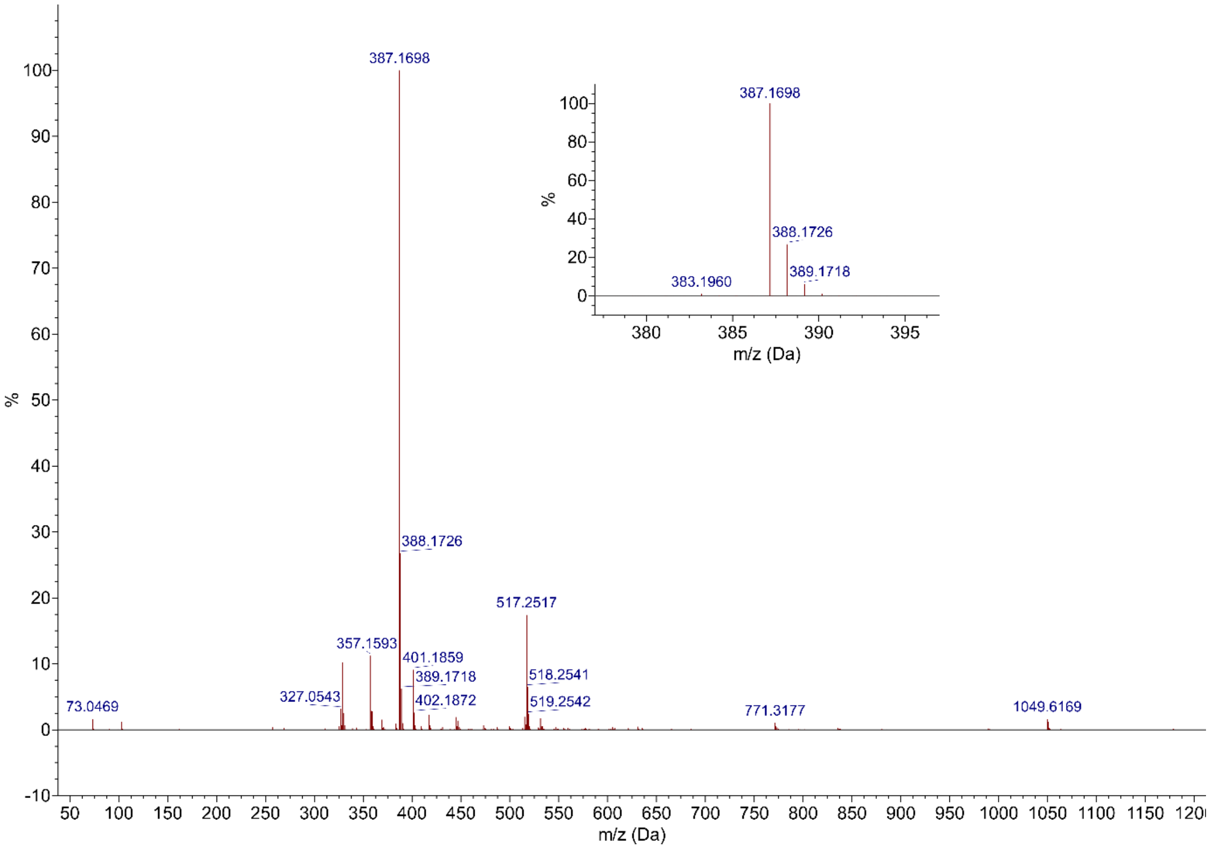

20a MS (ESI) *m/z*: calc. $[C_{23}H_{30}N_2O_3SiI]^+$ = 537.1070, found: 537.1051

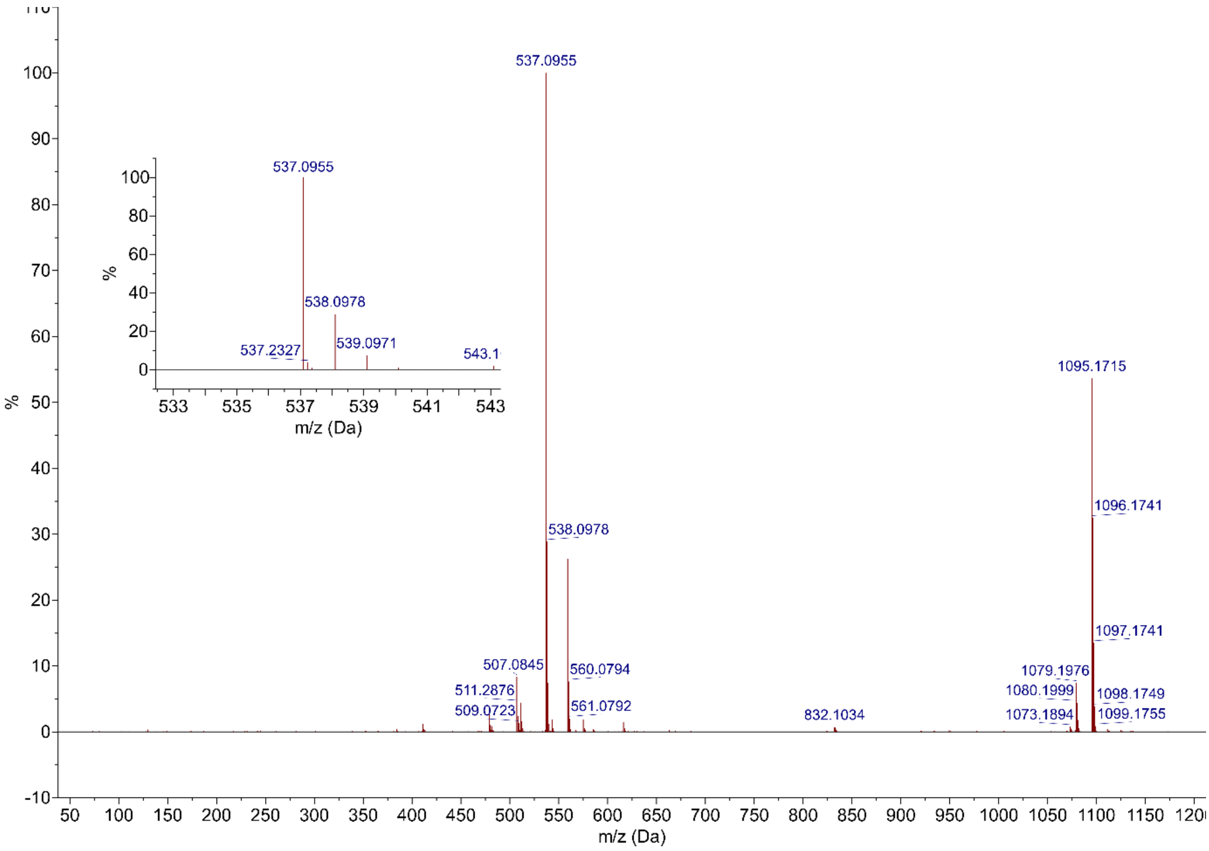

20b MS (ESI) *m/z*: calc. $[C_{21}H_{26}IN_2OSi]^+$ = 477.0859, found: 477.0874

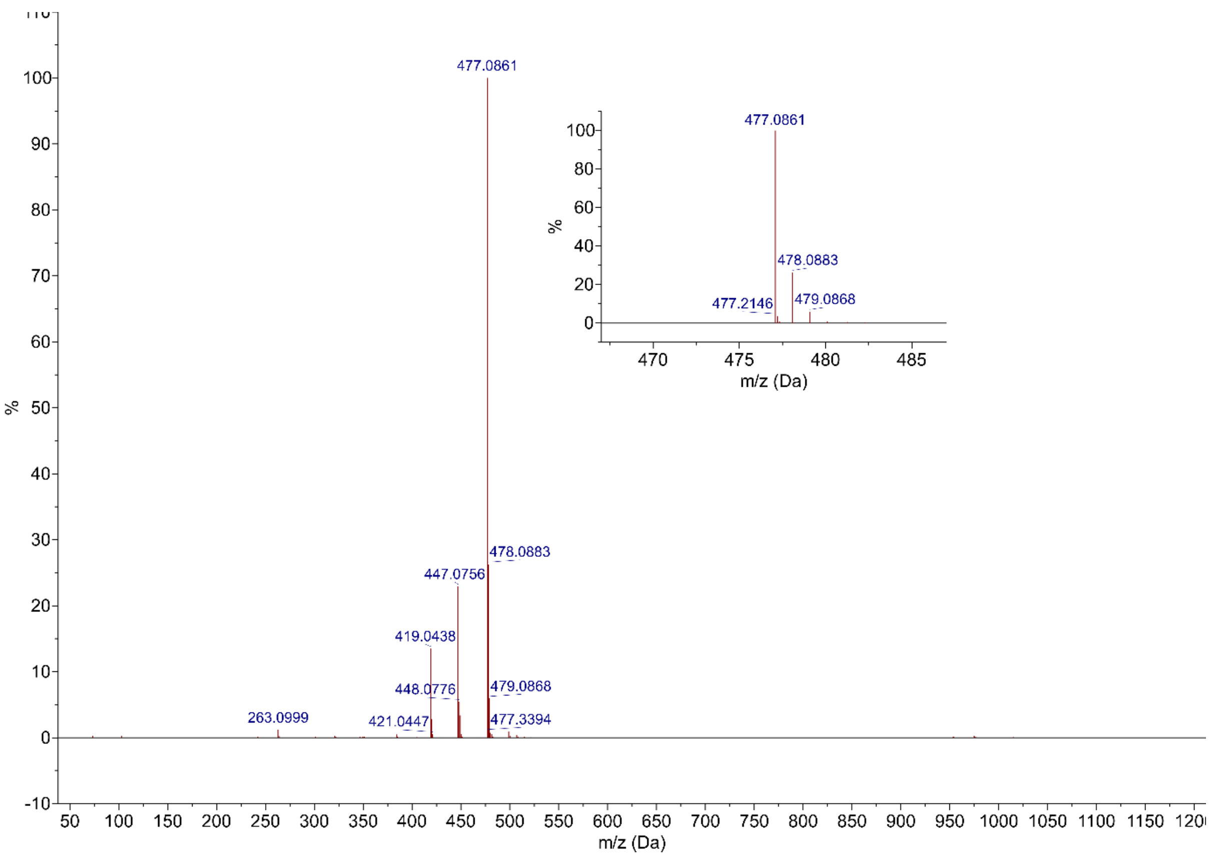

20c MS (ESI) *m/z*: calc. $[C_{21}H_{23}F_2IN_2OSi]^+$ = 513.0671, found: 513.0673

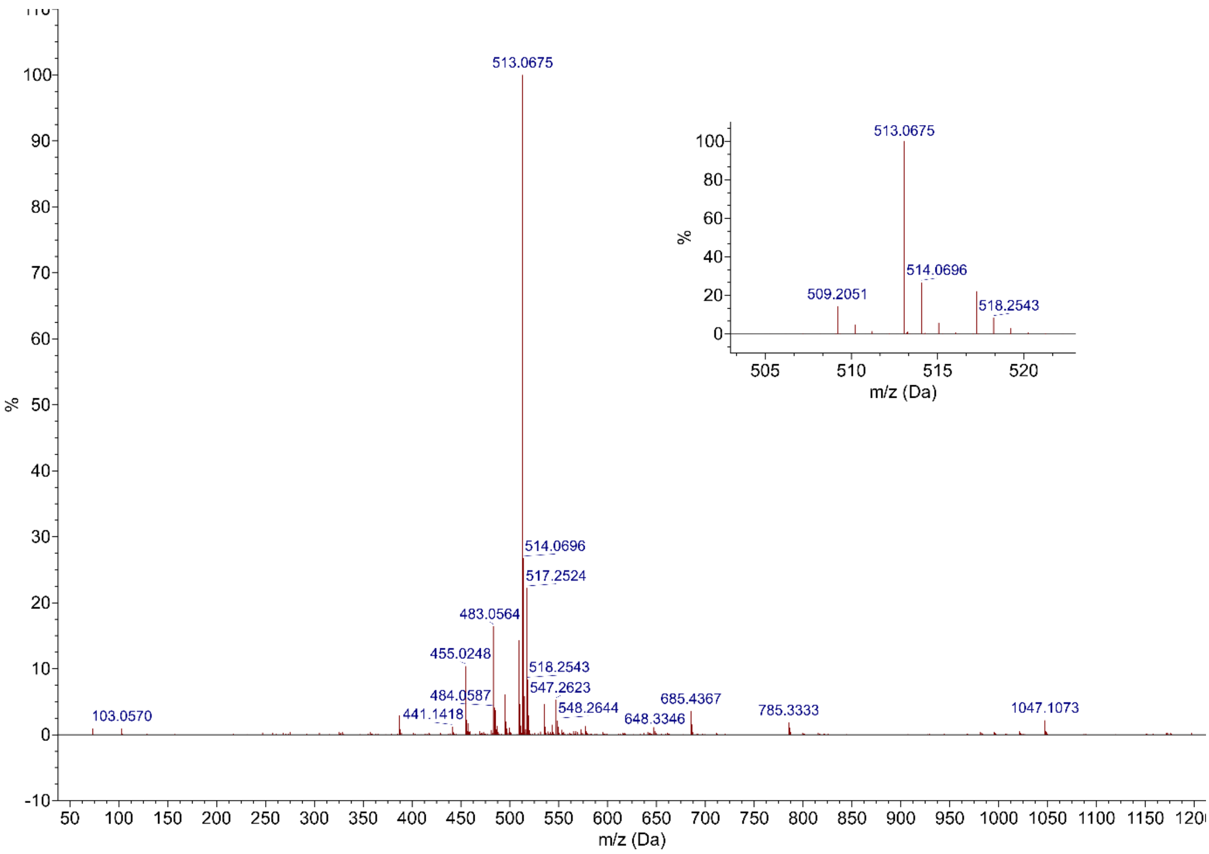

19 MS (EI) *m/z:* calc. [$C_{14}H_{18}Br_2Si_2S$] = 433.9014, found: 433.8999

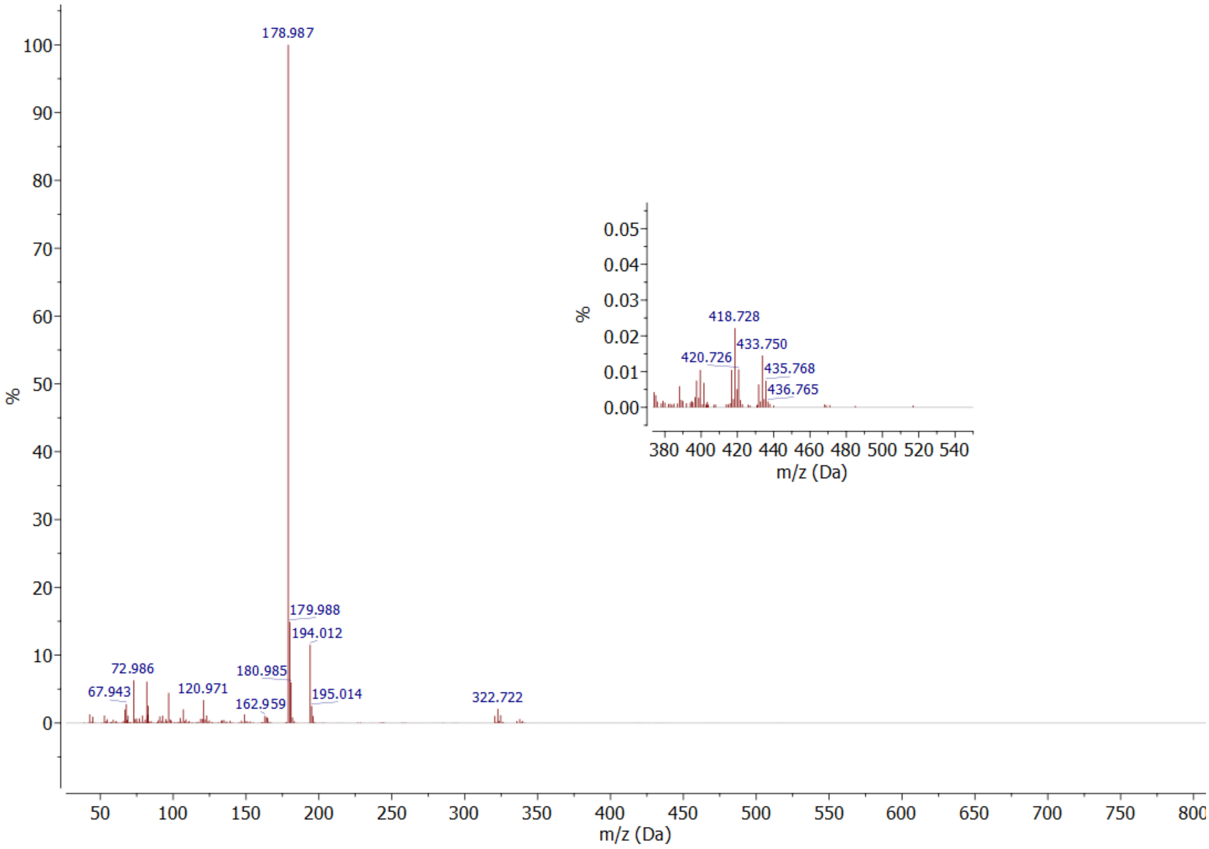

21a MS (ESI) *m/z*: calc. [$C_{54}H_{59}N_4O_6Si_2Br_2S]^+$ = 1107.2035, found: 1107.1840

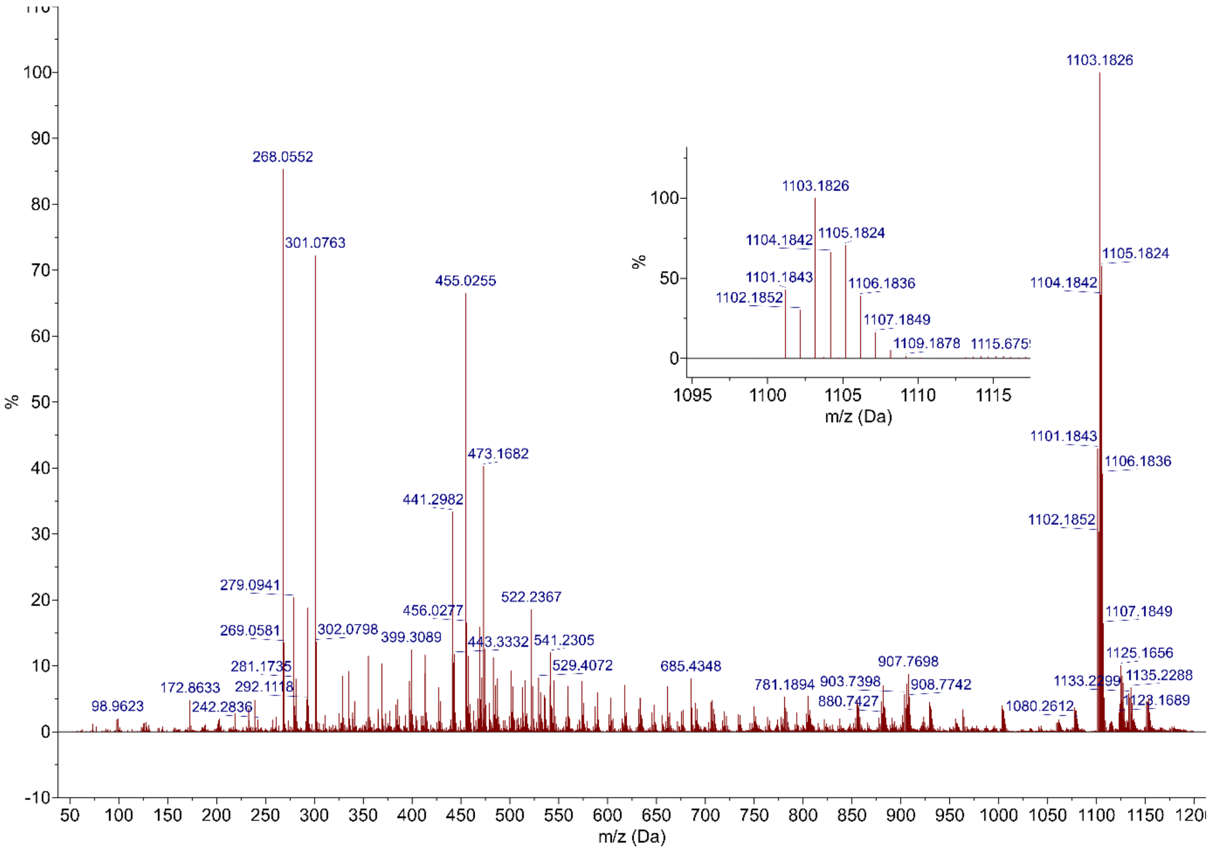

21b MS (ESI) *m/z*: calc. $[C_{50}H_{51}Br_2N_4O_2SSi_2]^+$ = 987.1618, found: 987.1635

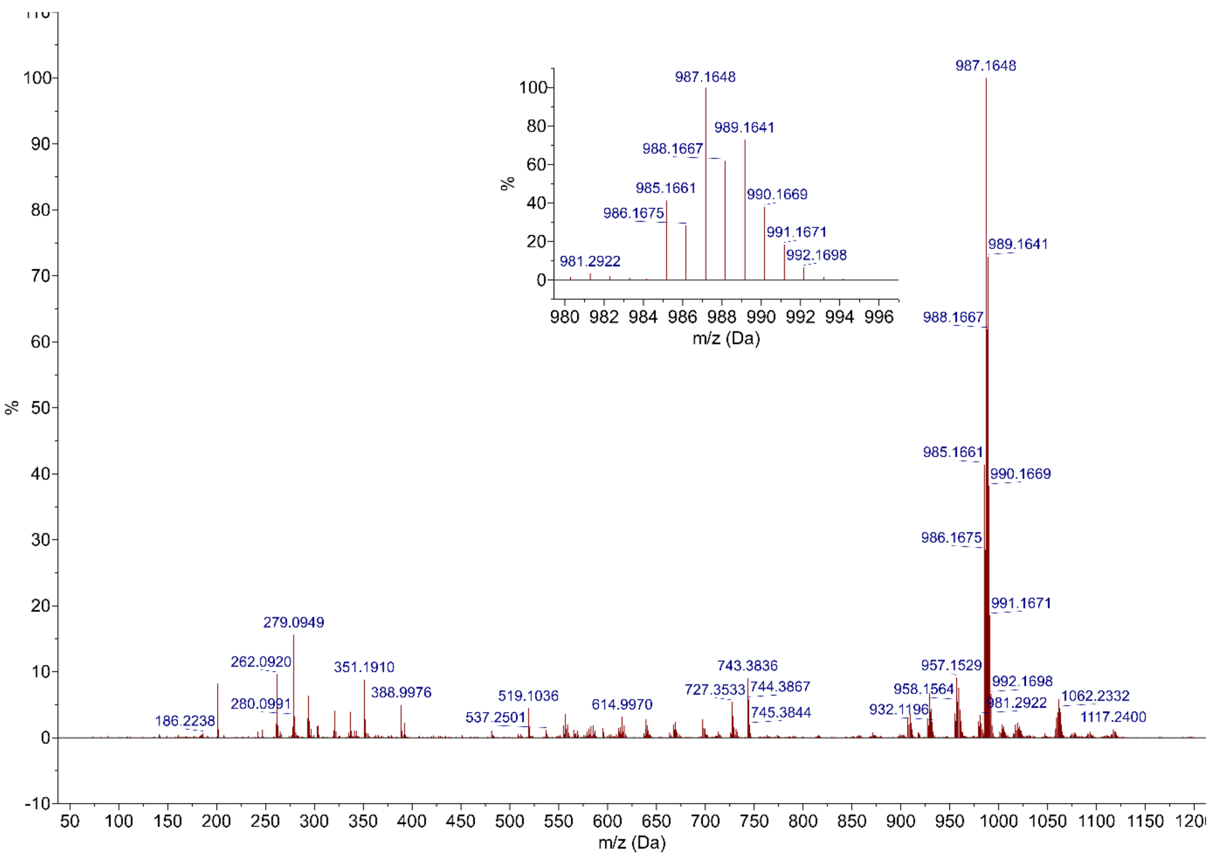

21c MS (ESI) *m/z*: calc. $[C_{50}H_{47}Br_2F_4N_4O_2SSi_2]^+$ = 1057.1261, found: 1057.1283

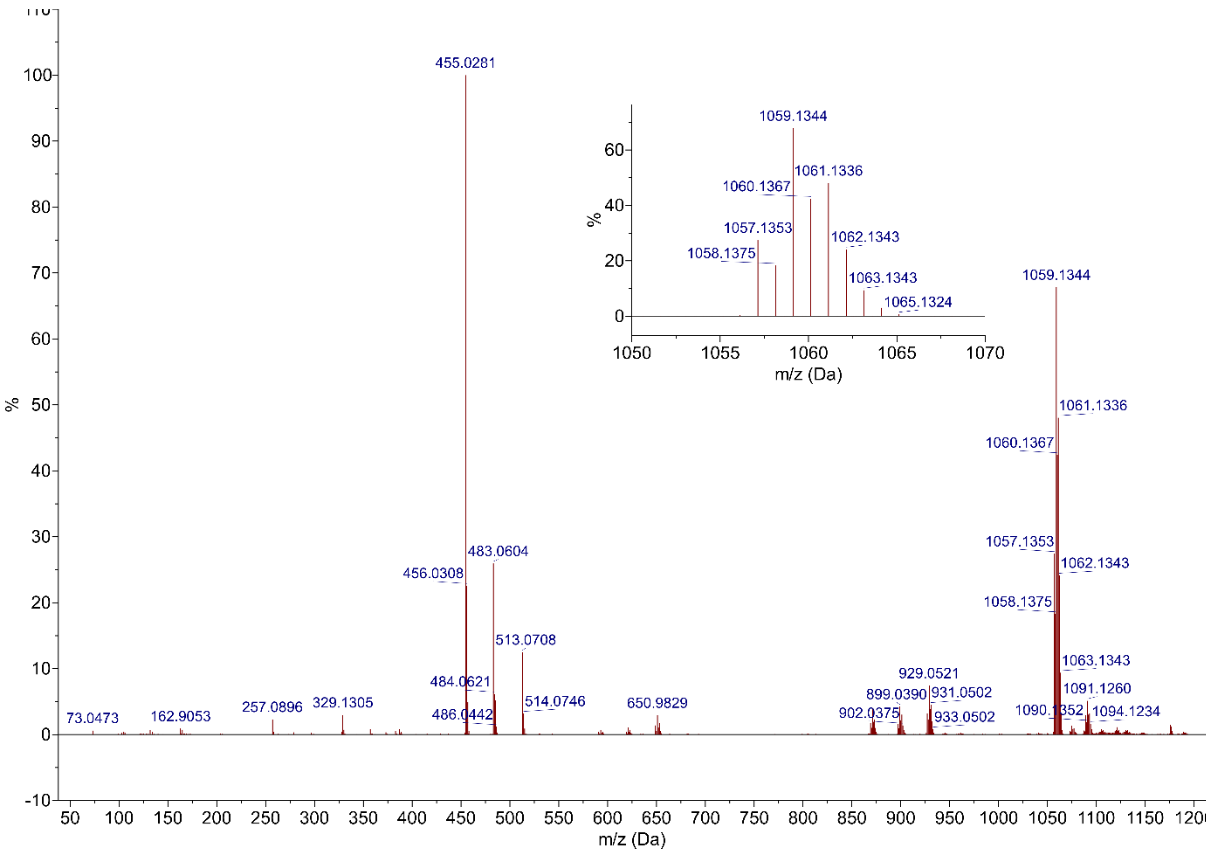

22a MS (ESI) *m/z*: calc. $[C_{54}H_{61}N_4O_6Si_2S_3]^+$ = 1013.3290, found: 1013.3360, calc. $[C_{54}H_{62}N_4O_6Si_2S_3]^{2+}$ = 507.1680, found: 507.1726

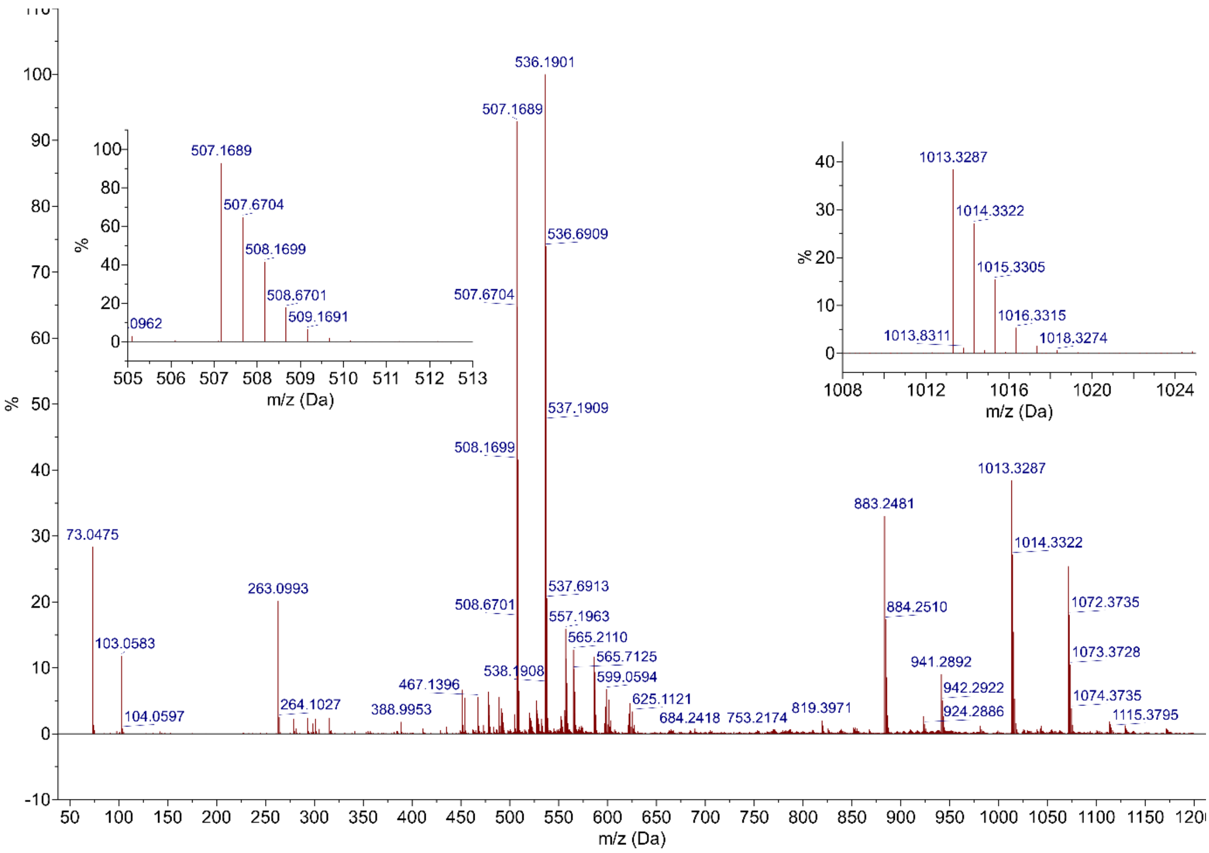

22b MS (ESI) *m/z*: calc. $[C_{50}H_{53}N_4O_2S_3Si_2]^+$ = 893.2869, found: 893.2881

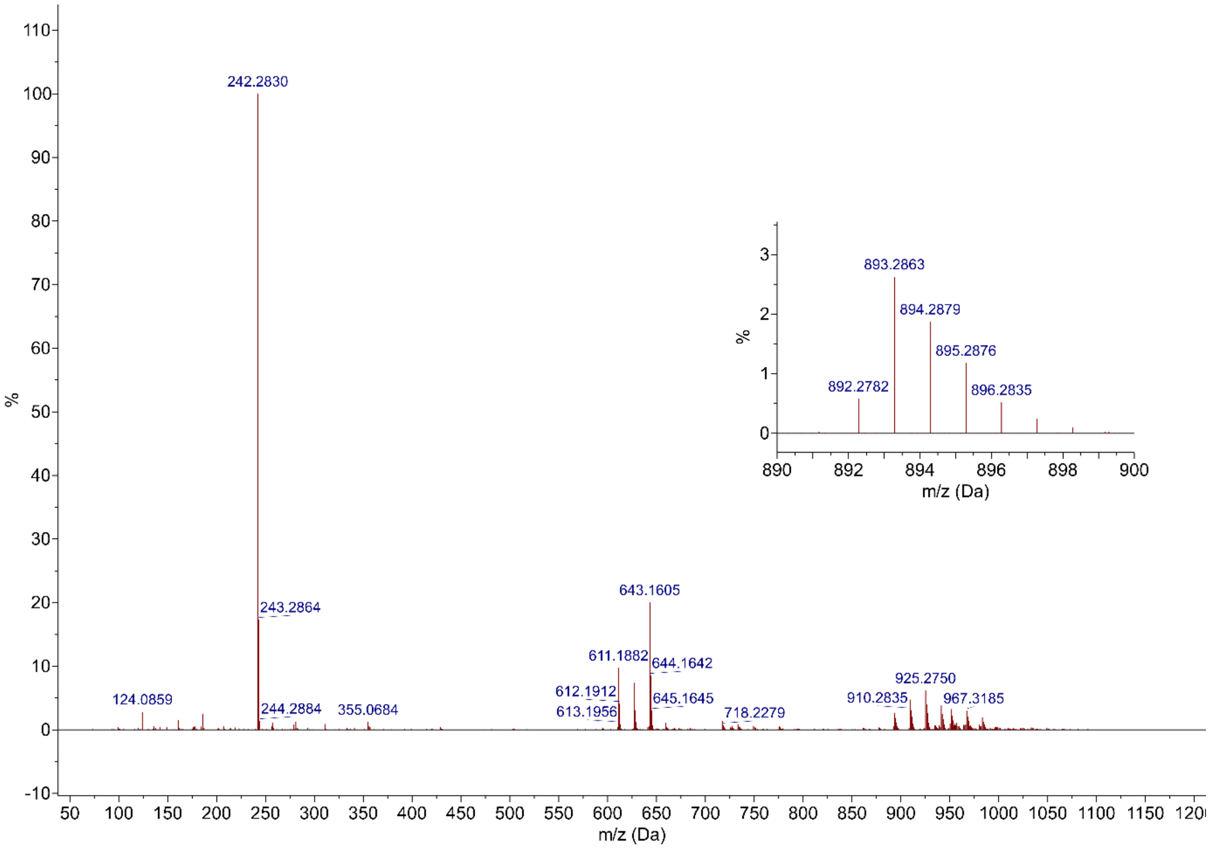

22c MS (ESI) *m/z*: calc. $[C_{50}H_{49}F_4N_4O_2S_3Si_2]^+$ = 965.2492, found: 965.2474

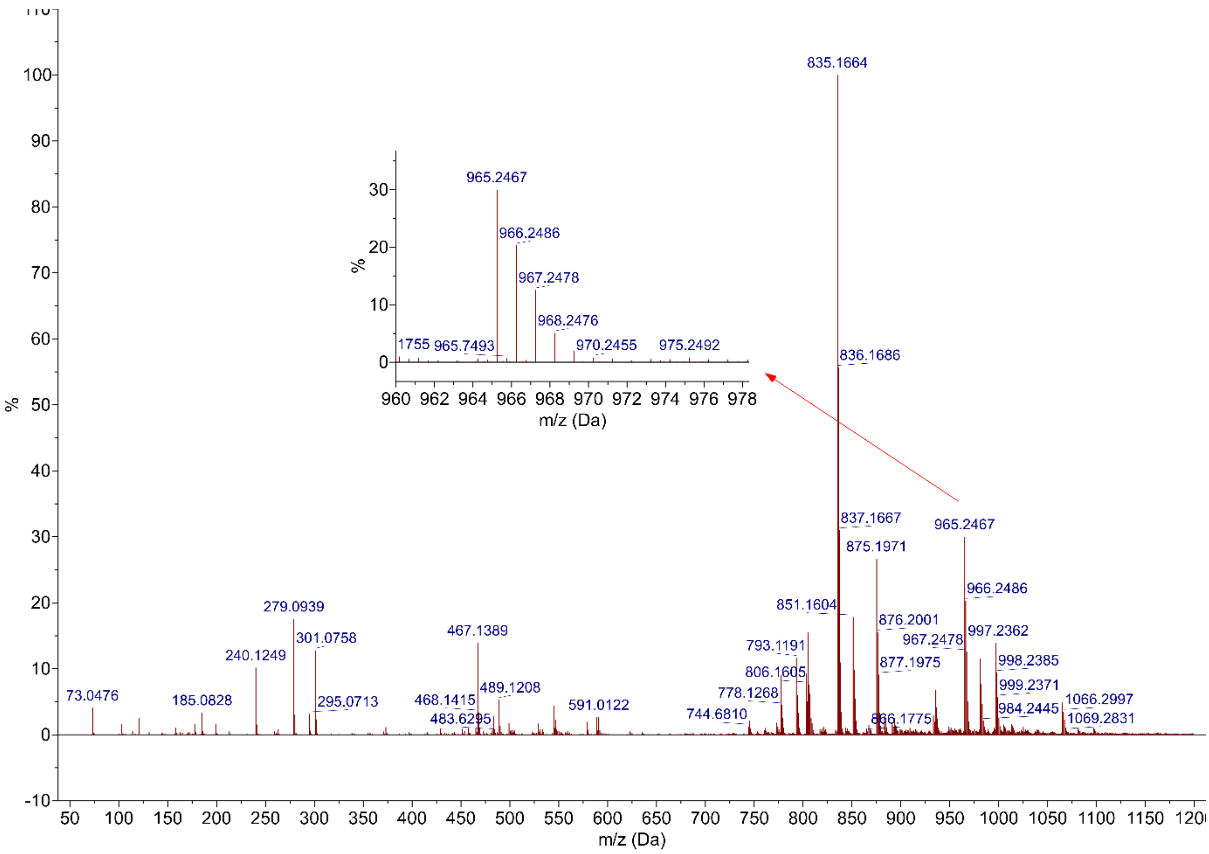

S4a MS (ESI) *m/z*: calc. $[C_{42}H_{33}N_4O_4S_3]^+$ = 753.1659, found: 753.1716

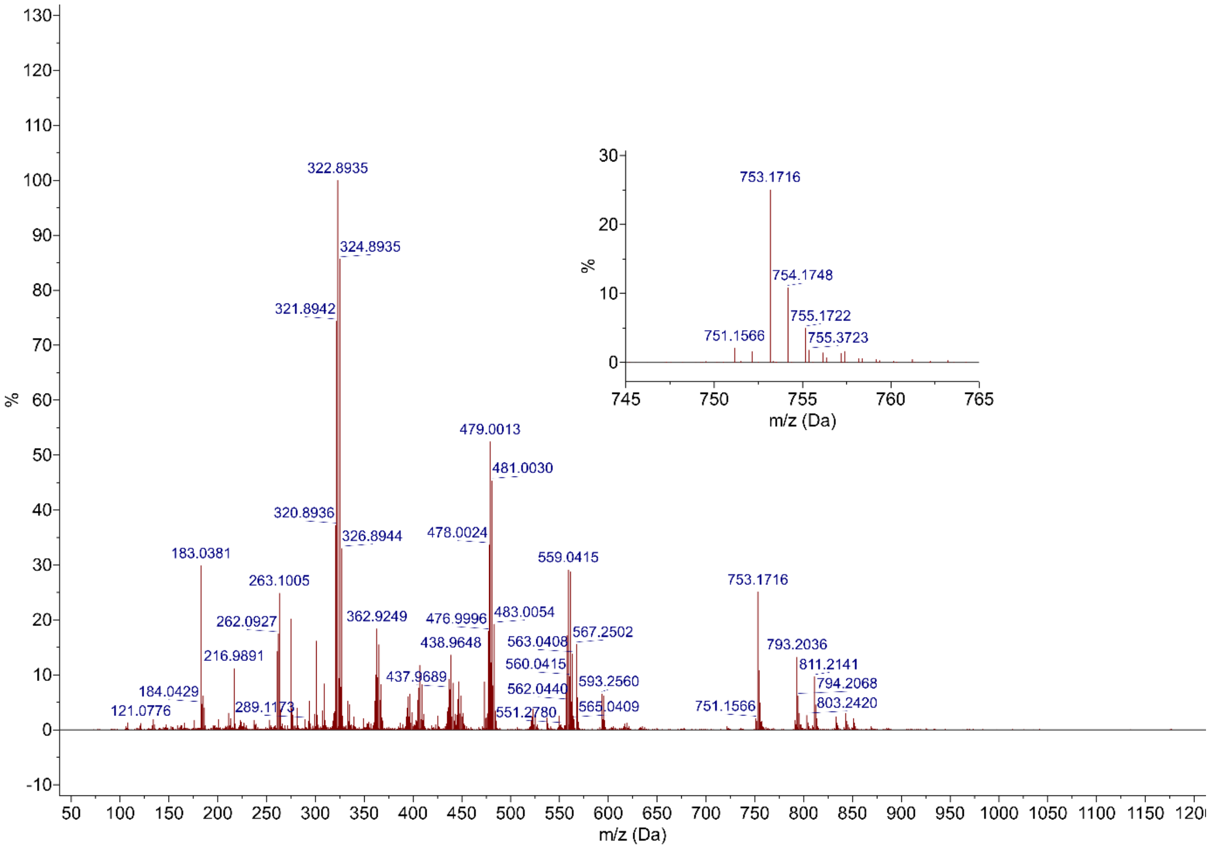

S4b MS (ESI) *m/z*: calc. $[C_{38}H_{25}N_4S_3]^+$ = 633.1236, found: 633.1234

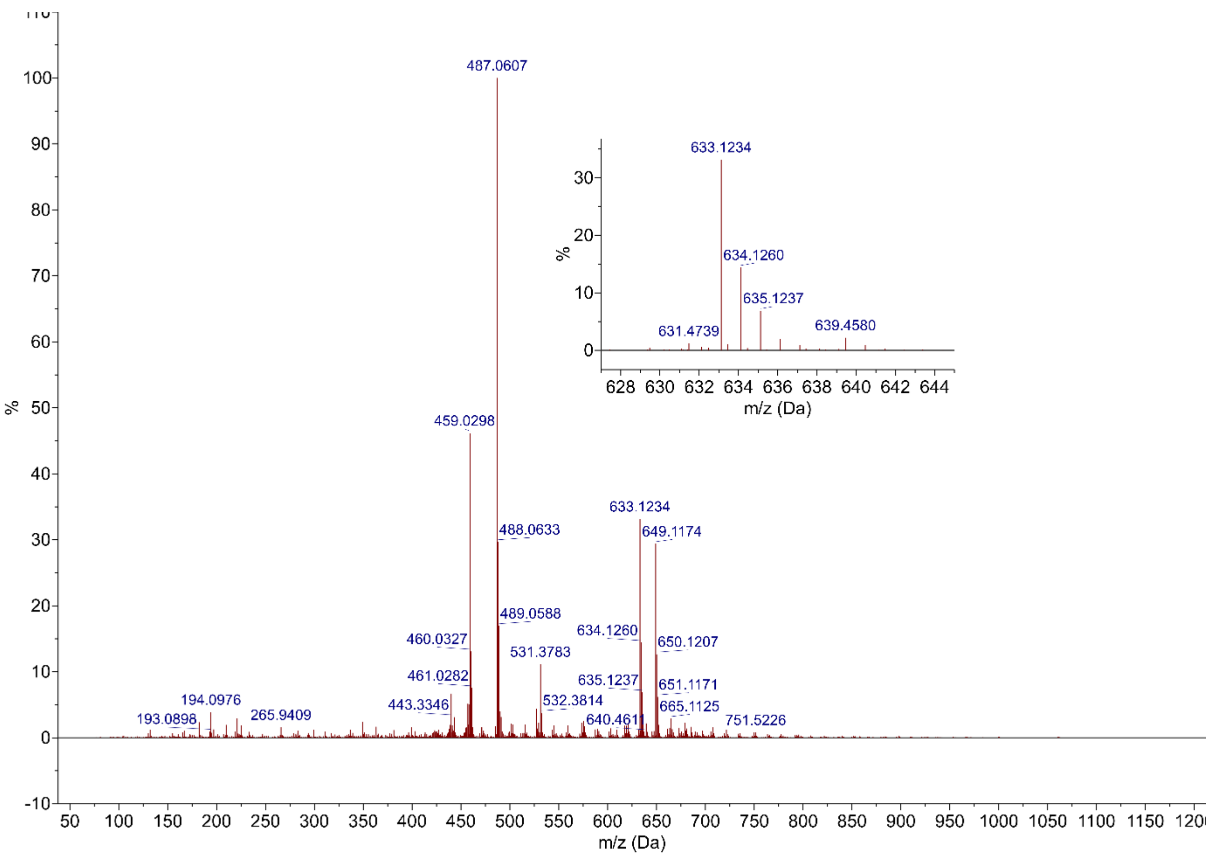

S4c MS (ESI) *m/z*: calc. $[C_{38}H_{21}F_4N_4S_3]^+$ = 705.0859, found: 705.0803

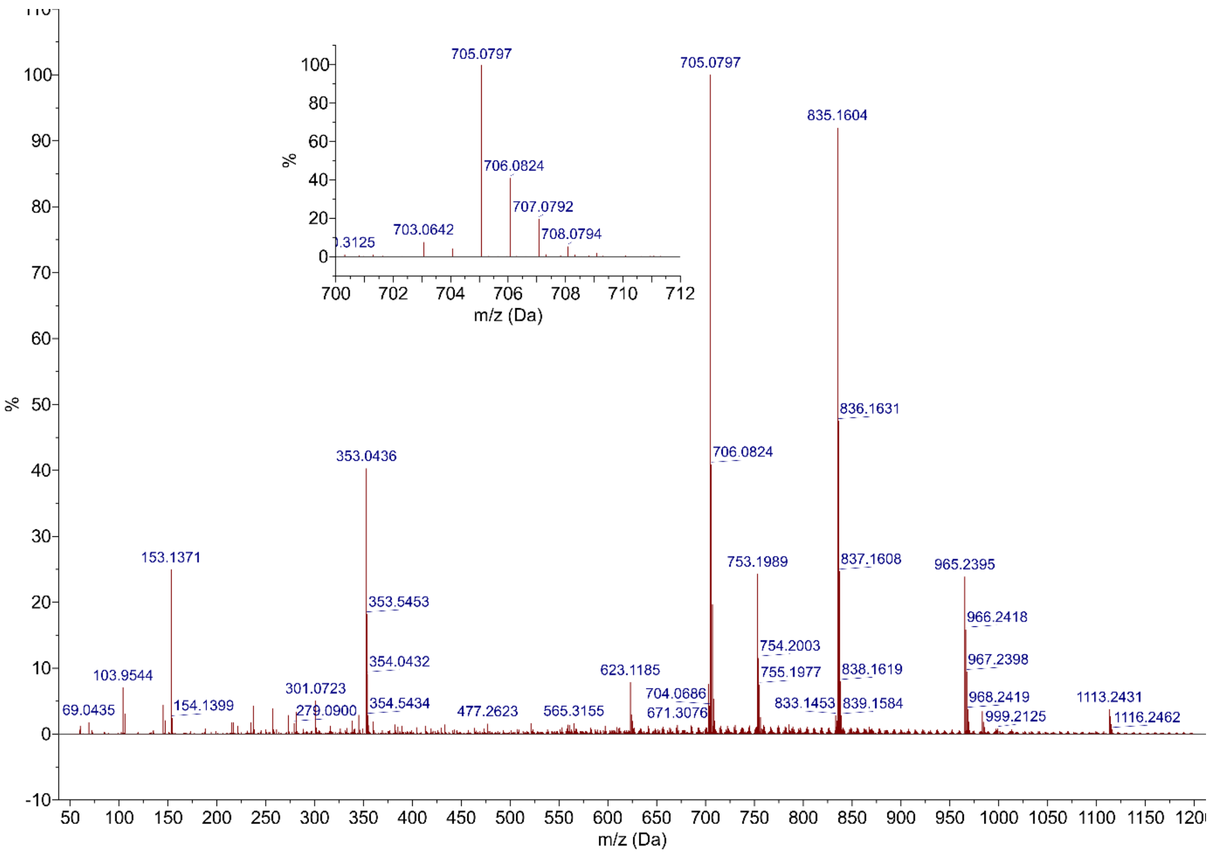

8a MS (ESI) *m/z*: calc. $[C_{42}H_{31}N_4O_4S_3]^+$ = 751.1516, found: 753.1660, calc. $[C_{42}H_{32}N_4O_4S_3]^{2+}$ = 376.0787, found: 376.0786

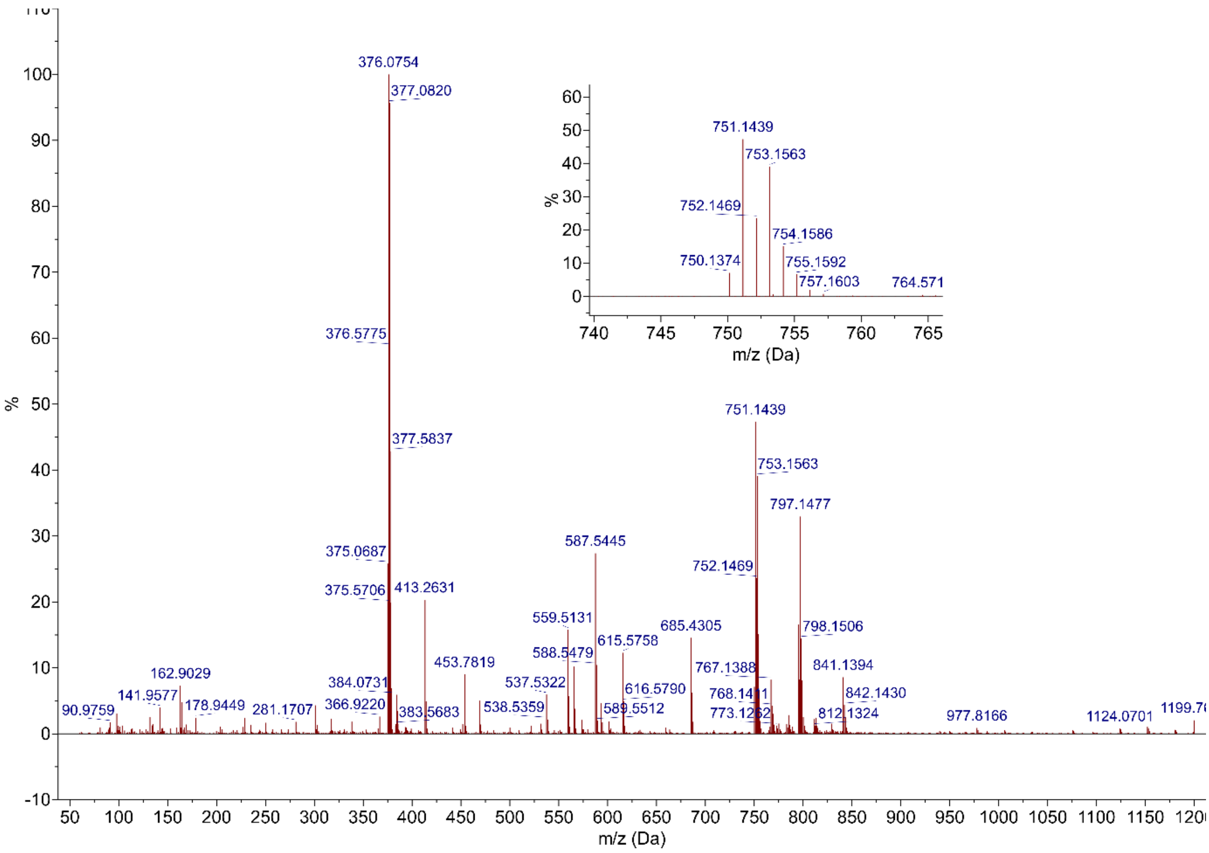

8b MS (ESI) *m/z*: calc. $[C_{38}H_{23}N_4S_3]^+$ = 631.1085, found: 631.1086

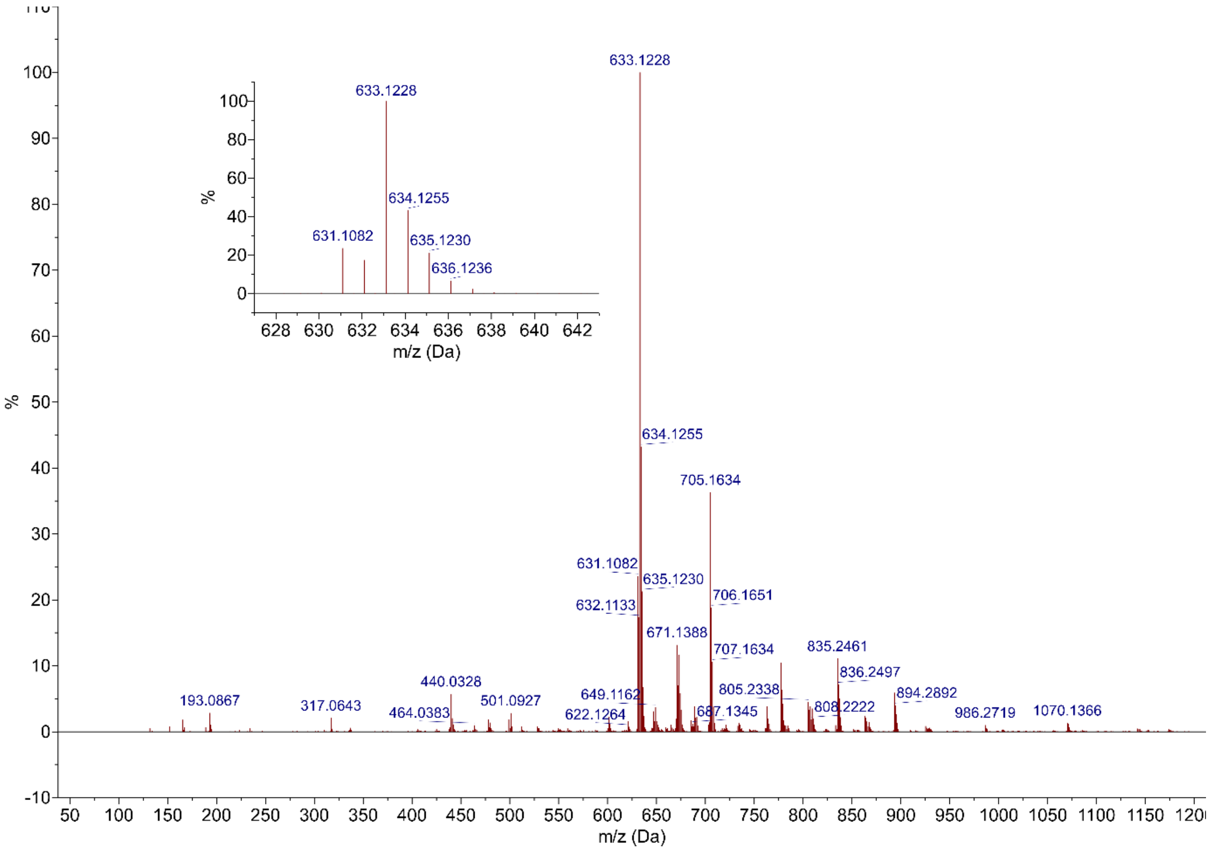

8c MS (ESI) *m/z*: calc. $[C_{38}H_{19}F_4N_4S_3]^+$ = 703.0708, found: 703.0714

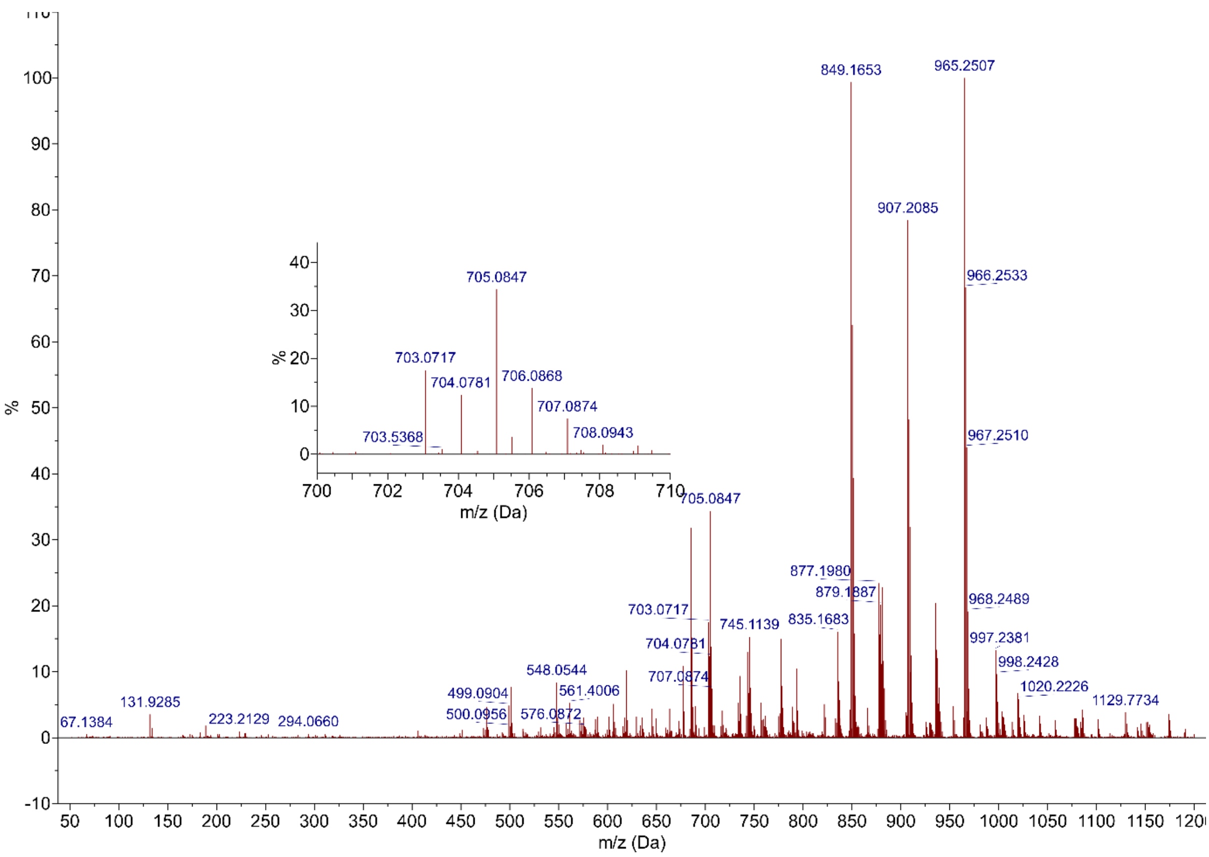

24 MS (ESI) *m/z*: calc. $[C_{16}H_{19}Si_2S_2Br_2]$ = 488.8833 $[M]^+$, found m/z: 488.8824

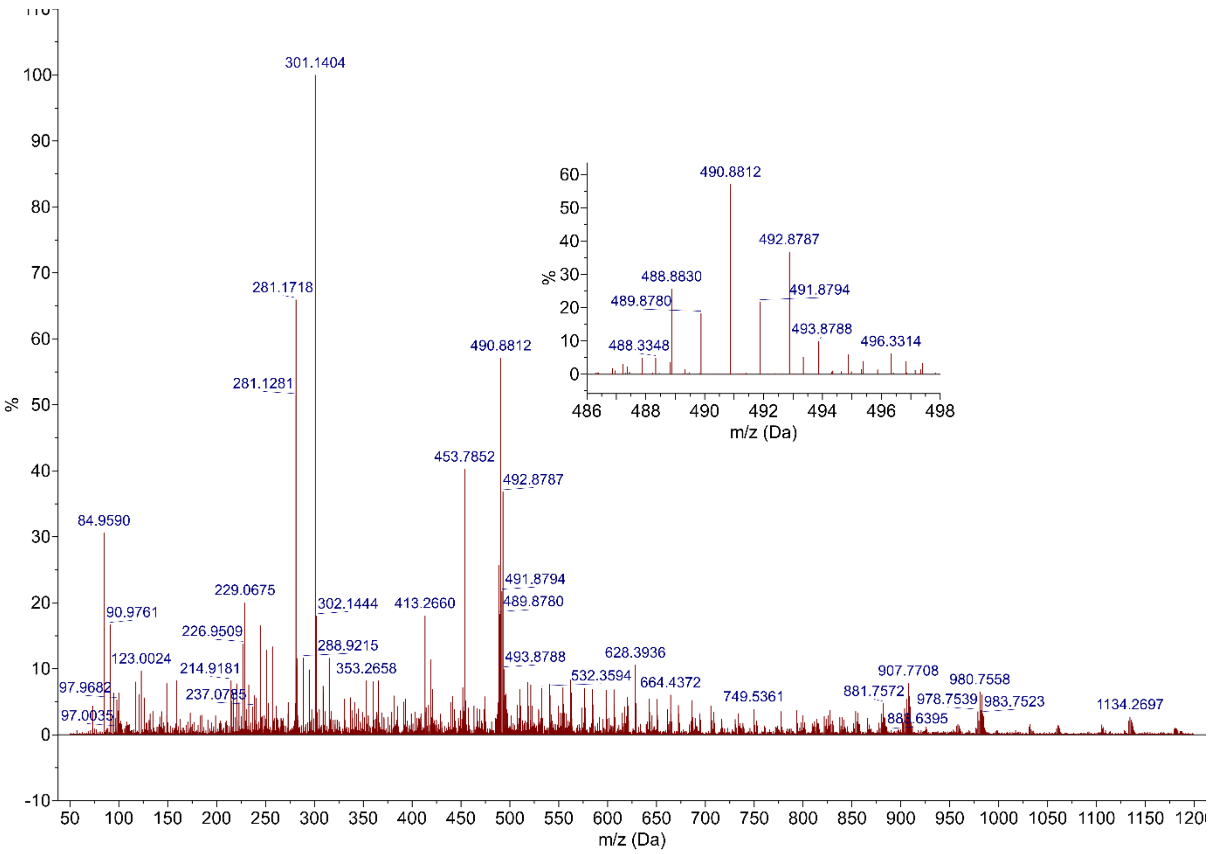

23 MS (ESI) *m/z*: calc. [$C_{56}H_{59}N_4O_6Si_2S_2Br_2$] = 1161.1781 [M]$^+$, found m/z: 1161.1721

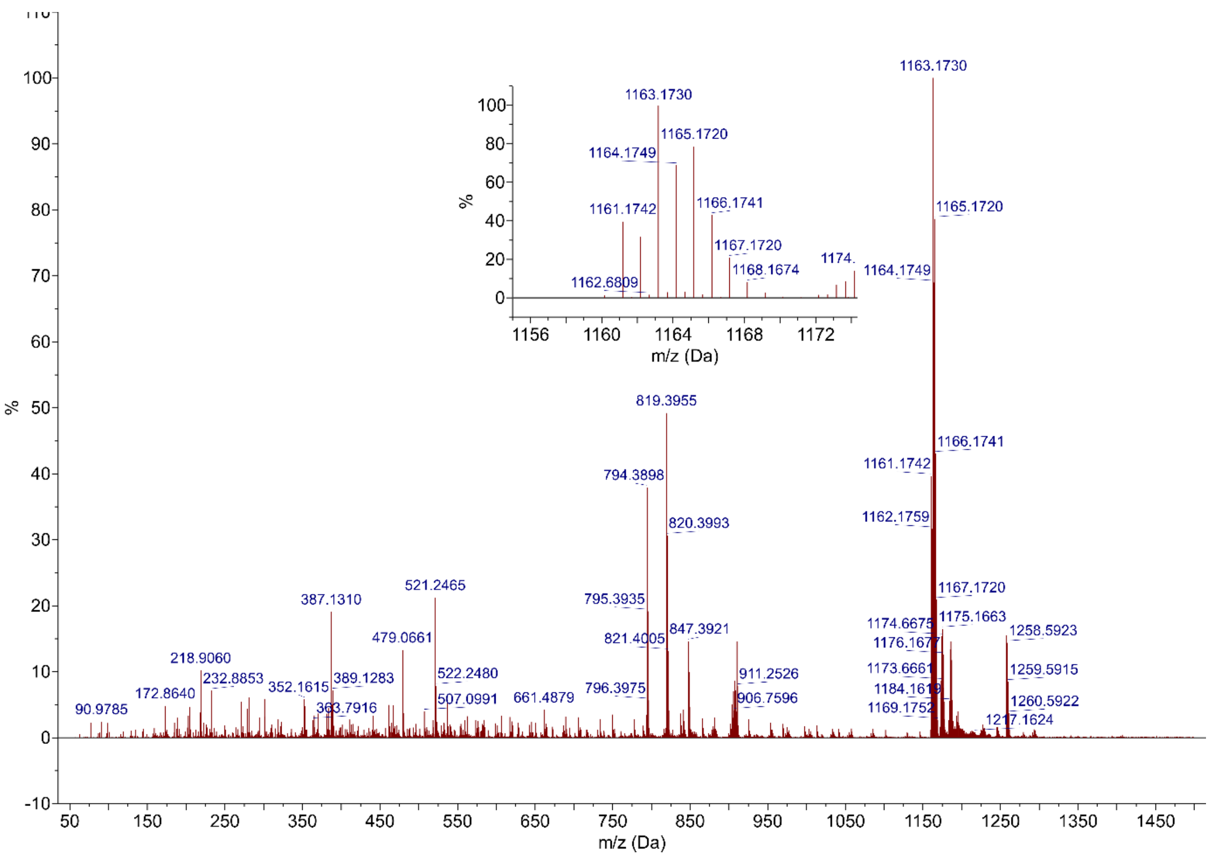

26 MS (ESI) *m/z*: calc. [$C_{44}H_{33}N_4O_4S_4$] = 809.1385 [M]$^+$, found m/z: 809.1378

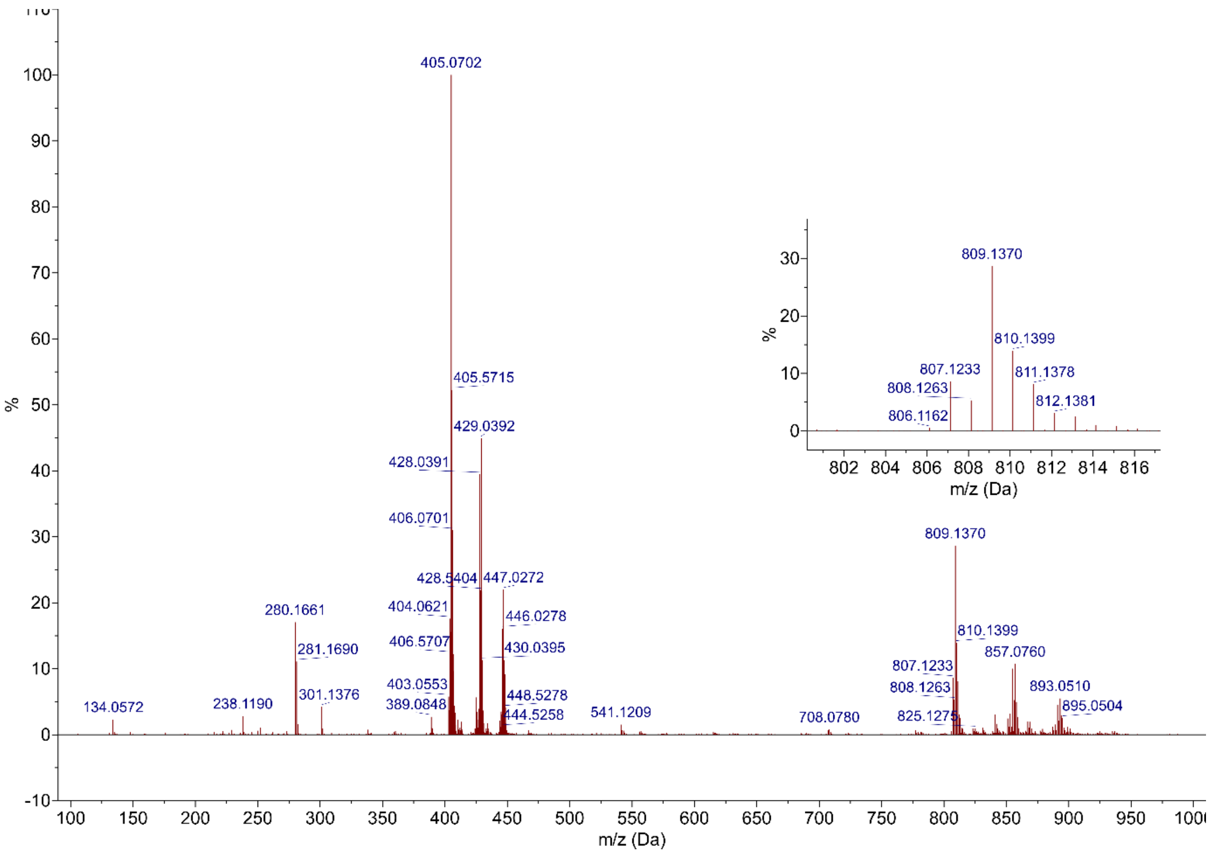

9 MS (ESI) *m/z*: calc. [$C_{44}H_{31}N_4O_4S_4$] = 807.1228 $[M]^+$, found m/z: 807.1215

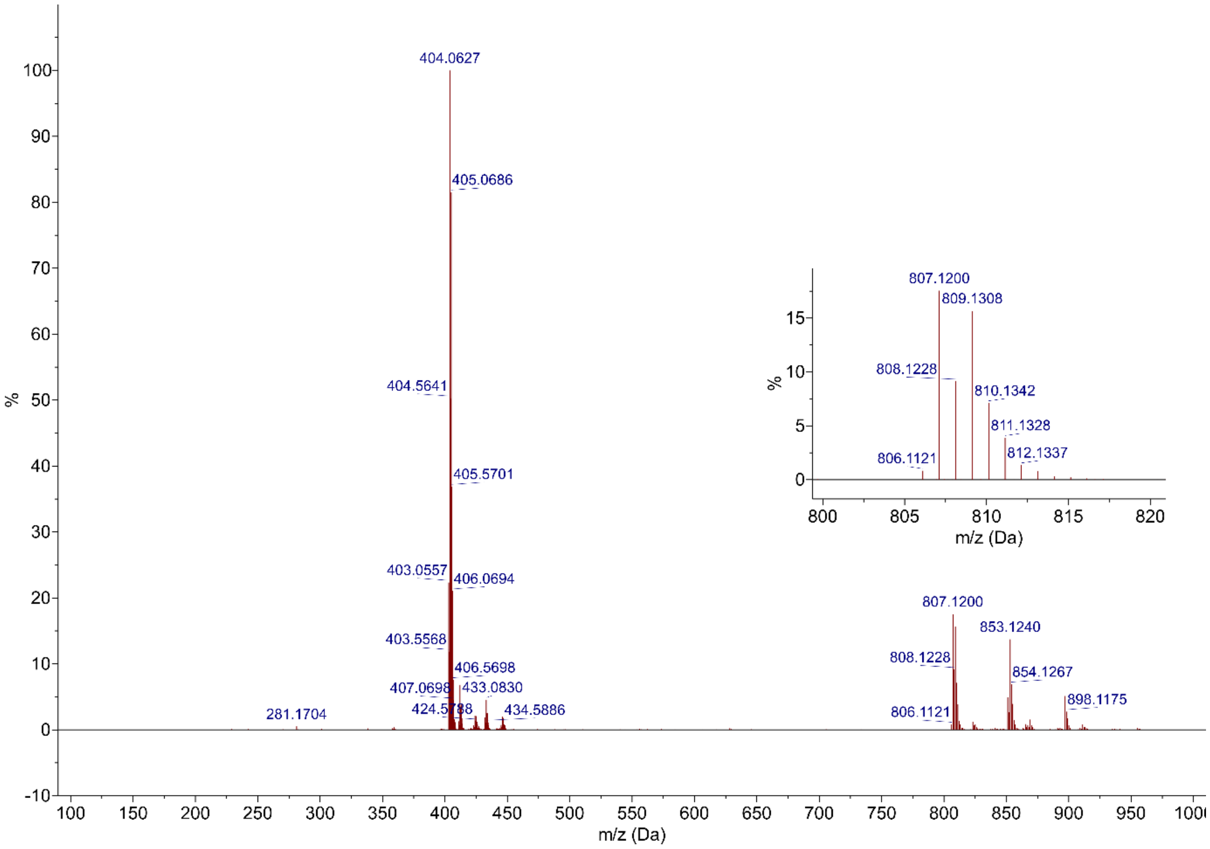

Mass spectra of compound **24** cannot be obtained due to the poor solubility.

3.5. UV-Vis and fluorescence spectra

12 UV-Vis and fluorescence spectra in moisture-free DCM (20.5 µM)

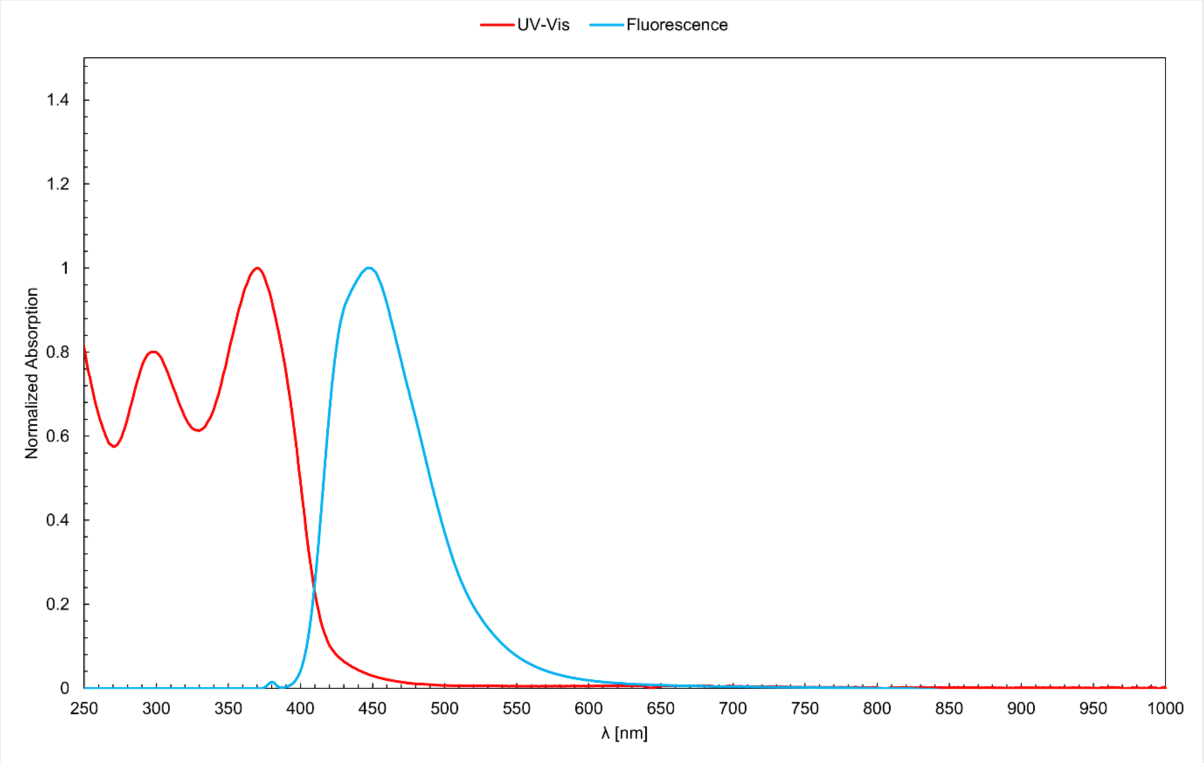

4 UV-Vis spectrum in moisture-free DCM (15.8 µM)

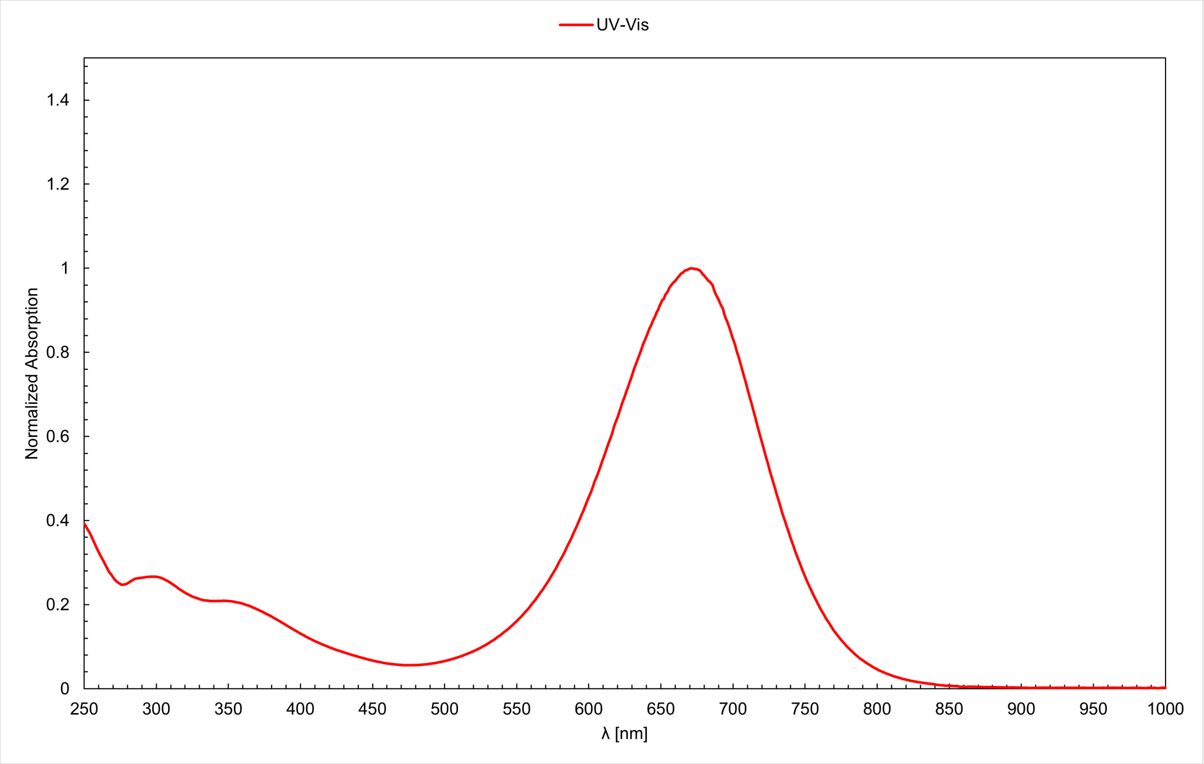

14 UV-Vis and fluorescence spectra in moisture-free DCM (23.4 μM)

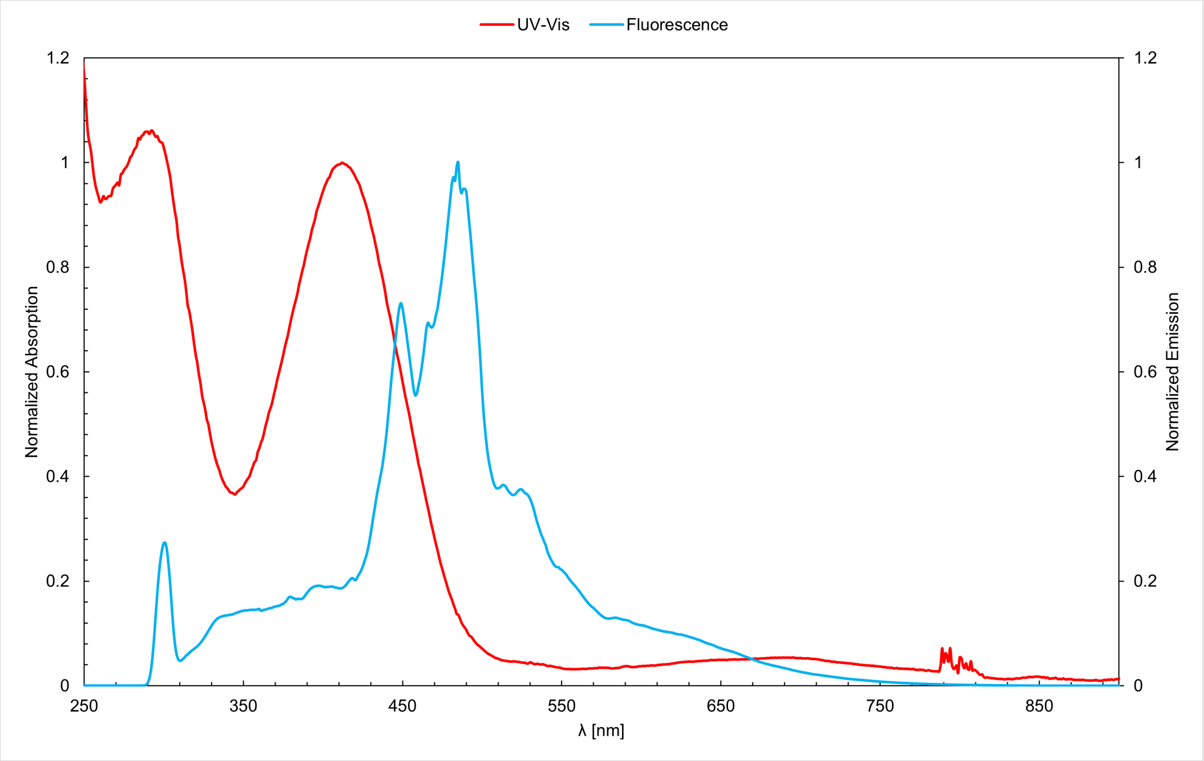

6 UV-Vis spectrum in moisture-free DCM (18.8 μM)

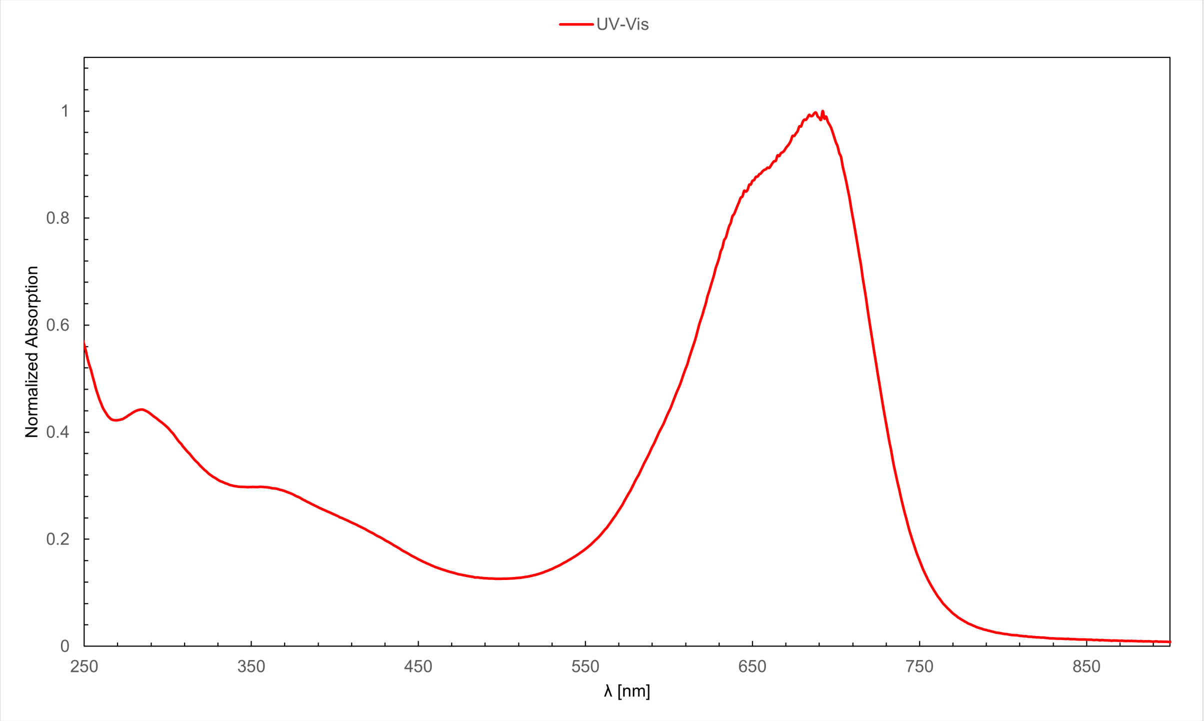

16 UV-Vis and fluorescence spectra in moisture-free DCM (20.0 µM)

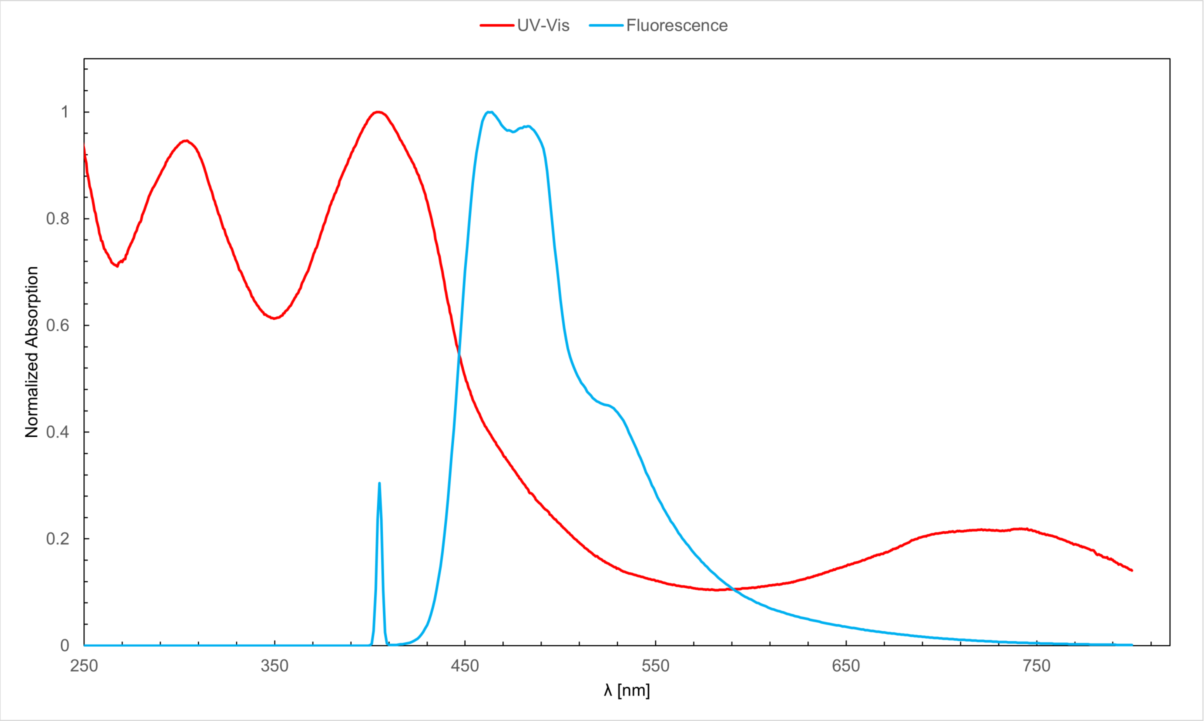

7 UV-Vis spectrum in moisture-free DCM (16.0 µM)

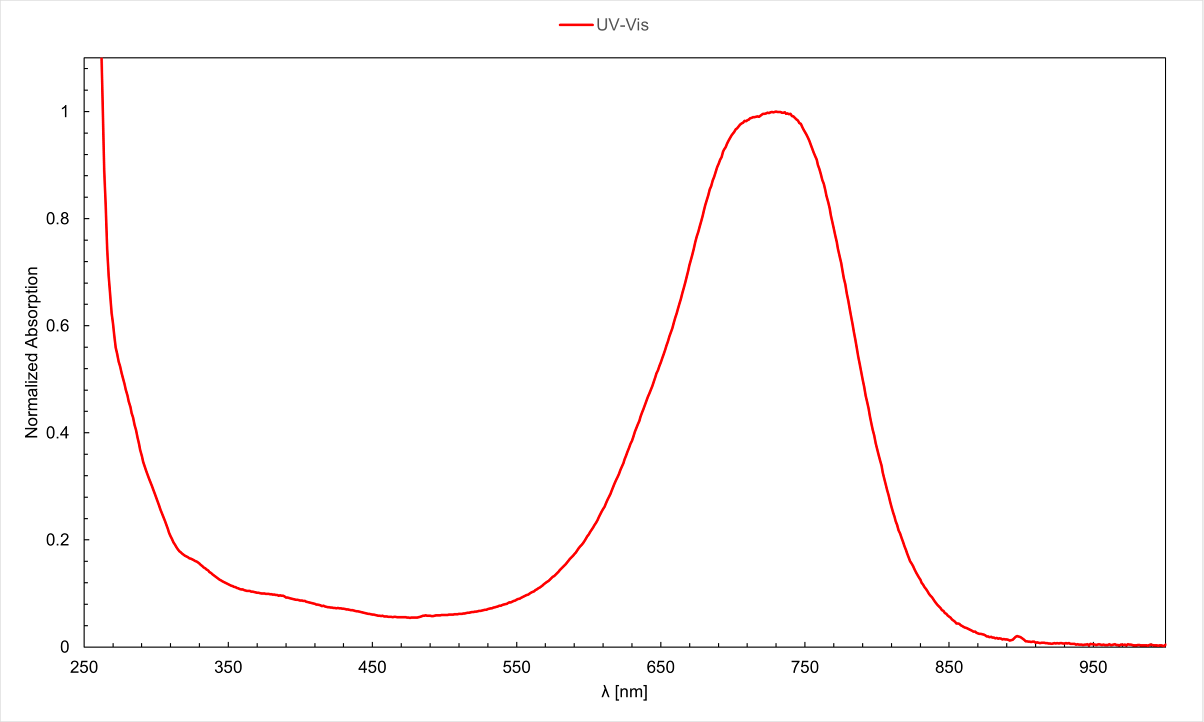

22a UV-Vis and fluorescence spectra in moisture-free DCM (13.1 µM)

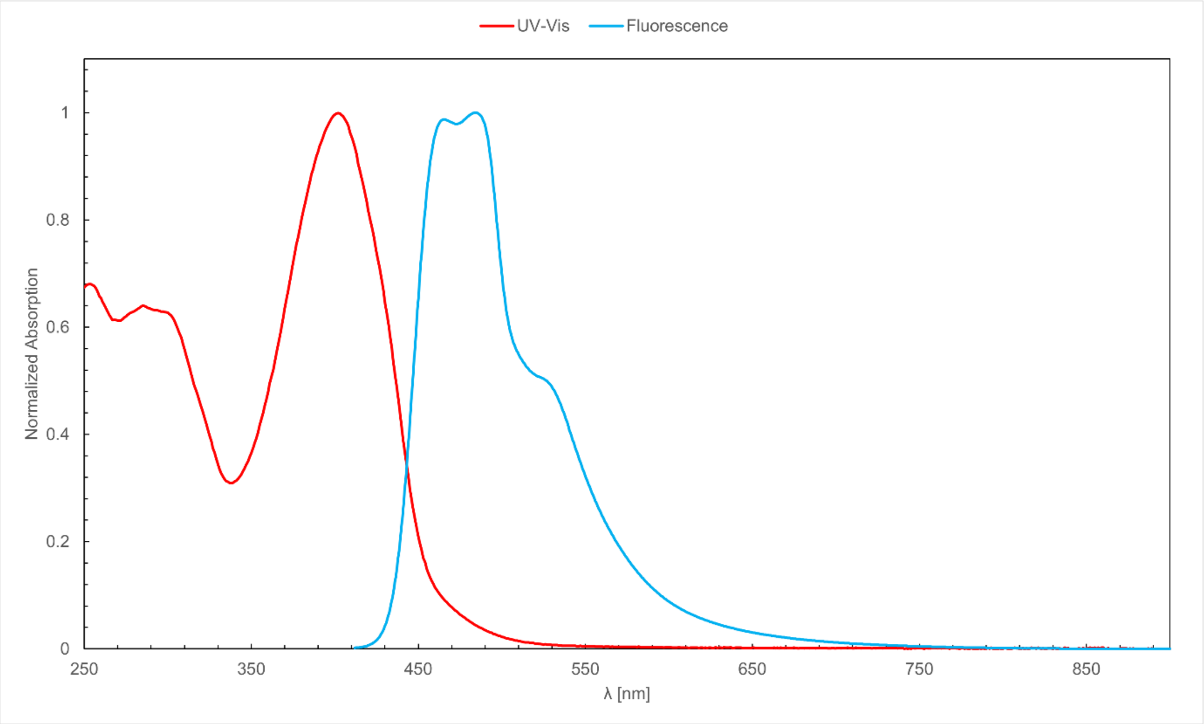

22b UV-Vis and fluorescence spectra in moisture-free DCM (16.8 µM)

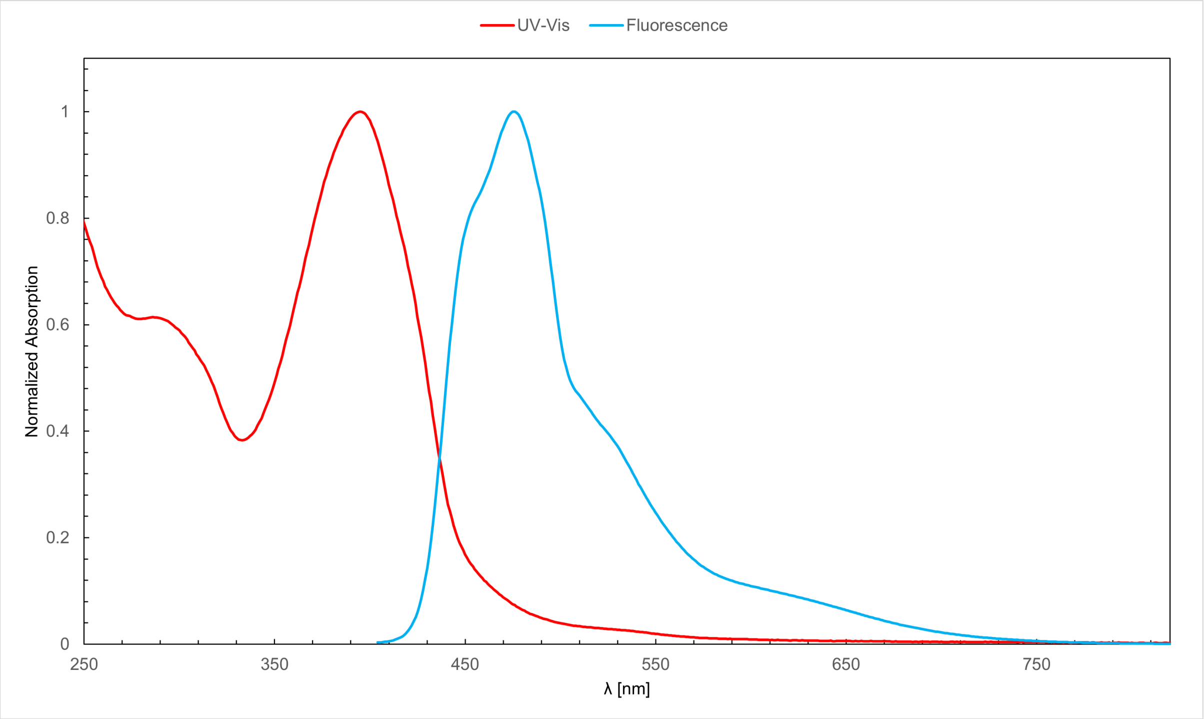

22c UV-Vis and fluorescence spectra in moisture-free DCM (12.4 μM)

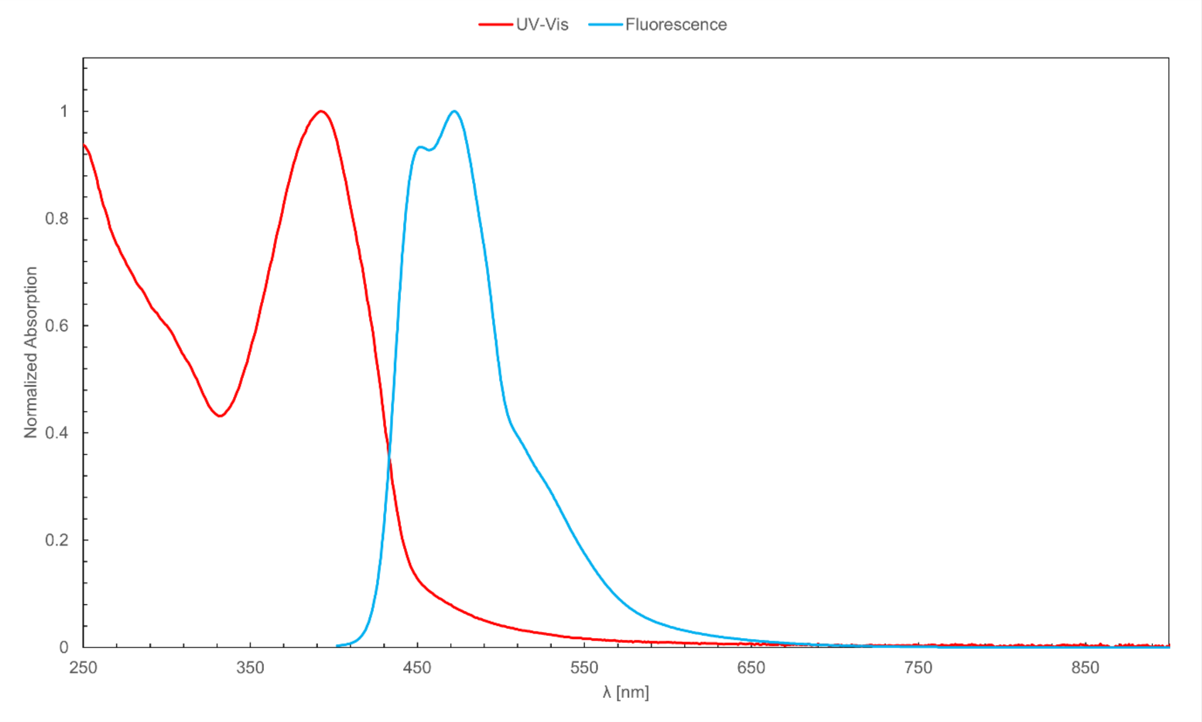

8a UV-Vis spectrum in moisture-free DCM (19.9 μM)

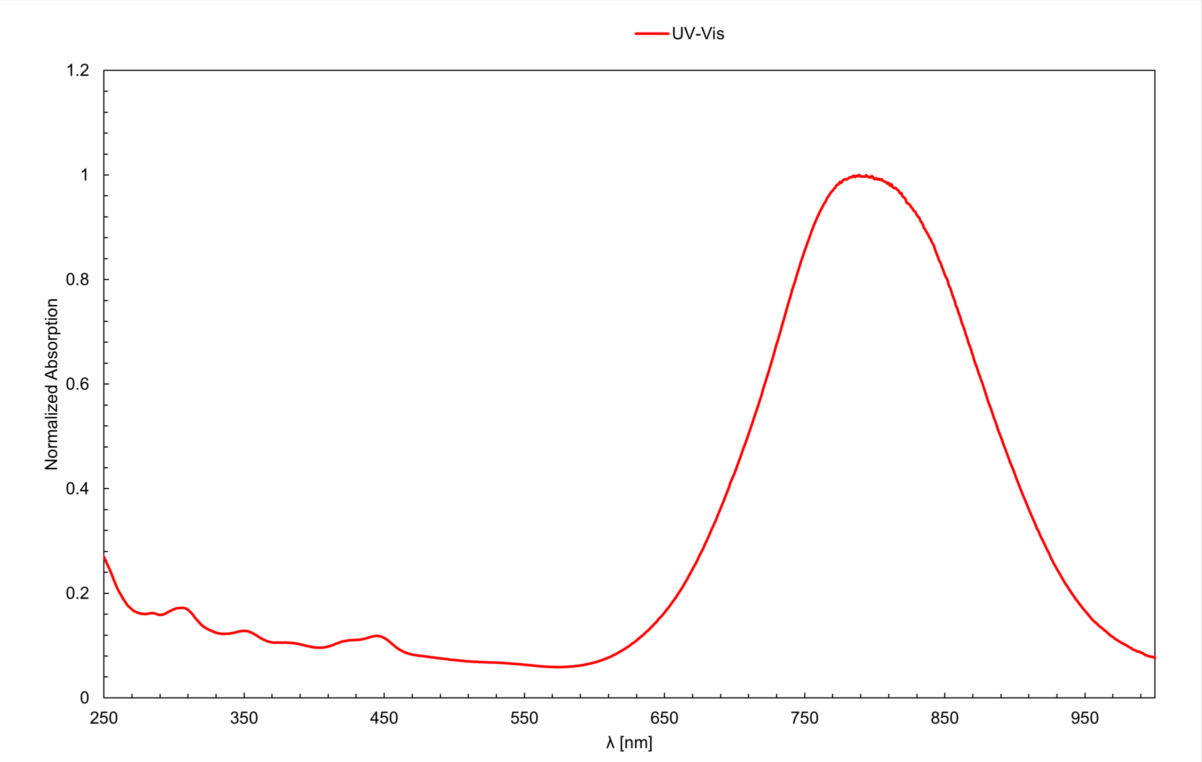

8b UV-Vis spectrum in moisture-free DCM (19.0 µM)

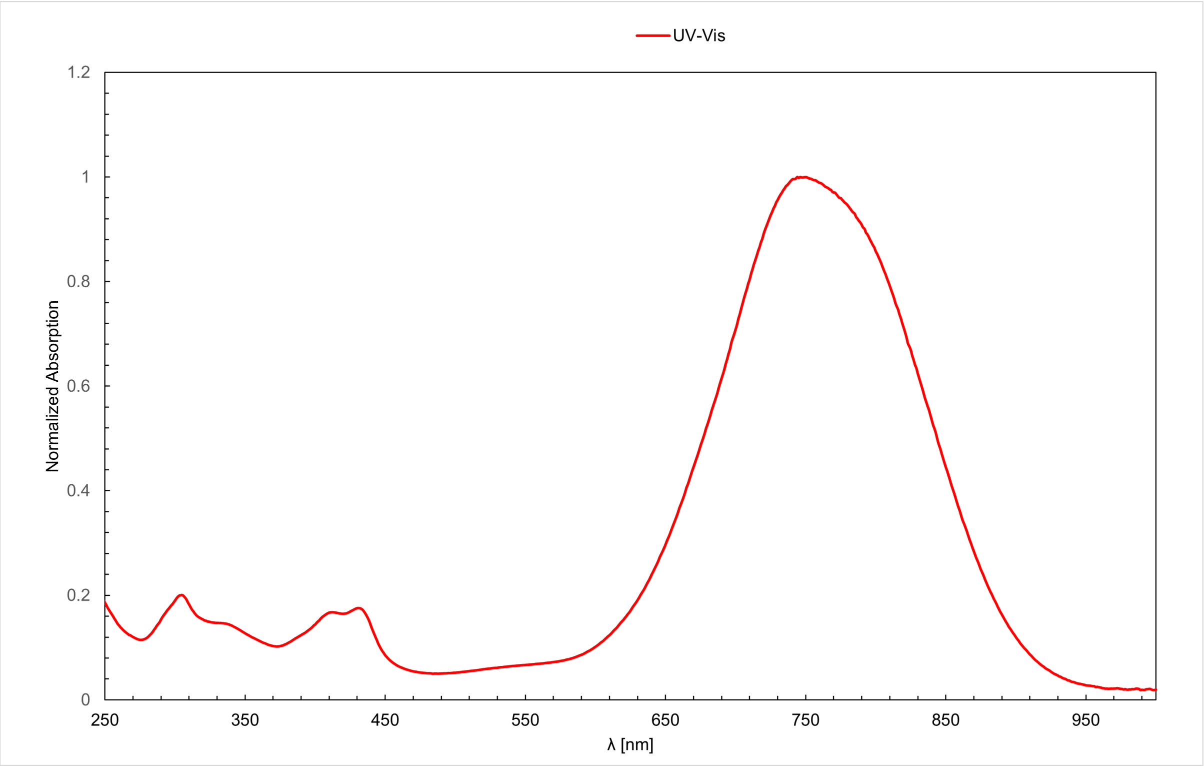

8c UV-Vis spectrum in moisture-free DCM (17.0 µM)

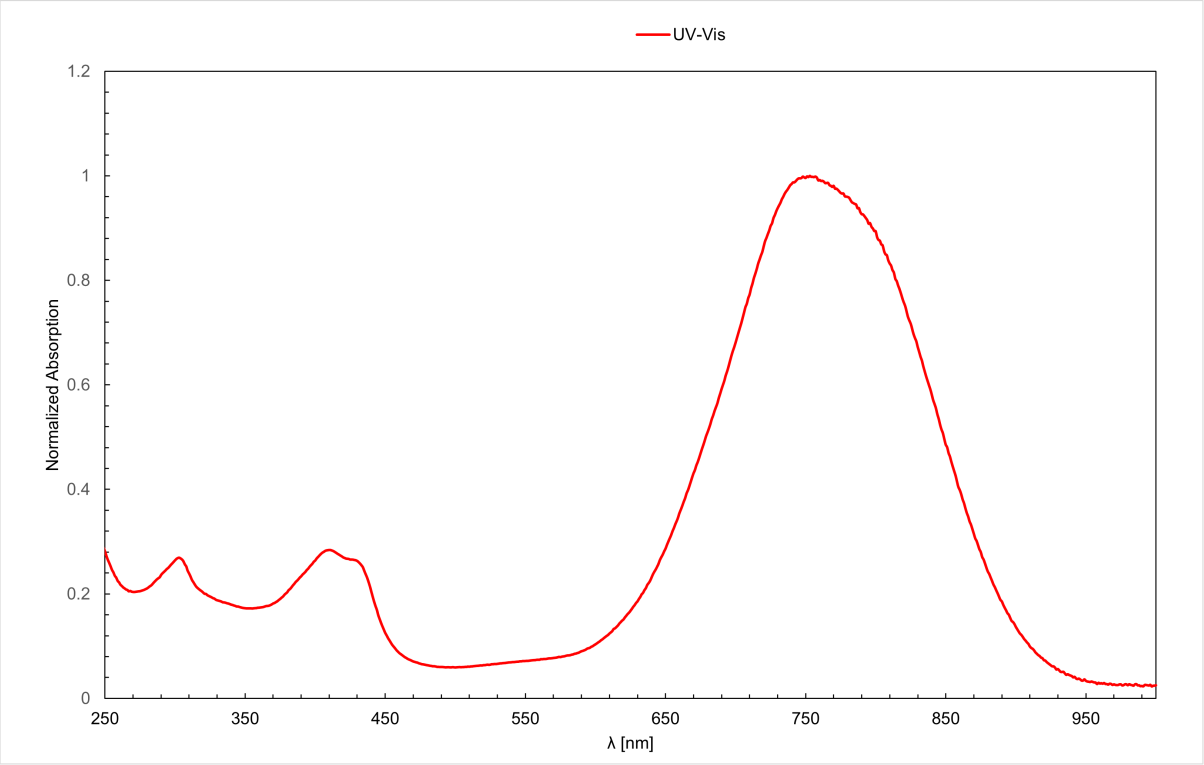

26 UV-Vis and fluorescence spectra in moisture-free DCM (9.4 µM)

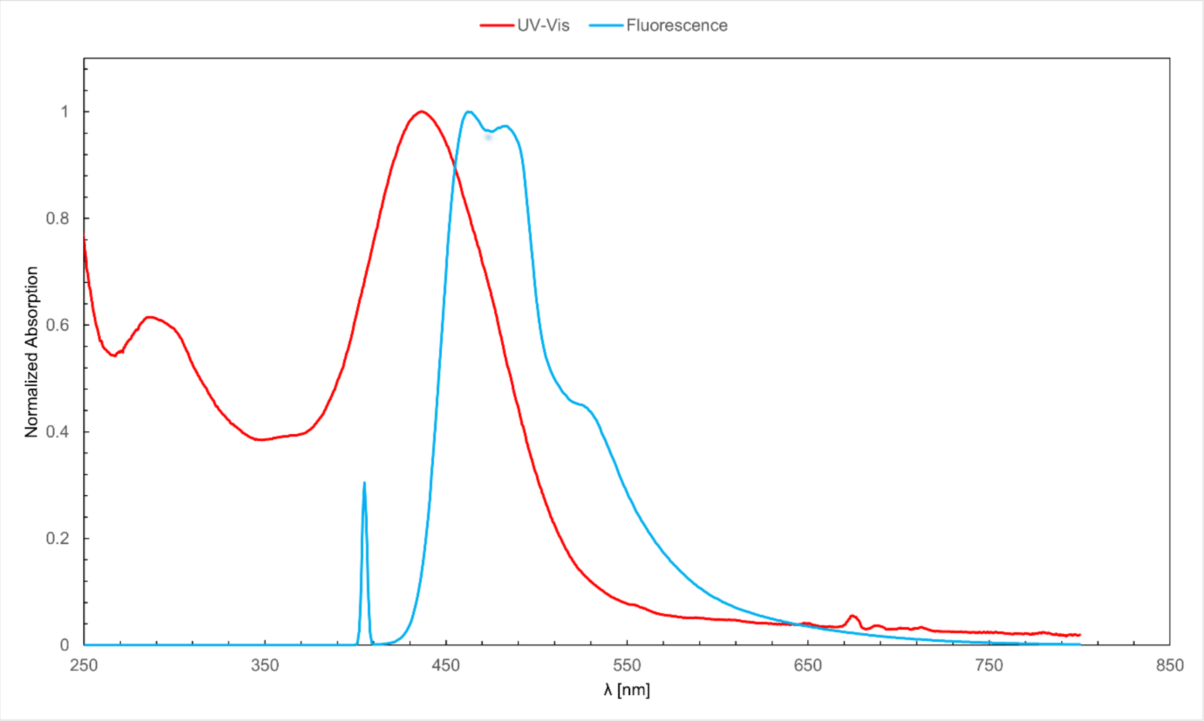

9 UV-Vis spectrum in moisture-free DCM (6.8 µM)

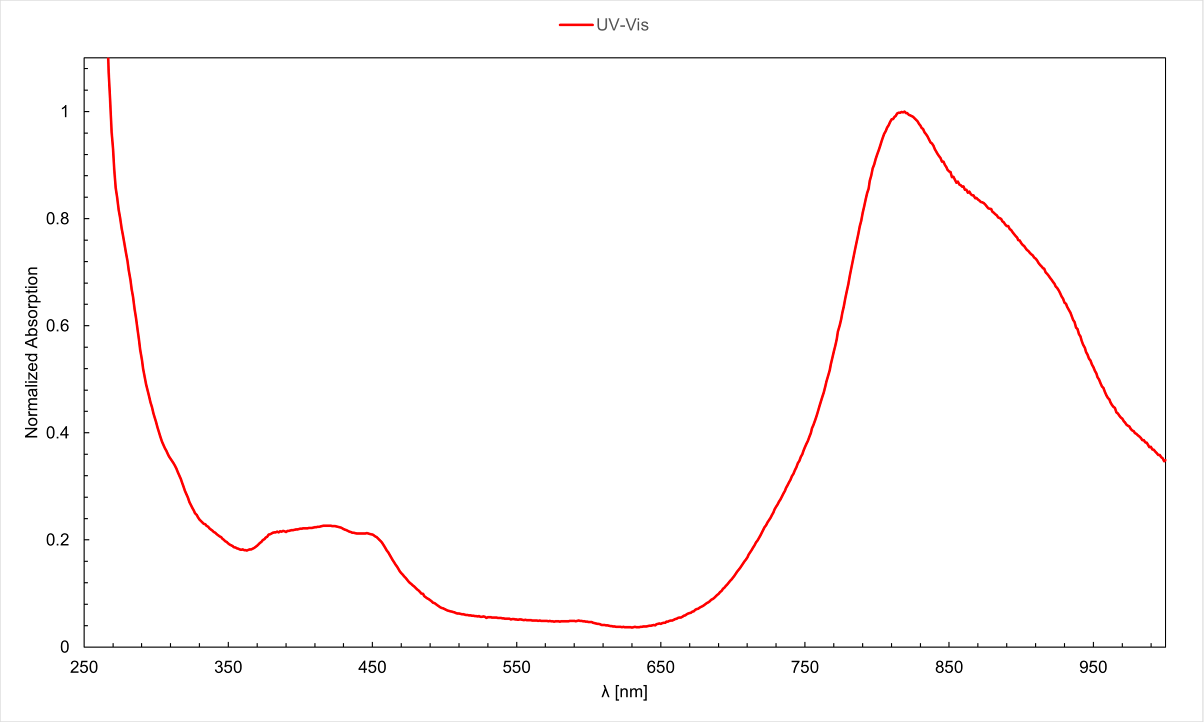

Comparation of UV-Vis spectra

Summarized UV-Vis spectra of oxidation precusors in solid lines (**12**, **14**, **16**, **22a**, **22b**, **22c**, **26**) and quinoids in dashed lines (**4**, **6**, **7**, **8a**, **8b**, **8c, 9**).

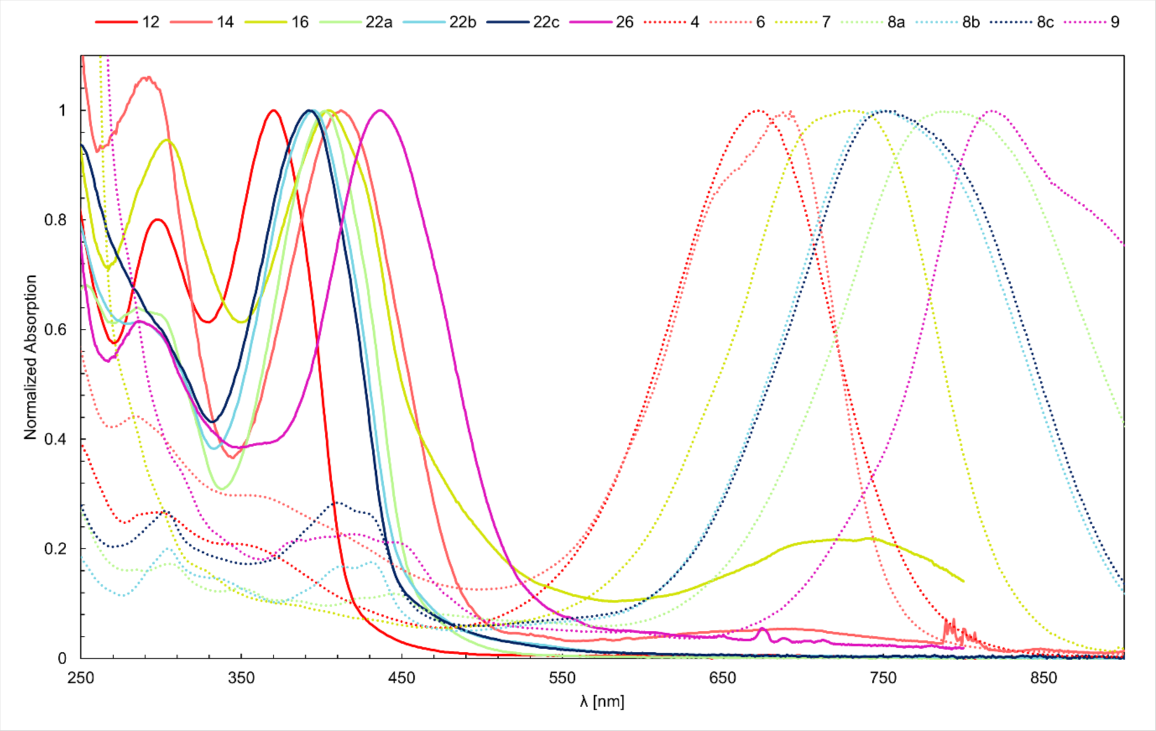

Comparation of fluorescence spetra

Summarized fluorescence spectra of all oxidation precusors. The oxidativ quinoids can not be recorded in fluorescence spetra.

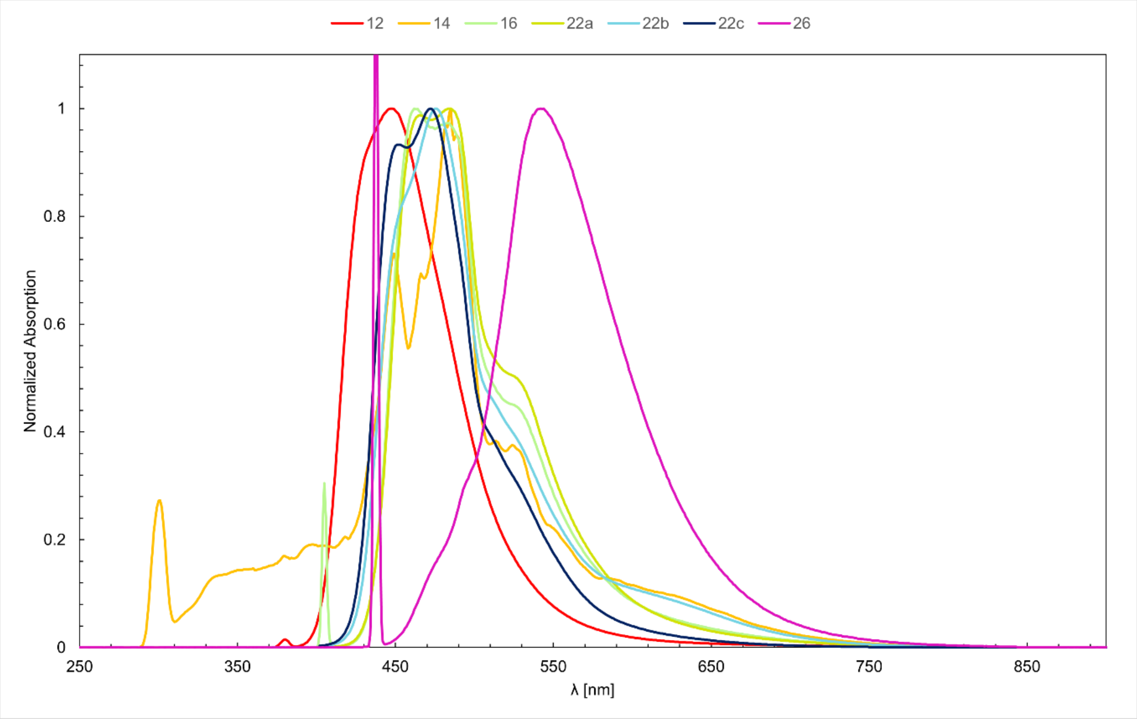

3.6. Cyclic voltammetry measurement

All CV measurements were conducted in dry and degassed solution with DCM as solvent. Tetrabutylammonium hexafluorophosphate (387.4 mg in 10 mL DCM or 774.8 mg in 20 mL DCM, 0.1M) was used as conducting salt and ferrocene was added during the measurement as internal standard to refer to the ferrocene/ferrocene$^+$ couple. The sweep speed was set to 100 mV/s.

Cyclic voltammogram of **12** in CH_2Cl_2 (0.4 mM) at a scan rate of 100 mV/s at room temperature *vs* Fc/Fc^+, Pt/$[NBu_4][PF_6]$/Ag

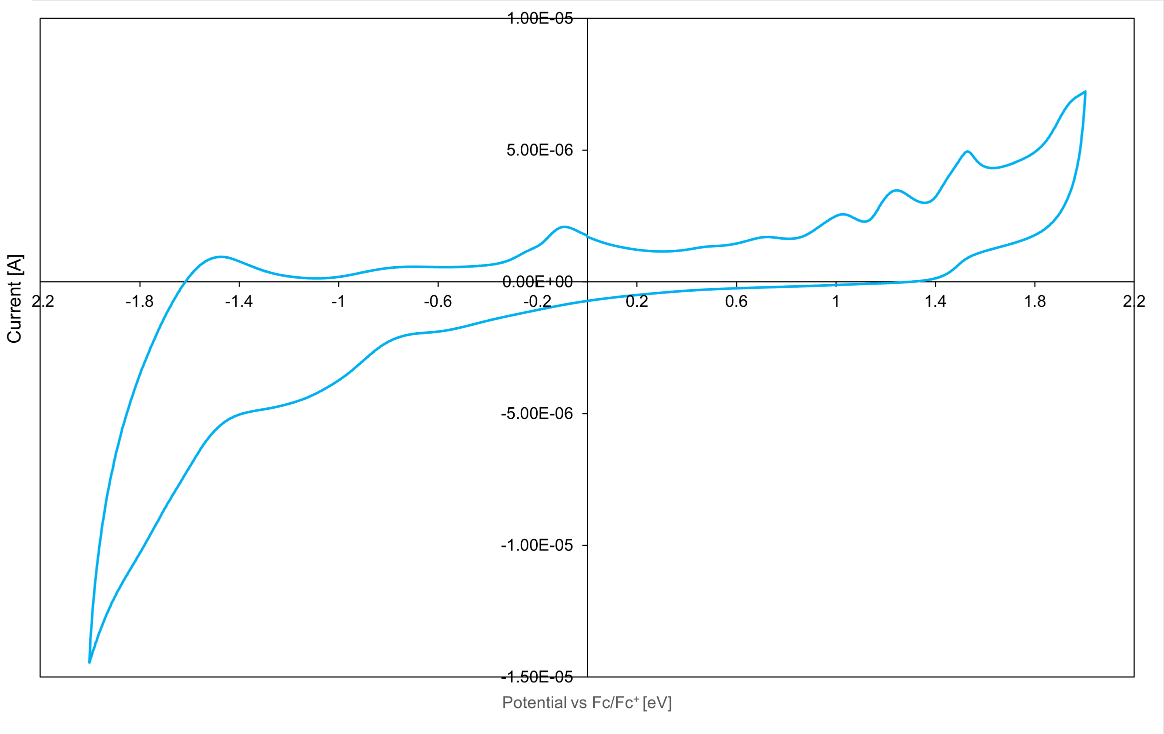

Cyclic voltammogram of **4** in CH_2Cl_2 (0.4 mM) at a scan rate of 100 mV/s at room temperature *vs* Fc/Fc^+, Pt/$[NBu_4][PF_6]$/Ag

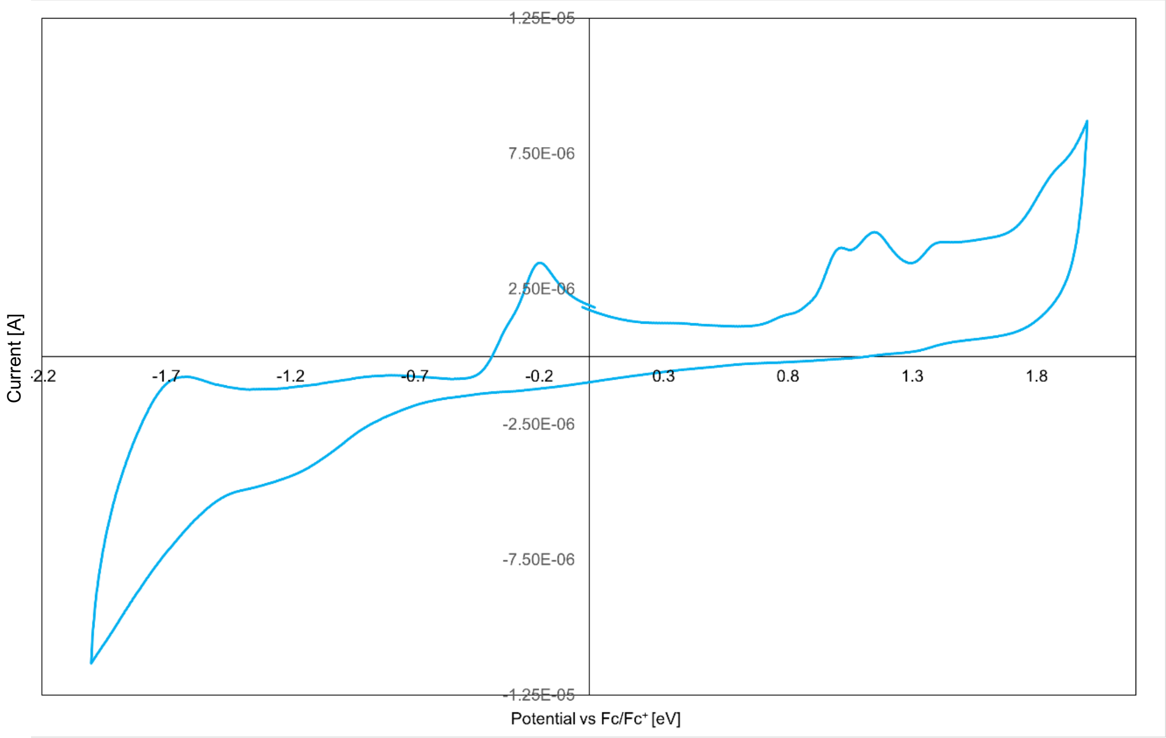

Cyclic voltammogram of **14** in CH_2Cl_2 (0.3 mM) at a scan rate of 100 mV/s at room temperature *vs* Fc/Fc^+, Pt/$[NBu_4][PF_6]$/Ag

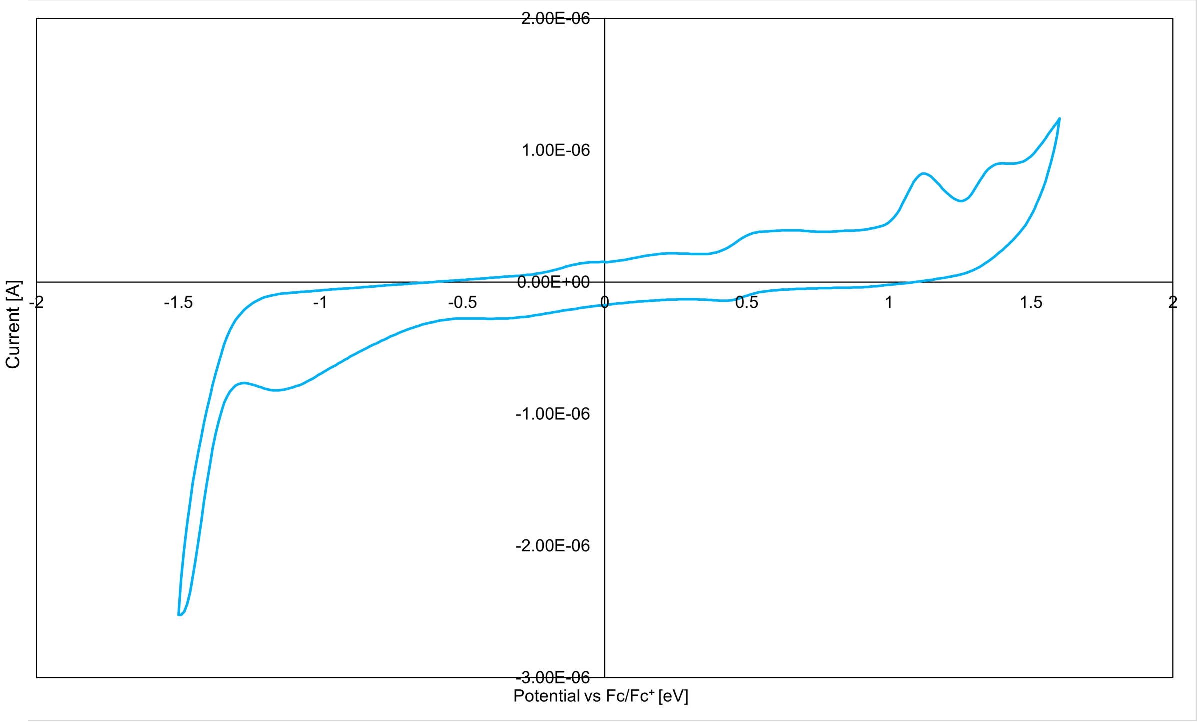

Cyclic voltammogram of **6** in CH_2Cl_2 (0.3 mM) at a scan rate of 100 mV/s at room temperature *vs* Fc/Fc^+, Pt/[NBu_4][PF_6]/Ag

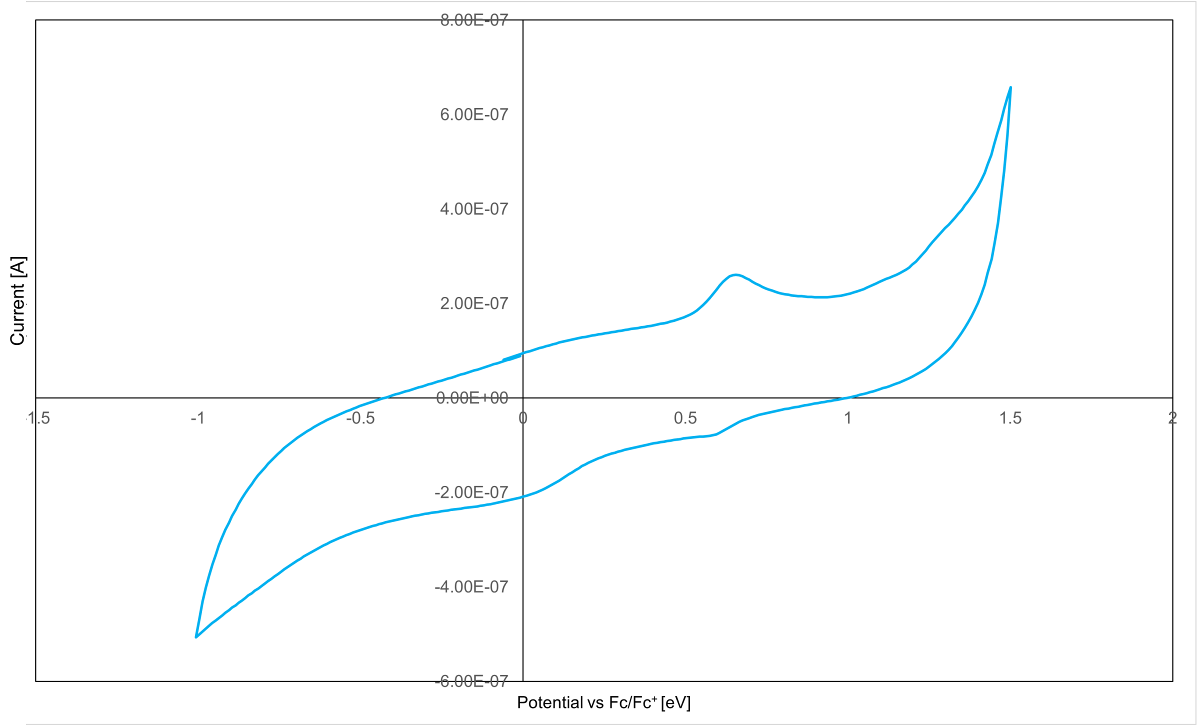

Cyclic voltammogram of **16** in CH_2Cl_2 (0.3 mM) at a scan rate of 100 mV/s at room temperature *vs* Fc/Fc^+, Pt/[NBu_4][PF_6]/Ag

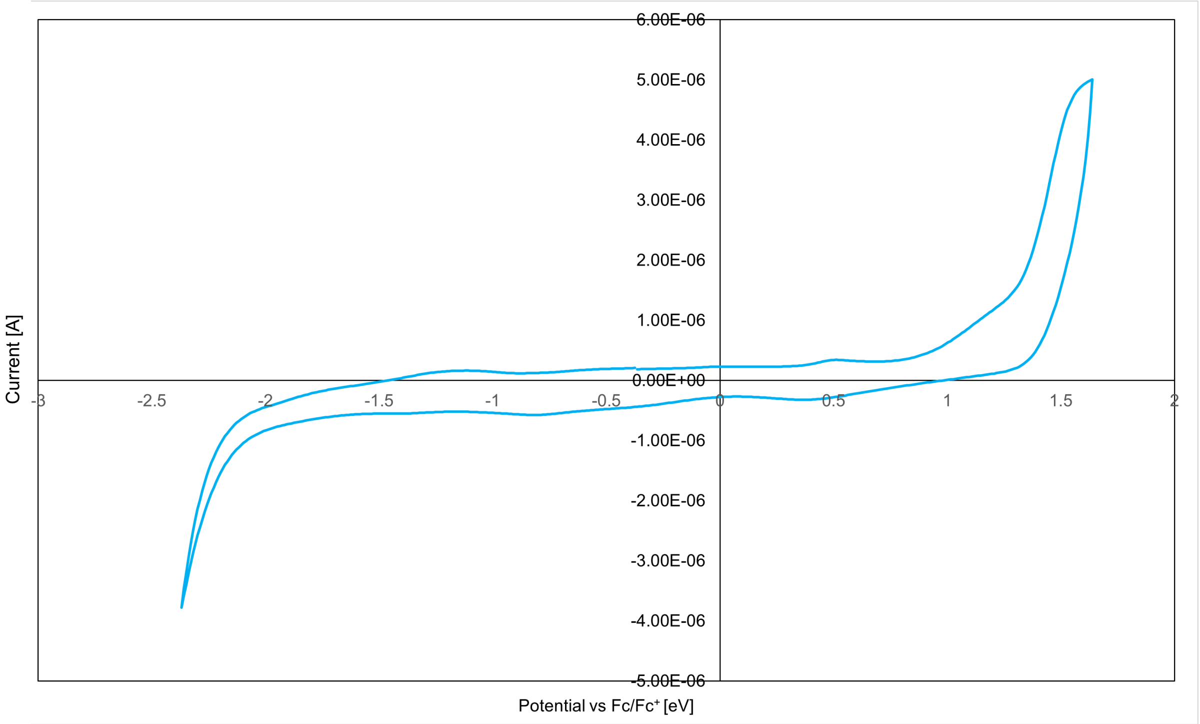

Cyclic voltammogram of **7** in CH_2Cl_2 (0.3 mM) at a scan rate of 100 mV/s at room temperature *vs* Fc/Fc^+, Pt/[NBu_4][PF_6]/Ag

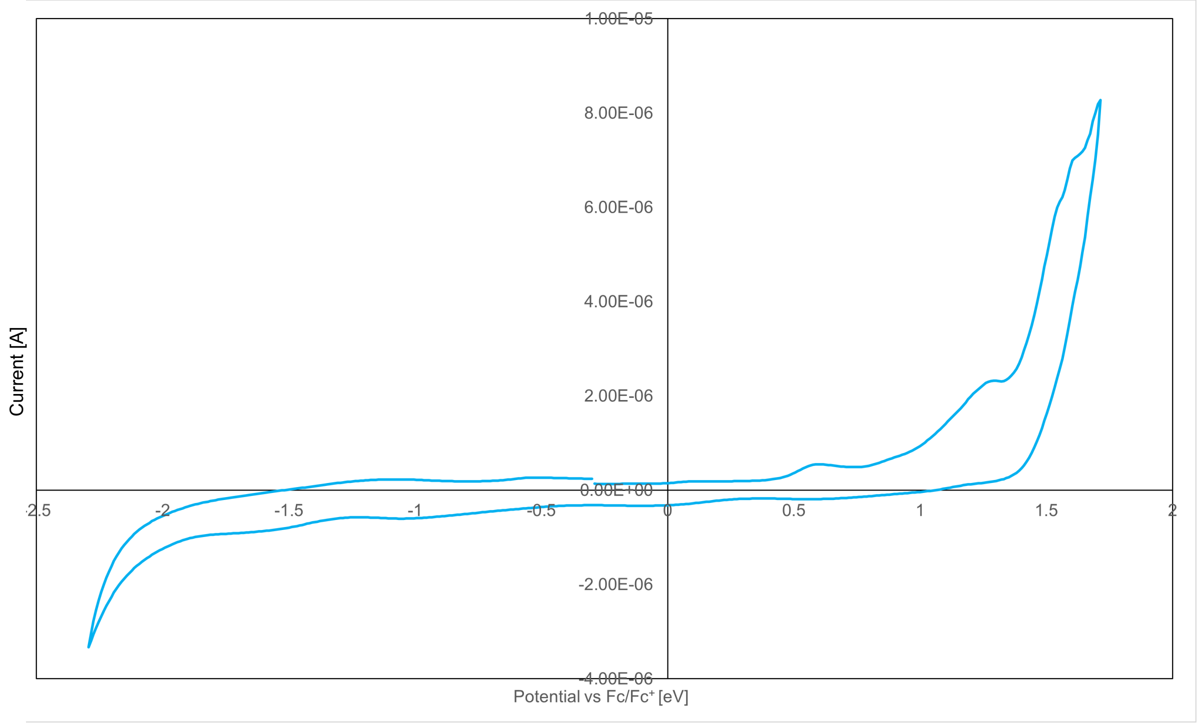

Cyclic voltammogram of **22a** in CH_2Cl_2 (0.2 mM) at a scan rate of 100 mV/s at room temperature *vs* Fc/Fc^+, Pt/[NBu_4][PF_6]/Ag

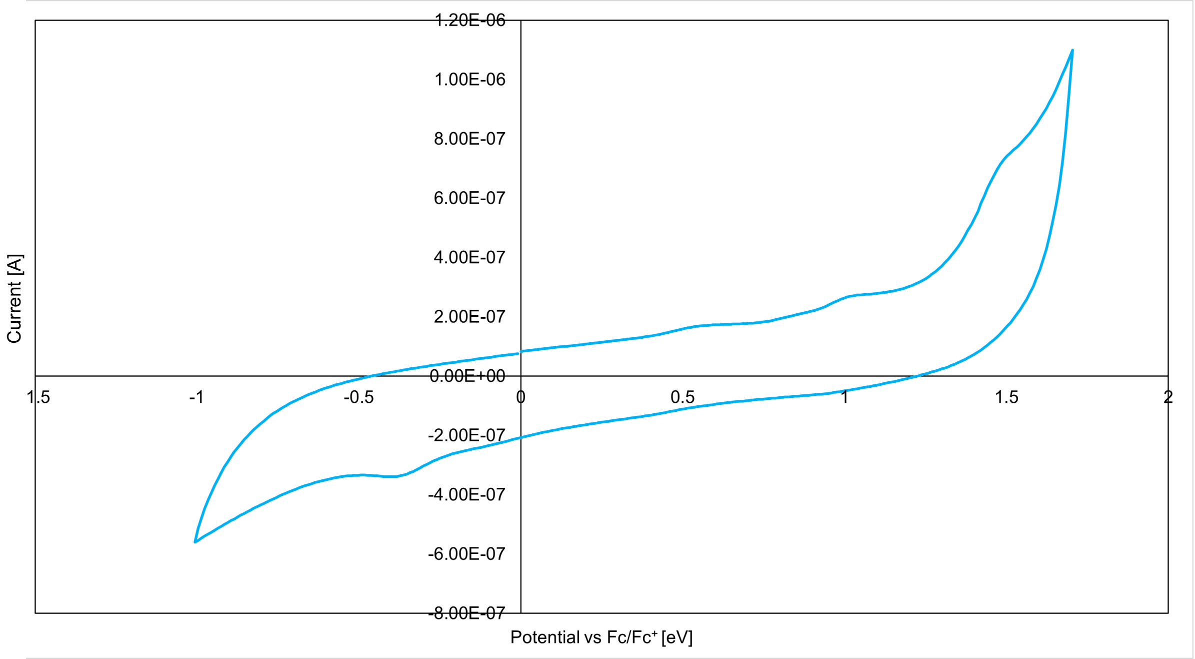

Cyclic voltammogram of **22b** in CH_2Cl_2 (0.2 mM) at a scan rate of 100 mV/s at room temperature *vs* Fc/Fc^+, Pt/[NBu$_4$][PF$_6$]/Ag

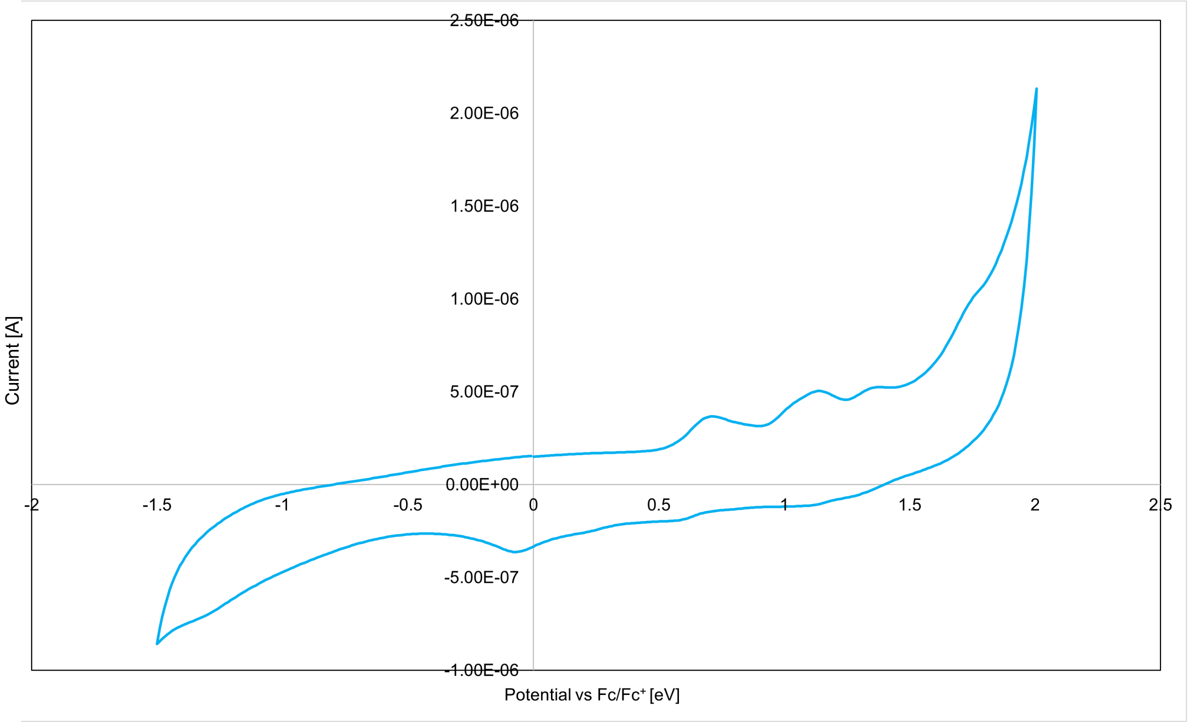

Cyclic voltammogram of **22c** in CH_2Cl_2 (0.2 mM) at a scan rate of 100 mV/s at room temperature *vs* Fc/Fc^+, Pt/[NBu$_4$][PF$_6$]/Ag

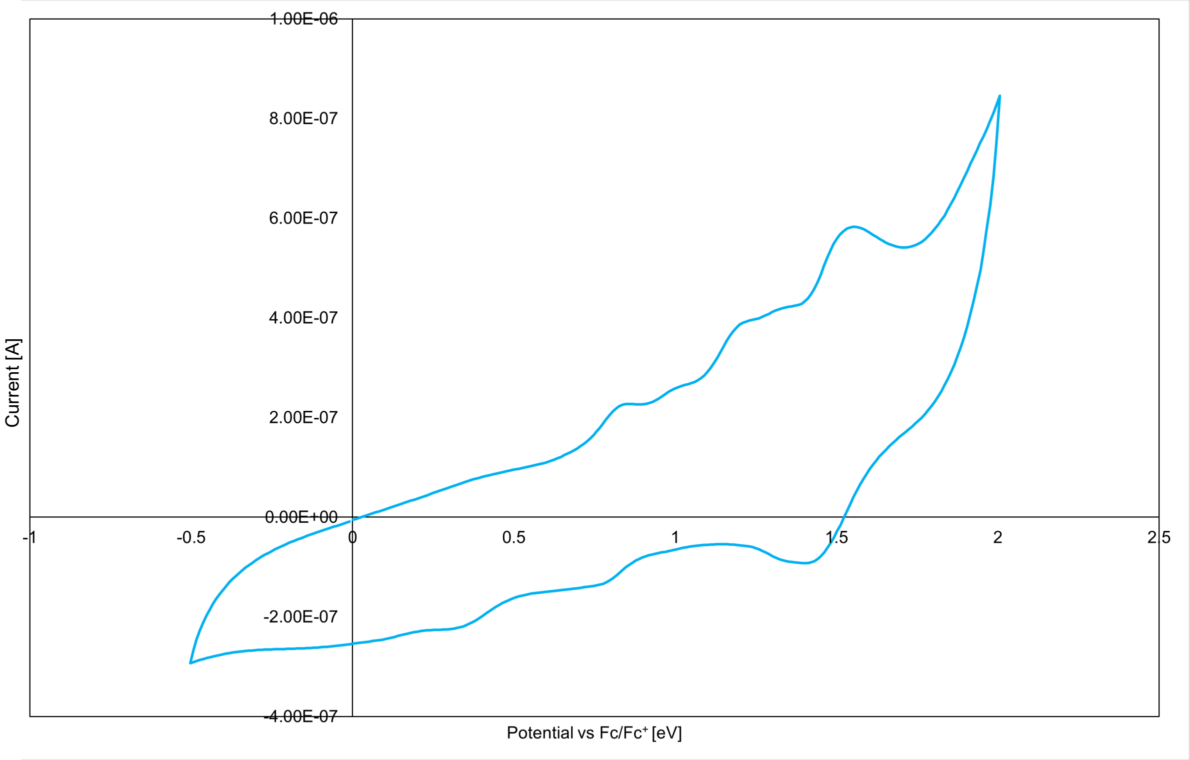

Cyclic voltammogram of **8a** in CH_2Cl_2 (0.2 mM) at a scan rate of 100 mV/s at room temperature *vs* Fc/Fc^+, Pt/[NBu_4][PF_6]/Ag

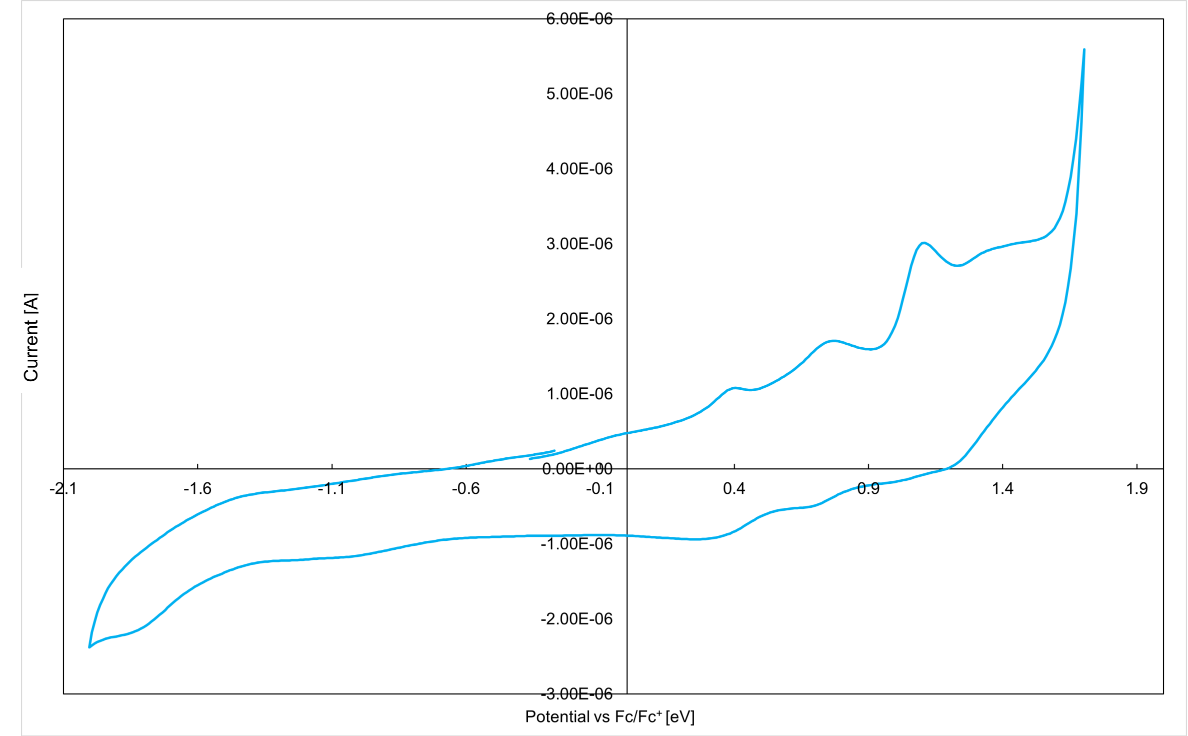

Cyclic voltammogram of **8b** in CH_2Cl_2 (0.2 mM) at a scan rate of 100 mV/s at room temperature *vs* Fc/Fc^+, Pt/[NBu_4][PF_6]/Ag

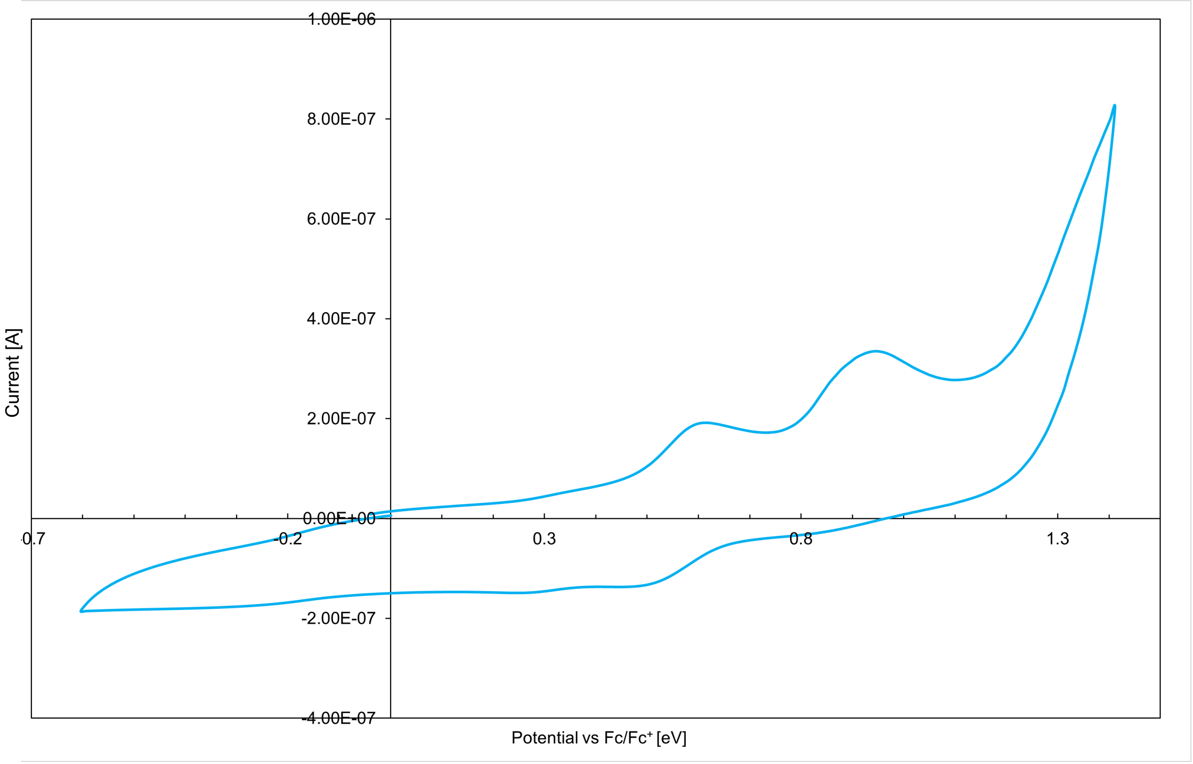

Cyclic voltammogram of **8c** in CH_2Cl_2 (0.2 mM) at a scan rate of 100 mV/s at room temperature *vs* Fc/Fc^+, Pt/[NBu_4][PF_6]/Ag

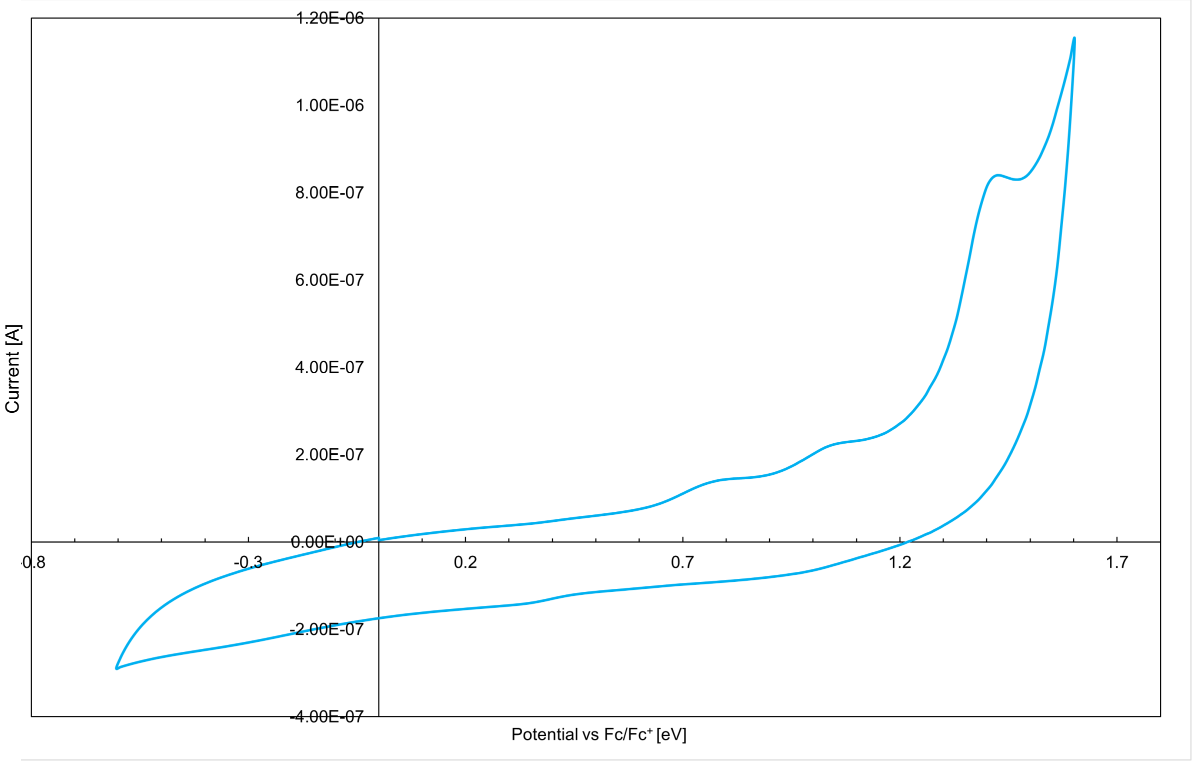

Cyclic voltammogram of **26** in CH_2Cl_2 (0.3 mM) at a scan rate of 100 mV/s at room temperature *vs* Fc/Fc^+, Pt/[NBu_4][PF_6]/Ag

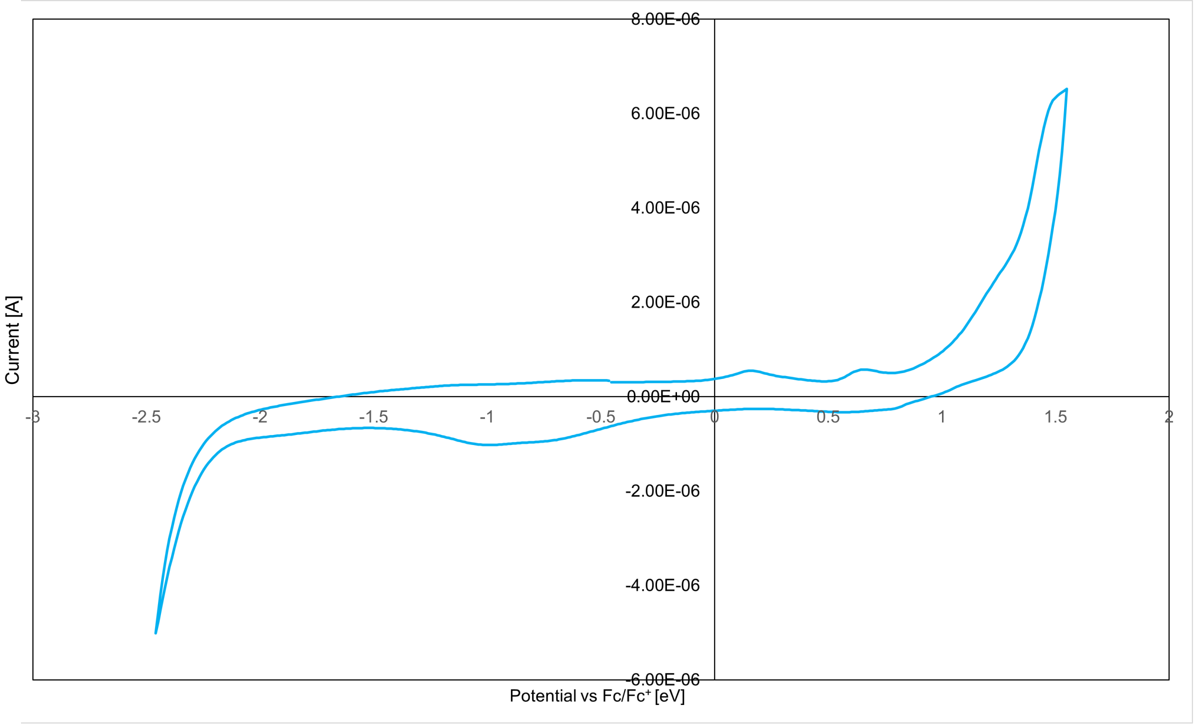

Cyclic voltammogram of **9** in CH_2Cl_2 (0.3 mM) at a scan rate of 100 mV/s at room temperature *vs* Fc/Fc^+, Pt/$[NBu_4][PF_6]$/Ag

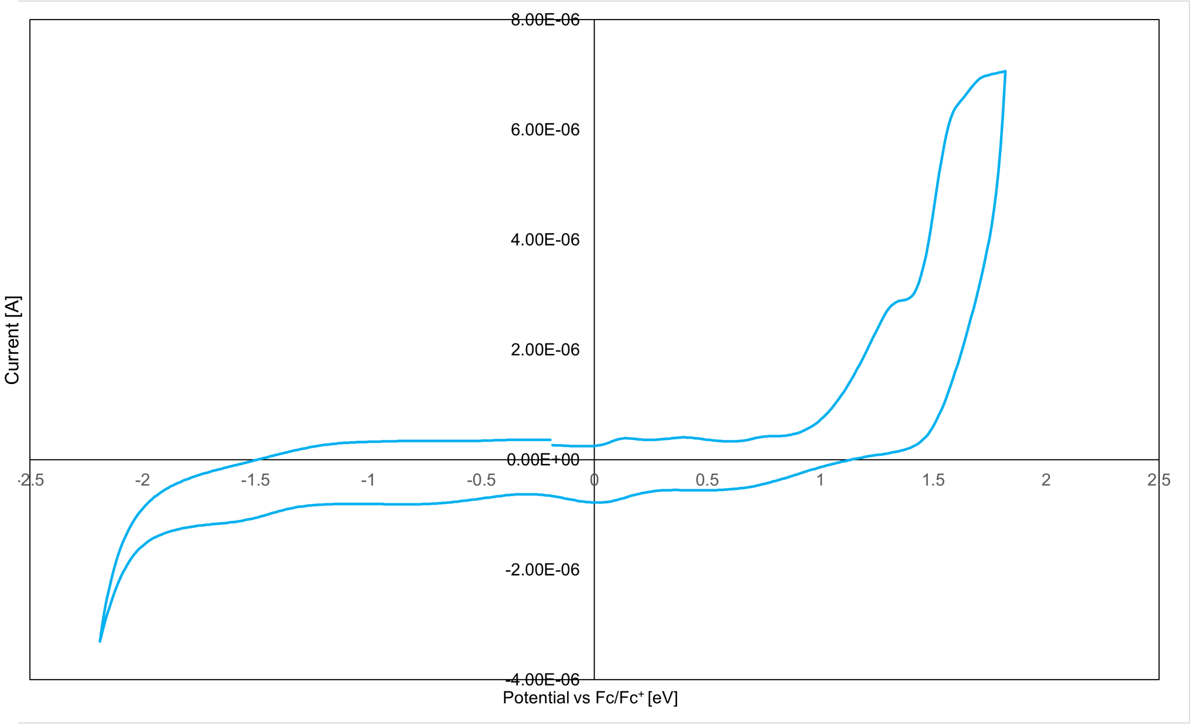

3.7. EPR measurement

EPR measurements at X-band (9.38 GHz) were carried out using a Bruker ELEXSYS E580 CW EPR spectrometer equipped with an Oxford Instruments helium cryostat (ESR900) and a MercuryiTC temperature controller. The spectral analysis was performed using MATLAB 9.10.0 (R2021a), the Optimization toolbox (version 9.1), and the EasySpin 6.0.0 toolbox.[SI12]

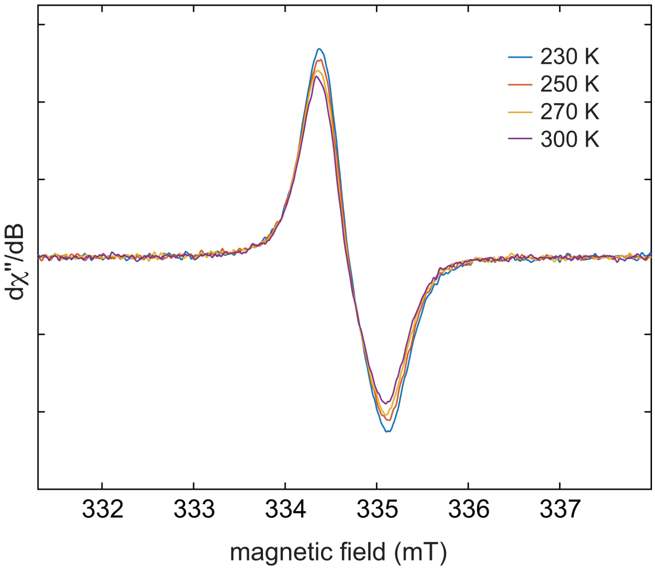

Figure S1. Variable temperature X-band EPR spectra of solid **7** between 230 and 300 K showing a decrease in signal intensity with increasing temperature (consistent with a S = 1/2 impurity).

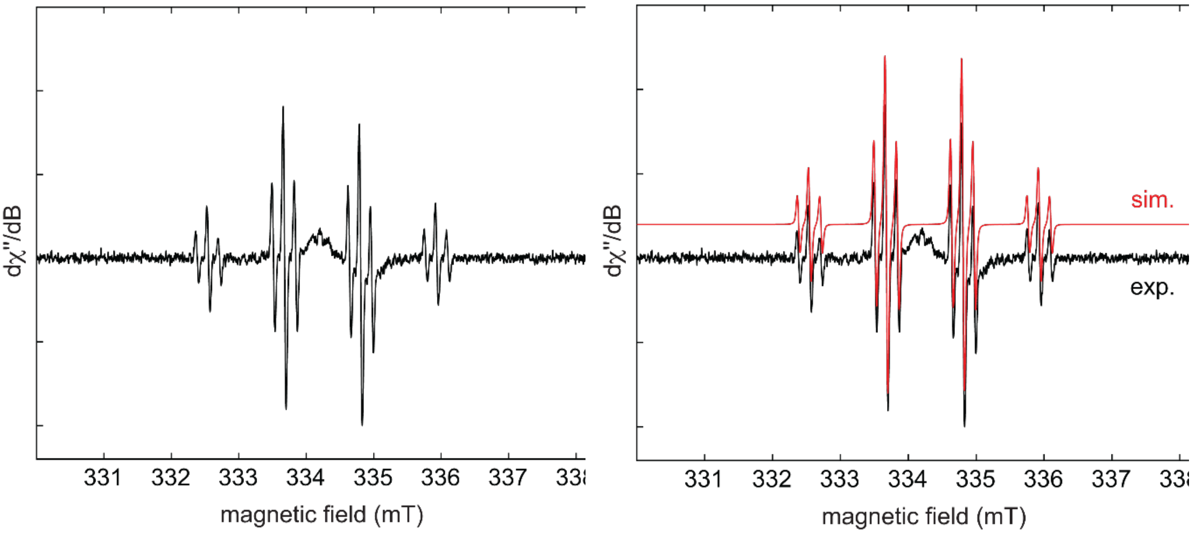

Figure S2. Experimental and simulated X-band EPR spectra of **7** in tetrahydrofuran at room temperature. The EPR signal is centered around g = 2.0047 with proton hyperfine couplings of $a(^1H)$ = 32 MHz (11 G, 3H) and 5 MHz (2 G, 2H).

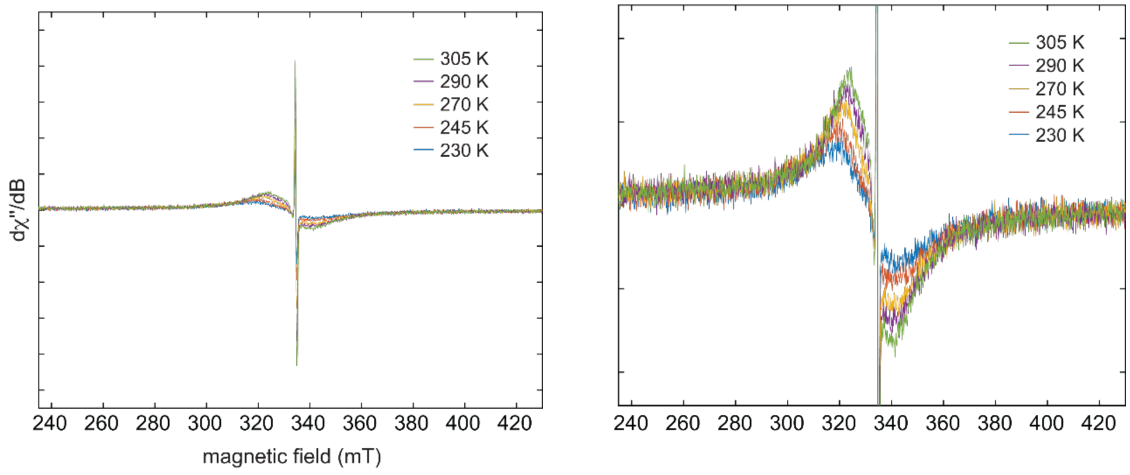

Figure S3. Variable temperature X-band EPR spectra of **8c** in thf between 230 and 305 K and zoomed in view of the broad signal on the right.

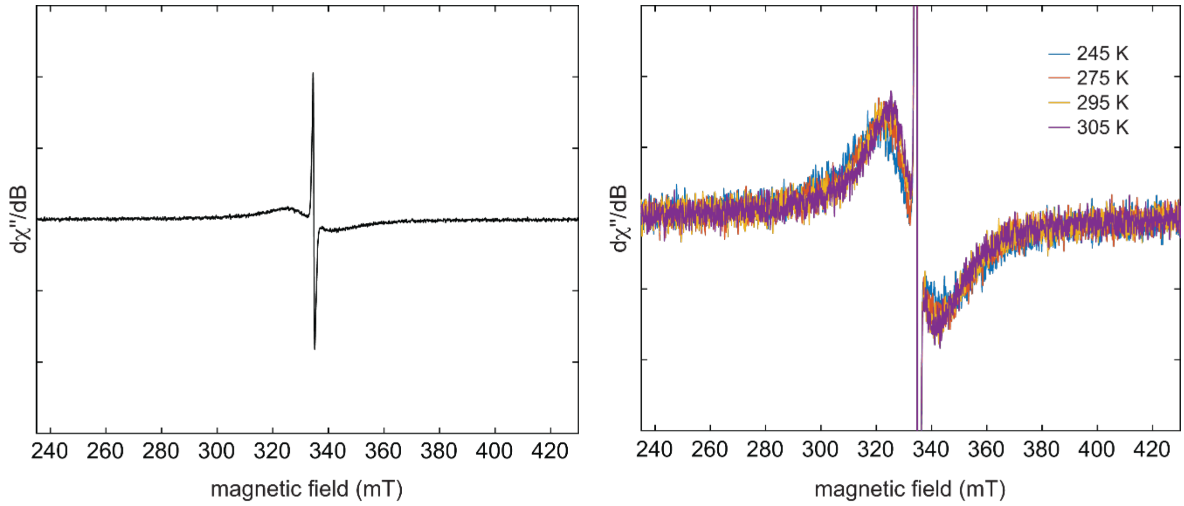

Figure S4. X-band EPR spectrum of solid **8c** at room temperature and expanded view of the temperature dependence of the broad signal between 245 and 305 K on the right.

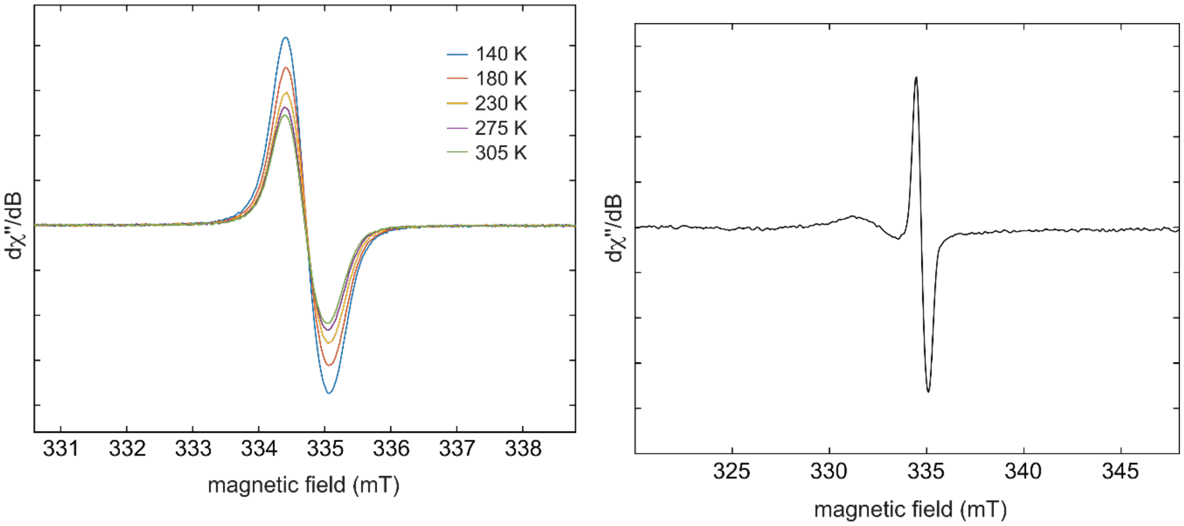

Figure S5. Variable temperature X-band EPR spectra of solid **8b** between 140 and 305 K (*left*) and spectrum in thf at 300 K (*right*). The broad signal (likely corresponding to a thermally populated triplet state) is weaker than in sample **8c**.

3.8. Quantum chemical calculations

Density functional theory (DFT) and time-dependent density functional theory (TDDFT) methods were applied as implemented in the TURBOMOLE[SI14] program. All calculations were carried out using the PBE0[SI15] functional, the def2-TZVP[SI16] basis set and fine quadrature grids (size 5). For geometry optimizations, tight convergence criteria were employed (SCF energy: 10^{-8} hartree, gradient: 10^{-4} hartree/bohr and inclusion of derivatives of quadrature weights). Vibrational frequencies were computed in order to ensure that all structures represent minima on the respective potential energy surface. Contour plots of molecular orbitals and spin densities were generated with values of ±0.04 (bohr)$^{-3/2}$ and ±0.0036 (bohr)$^{-3}$, respectively.

Complete active space self-consistent field (CASSCF) calculations were performed as implemented in the ORCA[SI17] program using the def2-TZVP basis set.

Atomic coordinates can be found in xyz-format in the deposited xyz-file.

3.9. Crystallographic data

The crystallographic data acquisition of **S2a**, **S2b** and **20a** was carried out using a *Bruker* SMART CCD area detector diffractometer equipped with graphite-monochromated Mo K_α radiation (λ= 0.71073 Å) at 130 K. The presented X-ray single crystal data of **4**, **12** and **21b** were recorded using a *Bruker Venture D8 diffractometer* – for the measurement of **4** the diffractometer was equiped with a Cu K_α (λ=1.54178 Å) μ-source, for **12** and **21b** a Mo K_α (λ= 0.71073 Å) μ-source was used. For all three measurements the radiation was monochromated using an *Incoatec* multilayer Montel optics, a Photon III area detector was applied and the crystal was kept at 120 K.

The obtained data were integrated with SAINT, a multi-scan absorption correction was carried out by SADABS and the structure solution by direct methods and the refinement of the structures using full-matrix least squares method based on F^2 were achieved in SHELX – all three software are parts of the Bruker APEX III package. [Sheldrick, G. M. SHELXT - Integrated space-group and crystal-structure determination (2015). Acta Cryst. A71, 3-8. DOI: 10.1107/S2053273314026370; Sheldrick, George M. (2015): Crystal structure refinement with SHELXL. In: Acta Cryst. C C71 (1), S. 3-8. DOI: 10.1107/S2053229614024218.] All non-hydrogen-atoms were refined anisotropically and the hydrogen atom positions were refined at idealized positions riding on the carbon atoms with isotropic displacement parameters $U_{iso}(H)$=1.2 $U_{eq}(C)$ resp. 1.5 $U_{eq}(-CH_3)$ and C-H bond lengths of 0.93-0.96 Å. All CH_3 hydrogen atoms were allowed to rotate but not to tip.

Crystallographic data have been deposited at the Cambridge Crystallographic Data Centre (CCDC) assigned to the deposition numbers 2121375-2121377 and 2127274-2127276. Copies are available free of charge via www.ccdc.cam.ac.uk.

S2a

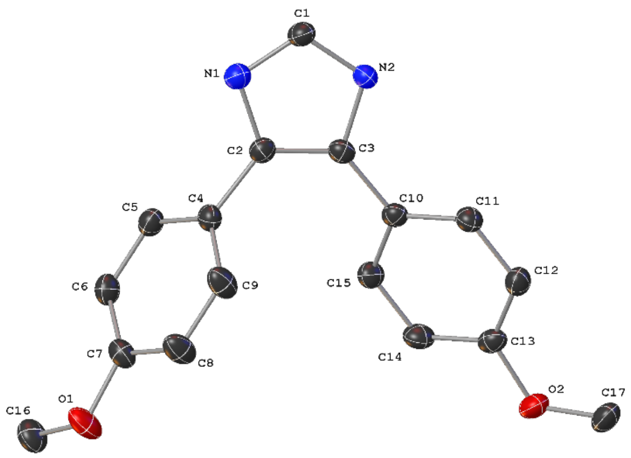

($C_{17}H_{16}N_2O_2$), M_r = 280.32 Da, colourless block, size: 0.45 x 0.44 x 0.20 mm³, monoclinic space group $P\,1\,2_1/c\,1$ with Z = 4, a = 11.680(2) Å, b = 10.029(2) Å, c = 12.408(3) Å, β = 103.032(4)°, V = 1416.1(5)Å^3, D_c = 1.315 mg/m³, μ = 0.087 mm^{-1}, F(000) = 592, 1.790° ≤ θ ≤ 25.937°, reflections collected: 7141, independent reflections: 2700, R_{int} = 0.0296, refinement converged at R1 = 0.0393 [I>2σ(I)], wR2 = 0.1069 [all data], min./max. ΔF: -0.224 eÅ^{-3} / 0.213 eÅ^{-3}, CCDC-No.: 2121376.

CCDC-No.	2121376	
Empirical formula	C17 H16 N2 O2	
Formula weight	280.32	
Temperature	130(2) K	
Wavelength	0.71073 Å	
Crystal system	Monoclinic	
Space group	P 1 21/c 1	
Unit cell dimensions	a = 11.680(2) Å	α = 90°
	b = 10.029(2) Å	β = 103.032(4)°
	c = 12.408(3) Å	γ = 90°
Volume	1416.1(5) Å^3	
Z	4	
Density (calculated)	1.315 Mg/m^3	
Absorption coefficient	0.087 mm^{-1}	
F(000)	592	
Crystal size	0.45 x 0.44 x 0.20 mm^3	
Theta range for data collection	1.790 to 25.937°.	
Index ranges	-14<=h<=13, -12<=k<=11, -15<=l<=13	
Reflections collected	7141	

Independent reflections	2700 [R(int) = 0.0296]
Completeness to theta = 25.242°	98.2 %
Absorption correction	Semi-empirical from equivalents
Max. and min. transmission	0.7453 and 0.6666
Refinement method	Full-matrix least-squares on F^2
Data / restraints / parameters	2700 / 0 / 192
Goodness-of-fit on F^2	1.059
Final R indices [I>2sigma(I)]	R1 = 0.0393, wR2 = 0.0990
R indices (all data)	R1 = 0.0521, wR2 = 0.1069

Largest diff. peak and hole 0.213 and -0.224 e.Å^{-3}

S2b

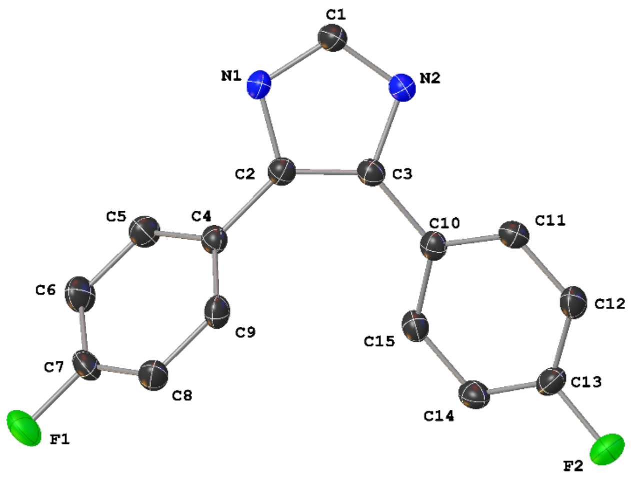

($C_{15}H_{10}F_2N_2$), M_r = 256.25 Da, colourless block, size: 0.35 x 0.34 x 0.28 mm³, orthorhombic space group $P2_12_12_1$ with Z = 4, a = 6.3620(8) Å, b = 9.5812(12) Å, c = 20.191(2) Å, V = 1230.7(3) Å³, D_c = 1.383 mg/m³, μ = 0.104 mm^{-1}, F(000) = 528, 2.017° ≤ θ ≤ 25.969°, reflections collected: 11334, independent reflections: 2407, R_{int} = 0.0263, refinement converged at R1 = 0.0275 [I>2σ(I)], wR2 = 0.0681 [all data], min./max. ΔF: -0.167 eÅ^{-3} / 0.174 eÅ^{-3}, CCDC-No.: 2121375.

CCDC-No	2121375
Empirical formula	C15 H10 F2 N2
Formula weight	256.25
Temperature	130(2) K
Wavelength	0.71073 Å

Crystal system	Orthorhombic	
Space group	$P2_12_12_1$	
Unit cell dimensions	a = 6.3620(8) Å	α = 90°
	b = 9.5812(12) Å	β = 90°
	c = 20.191(2) Å	γ = 90°
Volume	1230.7(3) $Å^3$	
Z	4	
Density (calculated)	1.383 Mg/m^3	
Absorption coefficient	0.104 mm^{-1}	
F(000)	528	
Crystal size	0.35 x 0.34 x 0.28 mm^3	
Theta range for data collection	2.017 to 25.969°.	
Index ranges	-7<=h<=7, -11<=k<=11, -24<=l<=24	
Reflections collected	11334	
Independent reflections	2407 [R(int) = 0.0263]	
Completeness to theta = 25.242°	100.0 %	
Absorption correction	Semi-empirical from equivalents	
Max. and min. transmission	0.7453 and 0.6836	
Refinement method	Full-matrix least-squares on F^2	
Data / restraints / parameters	2407 / 0 / 172	
Goodness-of-fit on F^2	1.047	
Final R indices [I>2sigma(I)]	R1 = 0.0275, wR2 = 0.0670	
R indices (all data)	R1 = 0.0292, wR2 = 0.0681	
Absolute structure parameter	0.1(2)	
Largest diff. peak and hole	0.174 and -0.167 $e/Å^{-3}$	

20a

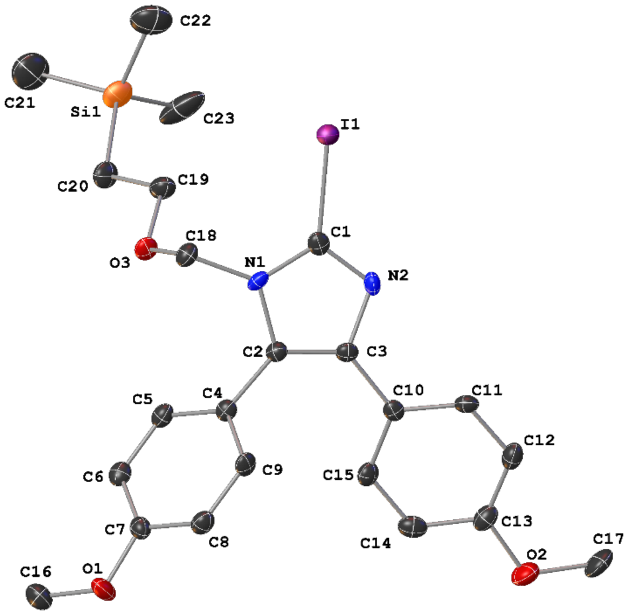

($C_{23}H_{30}IN_2O_3Si$), M_r = 537.48 Da, colourless needle, size: 0.35 x 0.12 x 0.11 mm³, monoclinic space group $C\ 1\ 2/c\ 1$ with Z = 8, a = 25.510(11) Å, b = 7.672(3) Å, c = 24.580(10) Å, β = 92.441(8)°, V = 4806(3) Å³, D_c = 1.486 mg/m³, μ = 1.408 mm^{-1}, F(000) = 2184, 1.598° ≤ θ ≤ 25.747°, reflections collected: 12982, independent reflections: 4595, R_{int} = 0.0809, refinement converged at R1 = 0.0454 [I>2σ(I)], wR2 = 0.0995 [all data], min./max. ΔF: -1.069 eÅ^{-3} / 1.151 eÅ^{-3}, CCDC-No.: 2121377.

CCDC-No.	2121377	
Empirical formula	C23 H30 I N2 O3 Si	
Formula weight	537.48	
Temperature	130(2) K	
Wavelength	0.71073 Å	
Crystal system	Monoclinic	
Space group	C 1 2/c 1	
Unit cell dimensions	a = 25.510(11) Å	α = 90°
	b = 7.672(3) Å	β = 92.441(8)°
	c = 24.580(10) Å	γ = 90°
Volume	4806(3) Å^3	
Z	8	
Density (calculated)	1.486 Mg/m^3	
Absorption coefficient	1.408 mm^{-1}	
F(000)	2184	
Crystal size	0.35 x 0.12 x 0.11 mm^3	
Theta range for data collection	1.598 to 25.747°.	

Index ranges	-30<=h<=24, -9<=k<=9, -29<=l<=30
Reflections collected	12982
Independent reflections	4595 [R(int) = 0.0809]
Completeness to theta = 25.242°	100.0 %
Absorption correction	Semi-empirical from equivalents
Max. and min. transmission	0.7453 and 0.5618
Refinement method	Full-matrix least-squares on F^2
Data / restraints / parameters	4595 / 0 / 276
Goodness-of-fit on F^2	0.943
Final R indices [I>2sigma(I)]	R1 = 0.0454, wR2 = 0.0896
R indices (all data)	R1 = 0.0738, wR2 = 0.0995
Largest diff. peak and hole	1.151 and -1.069 e.Å^{-3}

12

($C_{56}H_{66}N_4O_8$), M_r = 923.12 Da, yellow block, size: 0.26 x 0.20 x 0.18 mm³, monoclinic space group $P2_1/c$ with Z = 2, a = 12.2974(4) Å, b = 13.8459(4) Å, c = 16.2659(5) Å, β = 108.2310(10)°, V = 2630.55(14) Å³, D_c = 1.165 mg/m³, μ = 0.078 mm^{-1}, F(000) = 988, 1.978° ≤ θ ≤ 28.306°, reflections collected: 121191, independent reflections: 6536, R_{int} = 0.0698, refinement converged at R1 = 0.0453 [I>2σ(I)], wR2 = 0.1151 [all data], min./max. ΔF: -0.298 eÅ^{-3} / 0.495 eÅ^{-3}, **CCDC-No.: 2127276.**

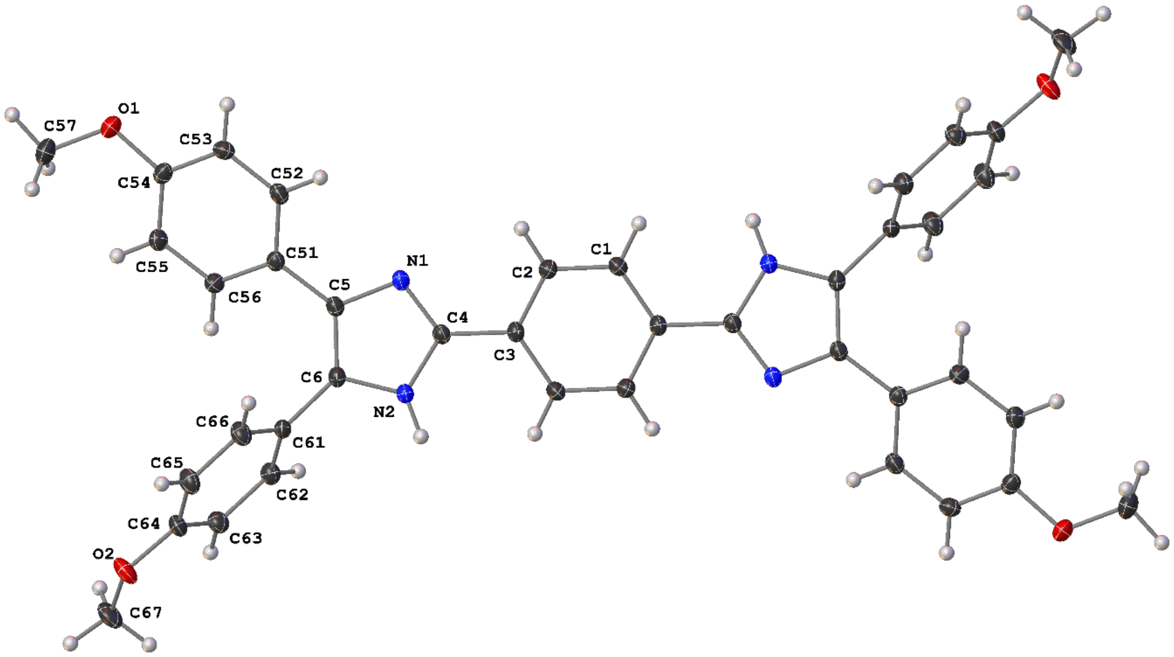

Identification code	hp_0023n_0m_a_sq	
Empirical formula	C56 H66 N4 O8	
Formula weight	923.12	
Temperature	120(2) K	
Wavelength	0.71073 Å	
Crystal system	Monoclinic	
Space group	$P2_1/c$	
Unit cell dimensions	a = 12.2974(4) Å	α = 90°
b = 13.8459(4) Å	β = 108.2310(10)°	
c = 16.2659(5) Å	γ = 90°	
Volume	2630.55(14) Å^3	
Z	2	

Density (calculated)	1.165 Mg/m^3
Absorption coefficient	0.078 mm^{-1}
F(000)	988
Crystal size	0.260 x 0.200 x 0.180 mm^3
Theta range for data collection	1.975 to 28.306°.
Index ranges	-16<=h<=16, -18<=k<=18, -21<=l<=21
Reflections collected	121191
Independent reflections	6536 [R(int) = 0.0698]
Completeness to theta = 25.242°	99.8 %
Absorption correction	Semi-empirical from equivalents
Refinement method	Full-matrix least-squares on F^2
Data / restraints / parameters	6536 / 0 / 313
Goodness-of-fit on F^2	1.025
Final R indices [I>2sigma(I)]	R1 = 0.0453, wR2 = 0.1062
R indices (all data)	R1 = 0.0572, wR2 = 0.1151
Largest diff. peak and hole	0.495 and -0.298 e.Å^{-3}

4

($C_{40}H_{32}N_4O_4$), M_r = 632.69 Da, brown needle, size: 0.28 x 0.12 x 0.06 mm³, monoclinic space group $P2_1/c$ with Z = 2, a = 11.5413(5) Å, b = 17.2726(7) Å, c = 8.0531(3) Å, β = 101.238(2)°, V = 1574.59(11) Å³, D_c = 1.334 mg/m³, μ = 0.702 mm^{-1}, F(000) = 664, 3.905° ≤ θ ≤ 79.376°, reflections collected: 99064, independent reflections: 3355, R_{int} = 0.0711, refinement converged at R1 = 0.0497 [I>2σ(I)], wR2 = 0.1338 [all data], min./max. ΔF: -0.324 eÅ^{-3} / 0.701 eÅ^{-3}, CCDC-No.: 2127274.

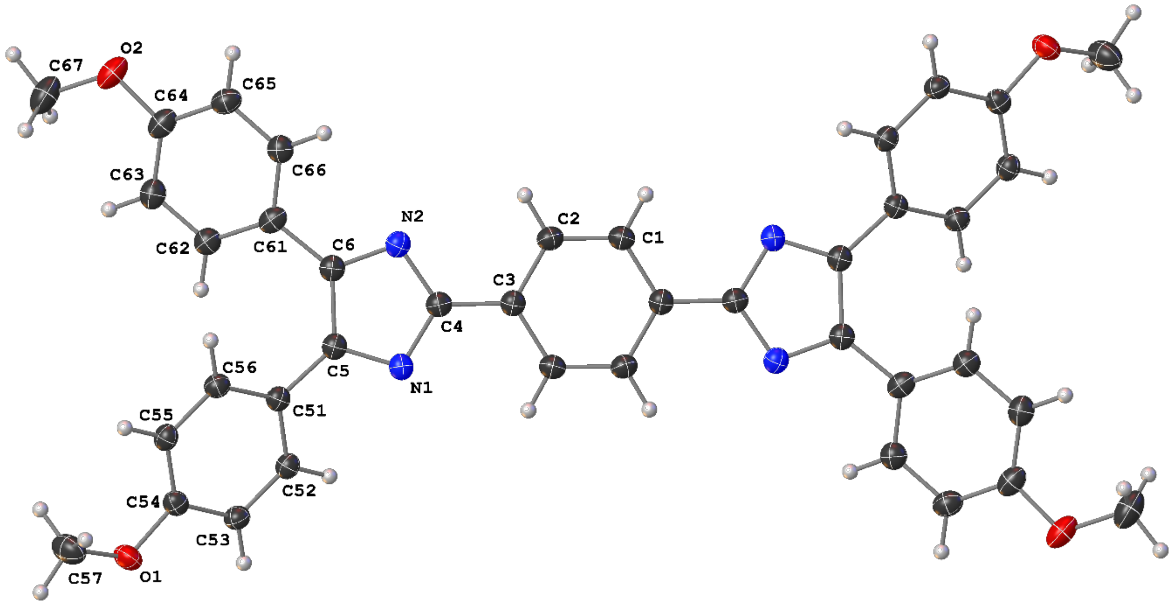

CCDC-No.	2127274	
Empirical formula	C40 H32 N4 O4	
Formula weight	632.69	
Temperature	120(2) K	
Wavelength	1.54178 Å	
Crystal system	Monoclinic	
Space group	$P2_1/c$	
Unit cell dimensions	a = 11.5413(5) Å	α= 90°
	b = 17.2726(7) Å	β= 101.238(2)°
	c = 8.0531(3) Å	γ = 90°
Volume	1574.59(11) Å^3	
Z	2	
Density (calculated)	1.334 Mg/m^3	

Absorption coefficient	0.702 mm^{-1}
F(000)	664
Crystal size	0.280 x 0.120 x 0.060 mm^3
Theta range for data collection	3.905 to 79.376°.
Index ranges	-12<=h<=14, -21<=k<=21, -9<=l<=9
Reflections collected	99064
Independent reflections	3355 [R(int) = 0.0711]
Completeness to theta = 67.679°	99.6 %
Refinement method	Full-matrix least-squares on F^2
Data / restraints / parameters	3355 / 0 / 220
Goodness-of-fit on F^2	1.061
Final R indices [I>2sigma(I)]	R1 = 0.0497, wR2 = 0.1247
R indices (all data)	R1 = 0.0571, wR2 = 0.1338
Extinction coefficient	0.0062(10)
Largest diff. peak and hole	0.701 and -0.324 e.$Å^{-3}$

21b

($C_{50}H_{50}Br_2N_4O_2SSi_2$), M_r = 987.00 Da, colourless block, size: 0.26 x 0.20 x 0.18 mm³, triclinic space group $P\bar{1}$ with Z = 2, a = 8.8434(4) Å, b = 15.2893(5) Å, c = 18.5618(9) Å, α = 97.318(2)°, β = 93.899(2)°, γ = 104.456(2)°, V = 2397.43(18) Å³, D_c = 1.367 mg/m³, μ = 1.827 mm^{-1}, F(000) = 1016, 2.224° ≤ θ ≤ 27.494°, reflections collected: 60407, independent reflections: 10994, R_{int} = 0.0566, refinement converged at R1 = 0.0287 [I>2σ(I)], wR2 = 0.0680 [all data], min./max. ΔF: -0.510 eÅ^{-3} / 0.436 eÅ^{-3}, CCDC-No.: 2127275.

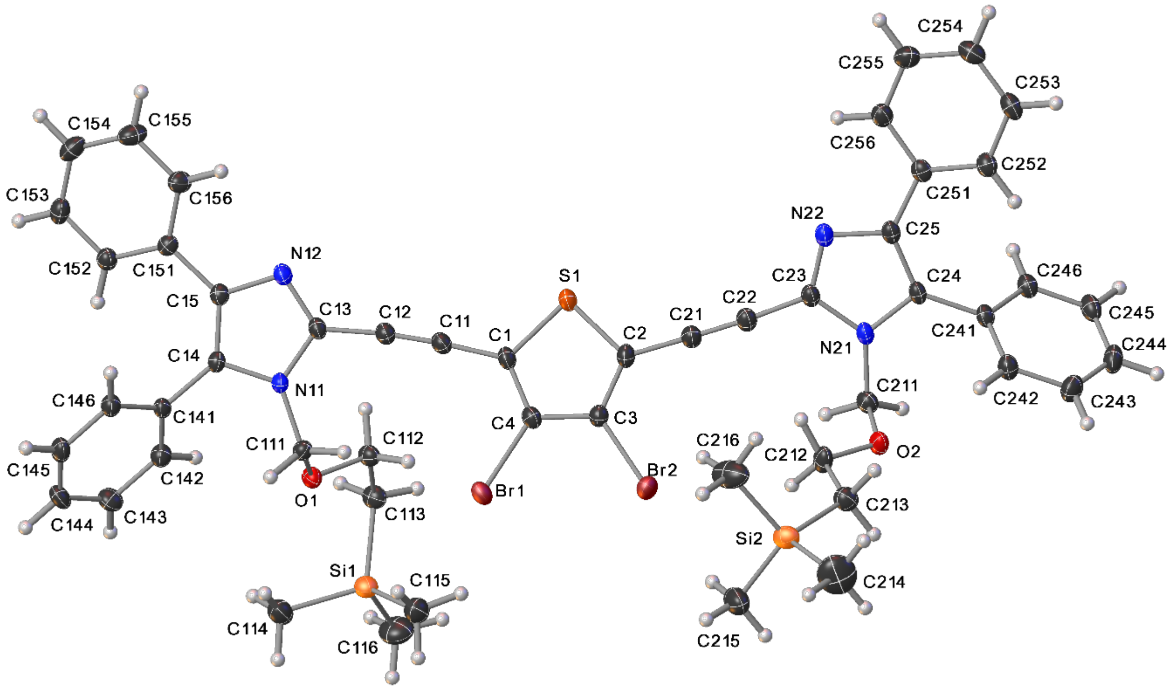

CCDC-No.	2127275	
Empirical formula	C50 H50 Br2 N4 O2 S Si2	
Formula weight	987.00	
Temperature	120(2) K	
Wavelength	0.71073 Å	
Crystal system	Triclinic	
Space group	P-1	
Unit cell dimensions	a = 8.8434(4) Å	α = 97.318(2)°
	b = 15.2893(5) Å	β = 93.899(2)°
	c = 18.5618(9) Å	γ = 104.456(2)°

Volume	2397.43(18) Å^3
Z	2
Density (calculated)	1.367 Mg/m^3
Absorption coefficient	1.827 mm^{-1}
F(000)	1016
Crystal size	0.26 x 0.20 x 0.18 mm^3
Theta range for data collection	2.224 to 27.494°.
Index ranges	-11<=h<=11, -19<=k<=19, -24<=l<=24
Reflections collected	60407
Independent reflections	10994 [R(int) = 0.0566]
Completeness to theta = 25.242°	99.9 %
Absorption correction	Semi-empirical from equivalents
Max. and min. transmission	0.7456 and 0.5667
Refinement method	Full-matrix least-squares on F^2
Data / restraints / parameters	10994 / 0 / 556
Goodness-of-fit on F^2	1.005
Final R indices [I>2sigma(I)]	R1 = 0.0287, wR2 = 0.0621
R indices (all data)	R1 = 0.0422, wR2 = 0.0680
Largest diff. peak and hole	0.436 and -0.510 e.Å^{-3}

4. Reference

[SI1] E. Ay, S. Furukawa, E. Nakamura, *Org. Chem. Front.* **2014**, *1*, 988.

[SI2] N. Fridman, M. Kaftory, Y. Eichen, S. Speiser, *Journal of Photochemistry and Photobiology A: Chemistry* **2007**, *188*, 25.

[SI3] J. Meng, M. Gao, H. Lv, X. Zhang, *Organic letters* **2015**, *17*, 1842.

[SI4] W. Liu, K. Bensdorf, M. Proetto, U. Abram, A. Hagenbach, R. Gust, *Journal of medicinal chemistry* **2011**, *54*, 8605.

[SI5] K. C. Majumdar, S. Mondal, T. Ghosh, *Molecular Crystals and Liquid Crystals* **2010**, *524*, 17.

[SI6] a) P. Oechsle, U. Flörke, H. Egold, J. Paradies, *Chemistry (Weinheim an der Bergstrasse, Germany)* **2016**, *22*, 18559. b) P. Oechsle, P. Hou, U. Flörke, J. Paradies, *Adv. Synth. Catal.* **2016**, *358*, 3770.

[SI7] Y. Sakaino, H. Kakisawa, T. Kusumi, K. Maeda, *J. Org. Chem.* **1979**, *44:8*, 1241

[SI8] L. Biniek, B. C. Schroeder, J. E. Donaghey, N. Yaacobi-Gross, R. S. Ashraf, Y. W. Soon, C. B. Nielsen, J. R. Durrant, T. D. Anthopoulos, I. McCulloch, *Macromolecules* **2013**, *46*, 727.

[SI9] K. Sonogashira, Y. Tohda, N. Hagihara, Tetrahedron Lett. **1975**, *50*, 4467-4470.

[SI10] V. Guilarte, M. A. Fernández-Rodríguez, P. García-García, E. Hernando, R. Sanz, *Organic letters* **2011**, *13*, 5100.

[SI11] M. I. Cherkashin, M. G. Chauser, A. I. Shienok. *Bulletin of the Academy of Sciences of the USSR, Division of chemical science*, **1982**, *31(2)*, 340-341.

[SI12] S. Stoll, A. Schweiger, *J. Magn. Reson.* **2006**, *178*, 42-55.

[SI13] Y. Kitagawa, T. Saito, Y. Nakanishi, Y. Kataoka, T. Matsui, T. Kawakami, M. Okumura, K. Yamaguchi, *J. Phys. Chem. A* **2009**, *113*, 15041–15046.

[SI14] TURBOMOLE, Version 7.5, 2020; University of Karlsruhe and Forschungszentrum Karlsruhe GmbH 1989–2007, TURBOMOLE GmbH since 2007; https://www.turbomole.org.

[SI15] a) P. A. M. Dirac, *Proc. Royal Soc. London A* **1929**, *123*, 714–733; b) J. C. Slater, *Phys. Rev.* **1951**, *81*, 385–390; c) J. P. Perdew, Y. Wang, *Phys. Rev. B* **1992**, *45*, 13244–13249; d) J. P. Perdew, K. Burke, M. Ernzerhof, *Phys. Rev. Lett.* **1996**, *77*, 3865–3868; e) J. P. Perdew, M. Ernzerhof, K. Burke, *J. Chem. Phys.* **1996**, *105*, 9982–9985.

[SI16] F. Weigend, R. Ahlrichs, *Phys. Chem. Chem. Phys.* **2005**, *7*, 3297–3305.

[SI17] ORCA, Version 4.0.1.2, 2017; a) F. Neese, *Wiley Interdiscip. Rev. Comput. Mol. Sci.* **2012**, *2*, 73–78; b) F. Neese, *Wiley Interdiscip. Rev. Comput. Mol. Sci.* **2018**, *8*, e1327.
